# Mitochondrial metabolism determines chemotherapy sensitivity in colorectal cancer

**DOI:** 10.1038/s42255-026-01578-w

**Published:** 2026-07-23

**Authors:** Deborah Y. Moss, Connor N. Brown, Andrew M. Shaw, Christopher McCann, Nikita Lewis, Matilda Downs, Rebecca Wurelly, Ciara Cunningham, Aaron Philips, Niamh Doherty, Sarah Gallagher, William J. McDaid, Andrew Roe, Aisling Y. Coughlan, Brenton Cavanagh, Callum Ormsby, Fiammetta Falcone, Rachel McCole, Scott Monteith, Emily Rogan, Sudhir B. Malla, Alexandra J. Emerson, Letitia Mohammed-Smith, Shaun Sharkey, Aoife Leonard, Peter F. Gallagher, Arindam Banerjee, Sandra Van Schaeybroeck, Sufyan Pandor, Brian Quinn, Brett Greer, Christopher Elliott, Sarah Maguire, Aideen E. Ryan, Philip D. Dunne, Mary Mallon, Stephanie Craig, Omar Aftab, Laura C. Greaves, Bryan P. Marzullo, Daniel A. Tennant, Vicky Coyle, Ian G. Mills, Owen Sansom, Tríona Ní Chonghaile, Daniel B. Longley, Simon S. McDade, Melissa J. LaBonte, Emma M. Kerr

**Affiliations:** 1 Johnston Cancer Research Centre, Queen’s University Belfast, Belfast, Northern Ireland UK; 2 Physiology and Medical Physics, Royal College of Surgeons in Ireland, Dublin, Ireland; 3 Cellular and Molecular Imaging Core, Royal College of Surgeons in Ireland, Dublin, Ireland; 4 Agilent Technologies, Cheadle, UK; 5 Institute for Global Food Security, Queen’s University Belfast, Belfast, Northern Ireland UK; 6 Agri-Food and Biosciences Institute, Chemical Surveillance Branch, Veterinary Sciences Division, Belfast, Northern Ireland UK; 7 International Joint Research Centre on Food Security (IJC-FOODSEC), Khong Luang, Thailand; 8 School of Food Science and Technology, Faculty of Science and Technology, Thammasat University, Khong Luang, Thailand; 9 Discipline of Pharmacology and Therapeutics, School of Medicine, College of Medicine, Nursing and Health Sciences, University of Galway, Galway, Ireland; 10 CRUK Scotland Institute, Glasgow, UK; 11 Biosciences Institute, Newcastle University, Newcastle Upon Tyne, UK; 12 School of Cancer Sciences, University of Glasgow, Glasgow, UK; 13 Department of Metabolism and Systems Science, College of Medicine and Health, University of Birmingham, Birmingham, UK

**Keywords:** Cancer metabolism, Cancer, Metabolism, Chemotherapy, Cancer therapeutic resistance

## Abstract

Therapy resistance is attributed to over 80% of cancer deaths per year, emphasizing the urgent need to overcome this challenge for improved patient outcomes. Despite its widespread use in colorectal cancer (CRC) treatment, resistance to 5-fluorouracil (5FU) remains poorly understood. As an antimetabolite, 5FU imposes substantial metabolic stress, forcing cells that survive treatment to rapidly adapt. We explored acute 5FU-driven changes in mitochondria, the organelle critical for coordinating metabolic stress responses. Here we demonstrate in a range of CRC models that 5FU treatment promotes mitochondrial biogenesis and increases mitochondrial function in surviving cells. Furthermore, we show that targeting mitochondrial metabolism, particularly by inhibiting Complex I, sensitizes CRC cells to 5FU, resulting in delayed tumour growth and prolonged survival in preclinical models. Additionally, analysis of patient data suggests that oxidative metabolism signatures may predict responses to 5FU-based chemotherapy. These findings shed light on mechanisms underlying 5FU resistance and propose a rational strategy for combination therapy in CRC, emphasizing the potential clinical benefit of targeting mitochondrial metabolism to overcome resistance and enhance patient outcomes.

## Abstract (web summary)

Mitochondrial oxidative metabolism is found to strongly affect the response of colorectal cancer (CRC) cells to the chemotherapeutic drug 5-fluorouracil (5FU). Targeting mitochondrial metabolism sensitizes CRC cells to 5FU.

## Main

Therapy resistance is a major clinical problem, limiting the effectiveness of cancer treatments. Resistance is promoted by a range of factors that are either present in the tumour before therapy (described as intrinsic resistance) or selected for during therapeutic exposure (termed acquired resistance). These factors can stem from the tumour cells themselves, the surrounding tumour microenvironment or systemic influences^1^. Understanding the molecular determinants of resistance and using this to guide rational combination therapy approaches is fundamental to improve clinical outcomes for patients with cancer.

Since its discovery in the 1950s, the antimetabolite 5FU has been the cornerstone of fluoropyrimidine-based chemotherapy treatment for patients with CRC. Given as a single agent, it promotes modest increases in survival, and these effects are enhanced when combined with oxaliplatin and/or irinotecan (FOLFOX, FOLFIRI)^2,3^. However, despite being the mainstay of CRC chemotherapy treatment, the primary anti-tumour mechanisms—and more importantly, mechanisms facilitating 5FU resistance and persistence—remain poorly defined. The multifaceted mechanism of action of 5FU involves direct incorporation of its metabolites into DNA (FdUTP) and RNA (FUTP), as well as inhibition of thymidylate synthase (TS) via FdUMP^4,5^, making it more difficult to identify rational combination partners that would enhance clinical responses without eliciting adverse systemic toxicity.

Reprogramming of cancer cell energetics is fundamental to tumour progression and spread^6,7^ but is also critically linked to therapeutic efficacy^8^. Targeting such metabolic programmes presents novel opportunities to enhance drug responses and improve patient outcomes^9^. Mitochondria are unique organelles, coordinating metabolic and apoptotic crosstalk to tightly regulate cell survival^10^. The ability of mitochondria to rapidly adapt to stress, including stress imposed by antimetabolites such as 5FU, means that they have significant potential to dictate therapeutic responses^11^. Therefore, a better understanding of the mitochondrial metabolic programmes critical in supporting cancer cell survival following 5FU treatment is required to rationally design more effective fluoropyrimidine-based chemotherapy combination regimens. Our goal was to focus on how mitochondria facilitate the acute stress response to 5FU in cells that tolerated treatment (that is, were not sensitive enough to treatment to die).

## Results

### 5FU treatment alters mitochondrial biogenesis and dynamics in cancer cells

**Fig. 1:**
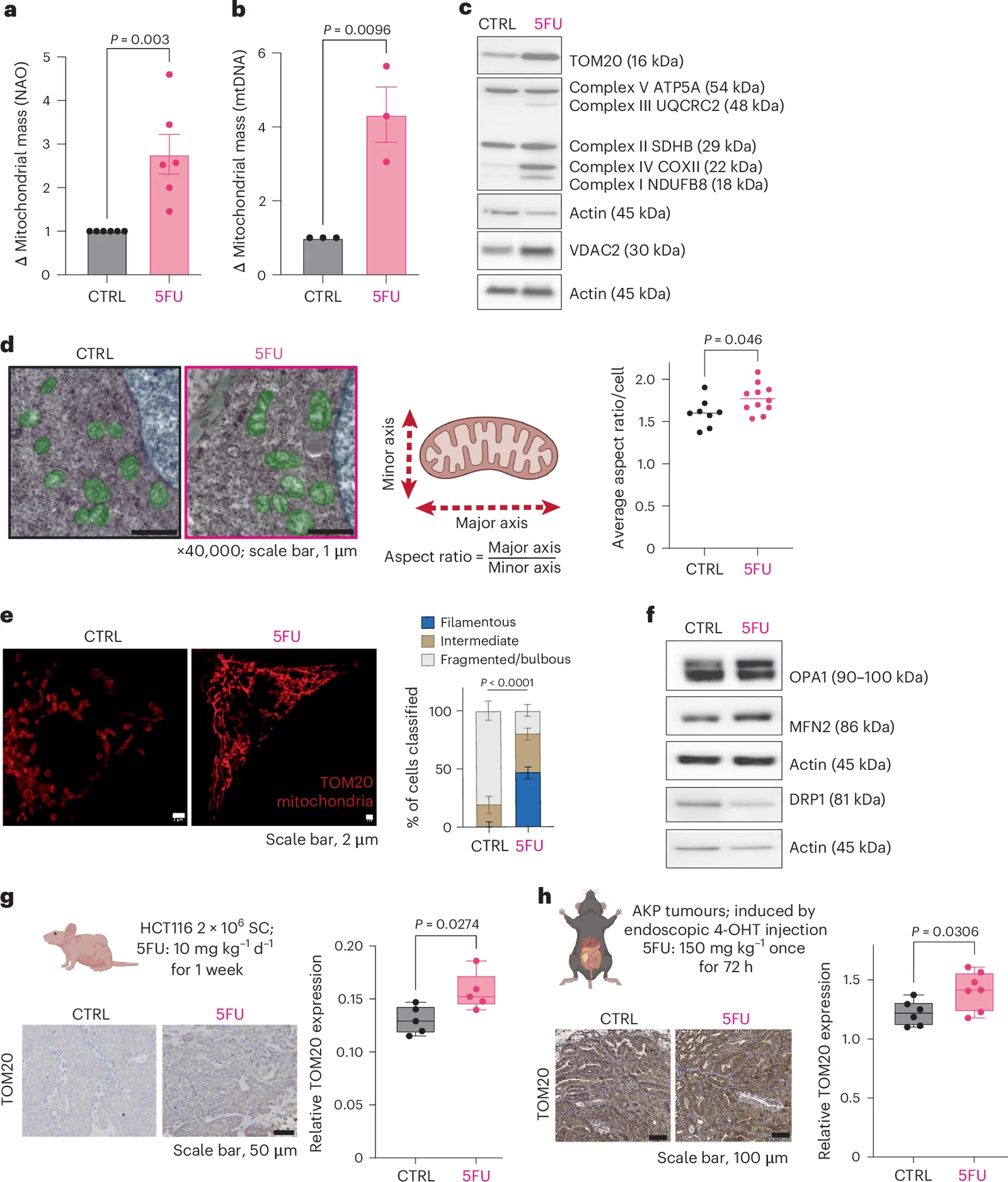
5FU treatment drives metabolic adaptation in CRC models. **a**,**b**, Mitochondrial mass in HCT116 cells treated with or without 5FU (±5FU) for 72 h, measured by NAO staining (**a**) or mtDNA content (**b**). Data points show biological replicates from triplicate means, normalized to mean control (CTRL) from technical replicates. Bars show biological means; error bars, s.e.m. **c**, Western blot analysis of HCT116 ±5FU (72 h) protein lysates for mitochondrial proteins. Representative blots of *n* = 5 are shown. **d**, Transmission electron microscopy images of HCT116 cells ±5FU, with the measurements taken from visible mitochondria to calculate aspect ratio illustrated. Each data point shows the cell mean of all mitochondria per individual cell, with eight cells (CTRL) or 11 cells (5FU) quantified; overall mean shown as line. **e**, confocal microscopy images of TOM20 staining in HCT116 ±5FU for 72 h, with network morphology quantified by classification. *n* = 3, with mean ± s.e.m. for each shown. **f**, Western blot analysis of HCT116 ±5FU (72 h) protein lysates for mitochondrial dynamics. Representative blots shown of *n* = 3 (DRP1) or *n* = 5 (OPA1/MFN2). **g**,**h**, Immunohistochemistry images of TOM20 staining in HCT116 xenografts (**g**; *n* = 5 mice) and AKP orthotopic colon tumours (**h**; CTRL *n* = 6 and 5FU *n* = 7 mice) treated ±5FU as indicated, with quantification of TOM20 expression by QuPath represented by box and whisker plots showing the mean value (central marker) and the minimum–maximum range of values (whiskers). 4-OHT, 4-hydroxytamoxifen. *P* values calculated by two-tailed *t*-test (**a**, **b**, **d**, **g**, **h**) and two-way ANOVA (**e**). Source data

**Extended Data Fig. 1:**
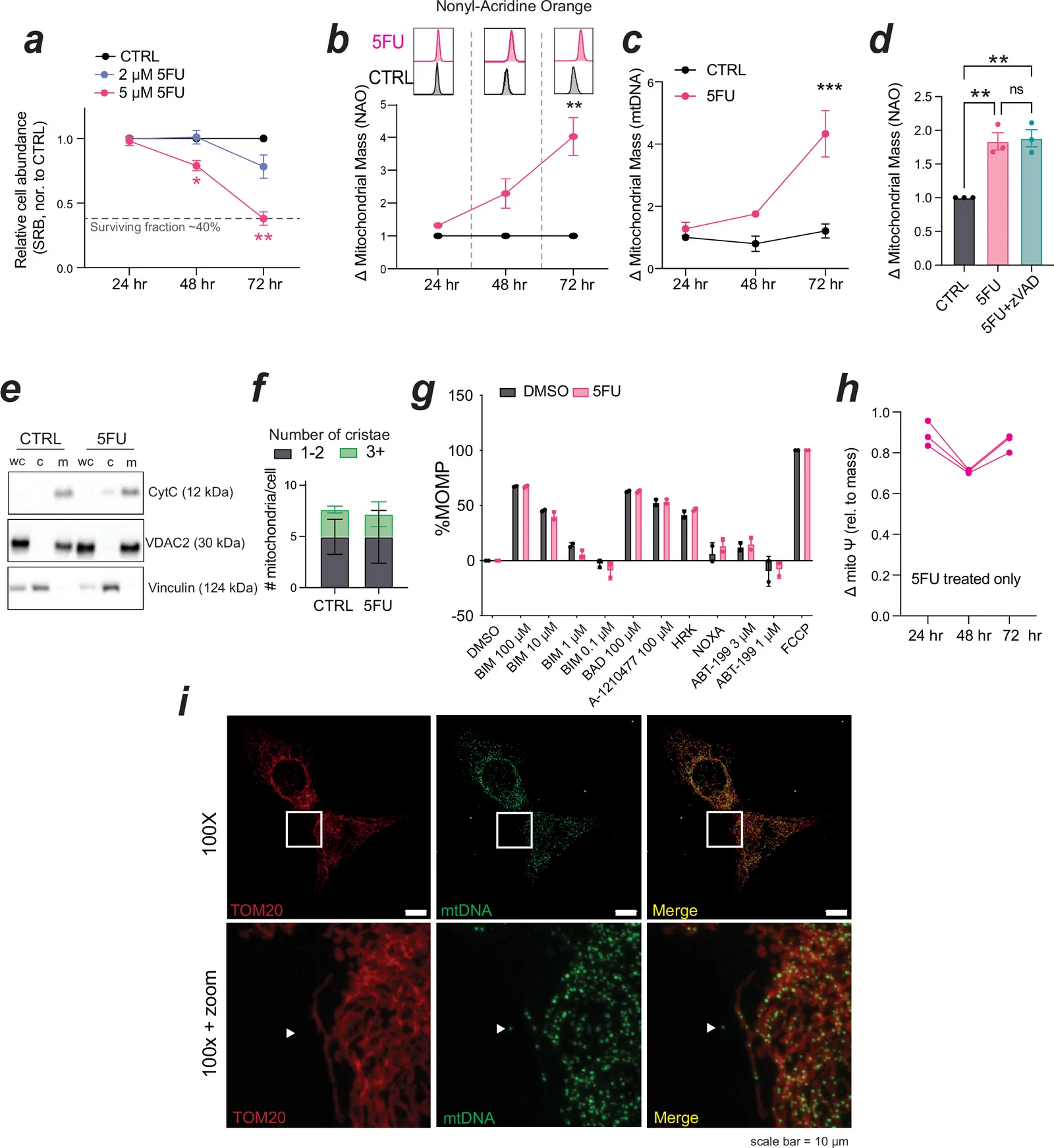
5FU treatment increased mitochondrial mass and activity independent of apoptosis. **a**) Change in cell growth in HCT116 cells treated with 5FU as indicated over 72 h measured by SRB assay. Data shows mean of *n* = 4 experiments ± s.e.m; Change in mitochondrial mass in HCT116 cells treated ± 5 μM 5FU over 72 h measured by nonyl-acridine orange (NAO) staining (**b**) or mitochondrial DNA content (**c**), data point shows biological mean, ± s.e.m. of *n* = 3 experiments; (**d**) Change in mitochondrial mass measured by NAO staining of HCT116 cells treated ± 5FU or 5FU + z-VAD-fmk. Data points show 3 biological replicates ± s.e.m; (**e**) protein lysates from whole cell (wc), cytosolic (c) and mitochondrial (m) fractions from HCT116 cells treated ± 5FU for 72 h. Representative blots of *n* = 3 shown; (**f**) Graph illustrating number of mitochondria analysed per cell, with *cristae* number noted, mean ± s.d.; (**g**) Degree of mitochondrial outer membrane permeabilization (% MOMP) in HCT116 cells ± 5FU when treated with compounds as indicated. Data shows 2 biological replicates ± s.e.m.; (**h**) Change in mitochondrial membrane potential, calculated by degree of TMRE staining, relative to mitochondrial mass (**b**) at each timepoint for HCT116 cells treated with 5FU, n = 3 biological replicates; (**i**) Confocal images of mitochondria (imaged by TOM20 staining) and mtDNA in cells treated with 5FU for 72 h. Images taken at 100x, with zoom applied. Scale = 10 μm, mtDNA release indicated by white arrow. Images representative of n = 2. P values indicated, calculated by two-way ANOVA with Tukey’s (**a**) or Šídák’s multiple comparisons test (**b,c**), and one-way ANOVA with Dunnett’s multiple comparisons test (**d**). Source data

Treatment with 5FU causes modest cell cycle arrest (Extended Data Fig. 1a), and a significant fraction of cells survive (~40% with 5 μM at 72 h). To determine the contribution of the mitochondria to this acute 5FU tolerance, we explored multiple mitochondrial phenotypes. We first examined the impact of 5FU treatment on mitochondrial abundance in the well-characterized CRC cell model HCT116. In cells that survive treatment, 5FU promotes a sustained increase in mitochondrial content over 72 h, as shown by changes in levels of nonyl-acridine orange (NAO) staining (Fig. 1a and Extended Data Fig. 1b), mitochondrial DNA (mtDNA relative to total genomic DNA per cell; Fig. 1b and Extended Data Fig. 1c) and mitochondrial protein expression, including TOM20, VDAC2 and electron transport chain (ETC) complex proteins (Fig. 1c) when compared to PBS control. Importantly, these changes in mitochondrial mass are not related to engagement of apoptosis: inhibition of caspase activity using zVAD-fmk did not prevent increases in mitochondrial abundance per cell (Extended Data Fig. 1d), and 5FU treatment did not promote cytochrome *c* release from mitochondria (Extended Data Fig. 1e). Using microscopy, we analysed the effects of 72 h 5FU treatment on mitochondrial morphology to identify potential organelle dysfunction. Upon examination of mitochondria by electron microscopy, both the 5FU-treated and control groups exhibited comparable mitochondrial structures. There was no noticeable swelling or rupture, and the appearance and abundance of cristae were consistent between the two groups (as illustrated in Fig. 1d and Extended Data Fig. 1f). However, looking at the aspect ratio (defined as the ratio of the major to minor axes lengths), we observed an elongation of the mitochondria (Fig. 1d), which is indicative of a more active organelle^12^. We next explored mitochondrial networks visualized by TOM20 staining and confocal microscopy. Treatment with 5FU resulted in the reorganization of mitochondrial networks into filamentous structures (Fig. 1e). These changes were accompanied by altered expression of the proteins that regulate mitochondrial morphology^13^. We observed an increase in OPA1 expression; specifically, increased expression of the upper band (ca. twofold; *P* = 0.0026, with upper to lower ratio increasing from 0.89 to 2.77), indicating a more active protein^14^, an increase in mitofusin 2 (ca. 1.7 fold; *P* = 0.0054), both of which control mitochondrial fusion, and a decrease in fission protein DRP1 expression (ca. threefold; *P* = 0.0216) 72 h post 5FU treatment (Fig. 1f), supporting changes in mitochondrial morphology and network formation. Supporting our cytochrome *c* assays, we confirmed no change in mitochondrial outer membrane permeabilization (MOMP) in cells surviving 5FU treatment (compared to the dimethyl sulfoxide group), and no increase in mitochondrial apoptotic priming based on BH3-profiling^15,16^ induced by 5FU treatment (Extended Data Fig. 1g). This finding was corroborated by observation of a modest transient dip in mitochondrial membrane potential at 48 h that fully recovered by 72 h (Extended Data Fig. 1h). Lastly, we confirmed that cells surviving 5FU treatment do not increase mtDNA content to compensate for overwhelming mtDNA release (Extended Data Fig. 1i).

**Extended Data Fig. 2:**
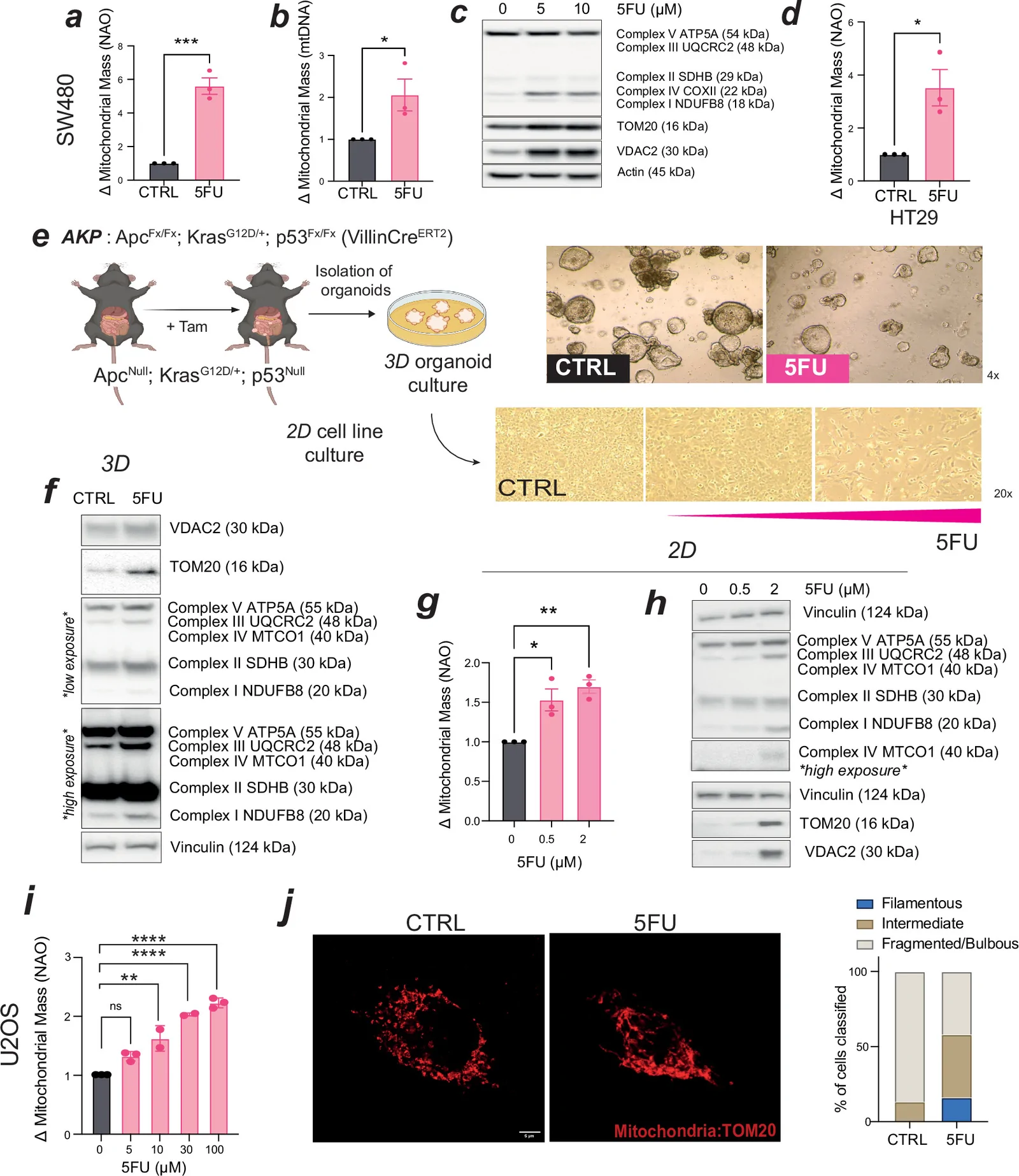
5FU drives metabolic adaptation in tumour cells. SW480 cells treated ± 5FU for 72 h analysed for mitochondrial mass by NAO staining (**a**) and mtDNA (**b**), n = 3 biological replicates shown with mean ± s.e.m in both, and mitochondrial protein expression (**c**), blots representative of n = 3 biological replicates; (**d**) NAO staining of mitochondrial mass in HT29 cells treated ± 5FU for 72 h, n = 3 biological replicates, with mean ± s.e.m. shown; (**e**) Schematic of *AKP* organoid isolation and culture (3D and 2D cell line) with representative images of 3D or 2D cultures treated ± 5FU at 72 h; (**f**) protein expression in lysates from 3D *AKP* organoids treated ± 5FU for 72 h, representative of n = 3 biological replicates; mitochondrial mass by NAO (**g**, *n* = 3 biological replicates; with mean ± s.e.m. shown) and protein expression in lysates (**h**) from 2D *AKP* cells treated ± 5FU for 72 h; Mitochondrial mass by NAO staining (**i**, n = 3 biological replicates) and mitochondrial imaging by TOM20 staining (representative images from n = 2) (**j**) in U2OS cells treated ± 5FU for 72 h, with mean ± s.e.m. shown. Representative blots of *n* = 3 shown. P values indicated, calculated by two-tailed *t*-test (**a,b,d**) or one-way ANOVA with Dunnett’s multiple comparisons test (**g,i**). Source data

HCT116 cells are an extensively used human colorectal model that carry a *KRAS* G13D mutation but retain wild-type *TP53* and *APC*^17^. KRAS signalling has been linked to enhanced mitochondrial metabolism by various mechanisms in colorectal, lung and pancreatic cancers^8,18^, as has APC and p53 (refs. ^11,19^). Therefore, we next tested these 5FU-driven mitochondrial adaptations across a range of cancer models. We tested human CRC cell models SW480 (a *KRAS* mutant, *TP53* mutant MSS line; Extended Data Fig. 2a–c) and HT29 (a *PIK3CA* and *BRAF* mutant CIN-high cell line; Extended Data Fig. 2d). Murine CRC organoids that were isolated from Vil-CreER^T2^; *Apc*^fl/fl^; *Kras*^G12D/+^; *Trp53*^fl/fl^ (AKP) mice 72 h post-tamoxifen intraperitoneal (i.p.) injection and cultured in 3D as previously reported (Extended Data Fig. 2e)^20,21^ or as a 2D cell line model also recapitulated findings from human CRC lines (Extended Data Fig. 2f–h). We also tested a non-CRC model, the osteosarcoma U2OS cell line, which is wild-type for APC, KRAS and p53, and confirmed a similar 5FU-driven change in mitochondrial mass and morphology (Extended Data Fig. 2i,j), suggesting that this response is independent of oncogenic driver mutation and culture conditions^22^.

**Extended Data Fig. 3:**
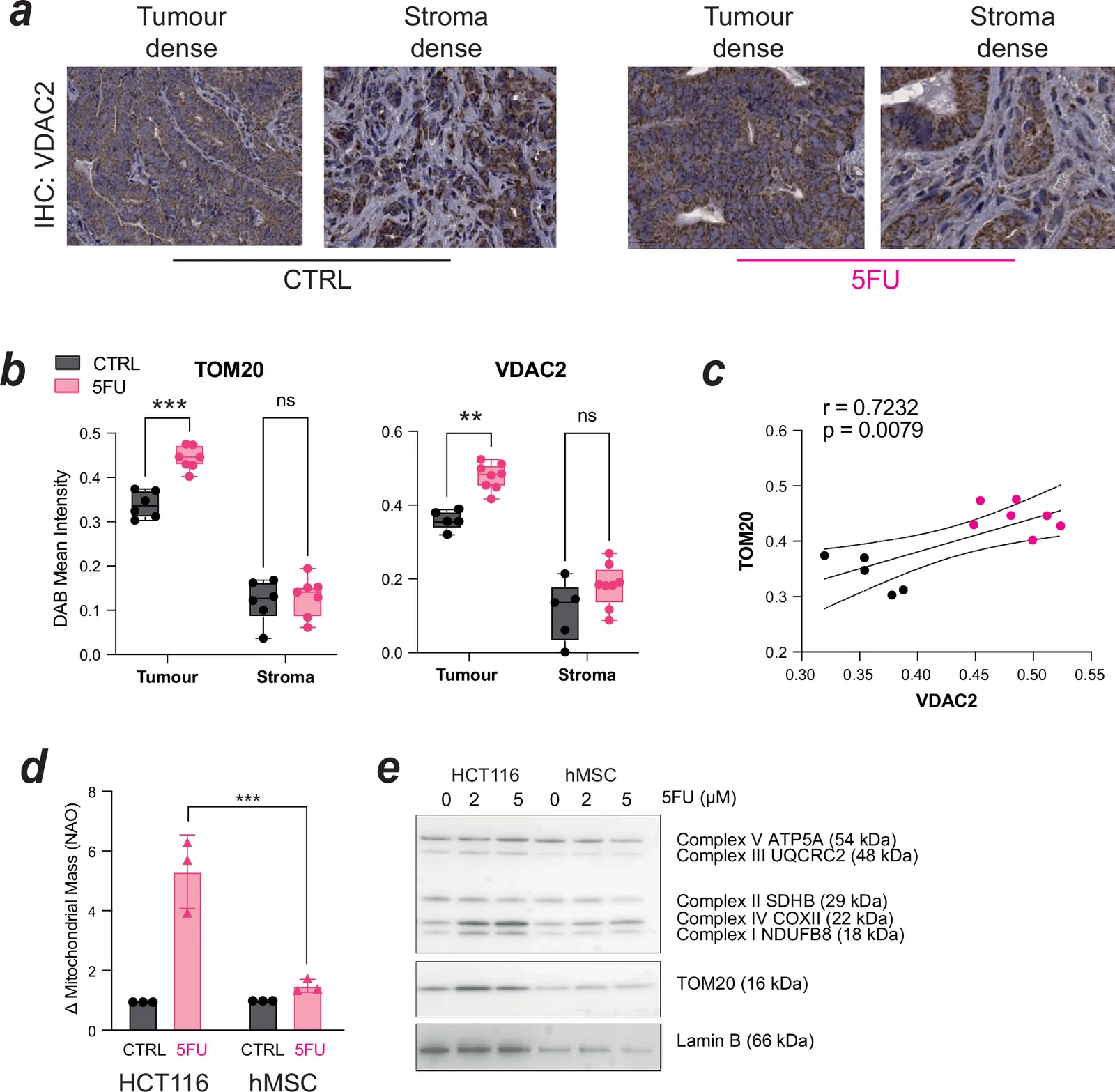
5FU drives increases in mitochondrial content specifically in tumour cells. Mitochondrial imaging in *AKP* tumours treated ± 5FU (as per Fig. 1h) after 72 h. (**a**) VDAC2 IHC images from tumour dense and stromal dense areas, scale bar = 50um; (**b**) QuPath quantification of TOM20 and VDAC2 in tumour and stromal segments within *AKP* tumours ± 5FU, where each dot represents mean staining per mouse. Data are presented as box and whisker plots showing the mean value (central marker) and the minimum-maximum range of values (whiskers); (**c**) Pearsons correlation of TOM20 and VDAC2 expression in tumour areas, where each dot represents mean staining per mouse. Pearsons’ *r* = 0.7232 and *P* = 0.0079; Mitochondrial mass by NAO staining (**d**, n = 3 biological replicates, with mean ± s.e.m. shown) or mitochondrial protein expression in lysates (**e**) from HCT116 cells or human mesenchymal stromal cells (hMSC) treated ± 5FU for 72 h. Representative blots of *n* = 3 shown. P values indicated, calculated by two-way ANOVA with Šídák’s multiple comparisons test (**b, d**). Source data

We next sought to examine this mitochondrial biogenesis phenotype in whole-tumour ecosystems to ensure that our observations were not linked to a culture phenomenon, as has previously been described for other metabolic reprogramming studies^23^. First, using an HCT116 xenograft model, we show that repeated 5FU treatment (10 mg kg^−1^ d^−1^ for 7 days) promoted increased TOM20 abundance (Fig. 1g). In a more complex murine CRC model, we used orthotopic endoscopy-guided submucosal injection^24–26^ of 4-hydroxytamoxifen to drive allelic recombination in AKP mice and promote tumour formation. Tumours were allowed to develop for 5–6 weeks until mice showed signs of tumour burden, at which point mice were randomized and treated with a single i.p. injection of 150 mg kg^−1^ 5FU or PBS vehicle for 72 h. This single 5FU treatment, mimicking the single dose in vitro, was sufficient to drive increased TOM20 expression (Fig. 1h). TOM20 staining patterns in AKP GEMM tumours suggested compartment differences (tumour vs stroma); therefore, we used VDAC2 staining as a second marker of mitochondrial abundance, and quantified staining in tumour or stromal compartments using QuPath software^27^ (Extended Data Fig. 3a). The 5FU treatment resulted in changes in mitochondrial markers in tumour but not stromal areas of AKP tumours (Extended Data Fig. 3b). Furthermore, there was a significant correlation in both markers used in tumour areas (Extended Data Fig. 3c), confirming that these are good representations of mitochondrial abundance. To confirm whether this change is cancer-cell-specific, we tested human mesenchymal stromal cells (hMSCs) representative of cancer stroma^28^. Here, we see that 5FU can induce changes in mitochondrial abundance in HCT116 cancer cells but fails to induce similar changes in hMSCs, evidenced by NAO staining and protein expression (Extended Data Fig. 3d,e).

Taken together, these data demonstrate that cancer cells capable of enduring 5FU treatment have significant, sustained mitochondrial adaptations, driving enhanced mitochondrial biogenesis and fusion in the absence of evident mitochondrial dysfunction. This response is independent of driver oncogene and environmental conditions but specific to cancer cells, both in vitro and in vivo, further highlighting the inherent mitochondrial plasticity in cancer.

### 5FU adaptation alters mitochondrial activity

**Fig. 2:**
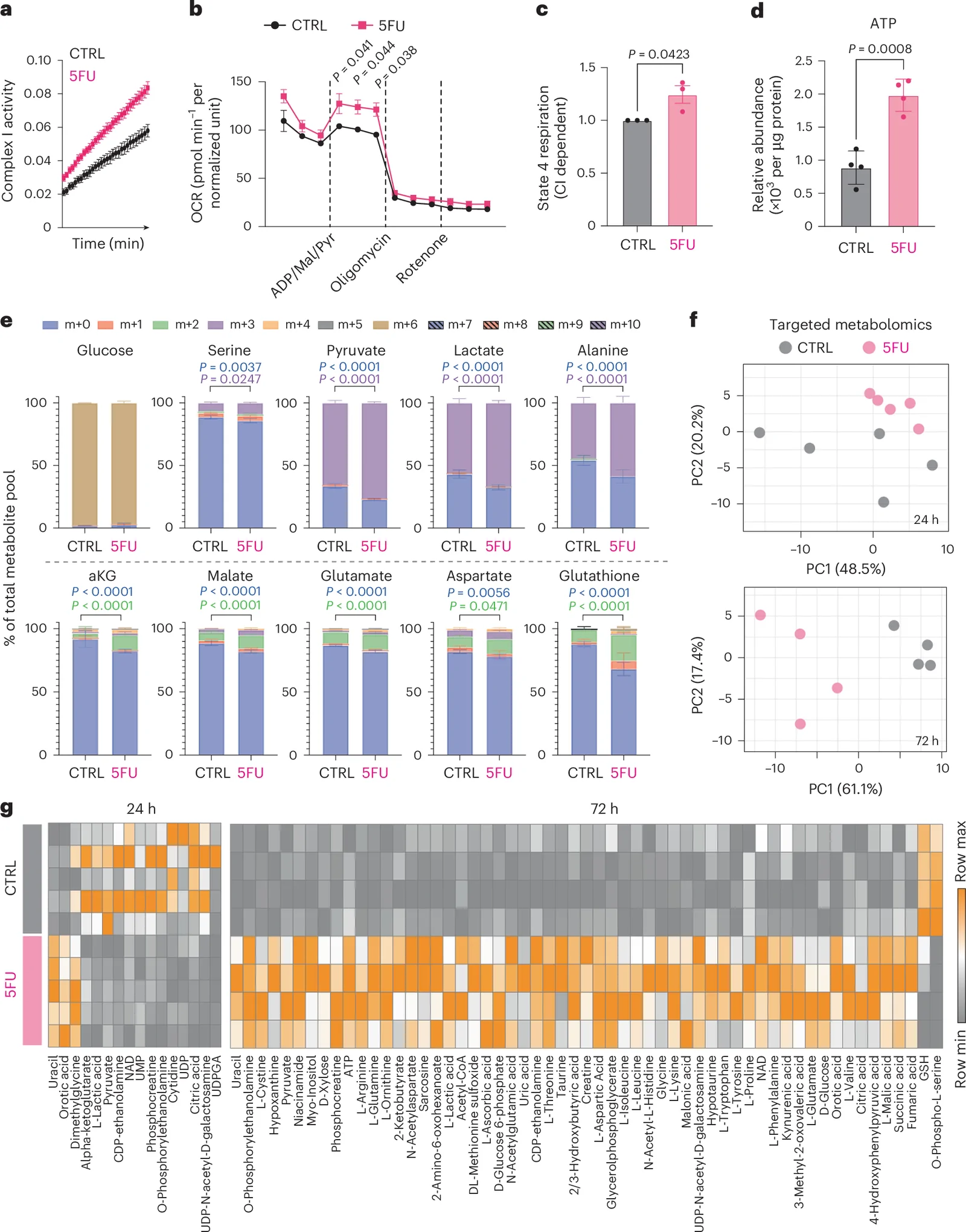
5FU treatment reprograms metabolic activity. **a**, CI kinetic activity assay in HCT116 cells treated ±5FU. Data points show the mean of technical replicates; error bars, s.d. Representative data are shown from three independent experiments. **b**,**c**, OCR measured by extracellular flux analysis in cells treated ±5FU for 72 h, at baseline, post CI substrates (ADP/malate/pyruvate), post oligomycin and post rotenone (**b**), with CI-dependent respiration calculated (**c**). Data show biological mean ± s.e.m. of *n* = 3 (**b**) or individual biological replicates as data points with mean ± s.e.m. **d**, Quantification of relative ATP abundance by LC–MS in cells treated ±5FU for 72 h. Data show technical replicates; mean ± s.d. **e**, LC–MS metabolomics analysis of labelling in metabolites downstream of ^13^C_6_-glucose from HCT116 cells ±5FU. Isoform abundance as percentage (%) of total pool is indicated in stacked bar charts; data show mean ± s.d. of five replicates, representative of two individual experiments. **f**, PCA plots of metabolite abundance analysis by targeted LC–MS metabolomics in cells treated ±5FU at 24 h (*n* = 5 replicates per group) and 72 h (*n* = 4 replicates per group). **g**, Relative abundance of differential metabolites ±5FU, identified by *t*-test, with multiple testing correction. *P* values indicated were calculated by multiple *t*-tests (**a**,**b**,**g**), two-tailed *t*-tests (**c**,**d**) and two-way ANOVA with Šídák’s multiple comparisons test (**e**). Source data

**Extended Data Fig. 4:**
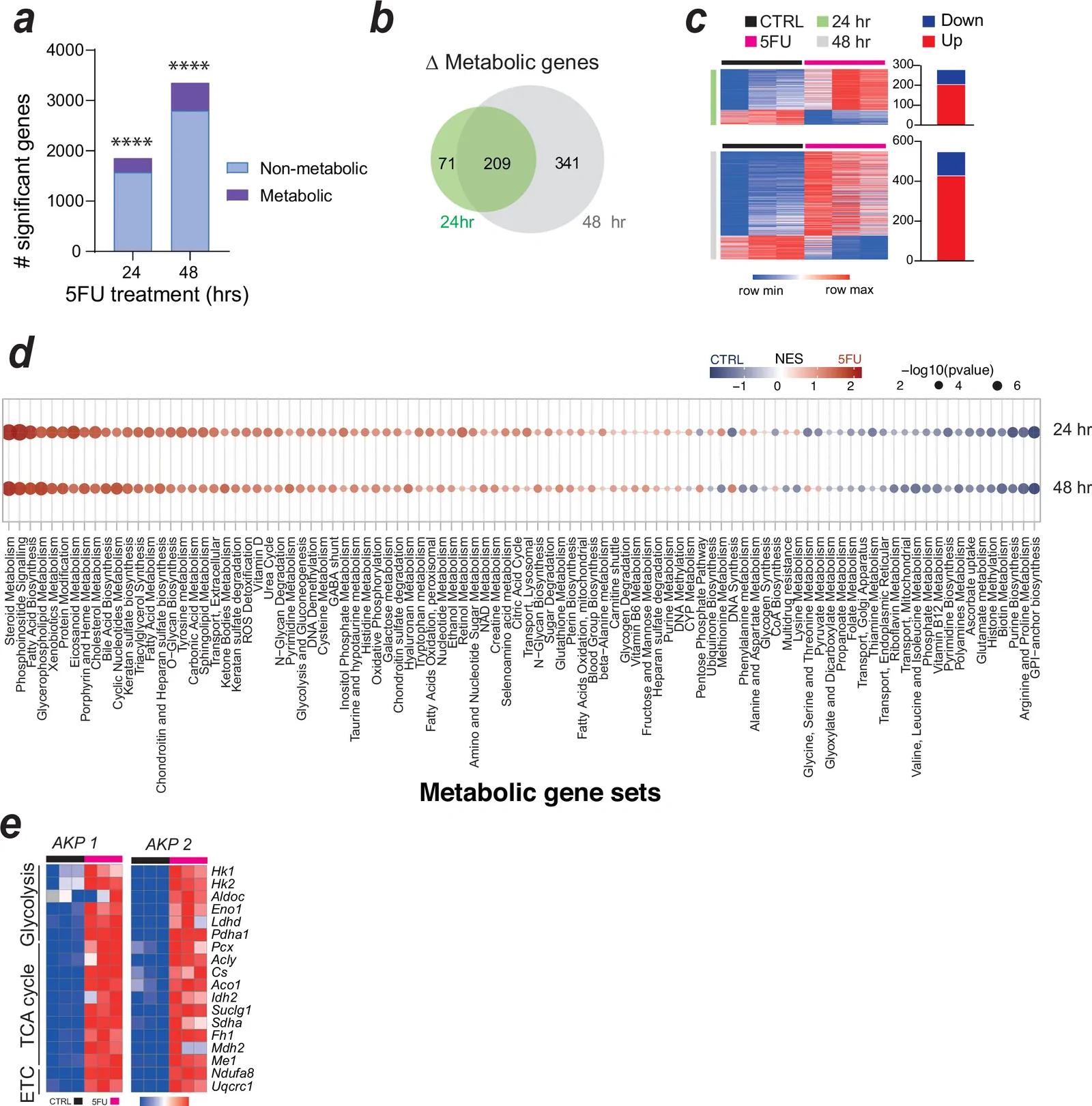
5FU treatment increases metabolic gene expression. (**a**) Number of significant genes (defined as adj *P* < 0.05) following DESeq2 analysis of transcriptional data (RNA-seq) from HCT116 cells treated for 24 and 48 h with 5FU. Metabolic genes (defined in^10,51^) are indicated, with over-representation significance (****P < 0.0001) calculated by Fisher’s exact test (two-sided); (**b**) Metabolic genes from (**a**) at 24 and 48 h; (**c**) Heatmaps and summary bar charts of metabolic gene expression patterns of genes from (**b**); (**d**) GSEA analysis of data from (**b, c**) run against metabolic gene sets (defined in^10^); (**e**) relative gene expression changes in two independent *AKP* organoid lines cultured in 3D ± 5FU for 48 h. Source data

Given the changes in mitochondrial abundance, without evident mitochondrial dysfunction (no cristae clearing, no change in apoptotic priming, no release of cytochrome *c* or mtDNA), we reasoned that the increase might be associated with increased functionality. Mitochondria are the site of a range of chemical reactions, including the tricarboxylic acid (TCA) cycle and ETC, and are responsible for the generation of electron carriers, adenosine triphosphate (ATP) and other biosynthetic molecules^29^. We first tested Complex I (CI) activity as the entry point for ETC flux, using a commercial activity assay (Fig. 2a) and published Seahorse Extracellular Flux (XF) protocols^30,31^ (Fig. 2b,c). Both readouts show that 72 h 5FU treatment increases mitochondrial CI activity. We then tested ATP abundance by liquid chromatography–mass spectrometry (LC–MS) and show increased ATP levels in cells that survive 5FU treatment (Fig. 2d). We next explored the broader metabolic impact of 5FU in these drug-tolerant cells. Using ^13^C_6_-glucose labelling, we see increased incorporation of labelled glucose into a range of metabolites (Fig. 2e), including those related to glycolysis (pyruvate, lactate), TCA cycle (α-ketoglutarate, malate) and redox metabolism (reduced glutathione), suggestive of altered mitochondrial metabolism. Targeted metabolomics analysis shows that 5FU treatment promotes significant changes in metabolite abundance over time (Fig. 2f), with a vast increase in metabolite abundance 72 h post treatment (Fig. 2g), correlating with increased mitochondrial abundance and activity. This broad metabolic reprogramming is supported by an increase in transcription of metabolic genes. Differential gene expression analysis using the DESeq2 package identified 1,856 and 3,350 differentially expressed genes (DEGs; adjusted *P* < 0.05) with 24 h and 48 h 5FU treatment, respectively (Extended Data Fig. 4a). When DEGs were cross-referenced to a metabolic gene list (2,921 genes) compiled from previously published studies^6,32^, we observed significant overrepresentation of metabolic DEGs, as determined by Fisher’s exact test (Extended Data Fig. 4a). Interestingly, the pattern of change in these metabolic genes (280 and 550 at 24 h and 48 h, respectively; Extended Data Fig. 4b) suggests that the metabolic response is mounted quickly but sustained and enhanced over time, given that ~75% of the metabolic DEGs at 24 h are also found at 48 h (Extended Data Fig. 4b). Further examining the change in gene expression revealed that an extensive number of these metabolic genes are increased in expression both at 24 h and 48 h (Extended Data Fig. 4c). Analysis of curated metabolic gene sets (Extended Data Fig. 4d) suggests significant changes in the expression of genes related to a range of metabolic signalling networks, including lipid, protein, nucleotide, carbon and reactive oxygen species metabolism. We confirmed these transcriptional changes in murine AKP organoid models treated with 5FU (Extended Data Fig. 4e).

Therefore, we conclude that 5FU promotes metabolic reprogramming in drug-tolerant cells, increasing mitochondrial activity supported by an acute increase in metabolic gene transcription.

### TS inhibition by 5FU regulates mitochondrial biogenesis through mTOR–PGC1α signalling

**Fig. 3:**
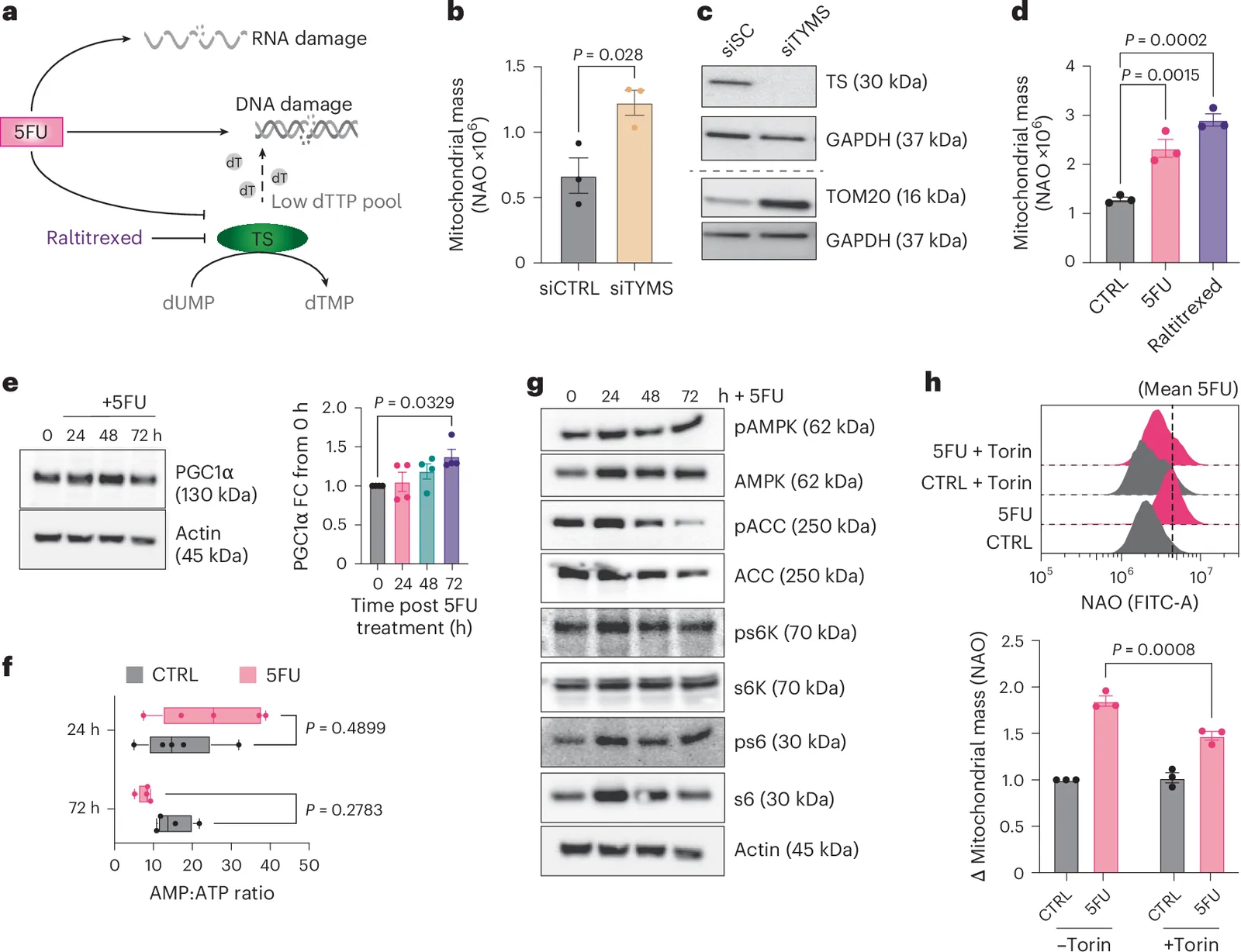
5FU treatment increases mitochondrial mass via TS inhibition and mTORC1 signalling. **a**, Schematic showing impact of 5FU and raltitrexed treatment. **b**,**c**, Mitochondrial mass assessed by NAO staining (**b**, *n* = 3 biological replicates; mean ± s.e.m.) or TOM20 expression in protein lysates (**c**) from HCT116 cells transfected with siRNA against CTRL or *TYMS*. Representative blots of *n* = 3 shown. **d**, NAO staining (*n* = 3 biological replicates; mean ± s.e.m.) of mitochondrial mass in cells treated with 5FU or raltitrexed for 72 h. **e**, PGC1α expression in protein lysates from cells treated ± 5FU over 72 h relative to actin loading control. Representative blots of *n* = 4 shown, with four replicates quantified by densitometry; mean ± s.e.m. FC, fold change. **f**, AMP:ATP metabolite abundance ratios in cells treated ±5FU for 24 h and 72 h, represented by box and whisker plots showing the mean value (central marker) and the minimum–maximum range of values (whiskers). **g**, protein expression in lysates from HCT116 cells treated ± 5FU over 72 h, relative to actin loading control. Representative blots of *n* = 3 shown. **h**, Representative staining histograms and geometric mean of NAO staining in cells treated ±5FU or ±Torin for 72 h. Data show biological replicates (mean ± s.e.m.; **b**, **d**, **h**) or technical replicates (mean ± s.d.; **f**). *P* values indicated were calculated by *t*-test (**b**), one-way ANOVA with Dunnett’s multiple comparisons test (**d**) or two-way ANOVA with Šídák’s multiple comparisons test (**f**, **h**). Source data

**Extended Data Fig. 5:**
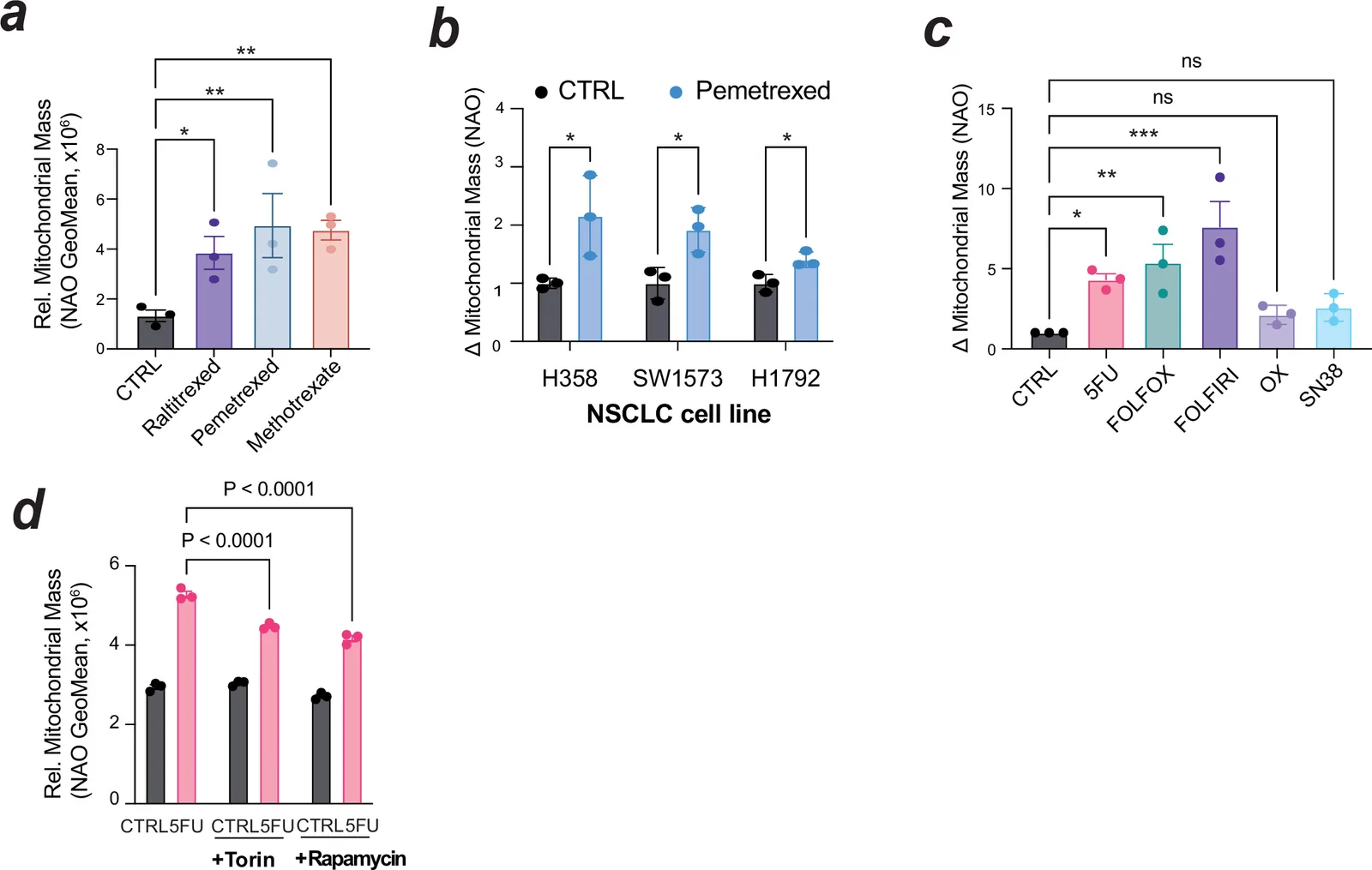
Thymidylate Synthase inhibition specifically increases mitochondrial mass in an mTOR dependent manner. Mitochondrial mass assessed by NAO staining in (**a**) HCT116 cells treated with TS-targeting therapies, (**b**) non-small cell lung cancer (NSCLC) cell lines treated with pemetrexed, (**c**) HCT116 cells treated with 5FU, FOLFOX (5FU+oxaliplatin), FOLFIRI (5FU + SN38), or single agent oxaliplatin and SN38, or (**d**) HCT116 cells treated ± 5FU ± Torin or rapamycin for 72 h. Data shows biological (**a,c**) or technical (**b,d**) replicates, with mean ± s.e.m. (**a,c**) or s.d. (**b,d**). P values indicated, calculated by one-way ANOVA with Dunnett’s multiple comparisons test (**a,c**), two-tailed *t*-test (**b**) or two-way ANOVA with Šídák’s multiple comparisons test. Source data

5FU has a multimodal mechanism of action: promoting DNA damage directly via its metabolite FdUTP or by inhibition of TS via FdUMP, depleting the pool of thymidine available for DNA synthesis, and causing RNA damage via FUTP (Fig. 3a)^4^. 5FU has previously been demonstrated to have a significant metabolic impact shortly after treatment, rapidly and consistently inhibiting TS^5^. We wondered whether inhibition of TS in cells surviving 5FU treatment promoted the observed reprogramming of mitochondria towards increased biogenesis and activity. We tested this idea using genetic and pharmacological inhibition of TS activity. Using small interfering RNA (siRNA) against *TYMS*, we show that knockdown of TS increases mitochondrial content in cells, indicated by NAO staining (Fig. 3b) and TOM20 expression (Fig. 3c; ca. twofold, *P* = 0.0008). We validated these data using the antifolate-specific TS inhibitor raltitrexed^33^. In line with our hypothesis, raltitrexed also significantly increased mitochondrial content (Fig. 3d), as did treatment with other TS-targeted antifolates pemetrexed and methotrexate in colon (Extended Data Fig. 5a) and lung cancer cell lines (Extended Data Fig. 5b). 5FU is often given in combination with DNA-damaging agents oxaliplatin (FOLFOX) or irinotecan (FOLFIRI)^3^. Importantly, 5FU-based combination treatment also shows an increase in mitochondrial mass (FOLFOX (5FU + 1 µM oxaliplatin) or FOLFIRI (5FU + 5 nM SN38, active metabolite of irinotecan)) that we attribute to 5FU-imposed stress, given that single-agent oxaliplatin or SN38 do not promote the same degree of mitochondrial adaptation (Extended Data Fig. 5c). Together, these data demonstrate that the observed changes in mitochondrial abundance caused by 5FU are mediated through its inhibitory action on TS.

We next explored the mechanism of biogenesis signalling. PGC1α is the master regulator of mitochondrial biogenesis^34^, and we see a small but sustained increase in PGC1α expression in response to 5FU over 72 h treatment (Fig. 3e; ~1.25-fold, *P* = 0.03, linear test for trend *P* = 0.0093, quantified from four independent repeats). The most common trigger for mitochondrial biogenesis via PGC1α is AMP-activated protein kinase (AMPK)^35^. AMPK is activated by energy stress; a drop in the AMP:ATP and ADP:ATP ratios indicates low energy availability and triggers AMPK activation. However, in our models, we observed no sustained increase in the AMP:ATP ratio (Fig. 3f) or in the phosphorylation status of AMPK or its direct downstream target, ACC (Fig. 3g). We also explored several other known regulators of PGC1α activity that are altered in response to 5FU treatment in HCT116, including NRF2, reactive oxygen species and PPARγ, but none of these prevented PGC1α-driven increases in mitochondrial mass. Furthermore, we assumed that this effect was independent of p53, given that the plethora of models with different p53 statuses all show similar phenotypes.

Given the reciprocal relationship between AMPK and mTOR signalling^36^ and the links between mTOR and PGC1α signalling^37^, we next explored the role of mTOR. Western blot profiling showed a modest but sustained increase in phosphorylation of S6 kinase and its target S6 with 5FU treatment over time (Fig. 3g; 72 h data: pS6K, ca. twofold, *P* = 0.11; pS6, ca. 2.5-fold, *P* = 0.0053). In addition, pretreatment with the mTORC1 inhibitor Torin1 significantly blunted the biogenesis driven by 5FU (Fig. 3h), demonstrating that it is, at least to some degree, under the control of the mTOR pathway. We further confirmed this effect using a second mTOR inhibitor, rapamycin (Extended Data Fig. 5d).

Given the robustness of this mitochondrial response, the multifaceted nature of PGC1α regulation in cells and a modest rescue at best by mTORC1 inhibition, we suspect that there is a degree of functional redundancy built into this metabolic plasticity system that can contribute to such an adaptive response, and complete inhibition of this system will prove difficult.

### Oxidative metabolism signatures predict response to 5FU-based chemotherapy treatment

**Fig. 4:**
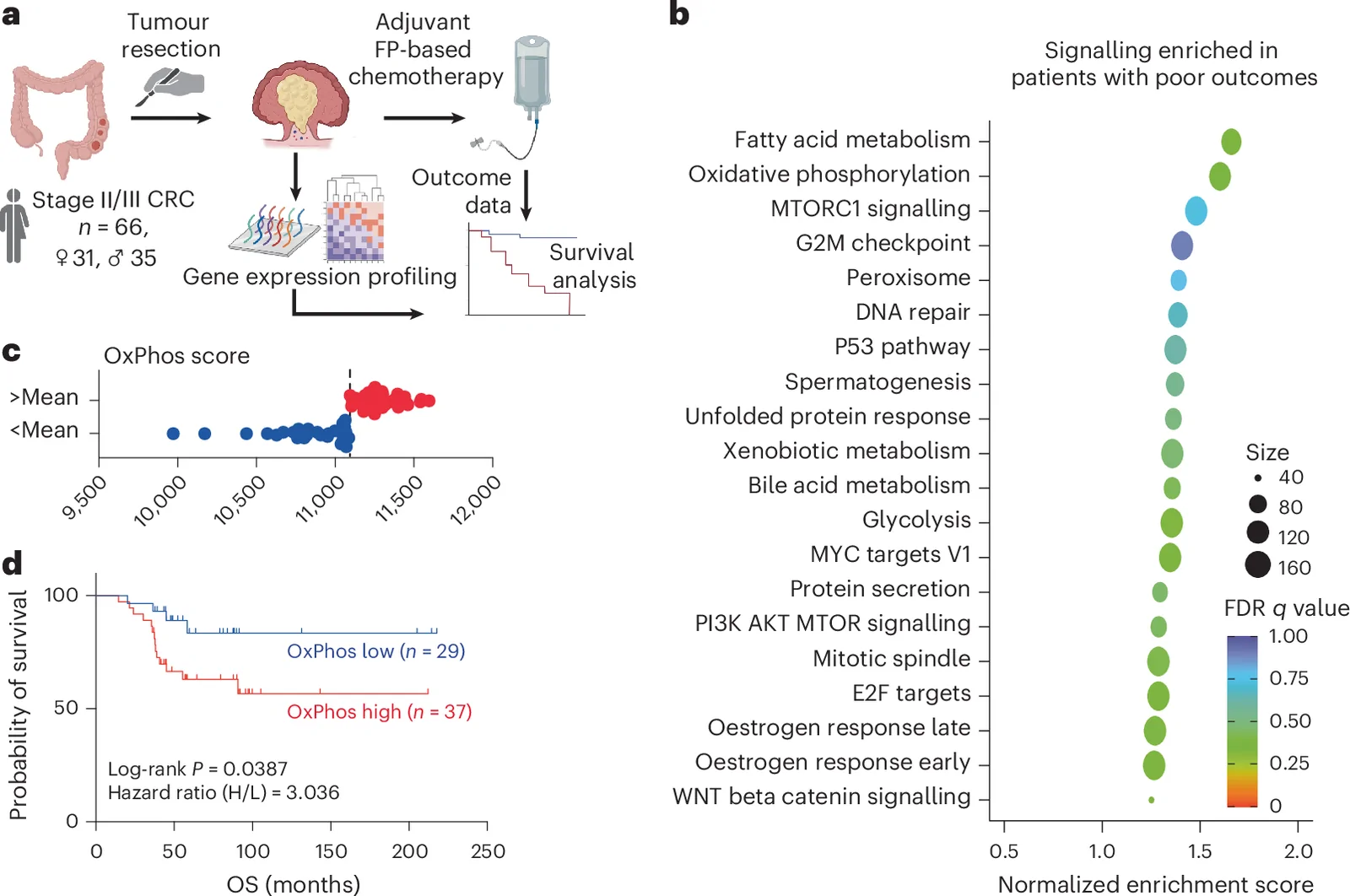
Mitochondrial metabolism predicts sensitivity to adjuvant 5FU-based chemotherapy. **a**, Schematic illustrating the Taxonomy cohort samples used for analysis. **b**, GSEA analysis of upper versus lower tertiles ranked on overall survival; graph shows normalized enrichment score for patients with poor outcomes. **c**, OxPhos scores per patient, split by cohort mean. **d**, Kaplan–Meier overall survival (OS) of patients split as in **c**, with log-rank test and hazard ratio analyses reported. Source data

**Extended Data Fig. 6:**
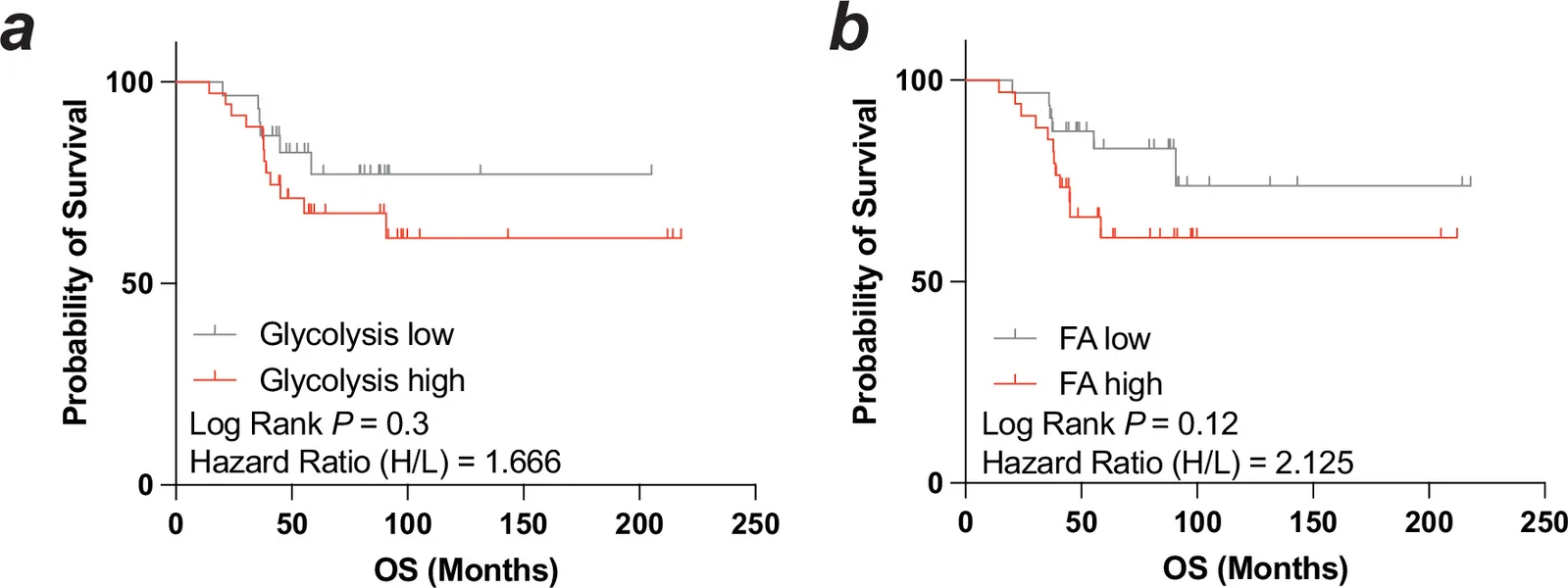
Glycolysis and Fatty Acid metabolism gene expression signatures and survival outcomes in adjuvant 5FU-based chemotherapy. Kaplan Meier Overall survival (OS) of patients split based on ssGSEA scores for Glycolysis (**a**) and Fatty Acid (FA) metabolism (**b**) signatures, with Log rank test and Hazard Ratio analyses reported. Source data

**Extended Data Fig. 7:**
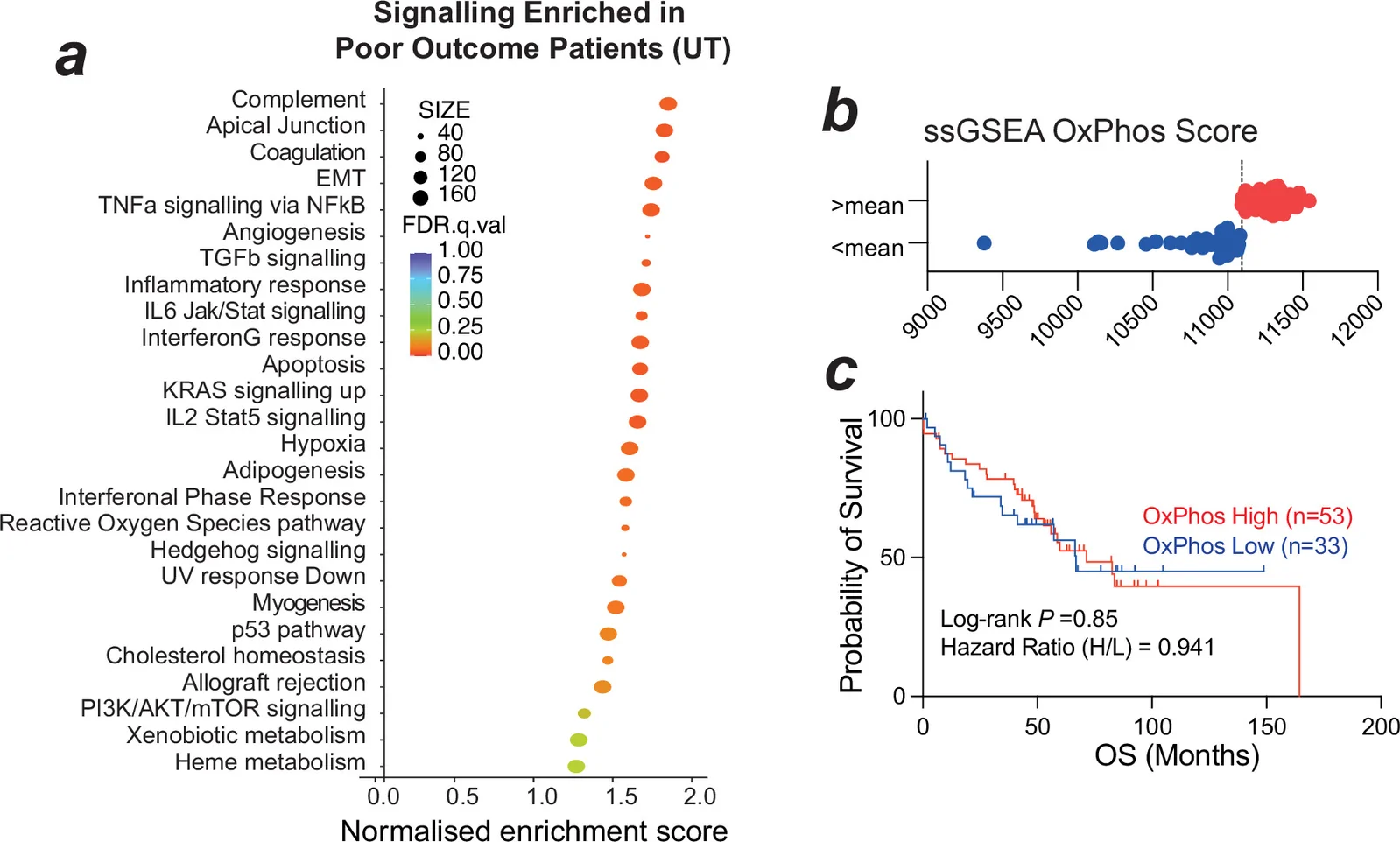
Mitochondrial metabolism does not predict poor outcomes following surgery alone. Analyses of patients included in Taxonomy cohort that did not receive adjuvant 5FU-based chemotherapy treatment; (**a**) GSEA analysis of upper vs lower tertiles ranked on overall survival, graph shows Normalised Enrichment Score (NES) for Poor Outcome patients; (**b**) OxPhos scores per patient, split by cohort mean; (**c**) Kaplan Meier Overall Survival (OS) of patients split as in (**b**), with Log rank test and hazard ratio analyses reported. Source data

Given this robust adaptation to facilitate cellular survival in cancer cells treated with 5FU, we next postulated that elevated mitochondrial metabolism may be a predictor of response to 5FU-based chemotherapy. We examined a cohort of patients with CRC who were treated with adjuvant fluoropyrimidine-based chemotherapy. This cohort, known as the Taxonomy cohort^38^, comprised 156 stage II or stage III patients, 66 (35 male, 31 female) of whom went on to receive adjuvant 5FU-based chemotherapy (illustrated in Fig. 4a). Tumour resection (of chemo-naive) samples were previously transcriptionally profiled, and data are available from the Gene Expression Omnibus (GEO) (GSE103479). Transcriptional data from these 66 treated patients were first analysed by gene set enrichment analysis (GSEA), comparing patients with good outcome (top 33% who had the best overall survival) with those with poor outcome (bottom 33% who had the poorest overall survival). Analyses showed that metabolic pathways ranked highly in patients with poor outcomes (Fig. 4b). Specifically, fatty acid metabolism (normalized enrichment score (NES) = 1.66), oxidative phosphorylation (OxPhos; NES = 1.60) and mTORC signalling (NES = 1.48) ranked as the top three pathways enriched in patients with poorer outcomes to 5FU-based treatment. We next assessed whether OxPhos signalling (as an indicator of mitochondrial metabolism) alone was sufficient to predict response to 5FU-based chemotherapy. Using single-sample GSEA, we assigned each patient an OxPhos score using the Molecular Signatures Database (MSigDB) Hallmarks Oxidative Phosphorylation signature^39,40^ and then split patients based on the cohort mean (Fig. 4c). Those above the mean were considered OxPhos-high, and those below as OxPhos-low. We compared survival outcomes between the two groups and observed a significant difference in overall survival (Fig. 4d; *P* = 0.0387, hazard ratio = 3.036). Interestingly, neither MSigDB Glycolysis nor Fatty Acid Metabolism Hallmark signatures held the same predictive power as OxPhos signalling (Extended Data Fig. 6). Importantly, when we compared OxPhos signalling in the same way to patients who did not receive 5FU-based chemotherapy treatment in the same cohort (*n* = 90), we observed no difference in survival (Extended Data Fig. 7), linking differences in patient survival directly to 5FU response. These data demonstrate that high OxPhos signalling is associated with 5FU resistance and suggest that enhanced mitochondrial metabolism promotes treatment failure clinically.

### Mitochondrial metabolism is directly linked to 5FU sensitivity

**Fig. 5:**
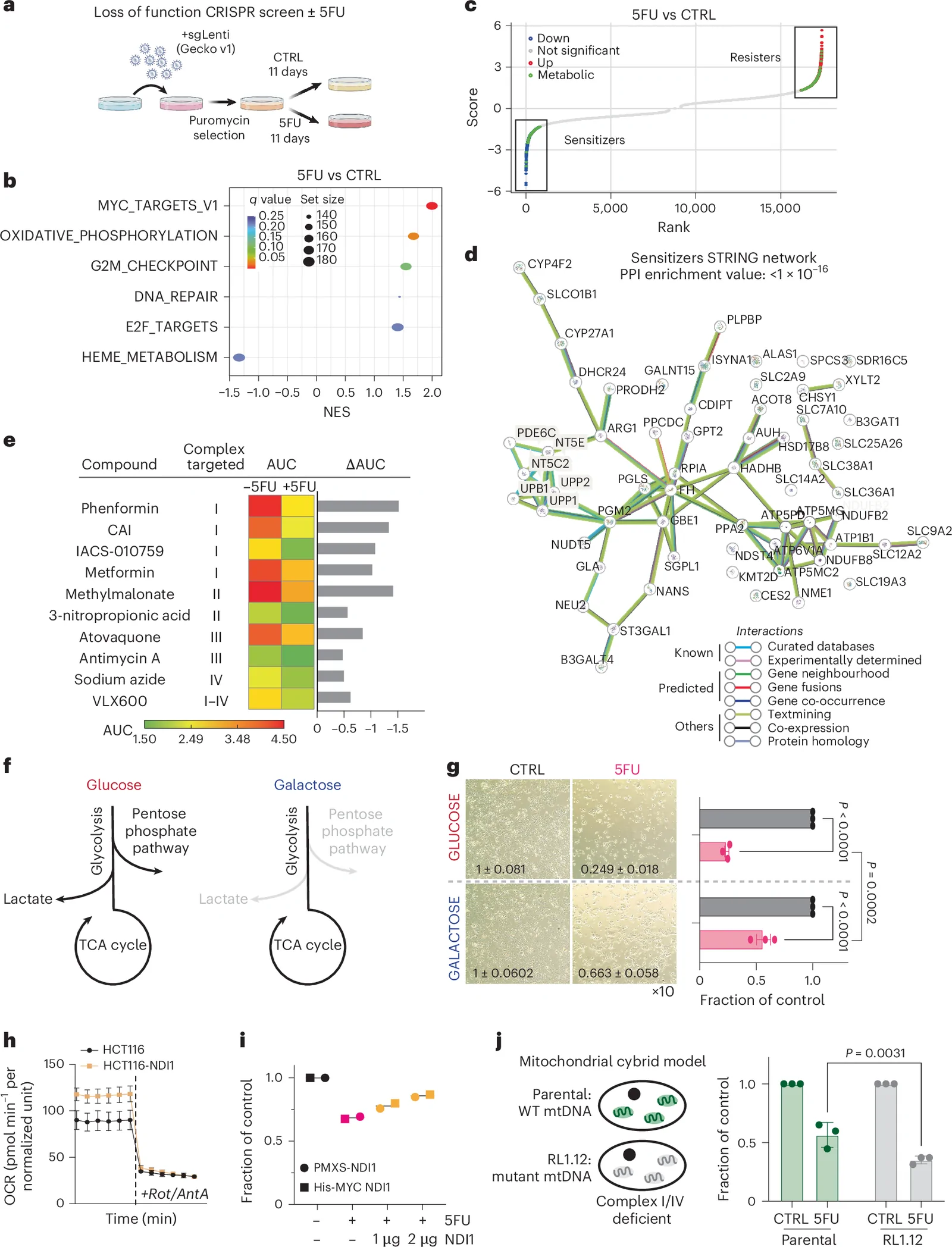
Mitochondrial metabolism dictates sensitivity to 5FU. **a**, Schematic representing experimental protocol for CRISPR screening with 5FU in HCT116 cells. **b**, GSEA analysis of MAGeCK RRA input. **c**, Sensitizers (blue) and resisters (red) identified by MAGeCKflute Cutoff Calling, with metabolic genes indicated in green. **d**, STRING network analysis of sensitizers with protein–protein interaction (PPI) enrichment indicated. **e**, AUC of mitochondrial ETC inhibitors in cells treated ±5FU at 72 h, with complex targeted detailed, and change in AUC (ΔAUC, +5FU – 5FU) shown. **f**, Schematic representing metabolic change imposed by culturing cells in galactose. **g**, Representative images and quantification (*n* = 3 biological replicates; mean ± s.e.m.) of cells cultured in glucose or galactose ±5FU treatment at 72 h, with SRB staining normalized to CTRL for each condition. **h**, OCR of HCT116 cells ±NDI1 overexpression for 72 h ±rotenone/antimycin A treatment. Data show technical replicates; mean ± s.d. **i**, HCT116 cells ±NDI1 overexpression ±5FU treatment for 72 h, with mean data for two independent NDI constructs shown, analysed by crystal violet. **j**, Parental (wild-type (WT)) and mtDNA mutant (RL1.12) 143B cells treated ±5FU for 72 h. Data show response as a fraction of CTRL; *n* = 3 (**g**, **j**) replicates; mean ± s.e.m. *P* values are indicated, calculated by one-way ANOVA with Dunnett’s multiple comparisons test (**i**), or two-way ANOVA with Šídák’s multiple comparisons test (**g**, **j**). Source data

**Extended Data Fig. 8:**
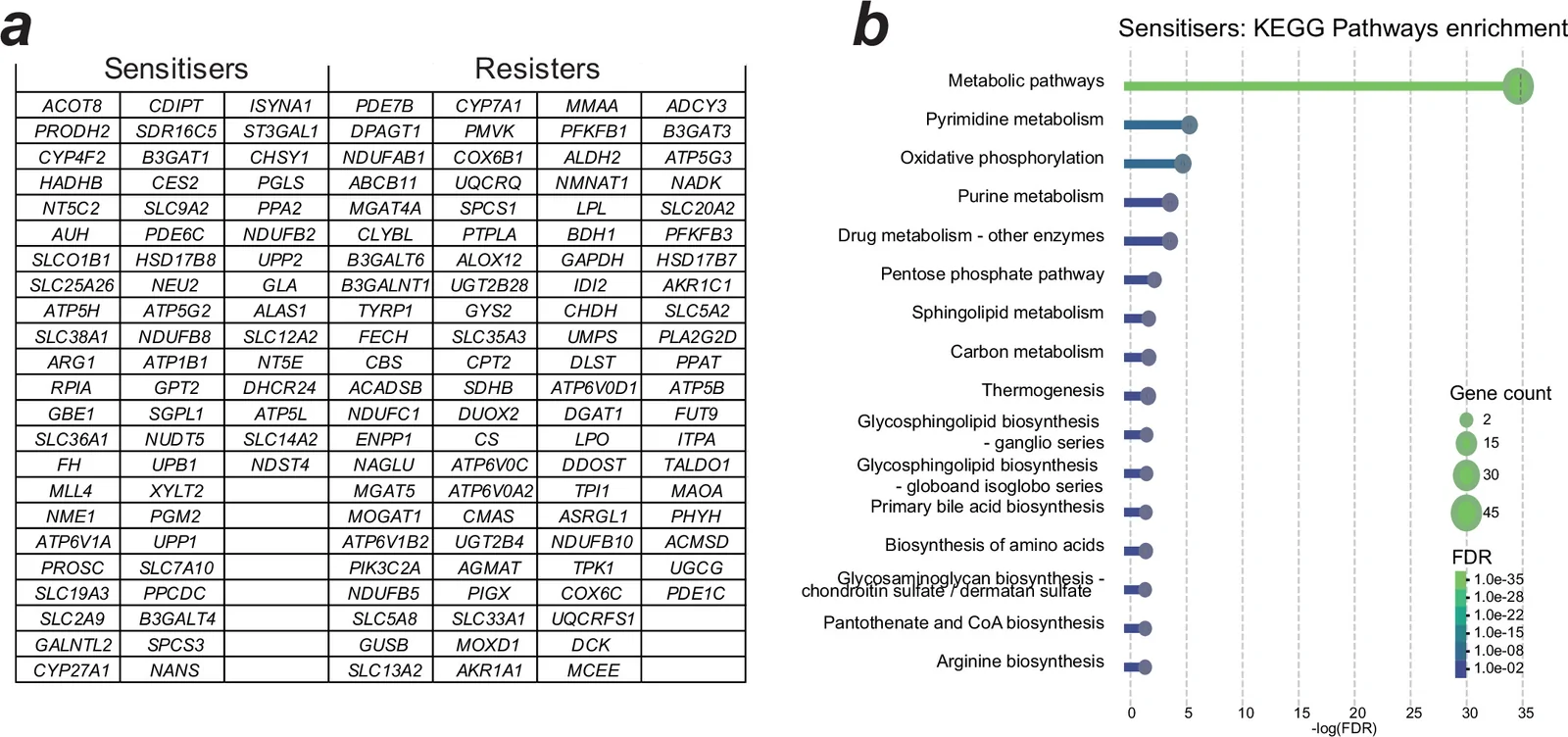
5FU sensitisers map to multiple metabolic pathways. (**a**) List of metabolic sensitisers and resisters from MAGeCKflute Cutoff Calling analysis of HCT116 ± 5FU CRISPR screen; (**b**) KEGG pathway enrichment analysis from STRING of sensitiser list.

**Extended Data Fig. 9:**
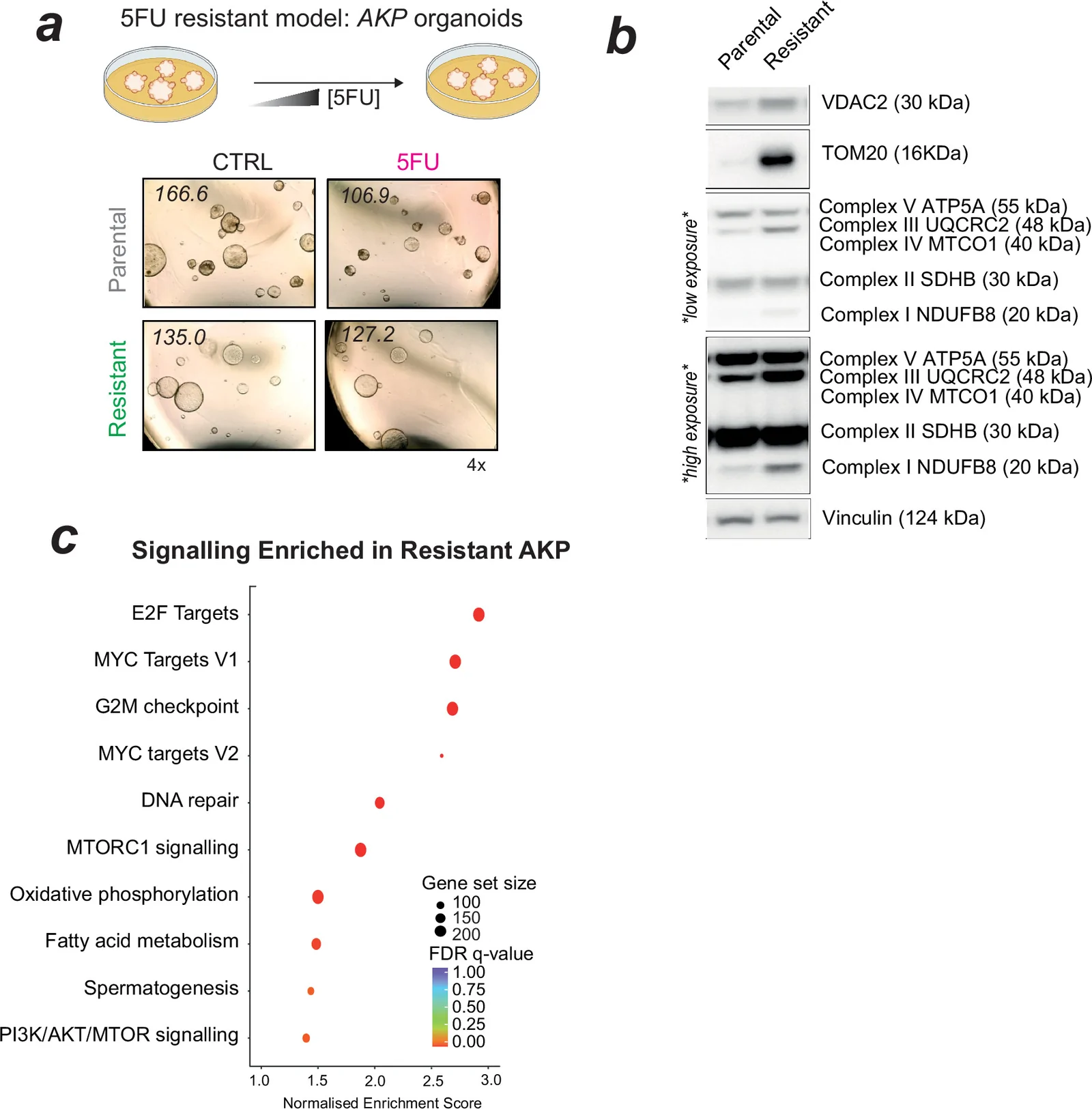
Acquired 5FU resistance is associated with increased mitochondrial content and signaling. (**a**) Schematic and representative images illustrating generation of 5FU resistant *AKP* organoids by continuous culture in increasing concentrations of drug, images at 4x magnification with mean organoid diameter denoted; (**b**) Mitochondrial protein expression in lysates from Parental and Resistant organoids, with low and high exposures noted. Representative blots of *n* = 3 shown; (**c**) GSEA analysis of RNA-seq data from Parental and Resistant *AKP*organoids. Source data

Clinically, patients with CRC usually undergo 6–12 cycles of 5FU-based or fluoropyrimidine-based chemotherapy^2,41^. However, after initial sensitivity, many patients develop resistance to treatment^42,43^. We postulated that 5FU treatment may promote an enhanced dependency on mitochondrial metabolism, and, in turn, mitochondrial metabolism may dictate sensitivity to 5FU. Using an HCT116 5FU–CRISPR screen, in which cells infected with the Gecko v1 library were cultured with or without 5FU for 11 days (Fig. 5a; 13–15 population doublings as per previous Gecko v1 screens), MAGeCK RRA overrepresentation analysis of genes enhanced in 5FU groups at endpoint showed that OxPhos genes were highly enriched (Fig. 5b). Using MAGeCKflute CutoffCalling, we compared the gene hits by category—sensitizers (those whereby a loss sensitizes to 5FU) and resisters (those whose loss promote insensitivity to 5FU)—and we observed several metabolic genes in both (Fig. 5c and Extended Data Fig. 8a). Given our focus on acute sensitivity, we mapped the network connections between sensitizer genes using STRING^44^. Here, we see significant clustering of genes involved in pyrimidine metabolism and OxPhos, including genes in CI and CV of the ETC (Fig. 5d and Extended Data Fig. 8b), identifying OxPhos as a key modulator of 5FU sensitivity. These data, when combined with mitochondrial mass and activity profiling in response to 5FU treatment presented above (Figs. 1 and 2), led us to reason that cells treated with 5FU become more dependent upon mitochondrial function for survival. To test this hypothesis, we treated HCT116 cells with or without 5FU with a range of ETC inhibitors, spanning CI–CIV (Fig. 5e). In each case, 5FU treatment reduced the IC_50_ of ETC inhibitors, demonstrating that 5FU treatment enhances dependency on ETC function. We then profiled mitochondrial phenotypes in chronically exposed 3D AKP organoid models that continue to grow on treatment (approximately four times more resistant to 5FU treatment; Extended Data Fig. 9a) and show that continued exposure to 5FU selects for increased mitochondrial mass (VDAC2, TOM20 expression; Extended Data Fig. 9b), ETC complex expression (CIII, CI; Extended Data Fig. 9b) and OxPhos signalling (Extended Data Fig. 9c). Together, these findings suggest that mitochondrial metabolism can dictate response to 5FU. Therefore, we next explored whether modulating mitochondrial metabolism was sufficient to alter 5FU sensitivity. First, we tested HCT116 cells cultured in DMEM media in which glucose (25 mM) was replaced with galactose (25 mM), a method previously described to promote enhanced TCA cycle flux (Fig. 5f)^45,46^. When compared with glucose-control cells, cells cultured in galactose were ~2.5 times less sensitive to 5FU (Fig. 5g), but the growth rate was slightly different (optical density of controls at 72 h GLC/GAL = 1.6), given that galactose culture makes cells divide more slowly^47^, meaning that further controls are needed to confirm the role of mitochondrial metabolism in modulating sensitivity. Therefore, we overexpressed NDI1 in HCT116 cells. NDI1 overexpression has been shown to rescue CI deficiency and limit the effects of mitochondrial dysfunction^48^. Here, we used transient overexpression of NDI1 (ref. ^49^) to increase NAD^+^ recycling and promote increases in mitochondrial-driven respiration, as shown by Seahorse XF analysis (Fig. 5h). Cells with increased mitochondrial activity were more resistant to 5FU treatment in the acute setting (72 h), as shown in a dose-dependent manner (Fig. 5i). Lastly, we tested whether cells that were functionally deficient in mitochondrial ETC metabolism, owing to mutations in CI and CIV, were differentially sensitive to 5FU treatment. Using the well-characterized 143B mitochondrial cybrid model^50^, we demonstrated that mtDNA mutant cells are more sensitive to 5FU treatment (Fig. 5j). Taken together, these data demonstrate that mitochondrial function is intrinsically linked to 5FU sensitivity, and that its inhibition may provide an opportunity to increase 5FU efficacy.

### Targeting ETC CI enhances 5FU response and extends survival

**Fig. 6:**
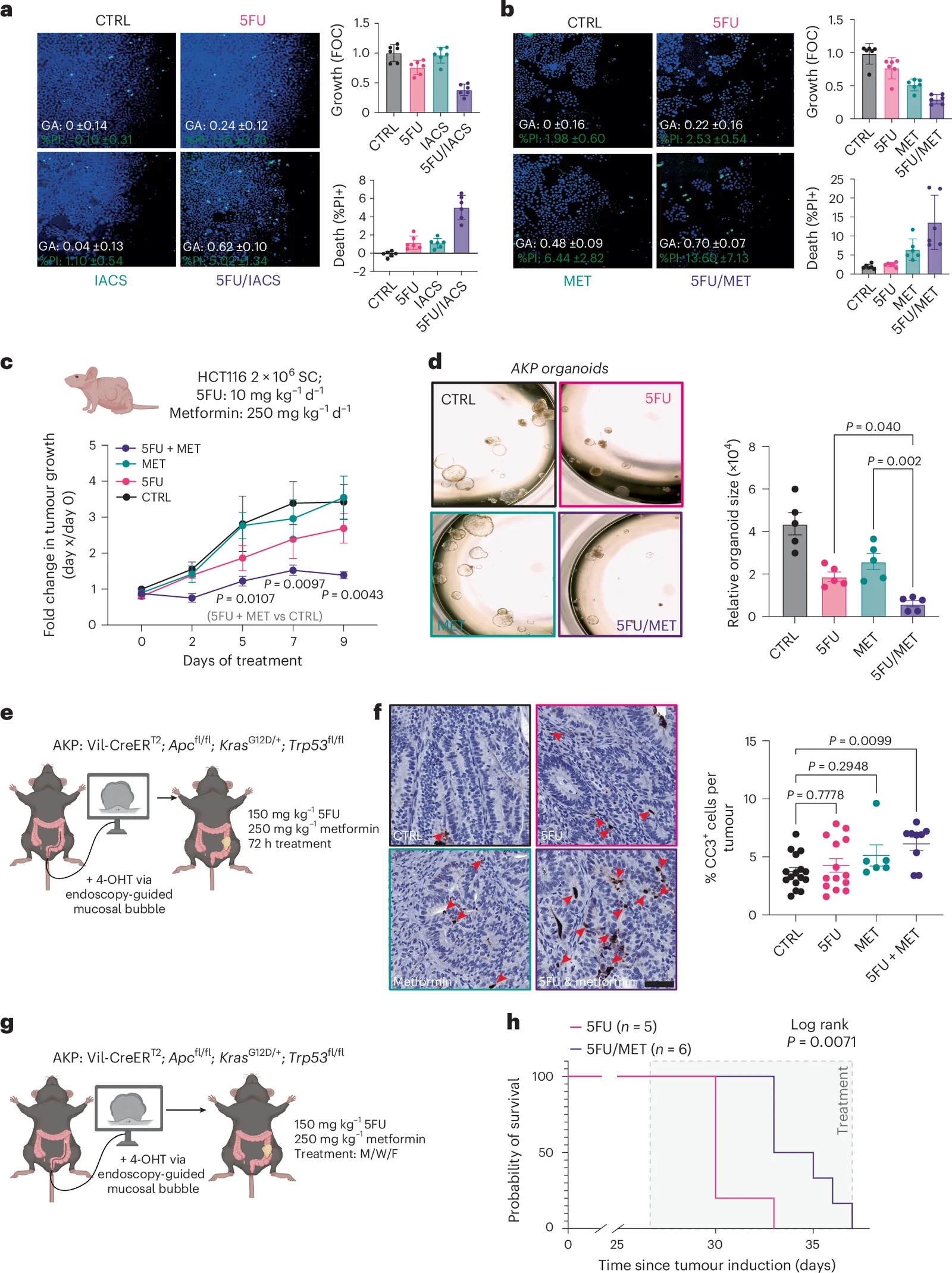
Inhibiting mitochondrial metabolism increases 5FU efficacy. **a**,**b**, High-content images (magnification, ×10) from HCT116 cells stained with Hoechst 33342 (blue) and propidium iodide (PI; green) following treatment ±5FU and ±CI inhibitors IACS-010759 (IACS; **a**) or metformin hydrochloride (MET; **b**) for 72 h. Change in growth (number of Hoechst-positive cells relative to vehicle control and media-only control) and death (% PI-positive cells relative to vehicle control) are shown; data points show technical replicates ± s.d., with mean growth arrest (1 − growth FOC (fraction of control)) and mean % PI-positive ± s.d. denoted. **c**, HCT116 subcutaneous xenograft growth over time in mice treated with PBS (CTRL), 5FU, metformin or 5FU + metformin combination, with tumour growth normalized to day 0. Data points show mean tumour volume per group ± s.e.m. at each timepoint (*n* = 4 or 5 animals per group). **d**, AKP organoids treated as indicated with 5 μM 5FU and/or 3 mM metformin for 72 h. Representative images (magnification, ×4) and quantification of relative organoid size are shown. Data points show mean of independent organoid cultures ± s.e.m. **e**, Schematic representing experimental design for tumour induction and 5FU/metformin treatment in AKP mice. **f**, Representative images and quantification of CC3 staining (% positive/total cells) by immunohistochemistry in colon tumours (**f**, from **e**; CTRL, *n* = 16; 5FU, *n* = 14; metformin, *n* = 6; 5FU + metformin, *n* = 9 tumour regions). Positive cells and clusters indicated by red arrows. Scale bar, 50 µm. Data points show mean % CC3^+^ cells per tumour ± s.e.m. **g**, Schematic of experimental design of 5FU or 5FU + metformin treatment in AKP mice for survival analysis. **h**, survival outcomes by Kaplan–Meier plot, analysed using log-rank test, with animal number per cohort and *P* values reported. *P* values were determined by one-way ANOVA with Tukey’s (**a,b**), Šídák’s (**d**) or Dunnett’s multiple comparisons tests against CTRL samples (**f**), or two-way ANOVA with Šídák’s multiple comparisons test (**c**). Source data

The ultimate goal of better understanding mechanisms promoting 5FU tolerance is to allow us to design rational treatment combinations that are more effective than the current standard of care. 5FU treatment increases mitochondrial mass and activity (Figs. 1 and 2) and promotes an imposed dependency on mitochondrial function (Fig. 5). Importantly, we see that the increase in mitochondrial mass is cancer-cell-specific (Extended Data Fig. 3), suggesting that there is a therapeutic window for co-treatment. We confirm that co-treatment with 5FU and mitochondrial inhibitors, particularly CI inhibitors IACS-010759 (a specific CI inhibitor under clinical evaluation^51^) and metformin hydrochloride (a treatment for diabetes that inhibits CI^52^), increases growth arrest and death in CRC cells (Fig. 6a,b). Other groups have reported increased efficacy with 5FU and metformin in models of acquired resistance^53–56^, in spheroid culture^53^ or xenograft/PDX models^57–59^, but the potential of combined 5FU–CI inhibitor co-treatment in a chemo-naive, immune-competent setting requires further investigation.

**Extended Data Fig. 10:**
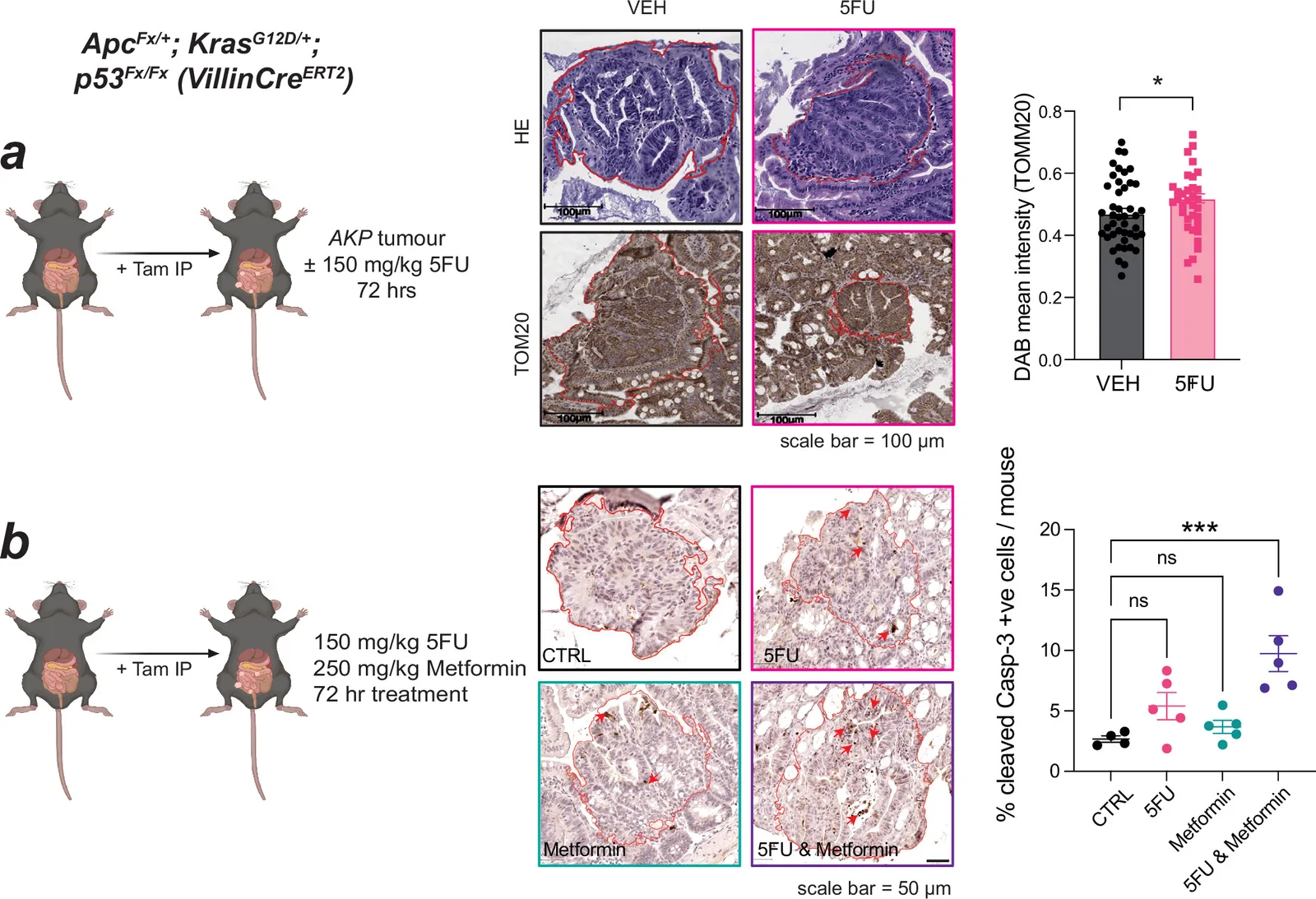
Inhibiting mitochondrial metabolism increases 5FU efficacy. Schematic representing experimental design for tumour induction and treatment in *VillinCre*^*ERT2*^*; Apc*^*Fx/+*^*; Kras*^*G12D/+*^*; Trp53*^*Fx/Fx*^ mice (**a-b**). Tumours stained and quantified for TOM20 intensity (**a**) or cleaved Caspase 3 positivity (**b**); P values indicated, determined by two-tailed *t*-test (**a**) or one-way ANOVA with Dunnett’s multiple comparisons test against CTRL (**b**). Source data

Given the recent clinical trial data that suggests neurotoxicity issues with IACS^51^, we moved forward with 5FU and metformin for in vivo evaluation. We first tested efficacy in HCT116 xenograft models. Subcutaneous tumours grown in athymic nude mice were randomized when the average tumour volume reached 100 mm^3^ and were allocated to one of four treatment groups: saline control; 5FU (10 mg kg^−1^ d^−1^); metformin (250 mg kg^−1^ d^−1^); and 5FU + metformin. Animals were treated daily for 1 week, and change in tumour volume was normalized to day 0. 5FU resulted in a modest, non-significant decrease in tumour growth compared to control, and metformin alone had no significant impact on tumour growth. However, the combination of 5FU with metformin treatment showed significant growth inhibition from day 5 of treatment (Fig. 6c). Given the enhanced efficacy of the 5FU + metformin combination in AKP organoid models in 3D (Fig. 6d), we moved forward to evaluate the combination treatment in the more complex orthotopic GEMM models of CRC. Colon-tumour-bearing AKP mice (Fig. 1) were randomized and treated with 150 mg kg^−1^ 5FU, 250 mg kg^−1^ metformin, a combination of both and/or respective saline vehicles, and tissues were collected 72 h post injection (Fig. 6e). Formalin-fixed, paraffin-embedded sections were stained for cleaved caspase-3 (CC3), a marker of apoptotic cell death, and the percentage of positive cells per tumour was quantified using QuPath^27^. Multiple colon tumour areas were assessed per animal, and the average percentage of CC3^+^ cells per tumour is shown. Only in the combination group was there significant induction of cell death compared to control groups (Fig. 6f). We also tested therapeutic response in less advanced colon lesions, using Vil-Cre; *Apc*^fl/+^; *Kras*^G12D/+^; *Trp53*^fl/fl^ mice injected i.p. with 80 mg kg^−1^ tamoxifen. Low-grade tumours developed over 3–6 weeks in the small and large intestines. At onset of tumour symptoms, animals were randomized and treated with 5FU to assess changes in mitochondrial mass by immunohistochemistry staining of TOM20 (Extended Data Fig. 10). Multiple tumour areas in the large intestine were quantified and demonstrate a similar increase in mitochondrial content when treated with 5FU (Extended Data Fig. 10a). To assess the efficacy of combination treatment, tumour-bearing mice were treated as above with 5FU + metformin and CC3-quantified, showing an increase in average cell death per tumour per mouse with combination treatment (Extended Data Fig. 10b). Lastly, we tested whether the 5FU + metformin combination treatment could extend survival when compared to 5FU alone in mice with advanced AKP tumours. Tumours were induced, and upon presentation of clinical symptoms of tumour burden, animals were randomized and treated with 150 mg kg^−1^ 5FU three times per week, with 250 mg kg^−1^ metformin or vehicle control delivered concurrently (Fig. 6g). Animals began treatment ~26 days post induction, and survival was recorded as days post-tumour induction at which the clinical endpoint was reached. Kaplan–Meier survival analysis demonstrates that 5FU with metformin treatment resulted in a significant extension in survival (Fig. 6h; log-rank test, *P* = 0.0071) and a hazard ratio of 5FU with metformin to 5FU alone of 0.3330.

Taken together, these in vivo data from multiple CRC models support our conclusion that inhibition of mitochondrial metabolism by metformin treatment can enhance the response of 5FU treatment by increasing tumour cell death and impeding tumour growth. Importantly, we show that combination treatment is efficacious in chemo-naive tumours of varying grade and has the potential to increase survival even in an advanced tumour setting.

### Mitochondrial biogenesis is only advantageous if mitochondria are functional

**Fig. 7:**
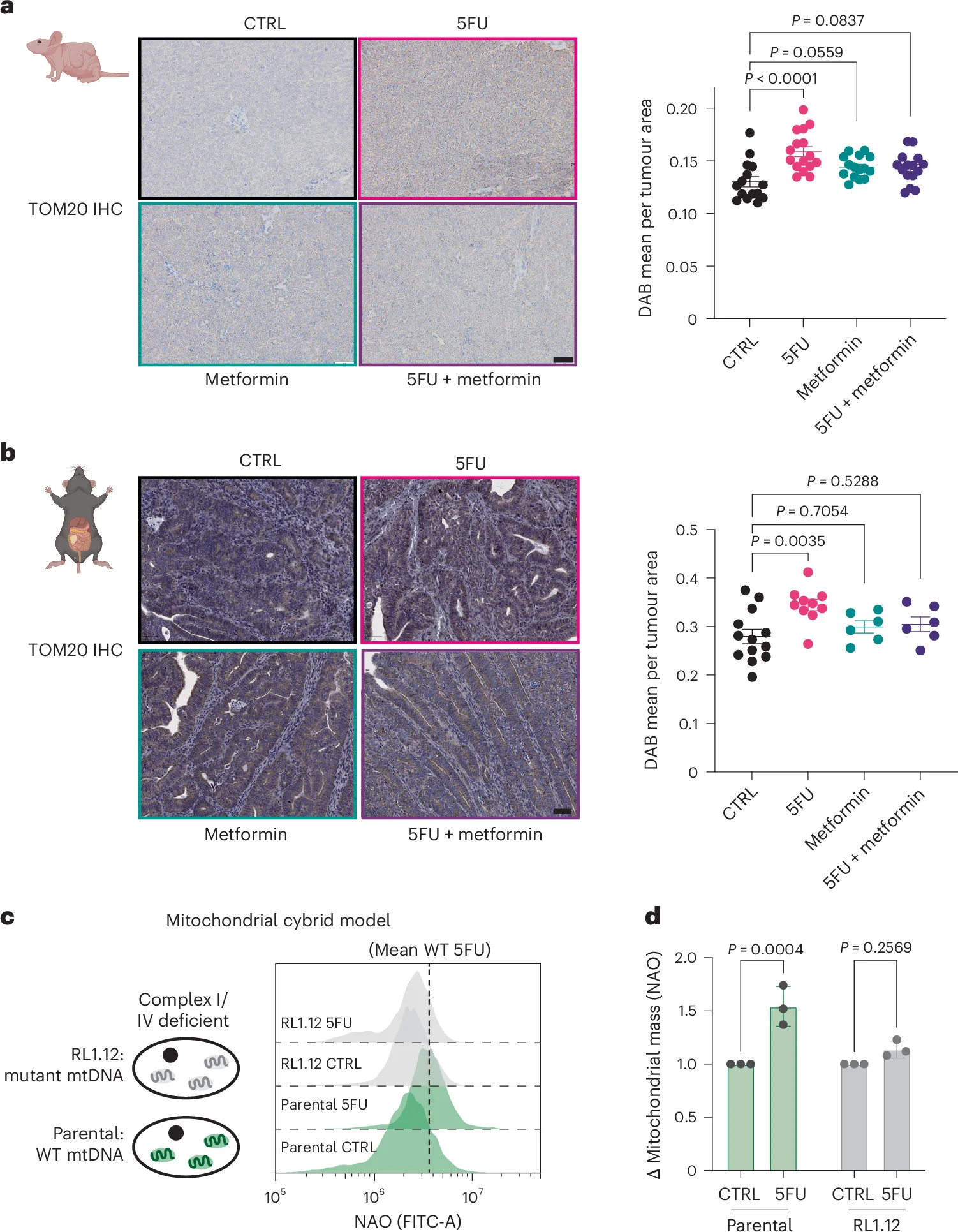
Inhibiting mitochondrial metabolism blunts 5FU-driven mitochondrial biogenesis. **a**,**b**, Relative mean intensity of TOM20 staining of HCT116 xenografts (**a**, from Fig. 6c) or AKP tumours (**b**, from Fig. 6e) per tumour area measured. Scale bar, 50 μm. **c**,**d**, Mitochondrial mass by NAO staining in WT mtDNA (parental) and mutant mtDNA (RL1.12) cybrid cells ±5FU, with representative histograms (**c**) and quantification of *n* = 3 biological replicates (**d**). Data show mean ± s.e.m; scale bar, 50 μm. *P* values were determined by one-way ANOVA with Dunnett’s multiple comparisons test against CTRL (**a**, **b**) or two-way ANOVA with Šídák’s multiple comparisons test (**d**). Source data

We examined mitochondrial staining by TOM20 immunohistochemistry in HCT116 xenografts and AKP orthotopic tumours treated with 5FU and/or metformin. 5FU treatment significantly increases TOM20 staining as shown in Fig. 1. Interestingly, metformin treatment blunted this increase in mitochondria (Fig. 7a,b). We reasoned that the ability of metformin to block activity rendered the increase in mitochondrial content non-advantageous. Therefore, to functionally test whether mitochondrial biogenesis is linked to mitochondrial function, we explored 5FU-driven changes in mitochondrial abundance in the 143B cybrid model (Fig. 7c), in which mtDNA mutant cells show increased sensitivity to 5FU (Fig. 5j). In parental (wild-type mtDNA) cells, 5FU drives significant increases in mitochondrial mass; however, this effect is significantly blunted in cells deficient for CI and CIV (Fig. 7d), confirming that the need to increase mitochondrial mass in 5FU-treated cells is driven by a dependence on increased mitochondrial activity, and metformin can block this mitochondrial adaptation to prevent drug tolerance in tumours.

### Mitochondrial biogenesis facilitates resolution of pyrimidine stress

**Fig. 8:**
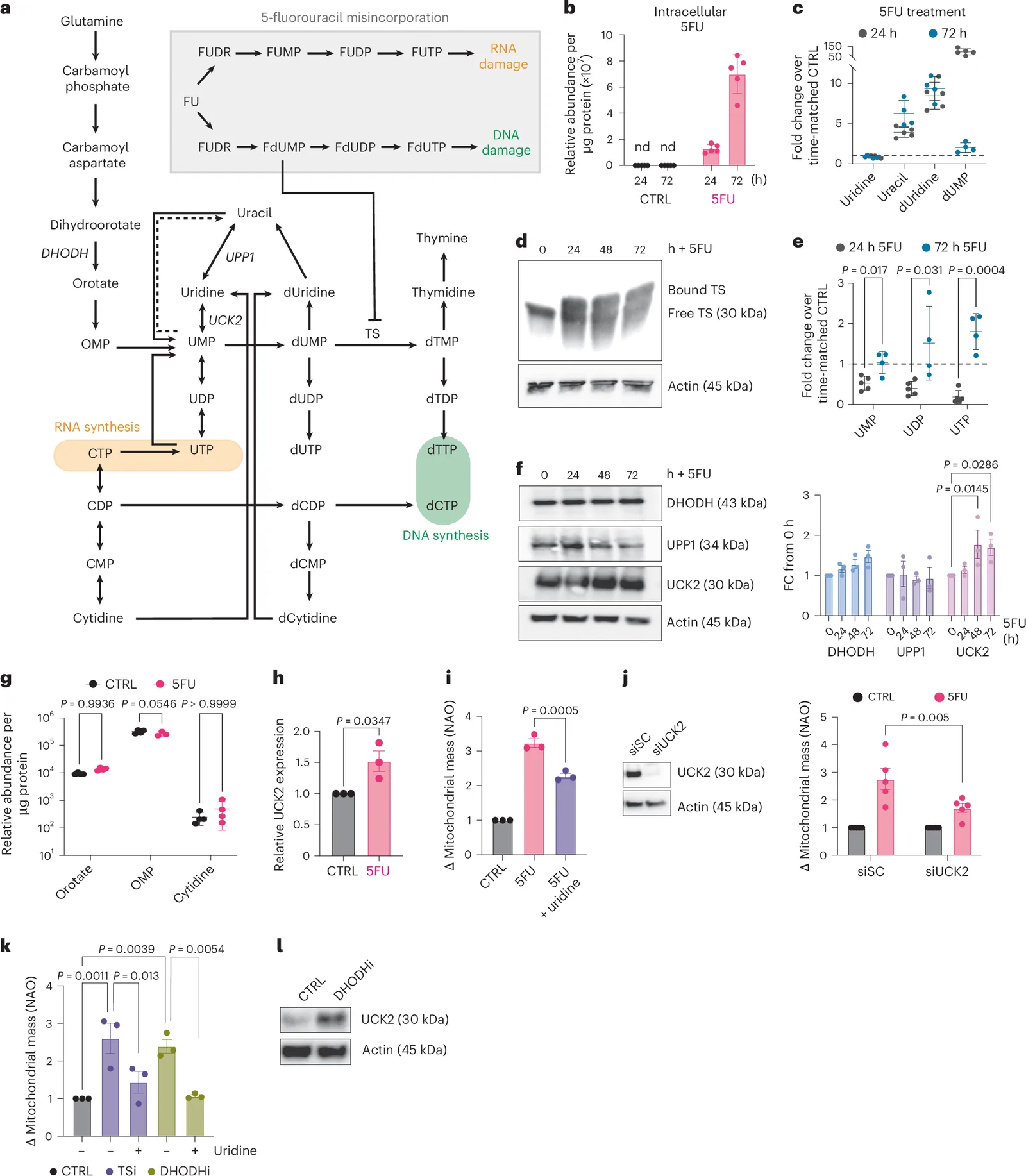
Metabolic adaptation rebalances 5FU-driven nucleotide stress. **a**, Schematic representing nucleotide biosynthesis and impact of 5FU. **b**, Relative intracellular abundance of 5FU by LC–MS quantification of HCT116 cells treated ±5FU; nd, not detected. Values are mean ± s.d. of technical replicates. **c**, Fold change in intracellular metabolite abundance over time-matched control at 24 h (black) and 72 h (blue) post 5FU treatment. Data show technical replicates, with mean ± s.d. denoted. **d**, TS expression and band shift in protein lysates from HCT116 cells treated ±5FU over time; representative blot of *n* = 4 experiments. **e**, Fold change in intracellular nucleotide abundance over time-matched control at 24 h (black) and 72 h (blue) post 5FU treatment. Data show technical replicates, with mean ± s.d. denoted. **f**, Protein expression in lysates from HCT116 cells treated ±5FU over time, as previously analysed in Fig. 3g. Representative actin blot is shown; densitometry from *n* = 3 experiments is presented as mean ± s.e.m. **g**, Relative intracellular metabolite abundance ±5FU for 72 h. Data show technical replicates; mean ± s.d. **h**, UCK2 expression from *n* = 3 independent experiments ±5FU at 72 h; mean ± s.e.m. **i**, Mitochondrial mass by NAO staining in HCT116 cells treated ±5FU ±uridine for 72 h, *n* = 3 independent experiments; mean ± s.e.m. **j**, representative UCK2 knockdown in HCT116 cells at 96 h post transfection, and mitochondrial mass by NAO staining in siSC and siUCK2 cells ±5FU treatment; *n* = 5 independent experiments; mean ± s.e.m. **k**, Mitochondrial mass by NAO in cells treated with raltitrexed (TSi) or BAY-2402234 (DHODHi), with or without uridine, for 72 h; *n* = 3 independent experiments; mean ± s.e.m. **l**, Representative UCK2 expression in cells treated ±BAY-2402234 for 72 h, representative of *n* = 6 experiments. *P* values were determined by multiple *t*-test (**e**, **g**), one-way ANOVA with Dunnett’s multiple comparisons test (**f**, **h**), two-tailed *t*-test (**i**) or two-way (**j**) or one-way (**k**) ANOVA with Šídák’s multiple comparisons test. Source data

Mitochondrial adaptations promote survival in 5FU-tolerant cells. Our data thus far show that 5FU drives increased mitochondrial biogenesis to address a functional requirement for increased mitochondrial ETC metabolism. However, the data do not explain what process this increased mitochondrial activity supports. To explore the enhanced dependency on mitochondrial function, we further explored the acute metabolic stress imposed by 5FU treatment. As summarized in Fig. 8a, 5FU inhibits TS and can cause an imbalance in uridine-derived and deoxyuridine-derived metabolites. We confirmed that 5FU treatment over 72 h continues to promote accumulation of 5FU within cells (Fig. 8b), suggesting that the cells do not buffer the impact of TS inhibition by increasing drug efflux over time. Furthermore, we continue to see increased deoxyuridine (fold increase at 24 h, 8.5×; 72 h, 9.3×) and uracil (fold increase at 24 h, 3.9×; 72 h, 6.2×) levels following 5FU treatment (Fig. 8c), indicative of detoxification of dUMP accumulation, a marker of TS inhibition^4,60,61^. We also see sustained TS inhibition over 72 h, as indicated by elevated dUMP levels (Fig. 8c; 24 h, 100×; 72 h, 2×) and a TS band shift on western blot (Fig. 8d), confirming continued formation of the ternary complex^62^. Interestingly, the levels of dUMP accumulation reduced over the 72 h despite continued TS inhibition, suggesting that there is rebalancing of the nucleotide pools to resolve stress. In response to 5FU treatment, we see little to no impact on other nucleotide families (apart from ATP as previously shown; Fig. 2d), but dramatic depletion of UMP, UDP and UTP after 24 h of 5FU treatment (Fig. 8e), demonstrating the specificity of nucleotide stress imposed by 5FU. Interestingly, this stress is resolved at 72 h 5FU (Fig. 8e), despite the accumulation of intracellular 5FU and continued TS inhibition. We first reasoned that changes in DHODH activity were responsible for buffering this imbalance. DHODH is significantly downregulated at the mRNA level in response to 5FU treatment (RNA sequencing (RNA-seq) data; Extended Data Fig. 4), but this is not reflected at the protein level (Fig. 8f); there is no change in orotate or OMP (Fig. 8g) in response to 5FU to offset UMP depletion, and there is no difference in cytidine levels. We do, however, see changes in UCK2 expression (Fig. 8f), which suggests that nucleotide salvage might instead be linked to our observed mitochondrial phenotypes.

The acute decrease in UMP/UDP/UTP levels that resolves despite continued TS inhibition supports the hypothesis that an increase in nucleotide salvage might be linked to our observed mitochondrial phenotypes. Without uridine, the cell compensates for pyrimidine shortage by over-activating ATP-consuming salvage pathways (UCK2, TK1, dCK), which potentially drives mitochondrial biogenesis to generate more ATP for these phosphorylation reactions^63^. Indeed, we see increased UCK2 expression 72 h post 5FU treatment (Fig. 8h). Increasing uridine availability would remove the stress on this system by resolving the UMP deficiency; therefore, we tested this hypothesis by exogenous supplementation of uridine. We see a significant reduction in 5FU-driven mitochondrial biogenesis when cells are supplemented with uridine (Fig. 8i). Uridine alleviating the need to increase mitochondrial biogenesis led us to conclude that increased pyrimidine salvage (probably via UCK2) is driving this mitochondrial adaptation. UCK2 (which converts uridine to UMP) in particular requires ATP as a substrate^63^ and has been linked with mitochondrial function^64^ and mTOR signalling^65^. We observe increased UCK2 expression 72 h post 5FU treatment (Fig. 8h), which increases over time (Fig. 8f), and this correlates with our reported increase in mitochondrial mass, ATP production and mTOR activity (Figs. 2 and 3). We also see an increase in uridine transporters SLC29A4 and SLC29A3 at the transcriptional level (RNA-seq analysis previously discussed; Extended Data Fig. 4). These data suggest that we might indeed have hyper-activity of pyrimidine salvage. Uridine supplementation results in decreased UCK2 activity over time owing to cellular feedback. To confirm whether an increase in UCK2 activity as a result of pyrimidine stress was the driving force behind mitochondrial biogenesis signalling, we knocked down UCK2 in cells using siRNA (Fig. 8j). This knockdown significantly reduced the increase in mitochondrial mass seen with 5FU treatment. We then tested whether other agents that specifically caused nucleotide imbalance (rather than the DNA-driven and RNA-driven effects of 5FU) have the same mechanism of action. Indeed, the increase in mitochondrial mass owing to specific TS inhibition using raltitrexed (Fig. 3d) is rescued by supplementation with uridine (Fig. 8k, blue bars). We postulated that if depletion of the UMP/UDP/UTP pool is driving this stress, then we would see similar effects with a DHODH inhibitor, BAY-2402234 (ref. ^66^). DHODH inhibition also promotes significant increases in mitochondrial mass with 72 h treatment that is rescued with uridine supplementation (Fig. 8k, yellow bars), and a strong increase in UCK2 expression (Fig. 8l; ~2.95, *P* = 0.0158). Taken together, these data confirm that the activity of UCK2 to restore UMP levels is the driving force behind increased PGC1α–mTOR-linked mitochondrial biogenesis to resolve pyrimidine stress when cells are treated with 5FU.

## Discussion

Drug resistance is not a new issue, but remains the greatest difficulty for effective treatment of patients with cancer worldwide. As previously described^1^, the combination of drugs targeting distinct, non-overlapping pathways has proven effective in enhancing treatment efficacy. In CRC, combining 5FU with DNA-damaging agents oxaliplatin and/or irinotecan was an empirical choice rather than a rationally designed strategy, and no new combination partners have been added since the introduction of the biological EGFR and VEGF-targeting agents in the early 2000s. Given that fluoropyrimidine-based therapy will remain a major treatment modality for patients with advanced CRC for the foreseeable future, the need for better, more effective chemotherapy combinations in biologically selected populations to overcome drug resistance is an urgent, unmet need for patients with CRC worldwide.

Our findings describe a role for mitochondrial plasticity as a mechanism for acute 5FU tolerance in CRC cells: mitochondrial changes acutely adopted by CRC cells in response to 5FU-based standard-of-care treatment result in increased mitochondrial biogenesis, which provides metabolic support for increased pyrimidine scavenging by UCK2 to resolve nucleotide stress. This adaptation presents a targetable vulnerability whereby the functional inhibition of this adaptive response greatly increased the effectiveness of 5FU across CRC models in vitro and in vivo.

Our findings demonstrate that increased mitochondrial function, acquired through mitochondrial biogenesis, is required to promote survival in response to acute 5FU treatment. We show that this mitochondrial plasticity is driven by a need to resolve nucleotide stress via ATP-consuming UCK2. We see an increase in CI activity and ATP production to support increased mitochondrial activity; the ^13^C_6_-glucose tracing experiments show increased TCA activity; and our targeted LC–MS data demonstrate a profound change in broad metabolic activity in cells, supported by RNA-seq analysis of metabolic gene expression changes. Although we cannot rule out a contribution of glycolysis to this ATP increase and did not directly assess the activity of other complexes, we observed increased 5FU sensitivity with inhibitors of ETC complexes I–IV, genetic ablation of CI and CIV, and sensitizer genes from CRISPR screening covering additional complexes (CIII and CV). Therefore, we conclude that mitochondrial function is required to support this acute adaptive response to 5FU.

Mitochondrial adaptations to promote drug tolerance are becoming more widely reported. Upregulation of oxidative mitochondrial metabolism is an accepted method of resistance to other therapeutic interventions in various diseases, such as pancreatic cancer and acute myeloid leukaemia^67,68^. Moreover, since the inception of this study, multiple metabolic mechanisms promoting drug resistance have been suggested for 5FU treatment, including changes in amino acid metabolism^69,70^, serine and glycine metabolism^71–73^ and glycolysis^74,75^, corroborated within our study and independently validating our findings. Furthermore, incomplete MOMP driven by mitochondrial adaptation and sublethal cytochrome *c* release has also been linked to the generation of BH3-mimetic drug-tolerant persister cells^76^. Interestingly, we see marginal release of cytochrome *c* following 5FU and a transient dip in MOMP, but no enhanced difference in sensitivity to BH3-mimetics or increase in ATF4 expression or predicted activity in RNA-seq analysis (data not shown); therefore, we cannot yet conclude whether incomplete MOMP contributes to the modulation of 5FU drug-tolerant persister cells by regulating mitochondrial biogenesis or through independent mechanisms, and we will be explored this possibility in subsequent studies.

We also demonstrate that mitochondrial metabolism may have a role in modulating both intrinsic and acquired 5FU resistance. Increasing mitochondrial metabolism makes tumour cells intrinsically more resistant to 5FU. We further demonstrate that oxidative mitochondrial signalling can be used as a biomarker of response to fluoropyrimidine-based chemotherapy treatment in patients with CRC and may have clinical utility for stratifying patients at higher risk who could benefit from metabolically targeted combination therapies. Furthermore, the acute metabolic response to 5FU treatment is profound and facilitates tolerance. Although the majority of readouts are at bulk level, mitochondrial changes in mass and morphology at single-cell resolution suggest an adaptive response rather than a selection for pre-existing tolerant cells in clonal lines. Therefore, we suggest that the response observed is a dynamic adaptation to 5FU-imposed pyrimidine stress. However, as detailed above, cells that exhibit higher levels of mitochondrial activity are less sensitive to 5FU treatment, and those with compromised mitochondrial function are more sensitive, correlating with intrinsic resistance. The widespread transcriptional metabolic reprogramming observed in response to acute 5FU treatment is contrary to the transcriptional patterns associated with long-term growth arrest. The timepoint chosen (when drug-tolerant cells are emerging after treatment) probably contributes to this effect, and later timepoints (when cells perhaps enter senescence or other more terminally arrested states) are more likely to reflect the expected gene expression patterns. However, chronic exposure can promote stable increases in these metabolic phenotypes, as evidenced by our resistant organoid models (Extended Data Fig. 9). In cell line models, which display limited heterogeneity, it is less likely that clonal populations would be evident at short timepoints; however, in vivo, one could imagine that repeated 5FU exposures would drive selection of pre-existing niches of tumours cells that had higher mitochondrial functionality. Therefore, we predict that both mechanisms will have a role in modulating tumour 5FU sensitivity.

Inhibition of oxidative metabolism to target cancer cell proliferation using various inhibitors has shown promise in both human and murine preclinical models^77^. Furthermore, in acquired models of 5FU resistance that show mitochondrial dysfunction, combination of ETC CI inhibition (by metformin and others) and 5FU treatment increased tumour control. However, the mechanisms driving this increased efficacy remained unclear. Indeed, recently there have been several studies reporting an impact of acquired 5FU resistance on mitochondrial biology; some report distinct dysfunction in 5FU-resistant models, and some, like our organoid model presented in Extended Data Fig. 9, show increases in mitochondrial mass and signalling. Here, we focused primarily on the acute mitochondrial impact of 5FU, and although we arrived at the combination of 5FU and mitochondrial inhibitors (more specifically, 5FU and metformin) somewhat serendipitously, we have now generated evidence to support the use of 5FU and metformin combinations not only in patients with resistant or relapsed disease as previously suggested by others, but also in treatment-naive, first-line chemotherapy settings. Using complex CRC GEMMs with proficient immune signalling, we demonstrate an increase in cell death and survival in these heterogeneous tumours, more akin to a tumour histology than xenograft or PDX counterparts^21,78^. Our choice of metformin as a proof-of-concept test as a CI inhibitor^79^ in these orthotopic models was based on several factors: (1) it could easily be taken forward safely into in vivo studies with known target concentrations; (2) the specific CI inhibitor IACS-010759 was recently reported to promote neurotoxicity, making this an unattractive agent for combination therapy in CRC given the neurotoxicity long-recognized with oxaliplatin treatment^51^; (3) the more effective combination partner, phenformin, has been linked to increased lactic acidosis in patients^80^, which is to be avoided in patients with CRC; and (4) metformin is safe, well-tolerated and cost-effective, making it an attractive addition to a cytotoxic therapeutic regimen, and it can be administered orally alongside infusional 5FU. Metformin treatment has previously been explored clinically in CRC, but contradictory findings have been reported^81–83^. Retrospective studies suggest a beneficial effect on CRC risk and recurrence in diabetes; however, the effects of these drugs on tumours in patients with diabetes may differ from those in individuals without diabetes, given differences in central metabolic programmes and confounding effects related to overall mortality. Furthermore, heterogeneity in tumour stage (or indeed, location), patient populations and treatment dose and duration is likely to confound reported response data, particularly given the links between changes in oxidative metabolism and metastatic spread^84–87^. Although a small number of randomized trials investigating metformin use in patients with CRC are ongoing, these are in unselected populations and may also include other interventions (such as aspirin and physical activity). Therefore, clinical testing in controlled populations is ultimately required to assess whether this preclinical efficacy is also observed in patients with CRC, both first-line in high-risk patients with high oxidative metabolic signalling (Fig. 4) and with a chemotherapy lead-in using mitochondrial biogenesis induction as a biomarker of vulnerabilities. As more specific ETC-targeted drugs enter clinical pipelines, these can also be deployed in defined cohorts of patients with CRC to examine combination efficacy.

The mechanism of action of metformin can be varied and has effects beyond CI inhibition, depending on concentrations used in vitro^88^. However, metformin collapses the oxygen consumption rate (OCR) in our cell lines; we see limited change in AMP/ATP ratios to trigger AMPK; and, most importantly, we see the same senitization to 5FU with other CI inhibitors, such as IACS-010759, phenformin and carboxyamidotriazole, as we do with metformin. Furthermore, other complex inhibitors show the same sensitization, and genetic loss of mitochondrial CI/IV phenocopies CI inhibition. Therefore, we can conclude that the inhibition of mitochondrial activity by metformin is probably the driver of the phenotype observed here.

Potent inhibition of CI by IACS-010759 resulted in neurotoxicity at concentrations required for therapeutic benefit^51^. However, our data suggest that such inhibitors may have more utility when given in combination with 5FU-based chemotherapy. When treated with 5FU, cancer cells specifically increase mitochondrial content to buffer nucleotide stress, whereas stromal cells do not, meaning that cancer cells become intrinsically more dependent on mitochondrial metabolism. Indeed, the concentrations of all ETC complex inhibitors required to inhibit 50% of cancer cell growth were significantly reduced when cells were exposed to 5FU (Fig. 5e). This provides a potentially greater window of opportunity to lower the dose required to provide therapeutic benefit while sparing normal tissues from unwanted side effects.

Our data demonstrate that the adaptive metabolic response to 5FU is mediated through the inhibition of TS function, as we recapitulate these phenotypes by genetic silencing or targeted inhibition of TS using antifolates. Over five million patients are treated with TS inhibitors annually^89,90^, expanding our findings beyond the CRC field to other disease indications that use pemetrexed or methotrexate, such as lung cancer. Indeed, we found a similar mitochondrial response to pemetrexed treatment in three independent KRAS mutant non-small cell lung cancer cell lines (Extended Data Fig. 5b) as well as KRAS wild-type models (Extended Data Fig. 2d,i,j). This finding highlights not only the key role that mitochondrial metabolism has in modulating response and resistance to TS inhibitors, but the wider opportunity afforded to enhance TS inhibitor-mediated anti-cancer effects by targeting mitochondrial metabolism using metformin or other compounds with similar mitochondrial impact. Furthermore, given that we have demonstrated that this mitochondrial adaptation is to promote rebalancing of pyrimidine levels, mitochondrial biogenesis could also be used to identify patient cohorts likely to benefit from UCK2 inhibitor combinations. UCK2 inhibitors are still in preclinical development, but are positioned in combination with 5FU or other cytotoxics that alter nucleotide pools.

In summary, we present a panoptic analysis of the role of mitochondrial metabolism in dictating sensitivity to 5FU, the most commonly used chemotherapy worldwide. We demonstrate mechanistically the need for increased mitochondrial function, facilitated by mTOR–PGC1α-mediated mitochondrial biogenesis, to support rebalancing of pyrimidines via UCK2 to support cellular survival in the face of continued 5FU treatment. We show that the heterogeneity in mitochondrial function and signalling observed across CRC samples could explain, and be used to predict, poor clinical responses in patients with locally advanced CRC. We demonstrate applicability beyond 5FU treatment in CRC to any TS-targeted therapeutics in other disease subtypes. Finally, we demonstrate the potential of rationally designing targeted combination therapy regimens for CRC. With the compelling preclinical data presented herein, a clinical trial demonstrating efficacy and mechanism of action in patients with CRC is in development.

## Methods

The research presented here complies with all relevant ethical regulations; human and animal study protocols were approved by the Queen’s University Belfast (QUB) Faculty Research Ethics Committee or the Animal Welfare and Ethical Review Body.

### Cell culture

The HCT116 CRC cell line was obtained from ATCC. Other cells were provided by co-authors or collaborators (SW480 and HT29 were provided by McDade and Longley (QUB); U2OS was provided by Butterworth (QUB); hMSC was provided by Ryan (University of Galway); and 143B mitochondrial cybrids were provided by Greaves (University of Newcastle)). Cells were maintained in DMEM (HCT116, SW480, U2OS), MEM-α with 1 ng ml^−1^ fibroblast growth factor 2 (Peprotech, hMSC) or DMEM with 1% non-essential amino acid (Gibco) and 50 μg ml^−1^ uridine (Sigma-Aldrich) supplementation (143B), with 10% dialysed foetal bovine serum (dFBS), 50 U ml^−1^ penicillin and streptomycin (ThermoFisher, 15070063) and 1 mM sodium pyruvate (ThermoFisher, 11360070). Cell lines were maintained at 37 °C in a humidified incubator with 5% CO_2_ and routinely tested for mycoplasma using MycoAlert Kit (Lonza). 5FU-resistant HCT116 cells were generated by chronic exposure to 5FU. Glucose/galactose adapted cells were cultured in DMEM containing 10% dFBS, 100 U ml^−1^ penicillin and 100 μg ml^−1^ streptomycin, 1 mM sodium pyruvate and 25 mM glucose (Sigma-Aldrich, G7021-1KG) or 25 mM galactose (Sigma-Aldrich, G5388-500G) for a minimum of 1 month before being used experimentally. siRNA transfections were performed using Lipofectamine 2000 and ON-TARGETplus Human SMARTpool siRNAs (Dharmacon) against *TYMS* and a scrambled control pool, according to the manufacturer’s instructions.

### Organoid culture

Organoid lines were generated by Sansom (CRUK Scotland Institute) and Kerr (QUB) labs from AKP mice (Villin-CreER^T2^; *Apc*^fl/fl^; *Kras*^G12D/+^; *Trp53*^fl/fl^) as previously described^20,91^ 3 days post i.p. administration of tamoxifen (80 mg kg^−1^). Organoids were resuspended in Matrigel (Corning), plated in six-well plates and supplemented with Advanced DMEM/F-12 medium (ADF; ThermoFisher, 11320033) with 2 mM L-glutamine, 10 mM HEPES (Sigma-Aldrich, H3375-25g), 50 U ml^−1^ penicillin–streptomycin, 1× B27 (ThermoFisher, 12587-010), 1× N-2 (ThermoFisher, 17502-048), 50 ng ml^−1^ EGF (Peprotech, AF-100-15) and 100 ng ml^−1^ Noggin (Peprotech, 250-38). 5FU-resistant organoids were generated through chronic exposure to 5FU. To evaluate the effect of CI inhibition on growth, organoids were seeded as fragments in Matrigel and treated the following day with water (CTRL), 5 μM 5FU, 3 mM metformin or a combination of 5FU and metformin for 72 h. Organoid diameter was measured using ImageJ, and the average was calculated.

2D CRC cell line was obtained by dissociating organoids cultured in Matrigel on a six-well plate. Organoids were incubated in Cell Recovery solution (354253) for 30 min at 4 °C. The recovered organoids were incubated in a 1:1 ratio with TypLE Express (1×; 12605-010) at 37 °C for 5 min after vigorous pipetting. This solution was neutralized with ADF supplemented with 2 mM L-glutamine, 20% FCS and 50 U ml^−1^ penicillin and streptomycin. After more vigorous pipetting, cells were pelleted by centrifugation at 400 RCF for 5 min and seeded onto a 24-well plate. Media (ADF plus 20% FCS) was changed every 2 days, and cells were split twice a week. Once cells were proliferating, they were moved up to higher cell volumes and adapted to DMEM cell culture media with 20% dFBS, 50 U ml^−1^ penicillin and streptomycin and 1 mM sodium pyruvate for at least 1 week before experimental setup.

### Cell reagents

Cells and organoids were treated with the following compounds as indicated: 5FU (Abcam, ab142387-5g; 0.5–5 µM), metformin (Cayman Chemicals, 13118; 0-50 mM), IACS-10759 (Selleckchem, S8731; 0–50 μM), zVAD-fmk (Sigma-Aldrich, 627610; 10 µM), siTYMS, siUCK2 and siSC siRNA SMARTpools (L-004717-00-0005, Horizon Discovery; 10 nM), oxaliplatin (Belfast City Hospital, 1 µM), SN38 (Selleckchem, S4908; 5 nM), methotrexate (Merck, M1000000, Batch 8.0; 15 nM), pemetrexed (Merck, PHR1596, lot LRAC1932; 0.5 µM), raltitrexed (Selleckchem, S1192, lot S119204; 5 nM), BAY-2402234 (Cambridge Bioscience, CAY33259-1mg; 20 nM), rapamycin (Sigma-Aldrich, 553210; 10 µM), Torin1 (100 nM, kindly gifted by the Jafarnejad lab at QUB) and uridine (Merck, U3003; 30 µM).

#### Cell viability by SRB

Cell viability was measured using sulforhodamine B (SRB; Sigma-Aldrich, S1402-5G)^92^. For the SRB assay, cells were seeded (5,000 cells per well) in a 96-well plate and then treated with the indicated drugs. At the experimental endpoint, the cells were fixed with 10% trichloroacetic acid (Sigma-Aldrich, T6399-500G) and stained with 0.05% SRB for 1 h and then washed repeatedly with 1% acetic acid. The protein-bound dye was dissolved in 10 mM Tris base solution (ThermoFisher, 15504-020) to obtain a reading at 510 nm. The effect on cell growth was assessed as a percentage of cell viability, with vehicle-treated cells considered 100% viable. The IC_50_ of each drug was calculated using GraphPad software.

#### High-content fluorescent microscopy

This method was used to assess concurrent growth arrest and apoptotic cell death in response to mitochondrial inhibitors with or without 5FU. Cells were seeded into a 384-well glass-bottom black plate (IBL, P384-1.5H-N) and left to adhere overnight. After 72 h treatments, 0.5 μg ml^−1^ propidium iodide (Sigma-Aldrich, P4864) and 1.4 μg ml^−1^ Hoechst 33342 (Invitrogen, H3570) were added to cells. Images were then taken on the ArrayScan XTI Live High Content Platform (ThermoFisher). Drugs were normalized to paired vehicle controls, followed by 0 µM 5FU. GraphPad Prism was used to calculate the area under the curve (AUC) for both parameters with or without 5FU, and then the difference in AUC was calculated as (AUC +5FU) − (AUC −5FU). A negative value for the valid object count signifies synergism of the CI inhibitors and 5FU, with the opposite true for per cent propidium iodide. Growth arrest was determined as 1 − average fraction of control.

### Mitochondrial profiling

Cells were stained with NAO (ThermoFisher, A1372), tetramethylrhodamine, ethyl ester perchlorate (TMRE; Sigma-Aldrich, 87917) and MitoSOX Red (ThermoFisher, M36008). NAO (200 nM) or TMRE (25 nM) was added to the culture medium and incubated for 1 h at 37 °C in 5% CO_2_. MitoSOX Red (5 µM) was added to the medium and incubated for 30 min at 37 °C in 5% CO_2_. Cells were washed, collected and resuspended in 0.3 ml of PBS. Cells were analysed by flow cytometry using a BD LSR II instrument. Cells were gated for singlets, and the GeoMean NAO, TMRE or MitoSOX Red fluorescence was evaluated using FlowJo software.

Genomic DNA was isolated using buffer (100 mM Tris-HCl, 5 mM EDTA, 0.2% SDS, 200 mM NaCl + proteinase K) and precipitation with isopropanol. Real-time PCR was conducted to quantify the mitochondrial DNA relative to nuclear DNA as previously reported. A total of 250 ng of target DNA was used per reaction. Primer sequences are available in the Supplementary Table 2).

### Extracellular flux analysis in NDI1 overexpressing cells

The Agilent Seahorse XF96 system was used to assess extracellular flux. Overexpression of NDI1 in HCT116 was achieved by transfecting 1 × 10^6^ cells with plasmid PXMS-NDI1 DNA (PMXS-NDI1 was a gift from D. Sabatini (Addgene, plasmid 72876; http://n2t.net/addgene:72876; RRID:Addgene_72876)) for 48 h before seeding for flux analysis at 10,000 cells per well and allowed to adhere overnight in full culture media under normal conditions. Probe plates were prepared by adding 200 μl of PCR-grade H_2_O, and calibration buffer was added to a 50 ml Falcon tube. Both were incubated overnight at 37 °C, without CO_2_. The following day, assay media was prepared by supplementing specialized DMEM pH 7.4 (Agilent, 103575-100) with 10% dFBS, 1% penicillin–streptomycin, 25 mM glucose and 2 mM L-glutamine. The 96-well assay plate media was then replaced with 180 μl per well of freshly prepared assay media, and the plate was incubated at 37 °C, no CO_2_, for 1 h to eliminate residues of carbonic acid. The H_2_O was replaced with 200 μl XF calibrant in the probe plate, and it was incubated at 37 °C, no CO_2_, for 1 h. The probe plate was then prepared with mitochondrial inhibitors: 25 μl 5 μM rotenone (Sigma-Aldrich, 45656) and 5 μM antimycin A (Sigma-Aldrich, A0149). The probe plate was then inserted into the Seahorse XF analyser and, after 15 min of calibration, the cell plate was inserted into the analyser. OCR was determined by six measurement cycles before and after injection: 3 min mixing, 3 min waiting and 3 min measuring. Results were normalized to SRB.

### Mitochondrial complex activity

The enzyme activity of CI was determined by a colourimetric analysis following the manufacturer’s protocol (Abcam, ab109721). A Seahorse XFe96 Analyser (Agilent Technologies) was used to assess mitochondrial complex-specific OCR of HCT116 CRC cells. In brief, 3,000 cells were seeded in a 96-well Seahorse culture plate, with six wells included per biological replicate. These were allowed to adhere overnight in full culture media (DMEM (41965039) with 4 mM L-glutamine, 10% dFBS (26400044), 1 mM sodium pyruvate (11360039) and 50 U ml^−1^ penicillin–streptomycin (15070063)) under normal cell culture conditions. After 24 h, fresh cell media with or without 5FU was applied to the cells and the cells were treated for a further 72 h. The day before the assay, Seahorse probe plates were prepared by adding 200 μl distilled water, and calibration buffer was added to a 50 ml Falcon tube. Both were incubated overnight at 37 °C, without CO_2_. On the day of assay, the mannitol and sucrose (MAS) buffer (1×) was prepared by diluting sterile-filtered MAS 3× in distilled water, resulting in a solution with the following final concentrations: 220 mM mannitol, 70 mM sucrose, 10 mM KH_2_PO_4_, 5 mM MgCl_2_, 2 mM HEPES, 1 mM EGTA and 0.2% w/v fatty-acid free BSA (3117057001) (pH 7.2), and warmed to 37 °C. Approximately 45 min before running the assay, the distilled water in the probe plate was replaced with 200 μl XF calibrant and incubated at 37 °C, no CO_2_. The probe plate was then prepared with the substrates and mitochondrial inhibitors to probe the OCR driven by specific ETC complexes. For CI assessment, 20 μl pyruvate (107360; 50 mM), malate (M0875; 25 mM), ADP (A5285; 10 mM) and plasma membrane permeabilizer (102504-100; 10 nM) were added to port A, 22 µl oligomycin (O4876; 10 µg ml^−1^) was added to port B and 25 µl rotenone (10 µM) was added to port C. For CII assessment, 20 µl succinate (100 mM), ADP (10 mM), rotenone (10 µM) and plasma membrane permeabilizer (10 nM) were added to port A, 22 µl oligomycin (10 µg ml^−1^) was added to port B and 25 µl antimycin A (A0149; 200 µM) was added to port C.

The probe plate was inserted into the Seahorse XF analyser to begin assay calibration. To minimize the time that cells are kept in MAS buffer, just before calibration finished, the cell media was removed, and the cells were washed once with MAS (1×) buffer before addition of 180 µl MAS (1×) buffer. Once calibration had finished, the cell plate was inserted into the analyser, and OCR was determined at basal and mitochondrial stress conditions by triplicate measurement cycles of 0.5 min mixing, 0.5 min waiting and 2 min measuring, carried out at baseline and after each port injection. No equilibration step was included before measurements. Results were normalized to either average SRB staining on a surrogate cell culture plate or to cell confluency measurements obtained on the Incucyte (Sartorius). State 3 respiration was calculated as the first OCR measurement after substrate injection minus the first OCR measurement after rotenone (for CI) or antimycin A (for CII) injection (measurement 4 − measurement 10).

### Cytochrome *c* release assay

Cells were fractionated to obtain cytoplasmic and mitochondria-enriched fractions using Dounce homogenization and differential centrifugation as per the Abcam cytochrome *c* release kit (ab65311). Immunoblotting was performed with 10 µg of cytoplasmic and mitochondrial-enriched lysate as per the immunoblotting methods described below relative to whole-cell lysates.

### Immunoblotting

Cells were washed with PBS and lysed with lysis buffer (150 mM NaCl, 20 mM EDTA, 50 mM Tris-HCl, 0.5% NP-40) containing protease and phosphatase inhibitors. Protein concentration was determined by BCA assay (ThermoFisher). After addition of loading buffer, lysates were vortexed, and supernatants were heated to 95 °C for 5 min. Equal amounts of protein lysates were loaded on Novex WedgeWell Tris-glycine or Bis-Tris gels (ThermoFisher) and blotted onto nitrocellulose or PVDF Trans-Blot Turbo membrane (0.2 µm, Bio-Rad) according to standard protocols. Membranes were incubated with the appropriate primary antibody overnight at 4 °C. Antibody details can be found in the Supplementary Table 1. Membranes were washed three times with PBS-T and incubated for 1 h at 20–22 °C with the corresponding horseradish peroxidase-labelled secondary antibodies: goat anti-rabbit IgG (H + L) (Vector Laboratories, BA-1000; 1:2,000) and rabbit anti-rat IgG (H + L) (Vector Laboratories, BA-4000; 1:2,000). Protein expression was analysed and detected using the Western Lightning Plus-ECL, Enhanced Chemiluminescence Substrate (PerkinElmer, NEL105001EA) and G:BOX ChemiXX6 gel doc system with GeneSys image software (Syngene). A representative loading control (actin/vinculin) is shown in each figure, with panels from Figs. 3g and 8f generated concurrently. Densitometry was completed using ImageJ/FIJI. Blots shown are representative of three or more independent experiments, with specific repeats detailed in each figure legend and shown and quantified in the Source data file.

### LC–MS metabolomics analysis

#### Sample preparation

HCT116s cells (1 × 10^5^) treated with or without 5 μM 5FU were cultured in DMEM for 24 h and 72 h or supplemented with media containing uniformly labelled ^13^C_6_-glucose (25 mM) for 6 h before sampling. At room temperature, media was removed, and cells were washed twice with 0.9% NaCl (aq). On ice, cells were scraped and collected in extraction buffer containing methanol:acetonitrile:H_2_O (4:4:2), 15 µM glutaric acid and 0.5% formic acid. This mixture was left on ice for 5 min before addition of neutralization buffer (final concentration, 0.17 mM ammonium carbonate (5330050050) in LC–MS-grade H_2_O) and gently mixed before being incubated on dry ice for 15 min. The samples were thawed on ice, centrifuged at 15,700 RCF for 5 min at 4 °C and the supernatant was collected for downstream processing. Samples were dried using an Eppendorf Concentrator Plus at 30 °C for up to 7 h. Samples were reconstituted in 100 μl 50% acetonitrile (aq) before centrifugation at 300*g*. The supernatant was transferred to vials for MS analysis.

#### Targeted LC–MS analysis

Samples were analysed using an Agilent Infinity II HPLC and an Agilent G6545A quadrupole time-of-flight mass spectrometer. Separation was achieved on a Waters Premier BEH Z-HILIC column (1.7 µm, 2.1 ×150 mm) using 20 mM ammonium bicarbonate solution with 0.1% ammonium hydroxide and 0.1% InfinityLab deactivator as mobile phase A and H_2_O/ACN (1:9 ratio) with 0.1% InfinityLab deactivator as mobile phase B. The LC flow was set at 0.2 ml min^−1^, and the gradient was as follows: 10% A held for 2 min, 35% A at 18 min, 70% A at 22 min, 90% A at 22.1 min and held for 2.9 min, 10 % A at 25.1 min and held for 5 min. Data were post-processed using Agilent MassHunter Profinder 10. Normalization of data to an internal standard was performed using an in-house R script, then values were normalized to protein content per treatment condition. Retention times for non-natural metabolites, including 5FU, were determined by single compound reference sample analyses, and both molecular weight and retention times were used for analysis in larger LC–MS experiments.

#### ^13^C_6_-glucose labelling analysis

LC separation was performed using an Agilent InfinityLab Poroshell 120 HILIC-Z (2.1 ×100 mm, 2.7 μm), PEEK-lined column on an Agilent G7167B and G7120A multisampler and pump, respectively. Separation was achieved using a gradient of 10 mM ammonium acetate in water (mobile phase A; pH 9.0) and 10 mM ammonium acetate in 90% acetonitrile (aq) (mobile phase B (MPB); pH 9.0). The flow rate was maintained at 0.5 ml min^−1^, and the gradient was as follows: 0.0 min 100% of MPB, 0.0–11.5 min 70% of MPB, 11.5–12.5 min 60% of MPB and 12.5–15.5 min 100% of MPB. The mass spectrometer (Agilent Dual ESI G6545B quadrupole time-of-flight) was operated in negative ion mode. Spectra were analysed using Agilent MassHunter Qualitative Analysis and Agilent MassHunter Profinder software by referencing to an internal library of compounds. Relative metabolite abundance was calculated as the percentage of the total metabolite pool.

### Mitochondrial imaging

#### Immunofluorescence

Imaging was performed on a STED-capable Stellaris DMI8 platform (Leica). Cells were prepared and stained as per the Leica protocol (https://www.leica-microsystems.com/science-lab/life-science/the-guide-to-sted-sample-preparation). In brief, cells were plated onto coverslips and treated with or without 5FU at the indicated doses for 72 h. Cells were fixed with 2% paraformaldehyde (15 min), permeabilized with 0.1% Triton X-100 (10 min) and blocked with 2% BSA (1 h). Primary antibody rabbit anti-TOM20 (Abcam, ab186734; 1:500) or mouse anti-DNA (Progen, 61014; 1:500) was incubated overnight at 4 °C. Secondary antibody donkey anti-rabbit Alexa Fluor 555 (Invitrogen, A31572; 1:500) or goat anti-mouse Alexa Fluor 488 (Invitrogen, A11001; 1:500) was incubated for 1 h at room temperature. Coverslips were mounted onto glass slides using ProLong Gold anti-fade mountant (Invitrogen, P36930). Imaging took place at least 24 h after mounting.

#### Transmission electron microscopy

HCT116 cells were treated with 5 µM 5FU for 72 h before being pelleted and fixed with 3% glutaraldehyde (Agar Scientific) in cacodylate (CACO). Cells were then washed three times for 10 min each in CACO buffer. Cells were post-fixed for 1 h with 1% osmium tetroxide (Agar Scientific) in CACO buffer, dehydrated in a graded series of methanol and embedded in LR white resin. Samples were sectioned using an ultramicrotome (Leica EM UC6) to 60–90 nm, transferred to copper grids and left to dry before imaging. Electron micrographs were obtained using a transmission electron microscope (Hitachi) at ×40,000 magnification. Analysis was carried out using ImageJ software. Images with fewer than five mitochondria were discounted. The number of cristae was manually counted. The aspect ratio was calculated as major axis/minor axis.

### BH3 profiling

HCT116 cells were treated with 5 µM 5FU for 24 h. BH3 profiling was then performed using whole-cell (JC-1) plate-based fluorimetry. BH3 peptides/mimetics in DTEP buffer (300 mM trehalose, 10 mM HEPES-KOH, 0.1% w/v BSA, 1 mM EDTA, 1 mM EGTA, 80 mM KCl, 5 mM succinate, final pH 7.4) were plated at 70 μM l^−1^ (unless otherwise stated) in triplicate in a black 384-well plate. The sequence of the BH3 peptides has been previously reported^93,94^. HCT116 cell lines were collected, washed and resuspended in DTEP buffer. An equal volume of dye Mastermix (1 μM JC-1, 0.005% digitonin, 10 μg ul^−1^ oligomycin, 5 mM β-mercaptoethanol in DTEP buffer) was added, and after 10 min at room temperature, the cells were added on top of the peptide template at a concentration of 40,000 cells per well. Mitochondrial potential loss was measured using the Varioskan kinetic plate reader at excitation 545 nm and emission 590 nm for 3 h at 21 °C (kinetic measurements every 5 min). Mitochondrial depolarization was normalized to dimethyl sulfoxide control (0%) and positive control FCCP (carbonyl cyanide 4-(trifluoromethoxy)phenylhydrazone; 100%).

### Gene expression profiling

#### Sample preparation and RNA-seq

Total RNA was extracted from HCT116 cells and AKP organoids using the Roche KAPA RNA HyperPrep kit with RiboErase, according to the manufacturer’s protocol. The kit is designed for NGS library construction from 25 ng to 1 μg of total RNA and depletes both cytoplasmic (5S, 5.8S, 18S and 28S) and mitochondrial (12S and 16S) ribosomal RNA species. Library quality control was performed on the fragment analyser (HS NGS kit) and Qubit. Finally, the libraries were sequenced using the Illumina NovaSeq 6000 sequencing system. The indexes used for these libraries were from the KAPA Unique Dual-Indexed Adapter kit.

#### Data analysis

RNA-seq reads were aligned to the human genome (hg37) using the STAR aligner, and the number of reads mapping to genes annotated in Gencode build 37 (v22) was calculated using HTseq. DEGs were identified using the DESeq2 R package, and results were visualized as a volcano plot using the ggplot2 R package (https://ggplot2.tidyverse.org). Metabolic genes were defined based on a curated list^6,40^. GSEA was performed using the clusterProfiler and msigdbr R packages (https://CRAN.R-project.org/package=msigdbr). Heatmaps were generated using Morpheus (Broad Institute). All data were generated from at least two pseudo-biological replicates per timepoint.

#### Taxonomy cohort

The gene expression series matrix was downloaded directly from GEO using accession number GSE103479. The datasets had already been robust multi-array average-normalized in batches using the makecdfenv, affy and limma R packages, and batch-corrected using the ComBat method in the sva R package. Patients were subdivided by whether they received surgery alone or adjuvant 5FU-based chemotherapy and subsequently grouped based on the median overall survival into either ‘good outcome’ or ‘poor outcome’. Overall survival comparisons were performed by Kaplan–Meier analysis using log-rank testing for patients separated based on the mean OxPhos score generated using single-sample GSEA. Both cohorts (surgery or surgery + adjuvant 5FU-based chemotherapy) used patient data from males and females.

#### Real-time PCR

Total RNA was isolated from the cells using the Roche High Pure RNA isolation kit (Roche Life Science, 11828665001) and reverse transcription was performed using the iScript cDNA synthesis kit (Bio-Rad, 1708891) according to the manufacturer’s instructions. Quantitative PCR assays were performed using 480 SYBR Green Master reagent mix (Roche Life Science, 4707516001, murine probes) or TaqMan assays (human ETC genes) on a real-time fluorescence qPCR instrument (LightCycler 480II, Roche Life Science). The expression of relative mRNA was normalized to that of the housekeeping gene 18S.Primer sequences used are provided in Supplementary Table 2.

### In vivo studies

#### Experimental work

Animals were maintained under specific-pathogen-free conditions and in compliance with the Northern Ireland Department of Health (under PPL 2874 and 2960) regulations with approval from the QUB Animal Welfare and Ethical Review Body. Mice were housed with no more than five animals per cage and were kept on an ad libitum diet. Husbandry conditions included a temperature of 21–23 °C; humidity in, between 43% and 48%; humidity out, between 50% and 59%; light cycle of 12 h light to 12 h dark; and chow diet T.2918.4x5R sourced from Inotiv. Sample sizes are shown in the figures or legends, based on power calculations for output measurement to detect *P* < 0.05 with 80% power. Data were assumed to be normally distributed. GEMM studies used both male and female mice induced at 8–10 weeks old, but xenograft studies used only females, transplanted at 8 weeks old.

For human xenograft studies, 2 × 10^6^ HCT116 cells were transplanted 1:1 in Growth Factor Reduced Matrigel (Corning, CLS356231) into athymic nude mice (purchased from Inotiv), and the volume was calculated using the formula ½(*a* × *b*^2^). When tumours reached ~100 mm^3^ on average per genotype, animals were randomized by ranked allocation across all groups and given 5FU (10 mg kg^−1^), metformin (250 mg kg^−1^) or vehicle (PBS) daily by i.p. injection (5FU) and oral gavage (o.g.) administration (metformin) for 11 days. The growth limit was tumour Geometric Mean Diameter of <10, and no tumour exceeded this measurement. Data collection and analysis were not performed blind to the conditions of the experiments, given the expertise required and the number of operators. Digital pathology analysis was completed by two independent researchers to ensure consistency.

For GEMM studies, intestinal tumours were generated through i.p. injection of 80 mg kg^−1^ tamoxifen in 8–10-week-old A(Het)KP mice (Villin-CreER^T2^; *Apc*^fl/+^; *Kras*^G12D/+^; *Trp53*^fl/fl^) or intracolonic induction of colon tumours by submucosal injection of 100 μM 4-hydroxytamoxifen (Sigma-Aldrich) in 8–10-week-old AKP mice (Villin-CreER^T2^; *Apc*^fl/fl^*;* *Kras*^G12D/+^; *Trp53*^fl/fl^). Treatment was initiated in mice when they displayed clinical signs characteristic of intestinal tumour burden as defined in the relevant licensing documents (for example, hunching, bloody stools). Mice of both sexes were randomly assigned to cohorts and received a single dose of indicated compounds: vehicle (PBS) by i.p. and/or o.g.; 250 mg kg^−1^ metformin (o.g.) alone; and 150 mg kg^−1^ 5FU (i.p.) alone or in combination with metformin. After 72 h, mice were killed, and tissue was collected. Only animals with no tumours at endpoint were excluded from analysis. For survival analysis, AKP tumour-bearing mice were generated as above, and 26 days after induction, they were treated with 150 mg kg^−1^ 5FU (i.p.) ± 250 mg kg^−1^ metformin (o.g.) three times weekly. Animals were killed at the clinical endpoint, and survival was analysed by the Kaplan–Meier method.

#### Tissue preparation, staining and analysis

Intestinal tissues were flushed with PBS and fixed as a Swiss roll in 10% buffered formalin overnight. The tissues were then transferred to 70% ethanol before processing for embedding. All haematoxylin and eosin and immunohistochemistry staining was performed on 5 μm formalin-fixed, paraffin-embedded sections that had previously been heated at 55 °C for 45 min.

Standard protocols were used for haematoxylin and eosin staining. Slides were deparaffinized and dehydrated, and antigen retrieval was performed using either Tris-EDTA or sodium citrate buffer as recommended by the antibody supplier. The slides were then stained with a standard tertiary method: incubated with indicated primary antibodies (TOM20 (Abcam, ab186734; 1:200), VDAC2 (ThermoFisher, PA5-28106; 1:500) and CC3 (Cell Signaling Technology, 9661L; 1:200)) at 4 °C overnight, followed by goat anti-rabbit IgG (H + L) (Vector Laboratories, BA-1000; 1:200) for 1 h and then streptavidin–horseradish peroxidase tertiary antibody (Vector Laboratories, SA-5004; 1:200) for 30 min. Samples were further stained with a DAB kit (Abcam, ab64238) and counterstained with haematoxylin. Images were acquired using brightfield microscopy with a ×20 or ×40 objective lens and analysed using QuPath (v0.5.1). For TOM20/VDAC2 quantification, stain vectors were established for each image, and the mean DAB intensity was calculated for each tumour and mouse. To assess tumour and stroma staining, pixel classification was carried out on each sample to identify tumour and stroma regions. Positive cell detection was used to quantify CC3 staining in tumour samples, calculated from DAB optical density mean.

### CRISPR

GeCKO Library V1 single-guide (sgRNA) library/pool targeting 17,419 genes was a kind gift from the Zhang laboratory. The library was amplified, and lentivirus was produced and titred with adaptations as described previously^95^. In brief, HCT116 cells were infected with the GeCKO v1 human CRISPR knockout pooled library using lentiviral transduction at a multiplicity of infection of 0.3, and populations stably expressing gRNAs were selected for using puromycin and expanded for 8 days to allow for essential gene drop-out. Then, 30 × 10^6^ cells were collected (representing an estimated coverage of 300×), and 30 × 10^6^ cells were plated for treatment the next day with either 5 μM 5FU or media only for a further 11 days. Cells were passaged when they reached 70–80% confluence, again maintaining 30 × 10^6^ cells at each serial passage, with 5FU added in fresh media every 3 days. DNA was extracted and sgRNA inserts amplified as previously described^95^. Adaptors were trimmed from de-multiplexed FASTQ files using Cutadapt (https://pypi.python.org/pypi/cutadapt), aligned to the Gecko V1 design file, and count tables were generated with MAGeCK software V5. Positively and negatively selected sgRNAs were identified at sgRNA, gene and pathway levels using the MAGeCK RRA algorithm.

### Statistical analysis

Data were visualized and statistical analyses performed using Prism 9.5.1 software (Graph Pad). *P* < 0.05 was considered statistically significant. In all cases, experimental groups showed comparable variance. *P* values for unpaired comparisons between two groups with comparable variance were calculated by two-tailed Student’s *t*-test. One-way ANOVA with Dunnett’s, Šídák’s or Tukey’s multiple comparisons was used for analysis between datasets with one independent variable. Ordinary two-way ANOVA was used for analysis that involved two independent variables, followed by Šídák’s or Tukey’s post hoc test for individual comparisons. Kaplan–Meier comparison was used for analysis of survival cohorts. RNA-seq gene expression data were analysed using a negative binomial generalized linear model (DESeq2). On the figures, asterisks indicate significance (*P < 0.05, **P < 0.01, ***P < 0.001, ****P < 0.0001); error bars indicate s.d. or s.e.m., as indicated.

### Reporting summary

Further information on research design is available in the Nature Portfolio Reporting Summary linked to this article.

## Supplementary information

Supplementary Information DESeq data from RNA analysis. All biological replicates and quantification data.

Reporting Summary

Supplementary Data 1 Survival data for Kaplan–Meier curves.

Supplementary Data 2 DESeq data from RNA analysis.

Supplementary Table 1 Details of antibodies used for western blot.

Supplementary Table 2 Details of primers used for qPCR.

## Source data

Source Data Fig. 1 Statistical source data.

Source Data Fig. 1 Unprocessed western blots.

Source Data Fig. 2 Statistical source data.

Source Data Fig. 3 Statistical source data.

Source Data Fig. 3 Unprocessed western blots.

Source Data Fig. 4 Statistical source data.

Source Data Fig. 5 Statistical source data.

Source Data Fig. 6 Statistical source data.

Source Data Fig. 7 Statistical source data.

Source Data Fig. 8 Statistical source data.

Source Data Fig. 8 Unprocessed western blots.

Source Data Extended Data Fig. 1 Statistical source data.

Source Data Extended Data Fig. 1 Unprocessed western blots.

Source Data Extended Data Fig. 2 Statistical source data.

Source Data Extended Data Fig. 2 Unprocessed western blots.

Source Data Extended Data Fig. 3 Statistical source data.

Source Data Extended Data Fig. 4 Statistical source data.

Source Data Extended Data Fig. 5 Statistical source data.

Source Data Extended Data Fig. 6 Statistical source data.

Source Data Extended Data Fig. 7 Statistical source data.

Source Data Extended Data Fig. 9 Statistical source data.

Source Data Extended Data Fig. 9 Unprocessed western blots.

Source Data Extended Data Fig. 10 Statistical source data.

## Data Availability

All RNA-seq data have been deposited in the GEO and are accessible through GEO Series accession numbers GSE272456 (HCT116) and 810 GSE272391 (AKP organoids) or at 10.5281/zenodo.17152295. RNA-seq data of the clinical cohort have previously been deposited at GSE103479 (ref. ^53^) (Supplementary Data Table 1). All schematics were created in BioRender; Kerr, E. https://BioRender.com/n74cqss (2026). Source data are provided with this paper.
