# Supplementary material for: Mitochondrial metabolism determines chemotherapy sensitivity in colorectal cancer
Source: Nat Metab. 2026 Jul 23;8(8):1751–71. doi: 10.1038/s42255-026-01578-w (PMC13489958; doi:10.1038/s42255-026-01578-w)
Supplement: Supplementary file 1 [file 42255_2026_1578_MOESM11_ESM.pdf]

Blots used for **Figure 3c** image

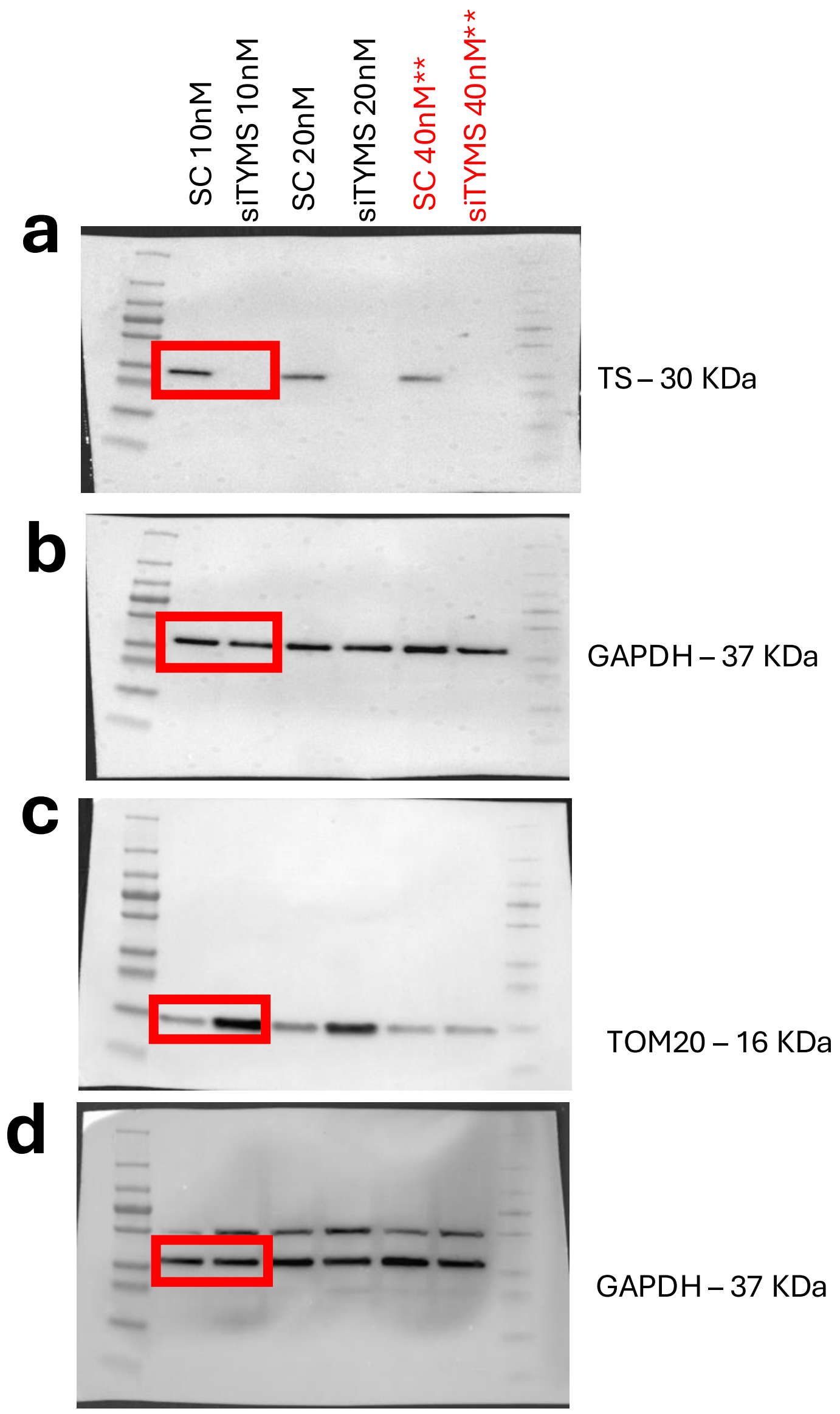

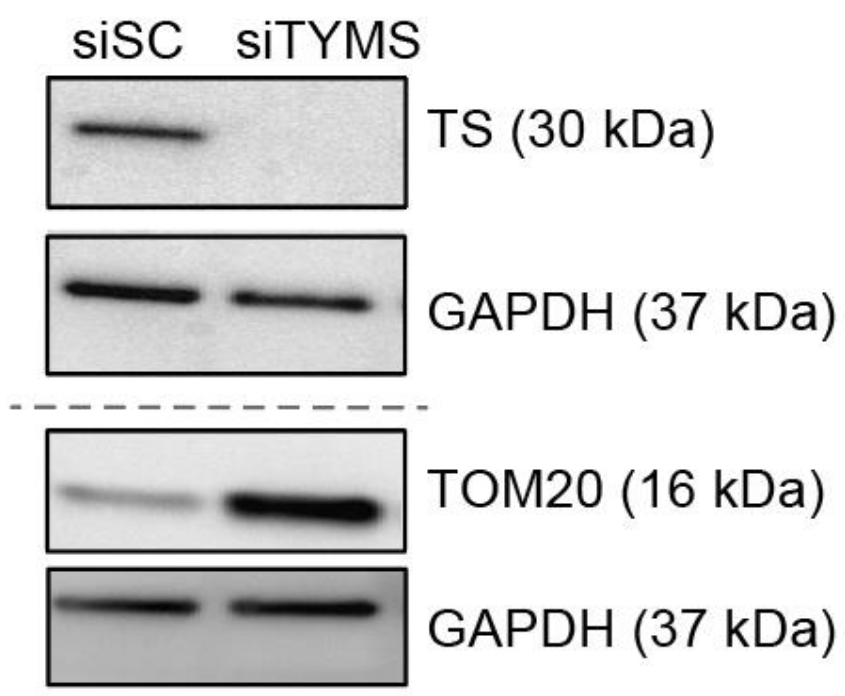

Blots show Western images for independent biological repeats of HCT116 cells transfected with siRNA against Scrambled Control (SC) or *TYMS* at indicated concentrations for 72 hrs for TS (a), TOM20 (c) with GAPDH (b,d) used as loading control. Size is indicated. 10nM data shown, as indicated by red boxes.

Blots used for **Figure 3e** image

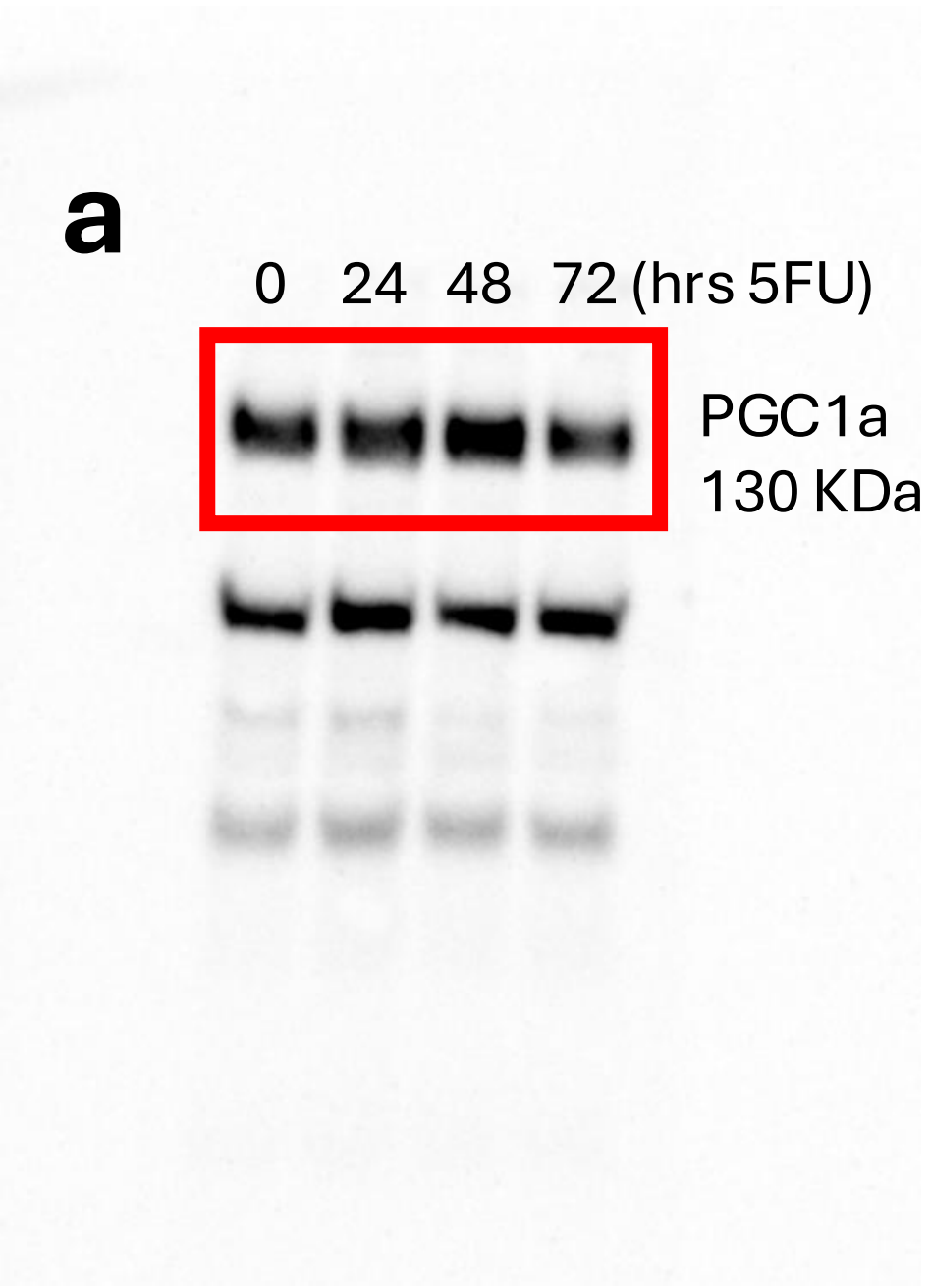

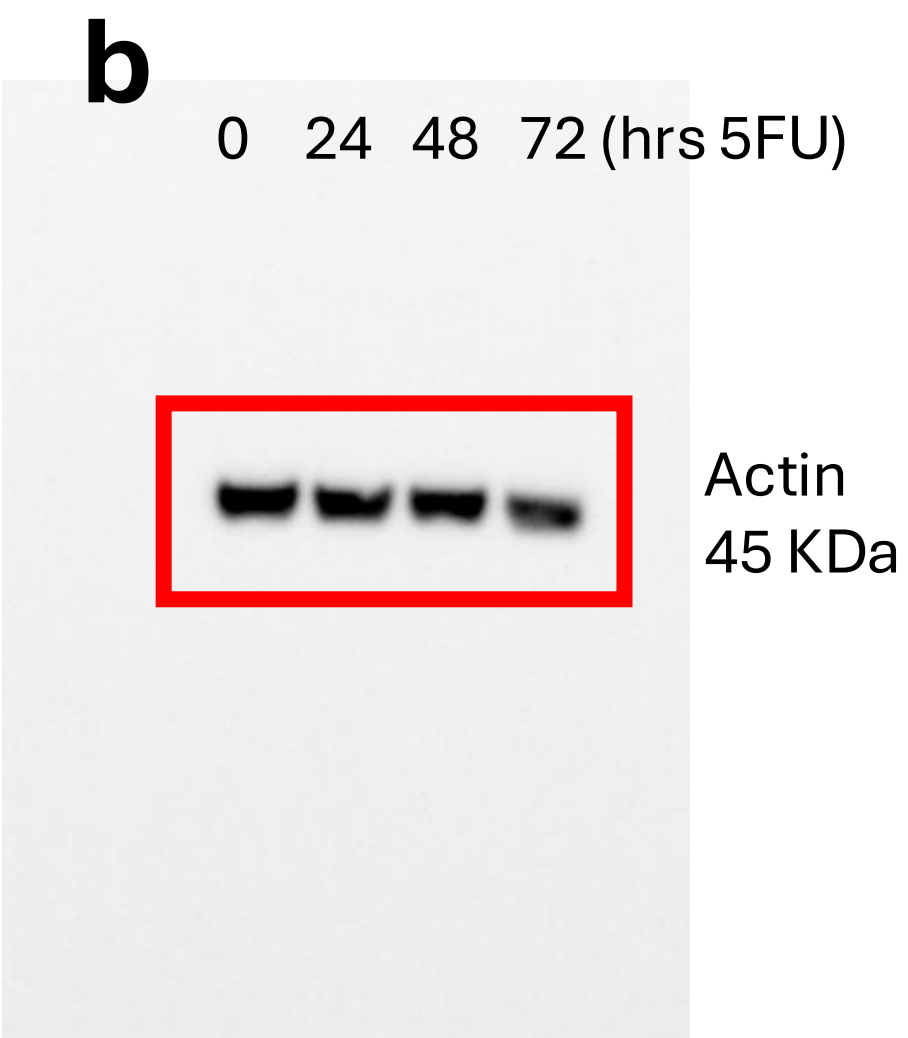

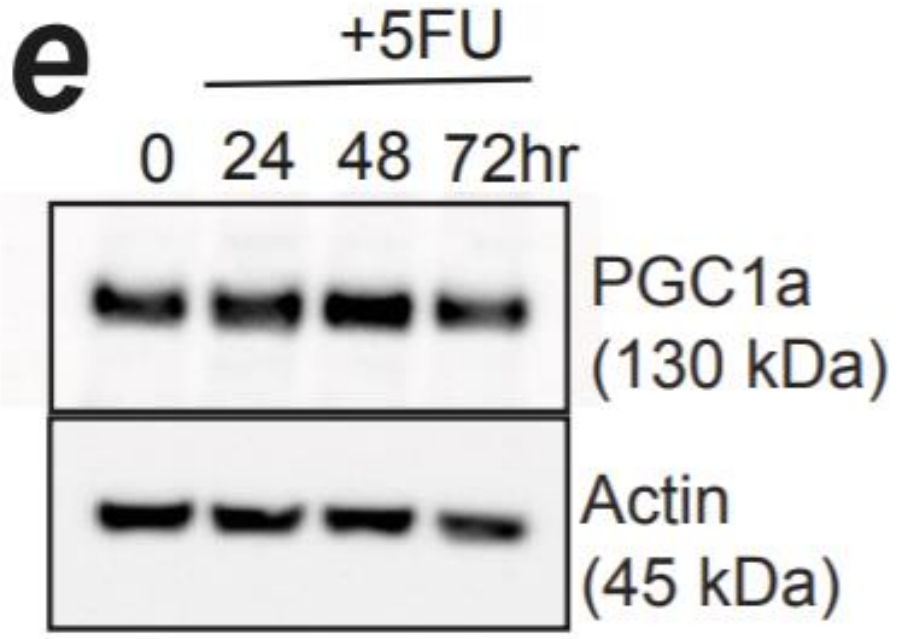

Blots show western images of HCT116 cells treated with 5uM 5FU over 72 hrs for PGC1a (a) and Actin (b) used as loading control. Size is indicated.

Blots used for **Figure 3g** image

0 24 48 72 (hrs 5FU)

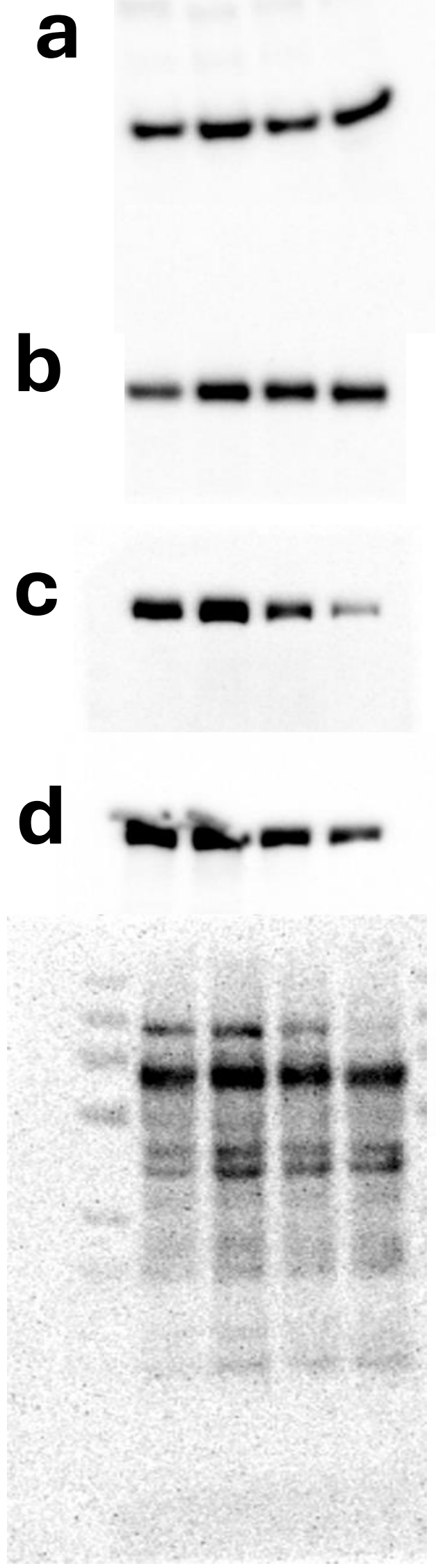

a pAMPK – 62 KDa

b AMPK – 62 KDa

c pACC – 250 KDa

d ACC – 250 KDa

e pS6K – 70 KDa

0 24 48 72 (hrs 5FU)

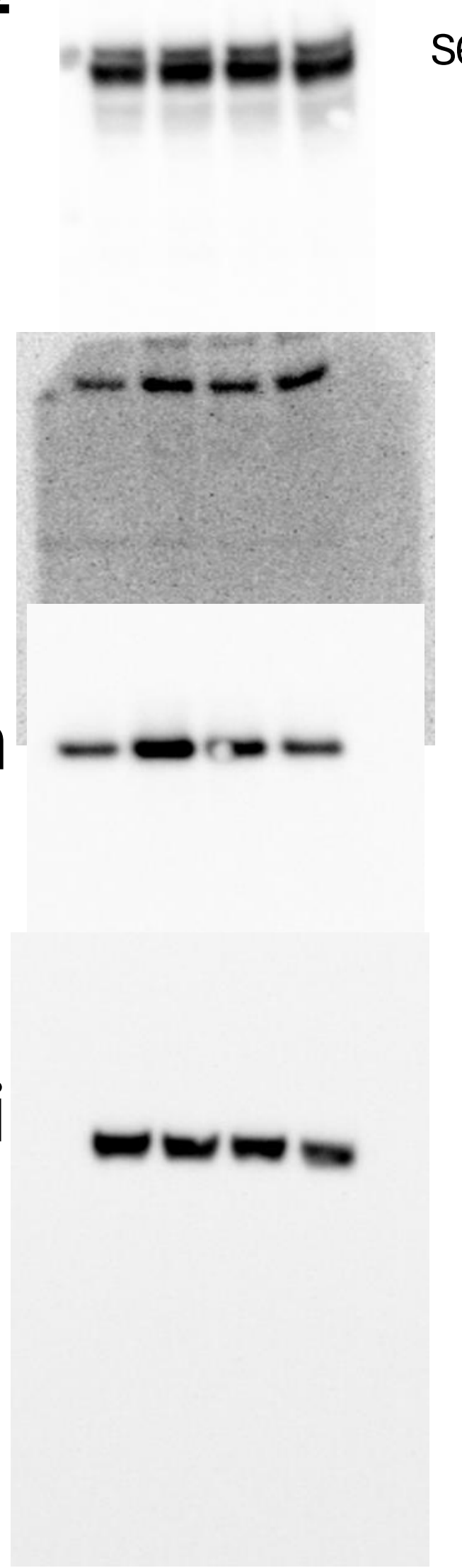

f S6K – 70 KDa

g ps6 – 30 KDa

h s6 – 30 KDa

i Actin – 45 KDa

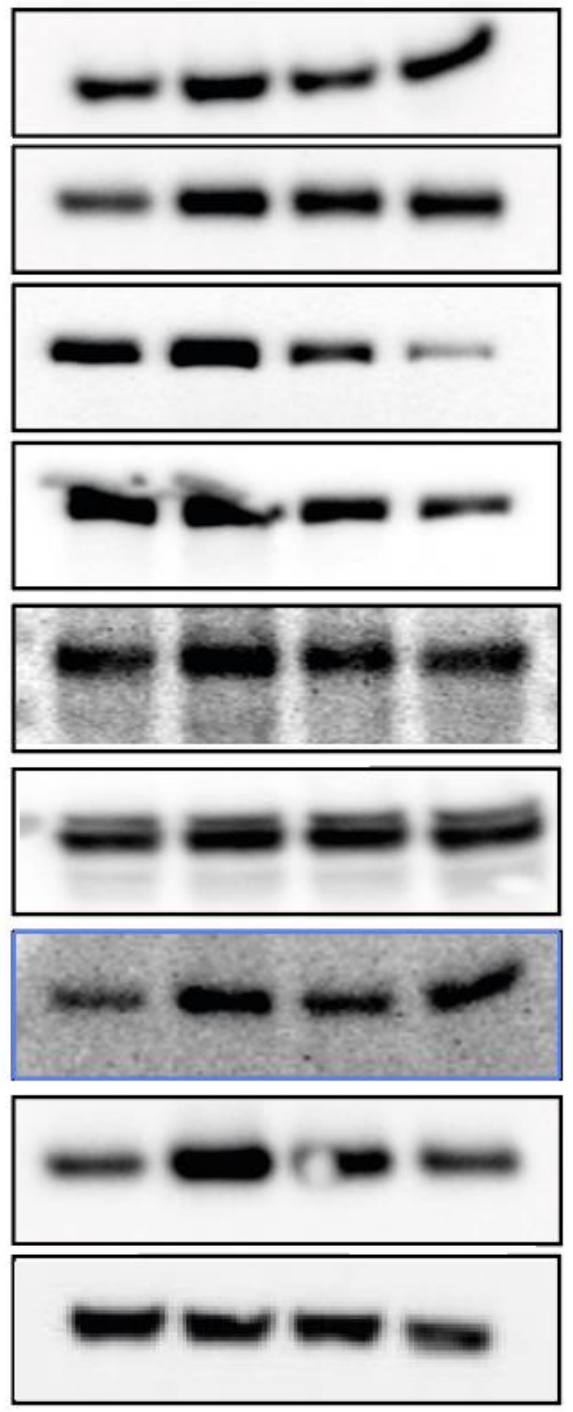

Blots show western images for HCT116 cells treated with 5uM 5FU over 72 hrs for pAMPK (a), AMPK (b), pACC (c), ACC (d), pS6K (e), S6K (f), pS6 (g), S6 (h), and Actin (i), used as loading control. Size is indicated.
