# Supplementary material for: Mitochondrial metabolism determines chemotherapy sensitivity in colorectal cancer
Source: Nat Metab. 2026 Jul 23;8(8):1751–71. doi: 10.1038/s42255-026-01578-w (PMC13489958; doi:10.1038/s42255-026-01578-w)
Supplement: Supplementary file 2 [file 42255_2026_1578_MOESM17_ESM.pdf]

Blots used for **Figure 8d**

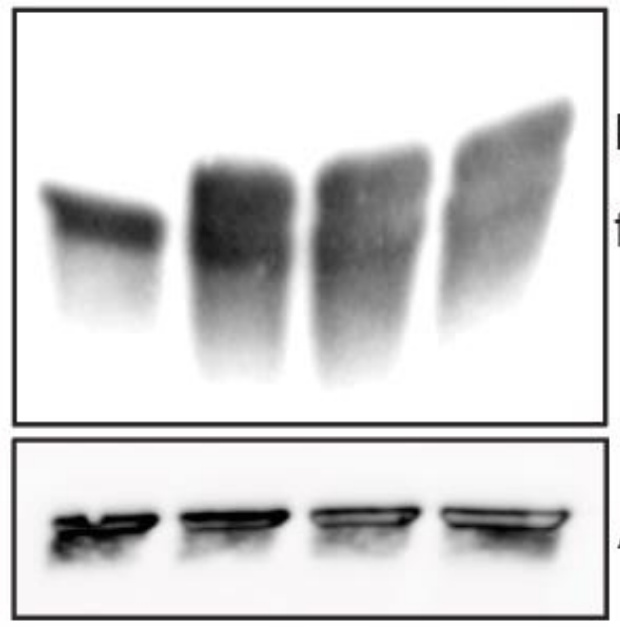

a

0 24 48 72 (hrs 5FU)

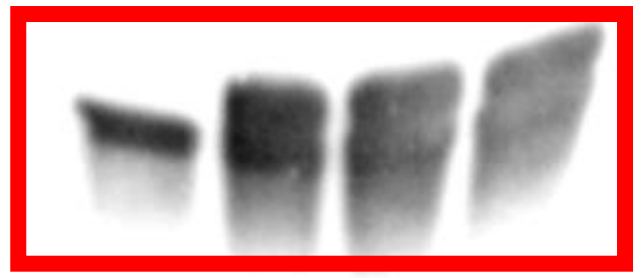

Bound TS

Free TS - 30 KDa

b

0 24 48 72 (hrs 5FU)

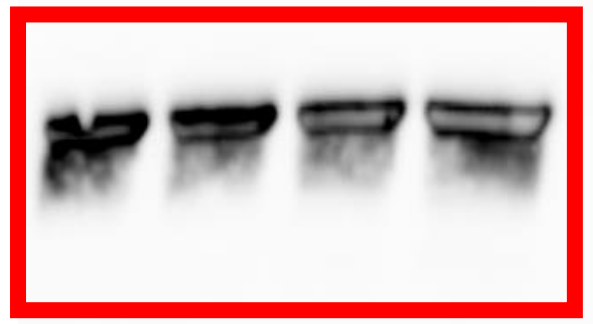

Actin –
45 KDa

c

0 24 48 72 (hrs 5FU)

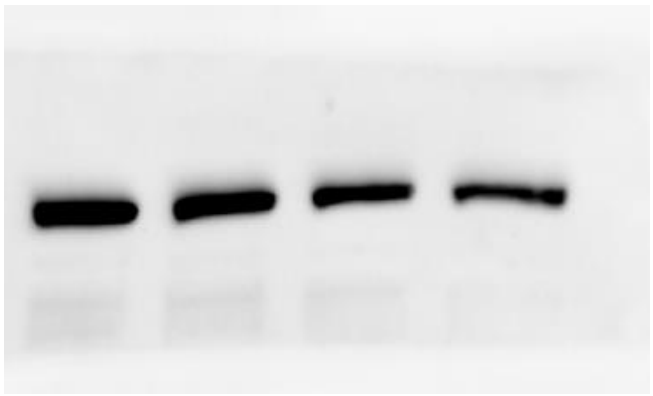

Vinculin –
124 KDa

Blots show western images for cells treated with 5uM 5FU over 72hrs for TS (a) with Actin (b) and Vinculin (c) used as loading. Free TS (lower band) and Bound TS (upper band) is indicated, with bound TS indicating TS inhibition. Size indicated, with red box shows image used for figure.

Blots used for **Figure 8f**

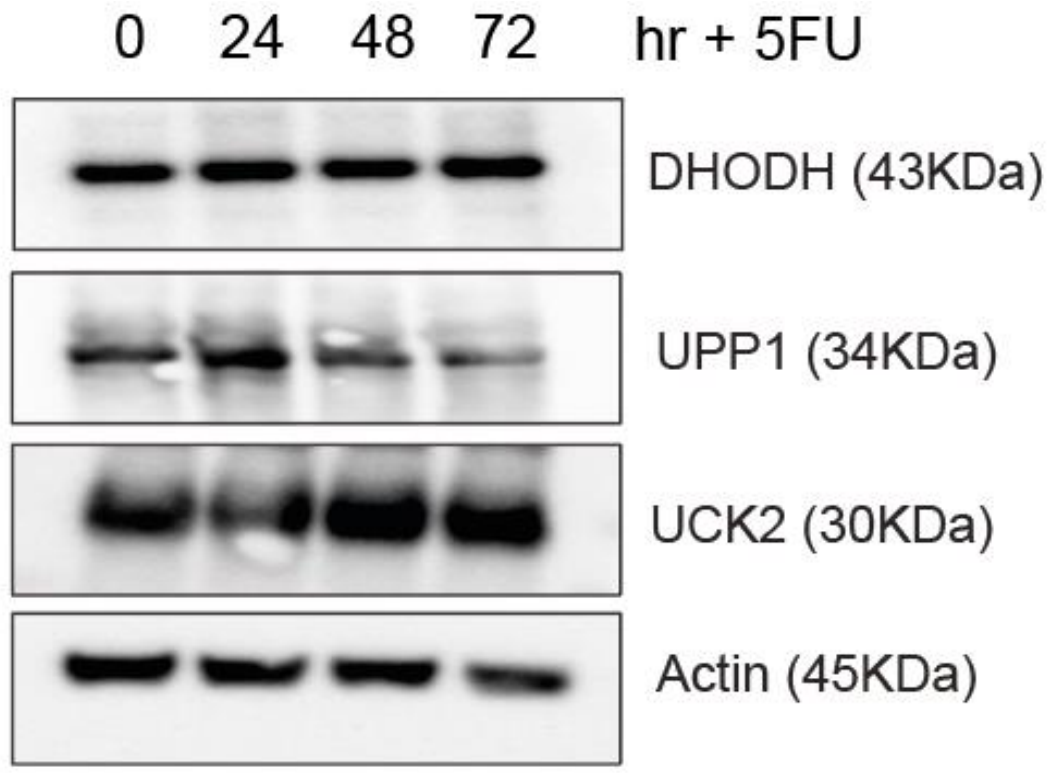

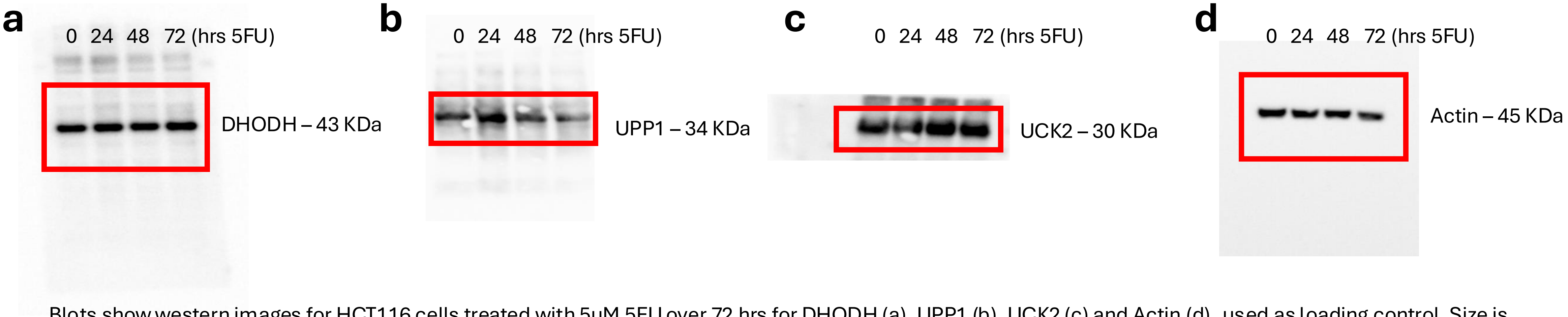

Blots show western images for HCT116 cells treated with 5uM 5FU over 72 hrs for DHODH (a), UPP1 (b), UCK2 (c) and Actin (d), used as loading control. Size is indicated. Red box shows area used for figure panel.

Blots used for **Figure 8l**

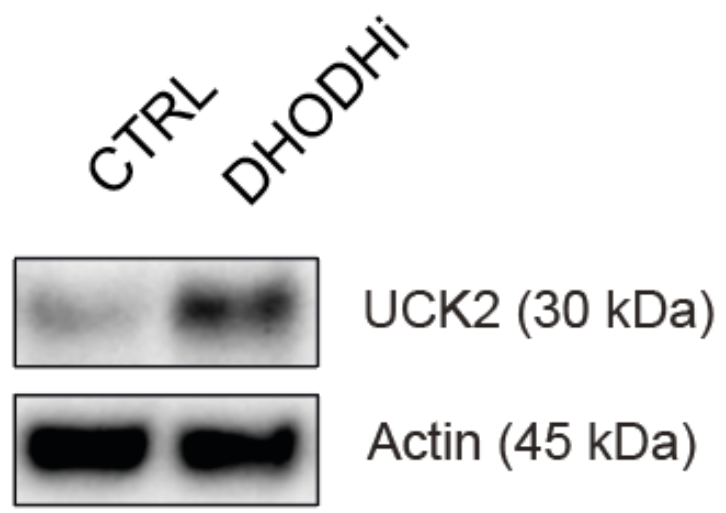

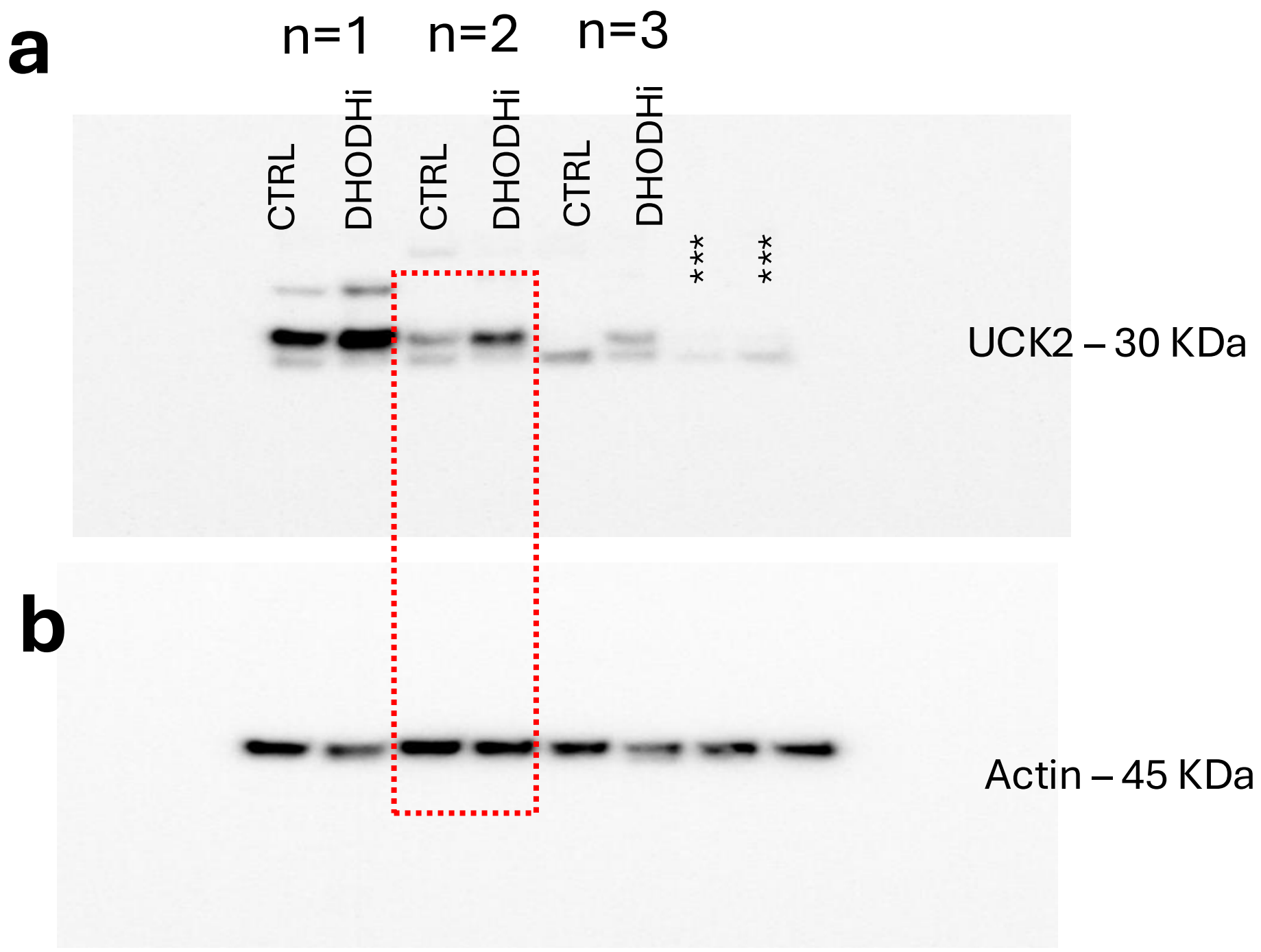

Blots show western images for HCT116 cells treated ± BAY-2402234 (DHODHi) for 72 hrs for UCK2 (a) and Actin (b) used as loading control. Size is indicated. n indicates biological replicate. *** experimental samples not related to these experiments also run on gel. Red box represents areas used for figure panel.

Blots used for **Figure 8j**

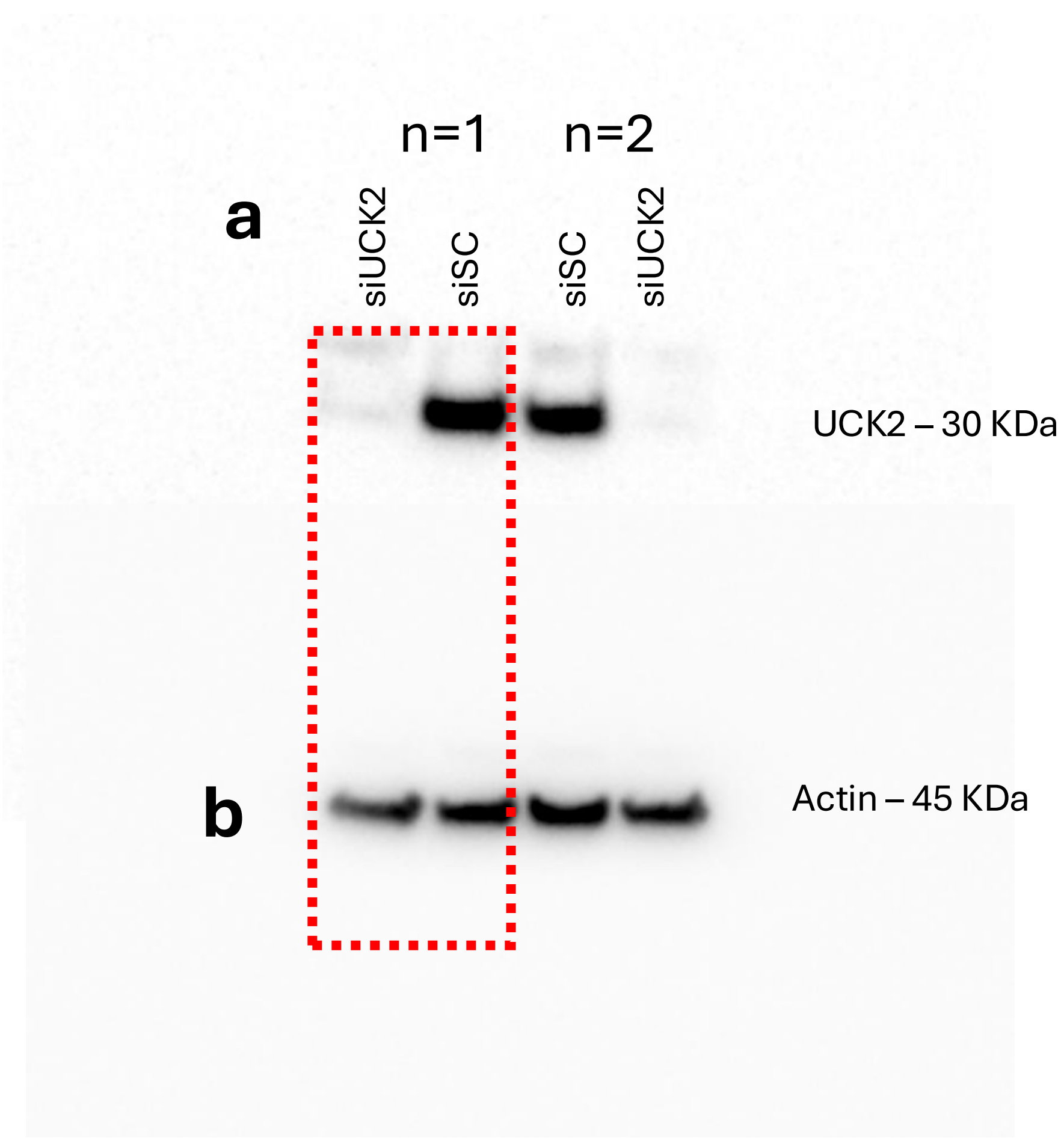

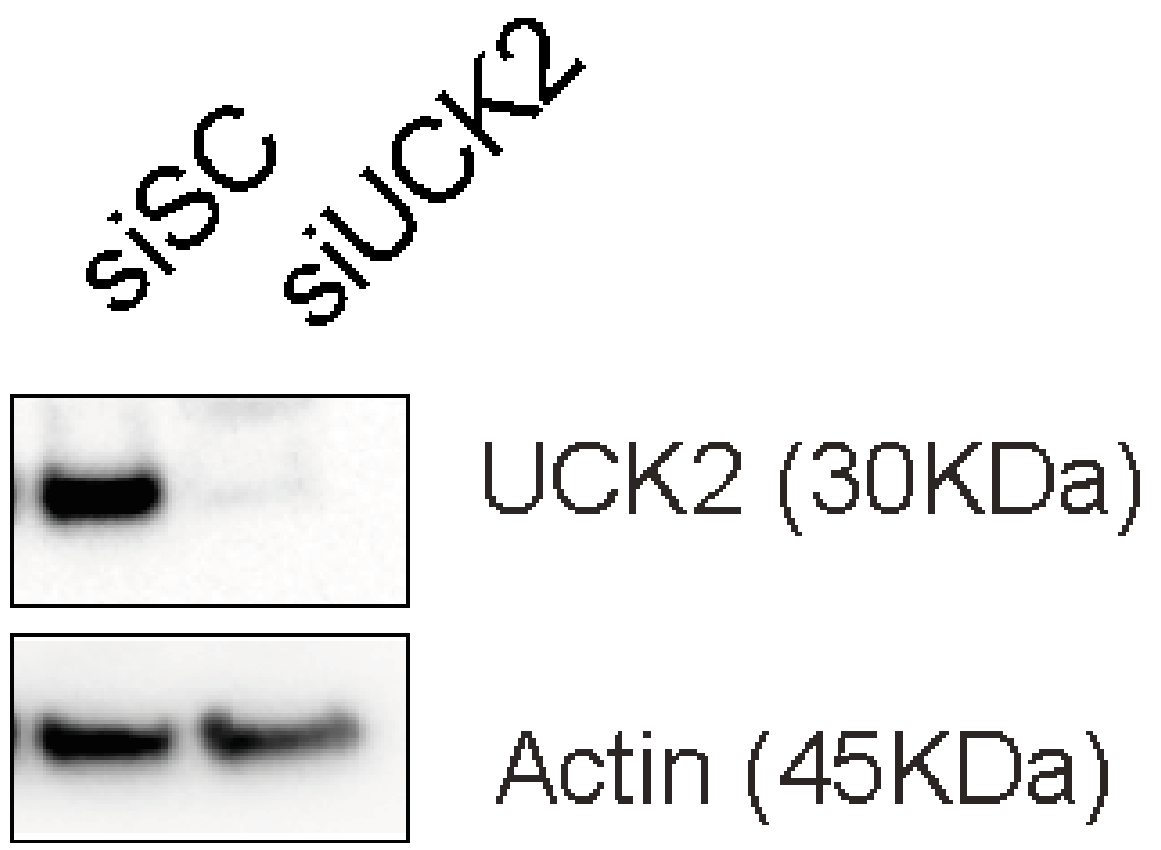

Blots show western images for HCT116 cells treated with siSC or siUCK2 for 72 hrs, blotted for UCK2 (a) and Actin (b) used as loading control. Size is indicated. n indicates biological replicate. Red box shows area used for figure panel.
