# Supplementary material for: Mitochondrial metabolism determines chemotherapy sensitivity in colorectal cancer
Source: Nat Metab. 2026 Jul 23;8(8):1751–71. doi: 10.1038/s42255-026-01578-w (PMC13489958; doi:10.1038/s42255-026-01578-w)
Supplement: Supplementary file 3 [file 42255_2026_1578_MOESM19_ESM.pdf]

Blots used for image panel in **ED Figure 1e**

a

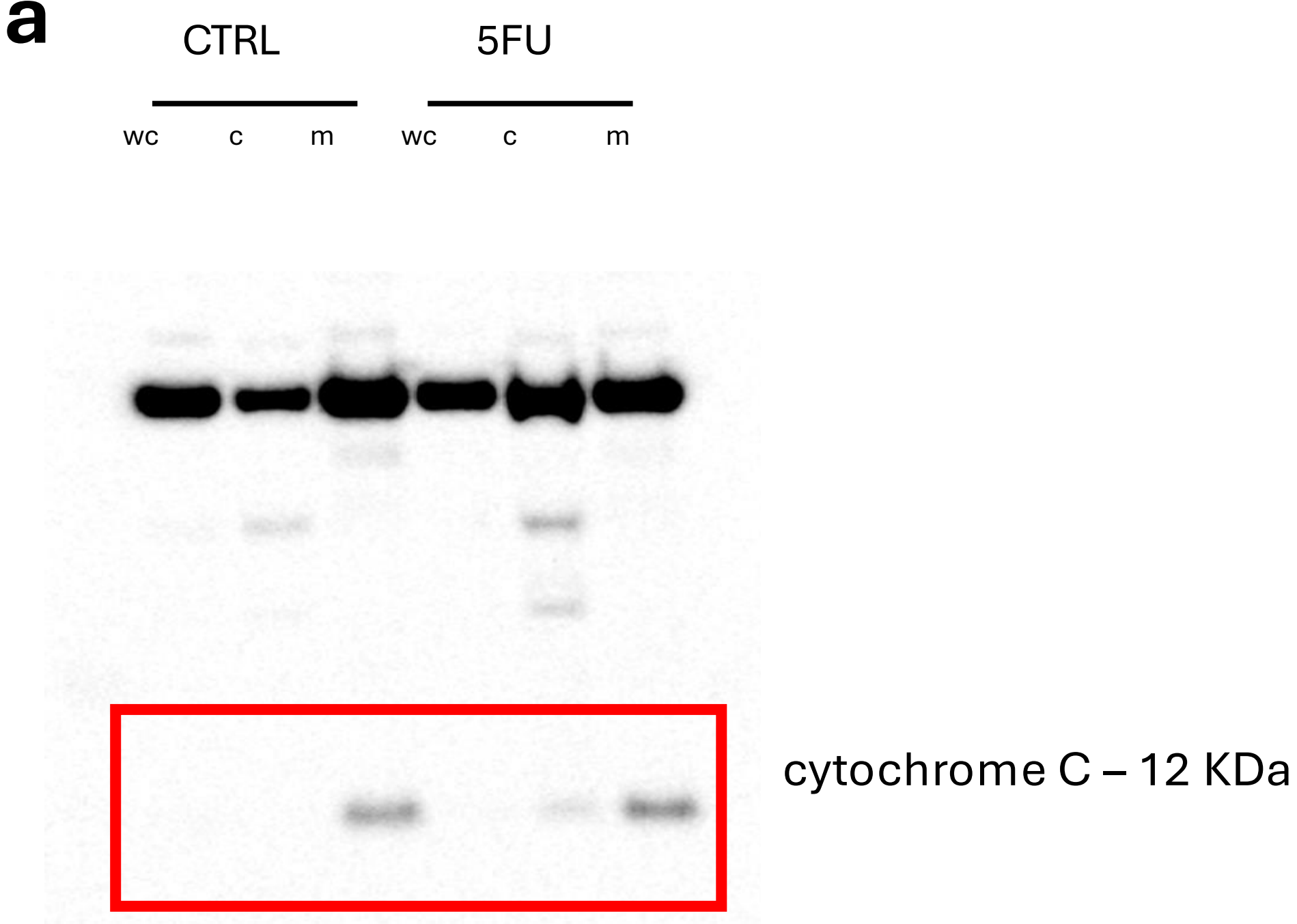

b

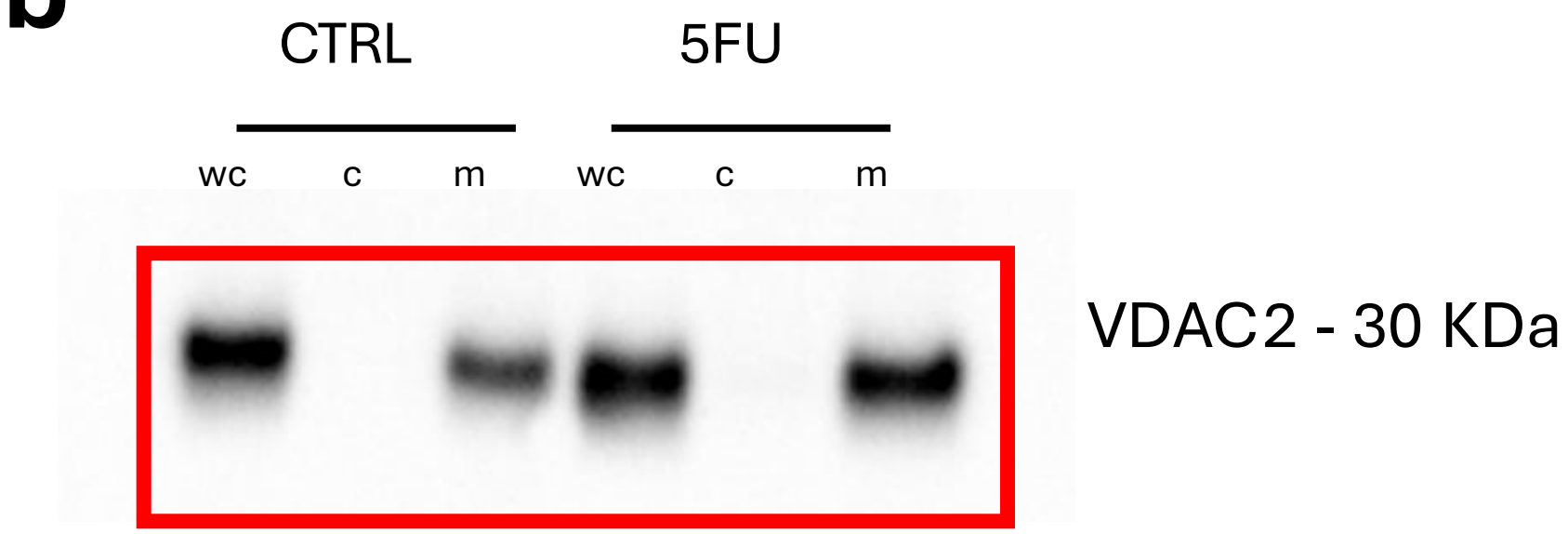

c

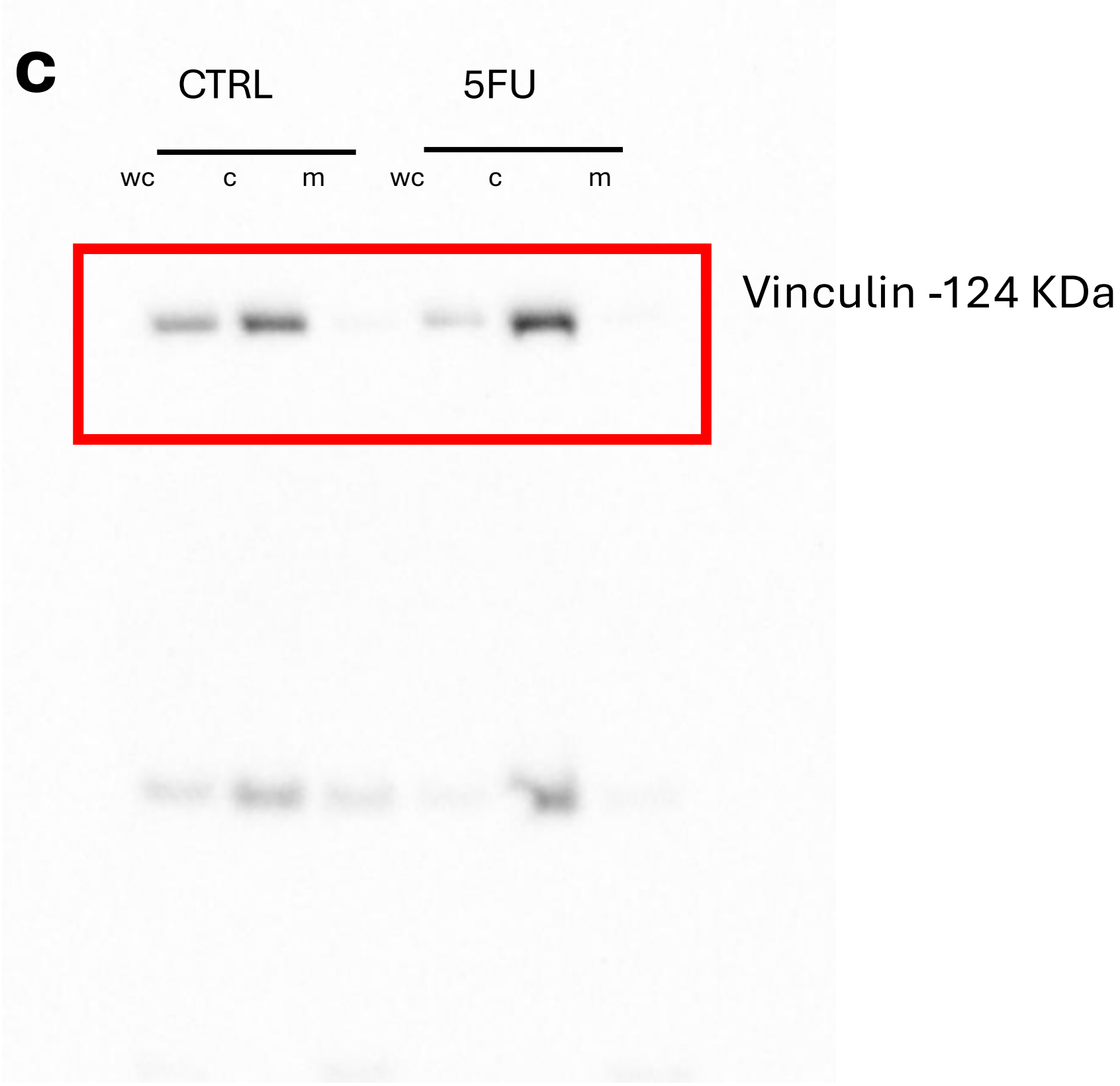

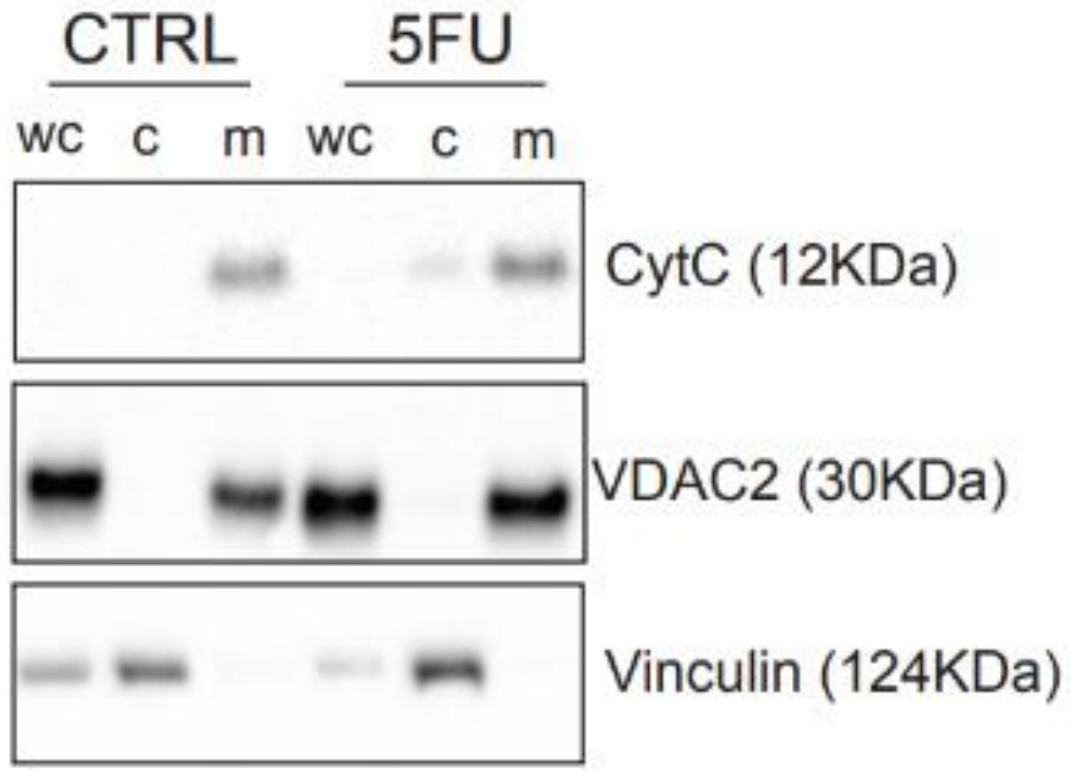

Blots show western images for cells treated ± 5FU for 72 hrs before mitochondrial fractionation according to kit (ab65311) as indicated for cytochrome c (a), VDAC2 (b) and Vinculin (c). Size is indicated. c= cytosolic, m=mitochondrial, wc=whole cell lysate. Red line indicates region shown.
