# Supplementary material for: Mitochondrial metabolism determines chemotherapy sensitivity in colorectal cancer
Source: Nat Metab. 2026 Jul 23;8(8):1751–71. doi: 10.1038/s42255-026-01578-w (PMC13489958; doi:10.1038/s42255-026-01578-w)
Supplement: Supplementary file 4 [file 42255_2026_1578_MOESM1_ESM.pdf]

nature metabolism

Supplementary information https://doi.org/10.1038/s42255-026-01578-w

Mitochondrial metabolism determines chemotherapy sensitivity in colorectal cancer

In the format provided by the authors and unedited

Figure 1c

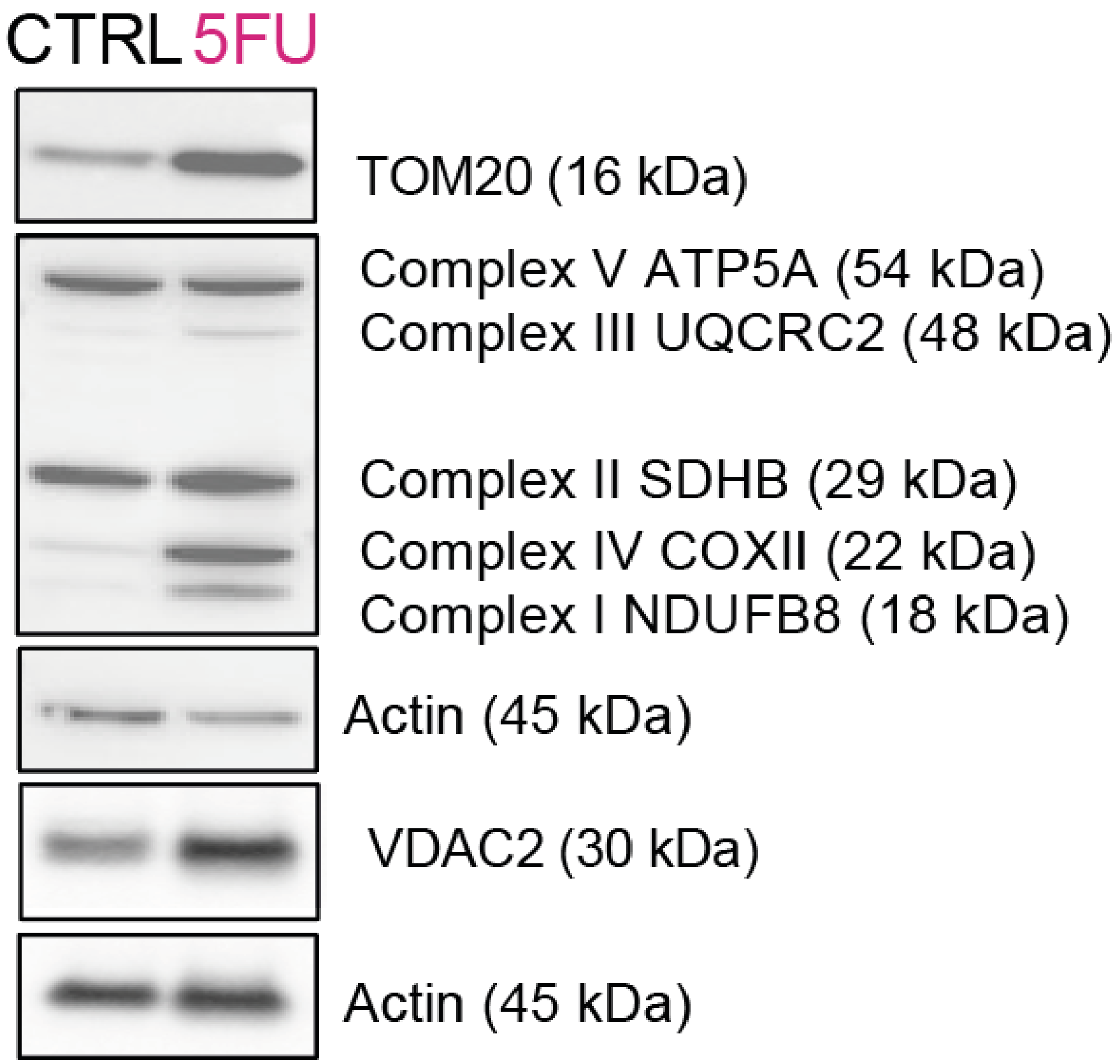

WB Observation 1

5FU treatment increases mitochondrial protein abundance

Blots used for image panel in **Figure 1c** – HCT116 cells

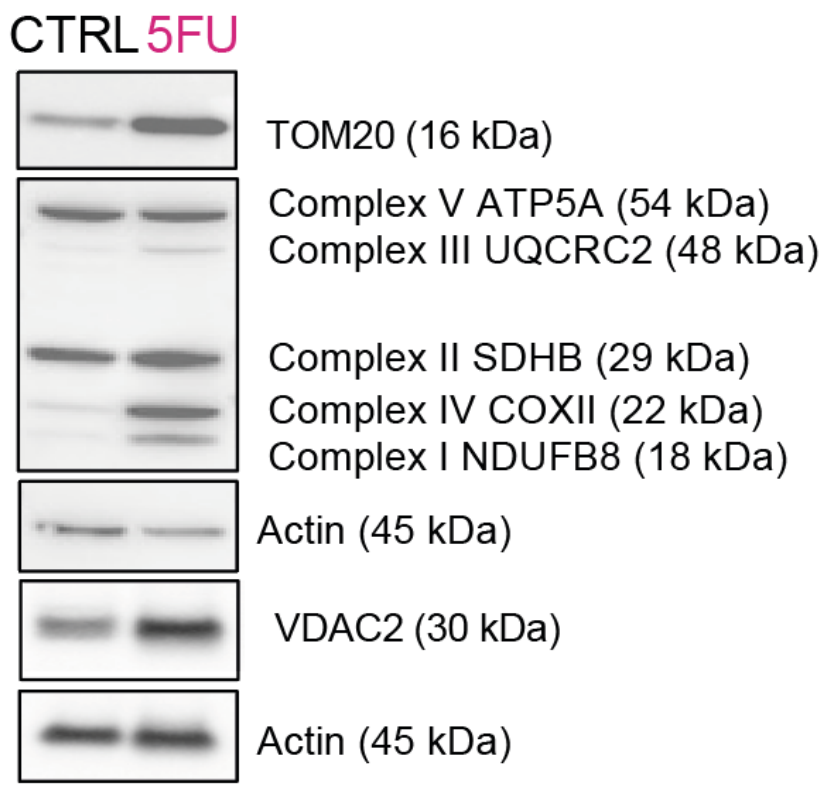

a

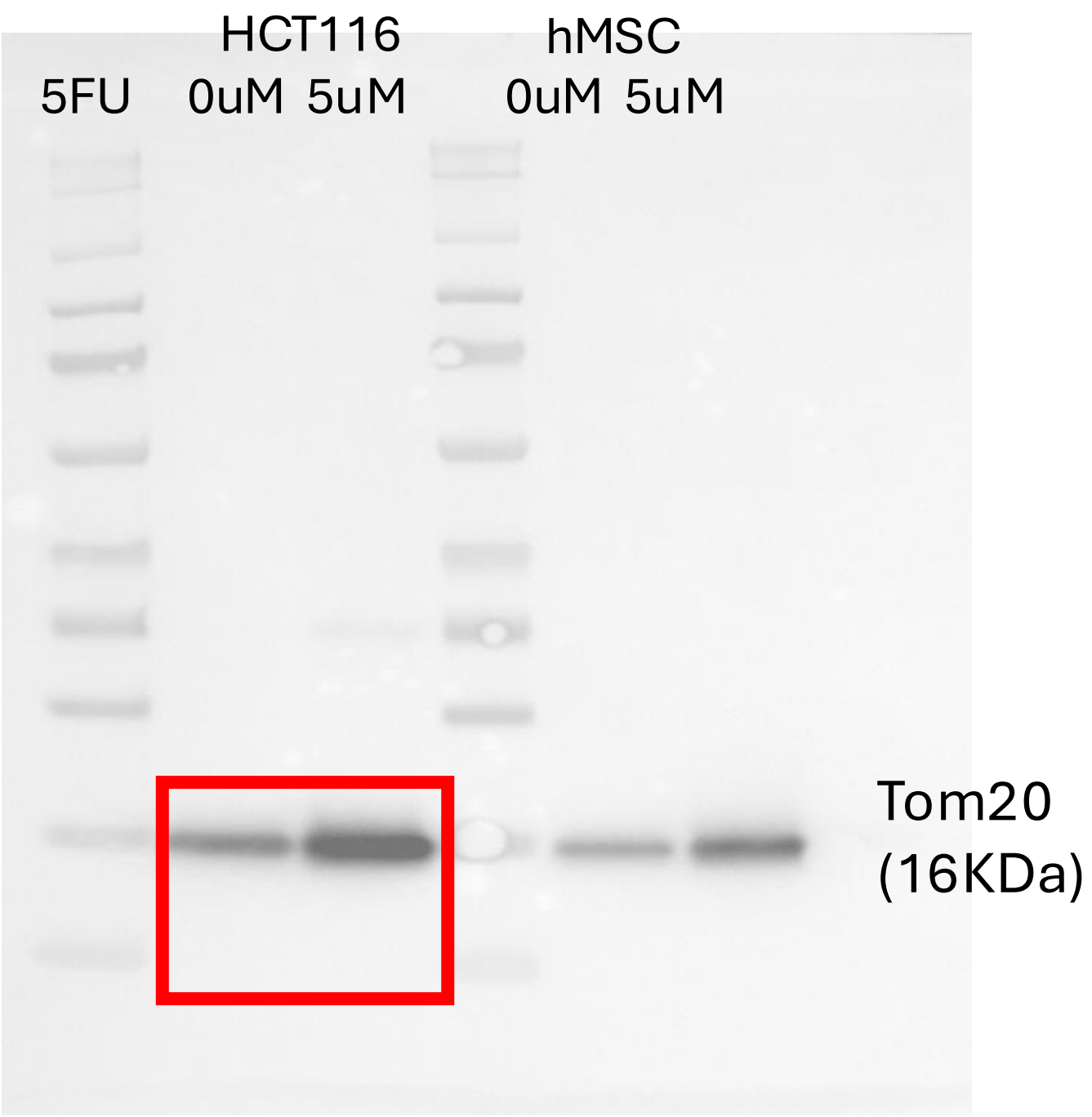

b

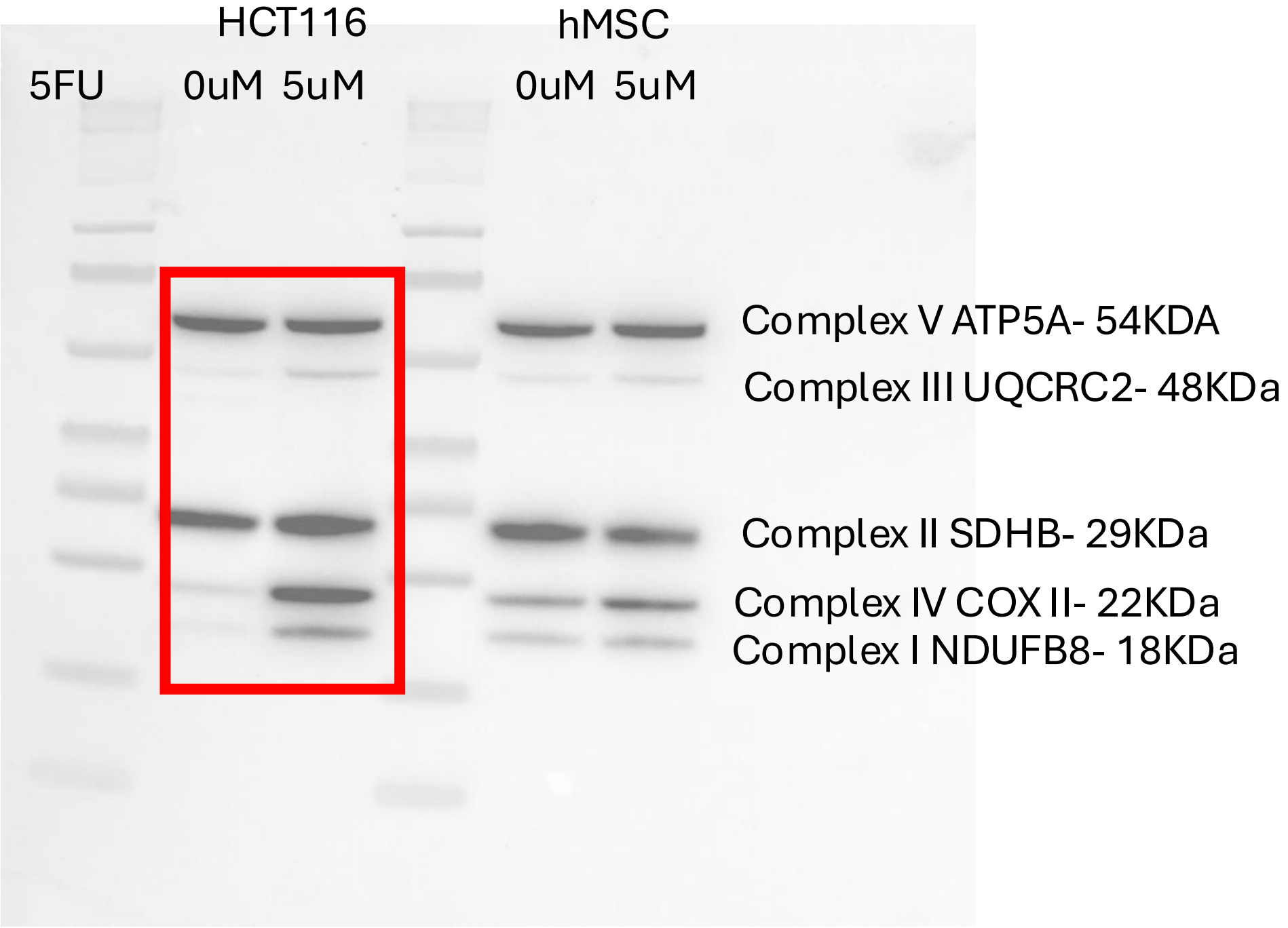

c

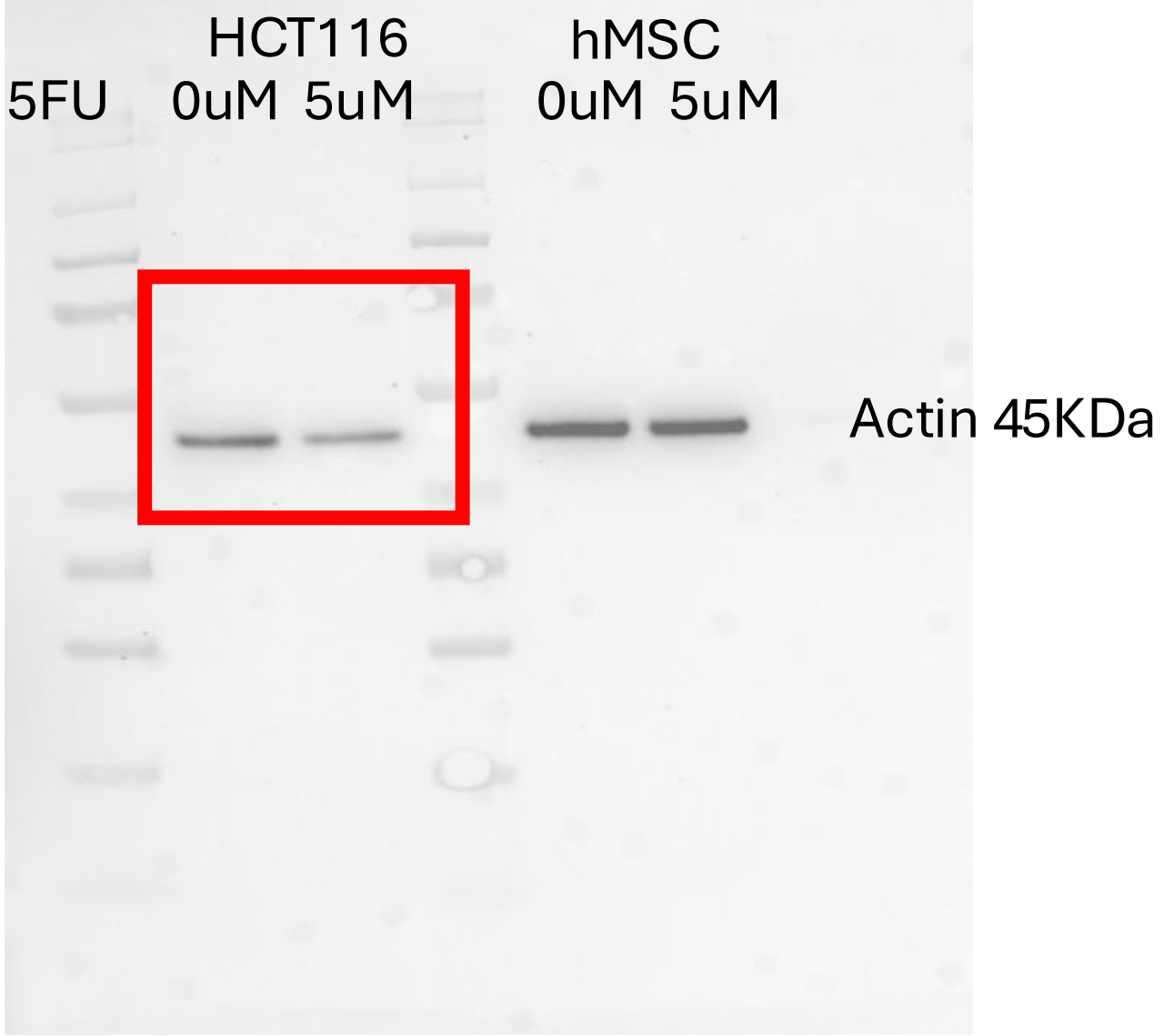

Blots show Western images for HCT116 cells and hMSC cells treated ± 5FU (5 uM, 72 hrs) for Tom20 (a), electron transport chain antibody panel (b) and Actin used as loading control (c). Size is indicated, and red boxes denote area used for paper figure.

Blots used for image panel in **Figure 1c**

NB. VDAC2 was run separately in response to reviewers' comments

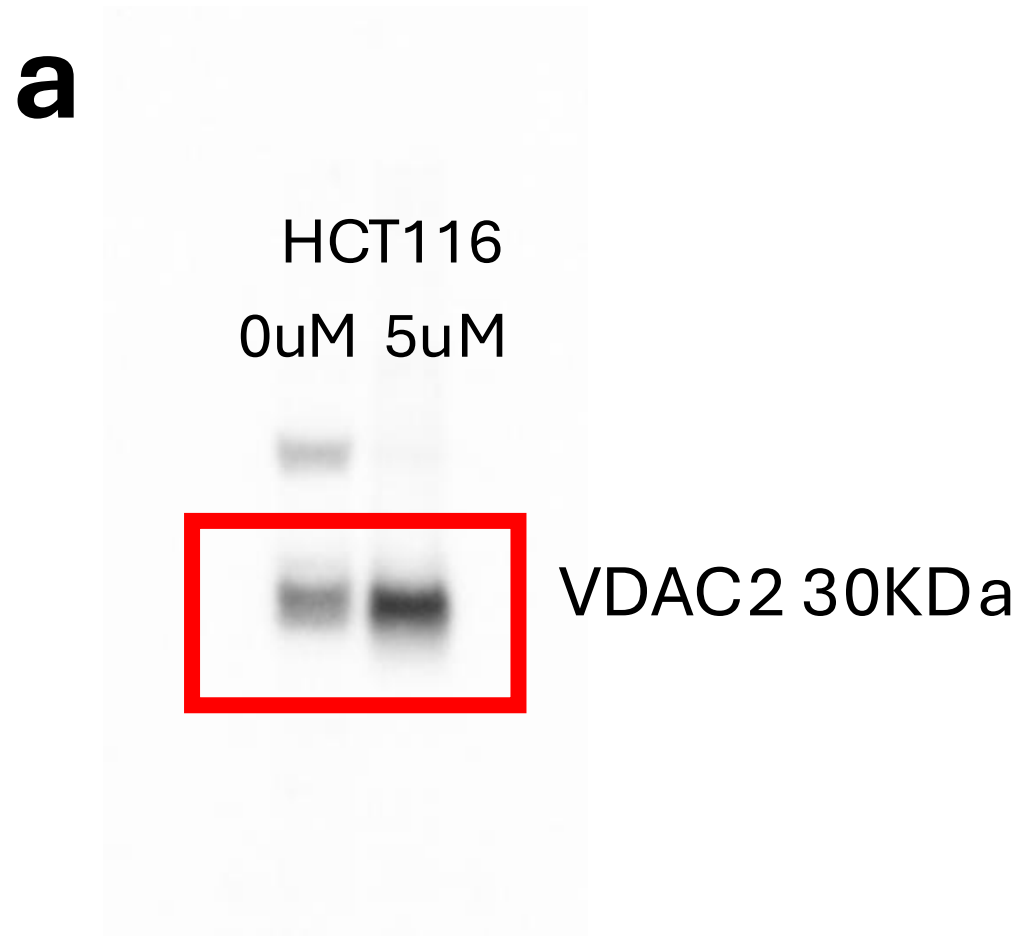

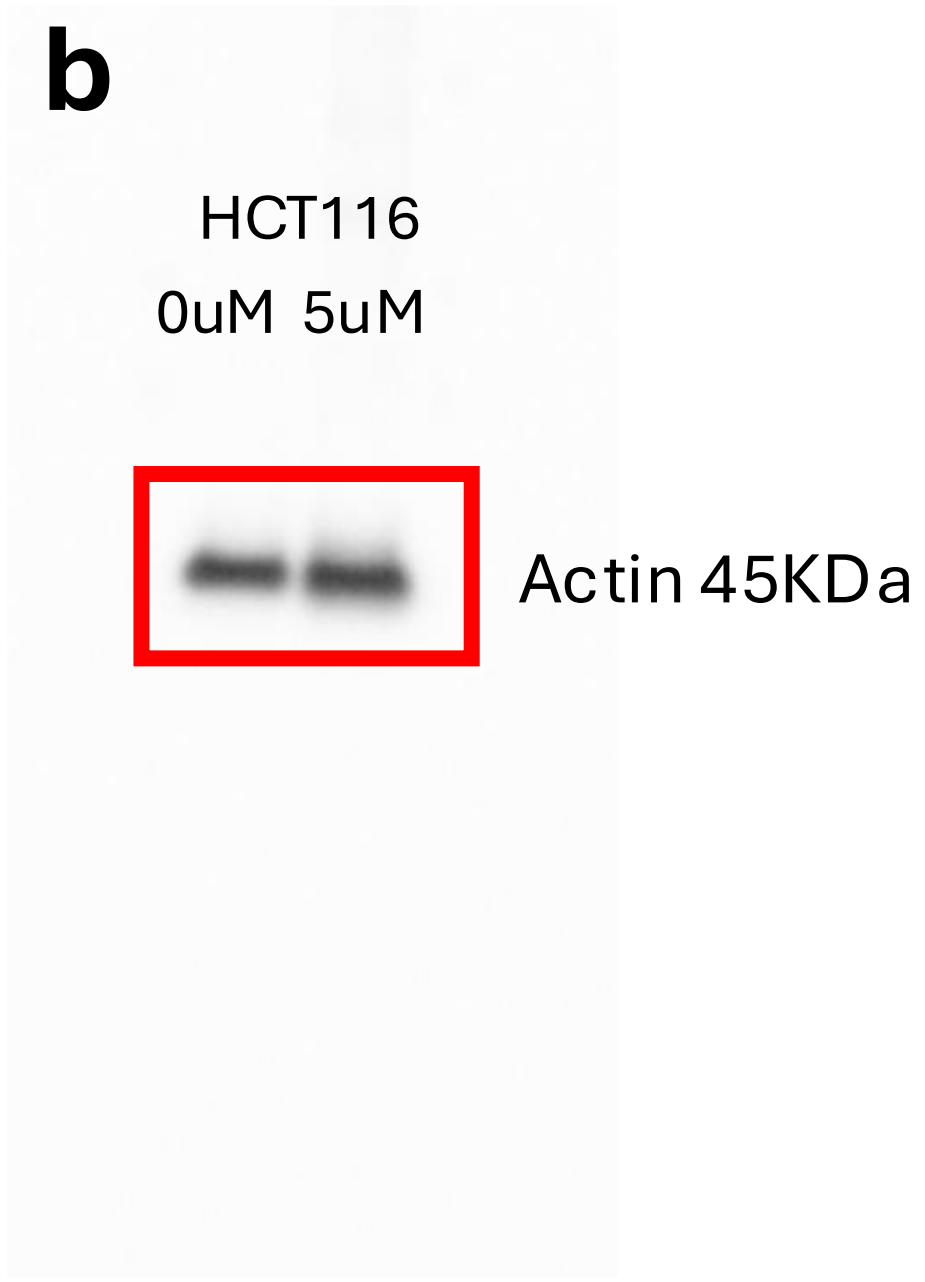

Blots show Western images for HCT116 cells treated ± 5FU (5 uM, 72 hrs) for VDAC2 (a) and Actin used as loading control (b). Size is indicated, and red boxes denote area used for paper figure.

Independent biological repeat experiments n=2 and n=3 for data in **Figure 1c**; blots show ETC, Tom20 and actin

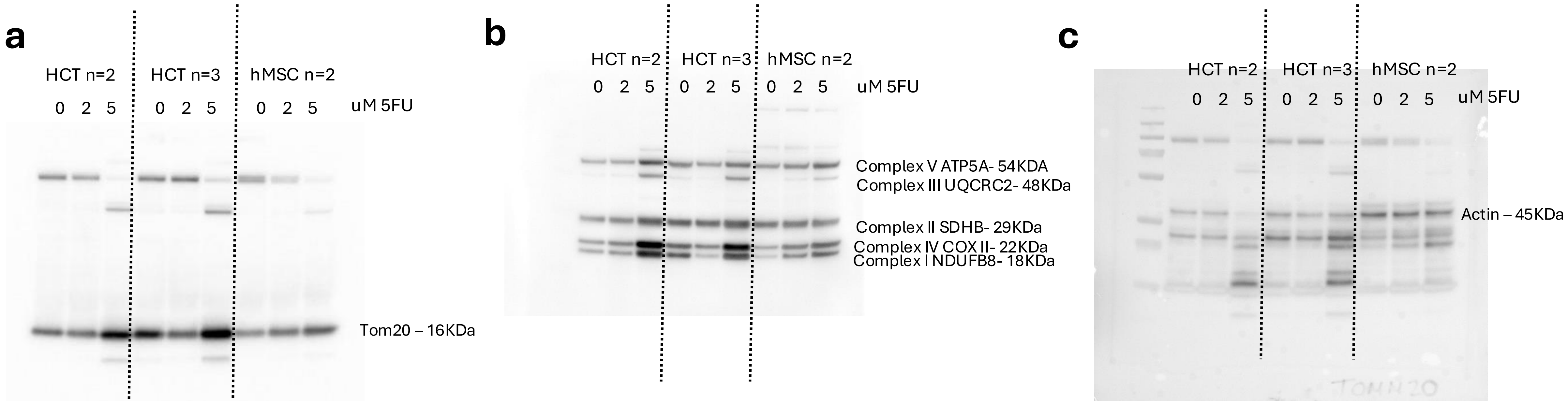

Blots show independent Western images for HCT116 cells and hMSC cells treated ± 5FU (0, 2, 5 uM, 72 hrs) for Tom20 (a), electron transport chain antibody panel (b) and Actin used as loading control (c). Size is indicated. n indicates biological replicate.

Two independent biological repeat experiments (n=2 and 3) for VDAC2 expression using HCT116 timecourse and ± 5FU at 72hrs, run later in response to reviewers' request; blots show actin and VDAC2 over timecourse with 5uM 5FU, and VDAC2 and actin ± 5uM 5FU at 72hrs

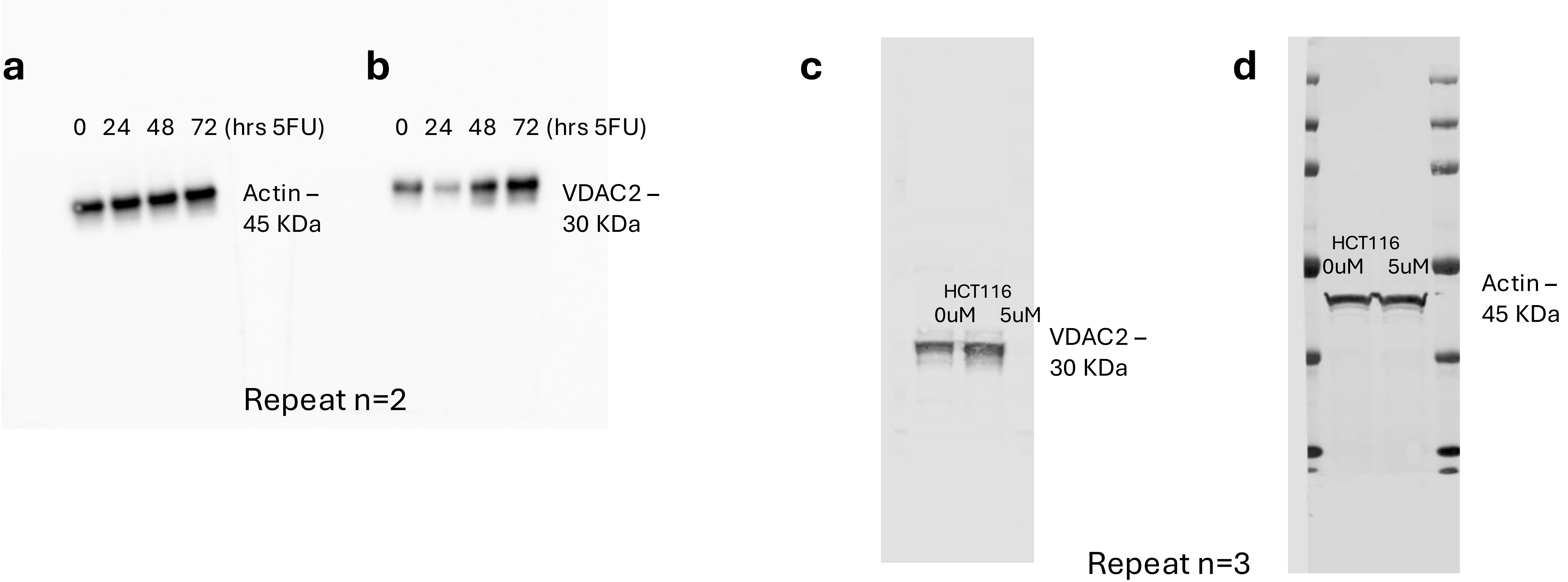

Blots show independent Western images for HCT116 cells treated ± 5uM 5FU at 0, 24, 48, 72 hrs (a-b) or ± 5uM 5FU for 72 hrs (c-d), for VDAC2 (b & c) and Actin used as loading control (a & d). Size is indicated. n indicates biological replicate.

Biological repeat experiments (n=4 and n=5) using two independent HCT116 lines; blots show ETC, TOM20, VDAC2, actin, vinculin and ponceau of membranes

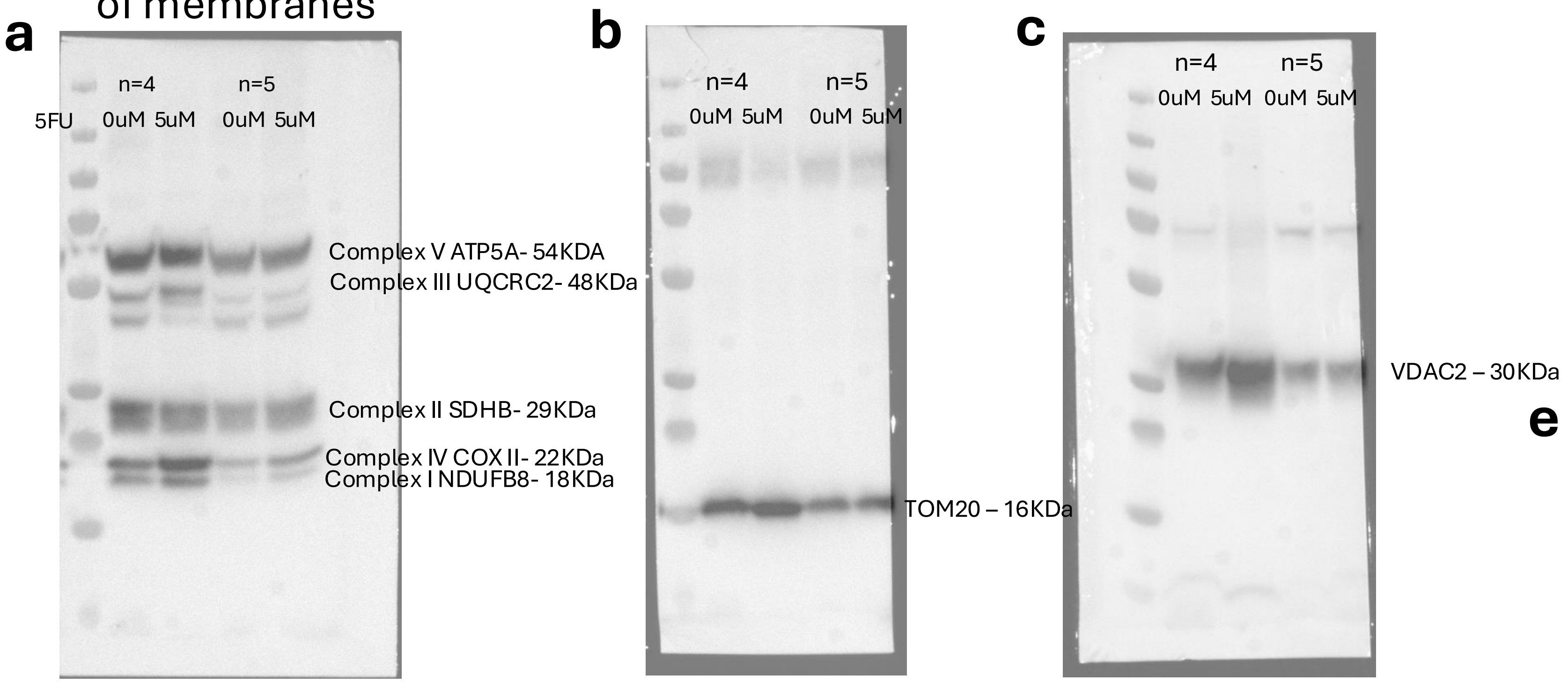

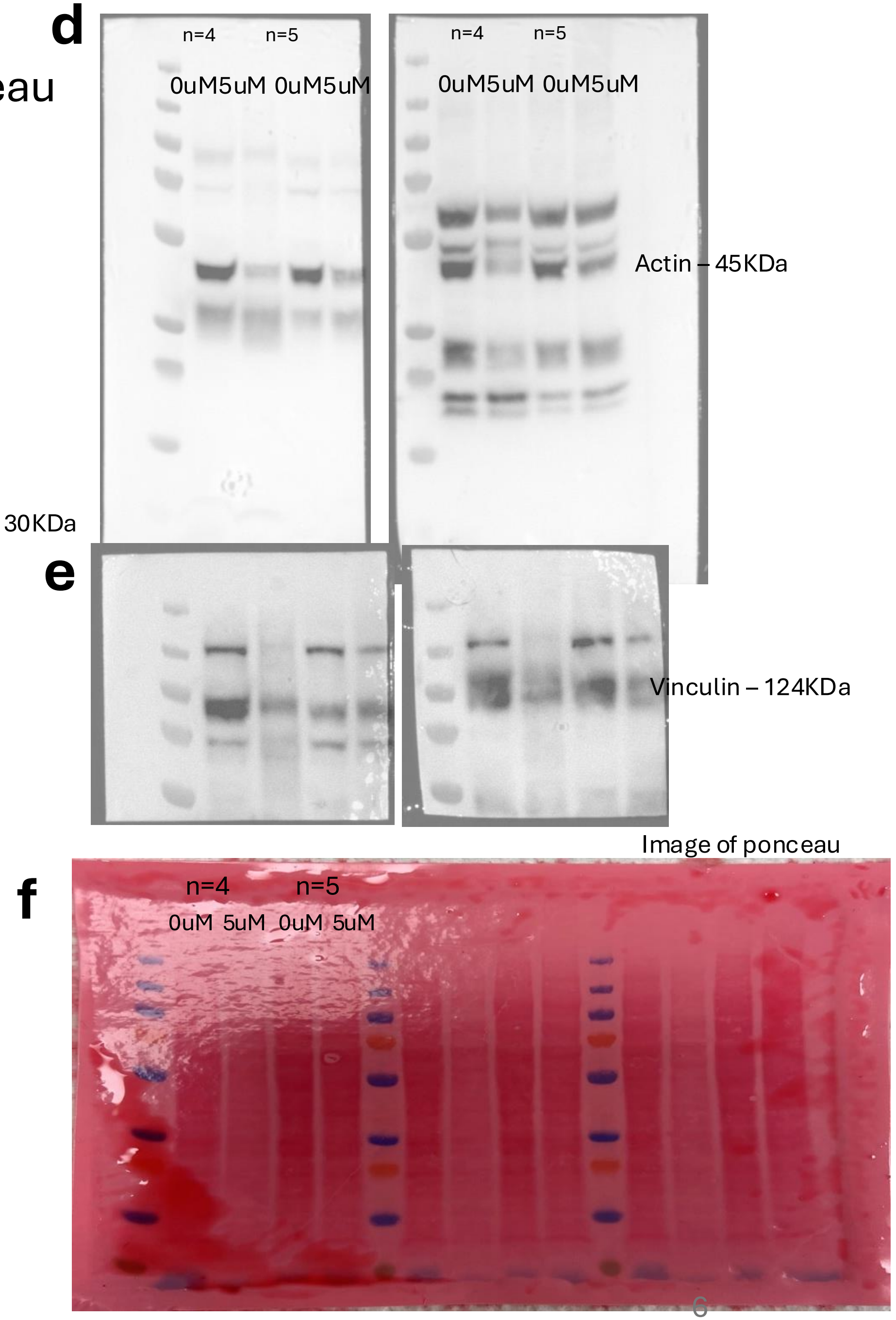

Blots show independent Western images for HCT116 cells treated ± 5uM 5FU for 72 hrs for electron transport chain panel (a), TOM20 (b), VDAC2 (c), Actin used as loading control (d), Vinculin used as a loading control (e) and ponceau image of blots (f). Size is indicated. n indicates biological replicate.

Densitometry of all HCT116 data presented for mitochondrial mass

1c HCTs														
Repeat 1	CTRL	5FU	Repeat 2			Repeat 3			Repeat 4			Repeat 5		
V	4301.406	4410.355	V	658.042	2633.891	V	1756.355	2057.355	V	9735.79	9769.205	V	7276.619	7060.619
III	220.849	712.799	III	100	786.556	III	159.435	1040.92	III	3484.113	4291.991	III	1754.335	1591.92
II	3929.527	5354.012	II	1159.406	3301.719	II	2768.891	3602.841	II	2814.527	1882.042	II	2214.941	2342.527
IV	657.335	4761.648	IV	940.456	4746.698	IV	1315.577	5129.406	IV	10964.882	10010.054	IV	7233.64	7985.64
I	274.971	1116.577	I	691.678	4427.355	I	1536.527	4583.82	I	7819.912	10809.497	I	4398.962	4761.912
TOM20	4868.276	9057.004	TOM20	2268.648	5017.962	TOM20	3251.305	7633.276	TOM20	7801.397	9088.397	TOM20	6117.983	6840.276
ACTIN	3461.426	2167.941	ACTIN	2121.355	1943.527	ACTIN	6820.77	5212.941	ACTIN	6729.82	3019.698	ACTIN	5620.527	3571.82
VDAC2	3917.719	7197.255	VDAC2	3474.77	6915.648	VDAC2	14257.64	19698.196	VDAC2	8669.477	10967.234	VDAC2	4310.77	4978.355
ACTIN	3874.941	4628.648	ACTIN	6669.619	7303.619	ACTIN	20539.882	19540.368	ACTIN	5497.749	3825.456	ACTIN	4385.456	3942.163
Normalised to Actin														
	CTRL	5FU												
V	1.24266877	2.03435195	V	0.31019891	1.35521194	V	0.25750099	0.39466301	V	1.44666425	3.23515961	V	1.29465066	1.97675667
III	0.0638029	0.32879077	III	0.04713968	0.40470547	III	0.02337493	0.19967999	III	0.51771266	1.42133121	III	0.31212998	0.44568875
II	1.13523357	2.46962994	II	0.5465403	1.69882847	II	0.40594991	0.69113405	II	0.41821728	0.62325504	II	0.39408066	0.65583568
IV	0.18990295	2.19639188	IV	0.44332797	2.44231132	IV	0.19287808	0.98397546	IV	1.62929796	3.31491891	IV	1.28700387	2.23573416
I	0.07943865	0.51504031	I	0.32605481	2.27800025	I	0.22527178	0.87931553	I	1.16197937	3.57966161	I	0.78266006	1.33318924
TOM20	1.40643654	4.17769856	TOM20	1.06943345	2.58188438	TOM20	0.47667712	1.46429357	TOM20	1.15922818	3.00970395	TOM20	1.088507	1.91506739
VDAC2	1.01103965	1.55493678	VDAC2	0.52098478	0.94687962	VDAC2	0.6941442	1.00807702	VDAC2	1.57691393	2.86690894	VDAC2	0.98296962	1.26284859
Fold Change over CTRL														
V		1.63708302			4.36884823			1.53266603			2.23628918			1.52686491
III		5.15322657			8.58523964			8.54248613			2.74540555			1.42789471
II		2.17543773			3.10833157			1.70251063			1.49026612			1.66421686
IV		11.5658651			5.50903959			5.10154121			2.03456887			1.73716196
I		6.48349794			6.98655621			3.90335413			3.08065849			1.70340779
TOM20		2.97041384			2.41425436			3.07187719			2.59629985			1.75935238
VDAC2		1.53795825			1.81748039			1.4522588			1.81805036			1.284728

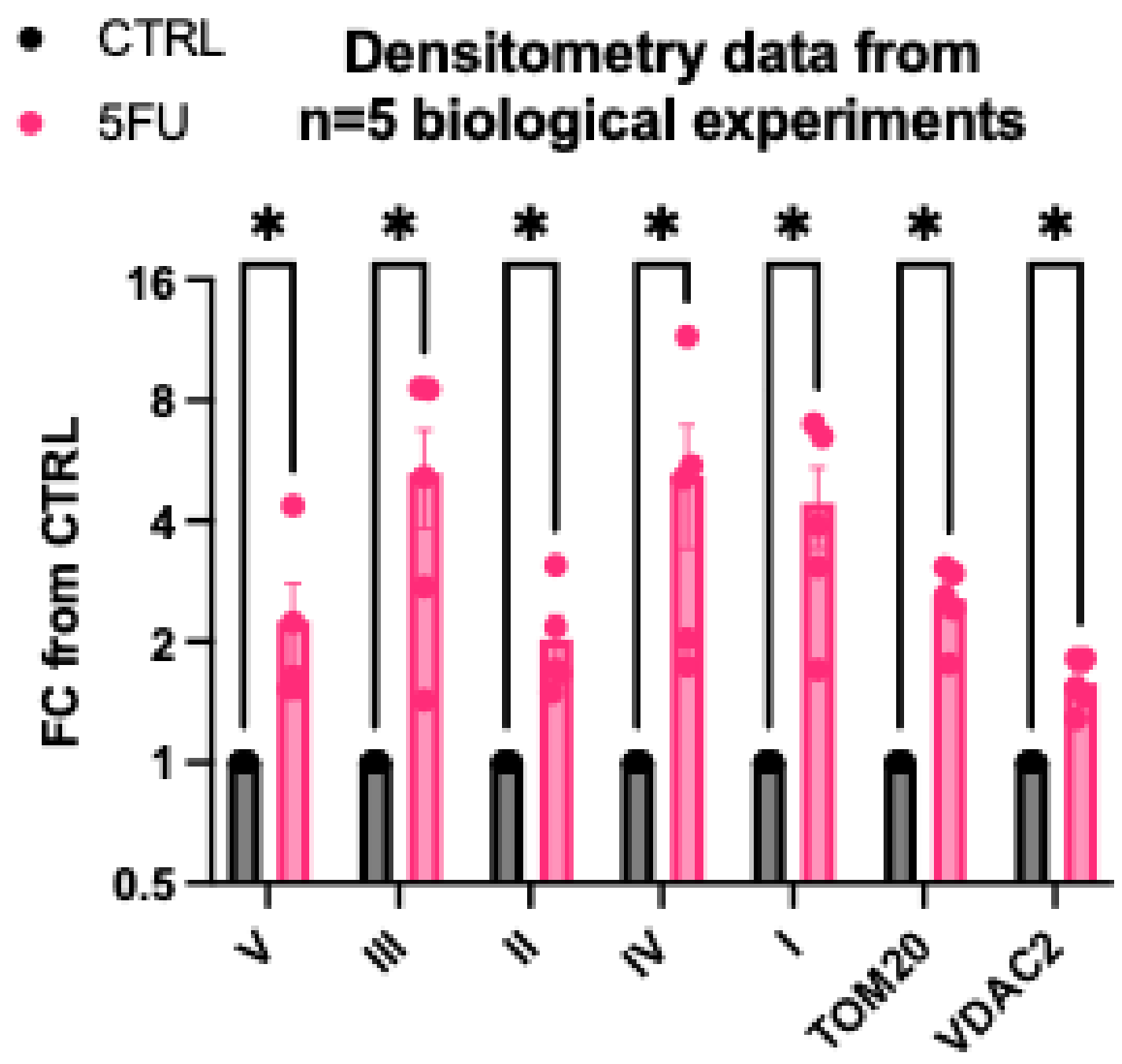

Table shows densitometry values for blots n=1-5, CTRL vs 5FU at 72hrs, with raw values then normalised to Actin and expressed as fold change over CTRL. Graph shows expression fold changes as a function of CTRL (FC from CTRL) for each replicate ± sem, with statistical testing by t-test indicated.

Additional models:
SW480 (EDFig2c)
AKP murine CRC 3D organoid line (EDFig2f)
AKP murine CRC 2D cell line (EDFig2h)

Extended Data Figure 2c: SW480 data; Biological repeat experiments (n=1-3) blots show ETC, Tom20, vdac2 and actin

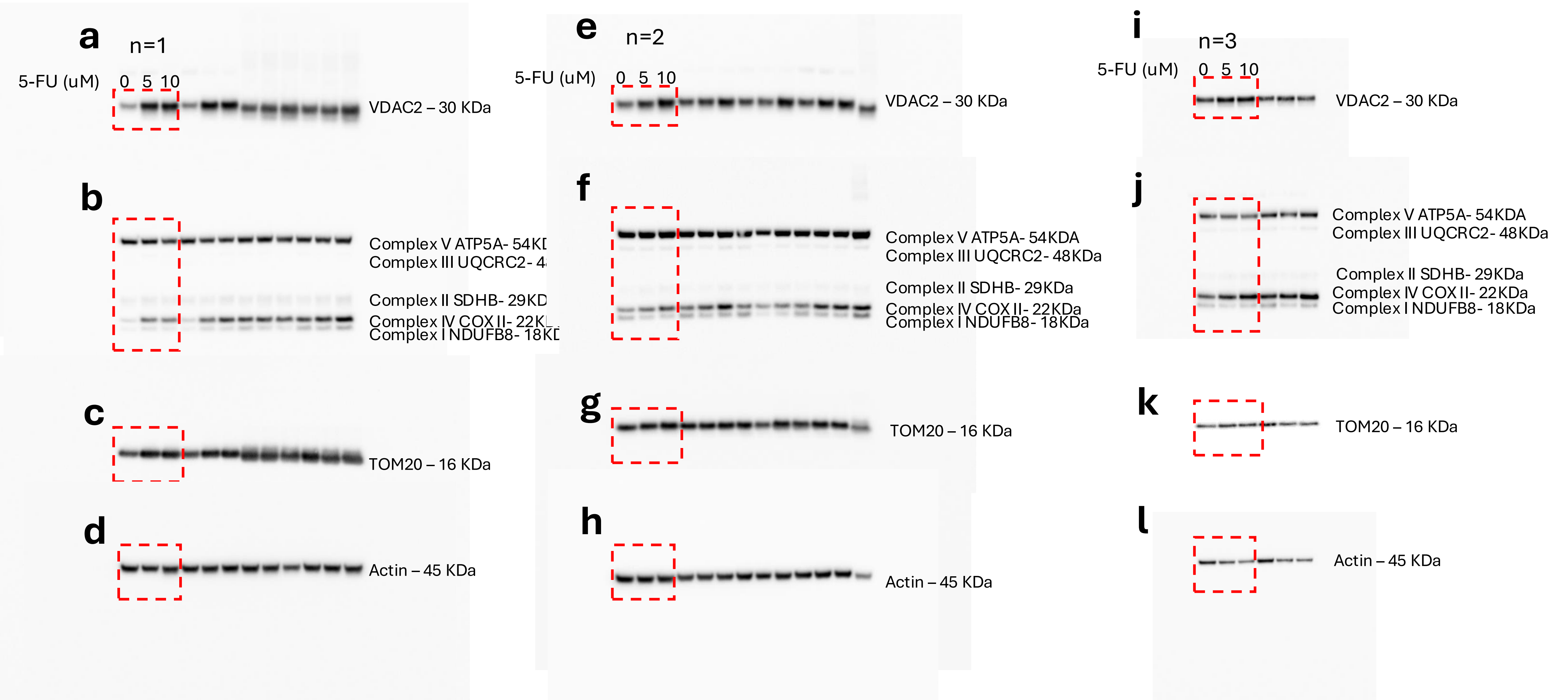

Blots show independent western images for SW480 cells treated ± 5 or 10uM 5FU for 72 hrs for VDAC2 (a,e,i), electron transport chain panel (b,f,j), TOM20 (c,g,k), Actin (d,h,l) used as a loading control. Size is indicated. n indicates biological replicate. Data from n=1 used in experiment.

AKP organoids 3D; *data shows three independent biological replicates run,*
CTRL (-) and 5FU (+)

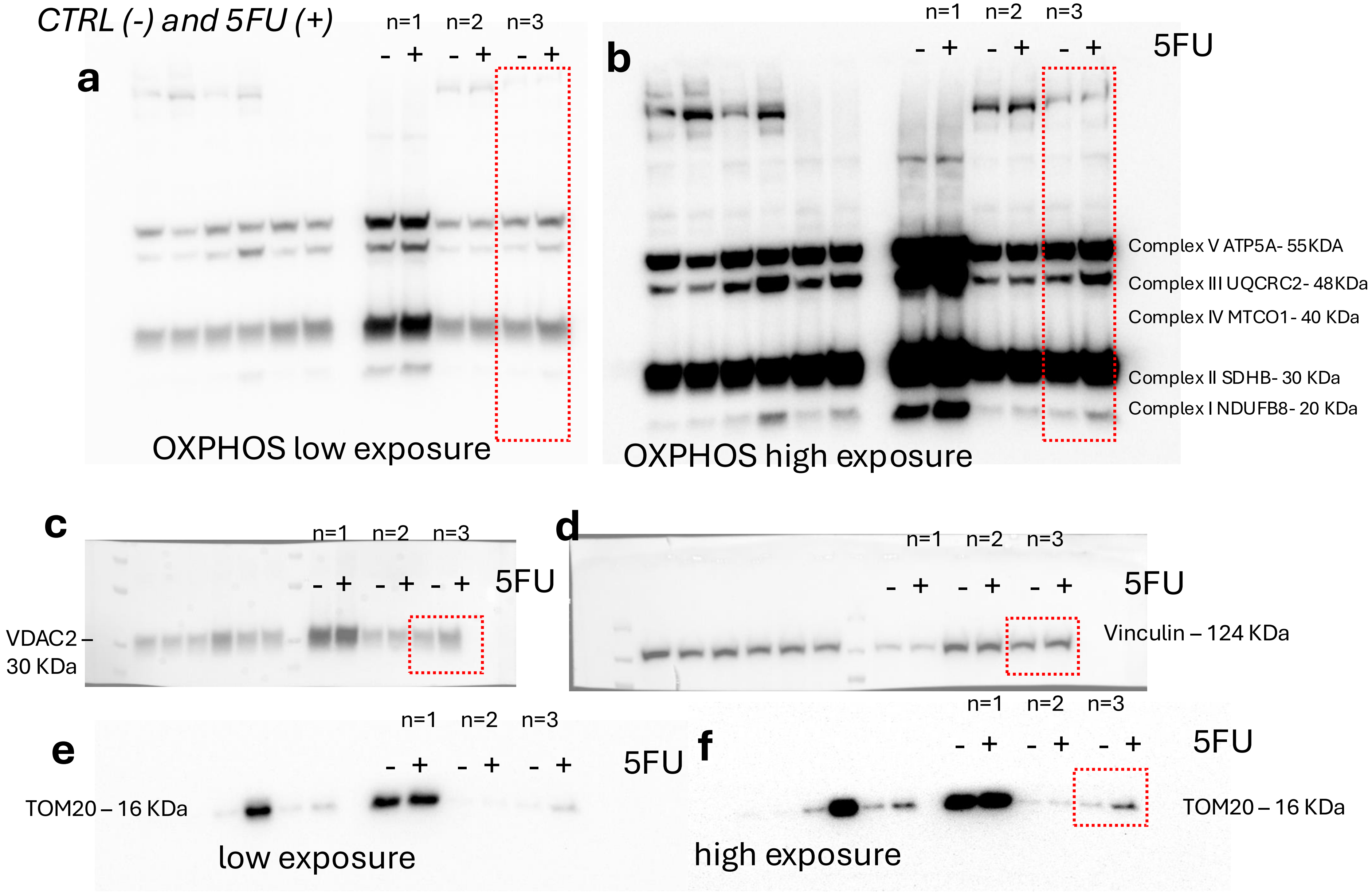

Ext. Data Fig 2f

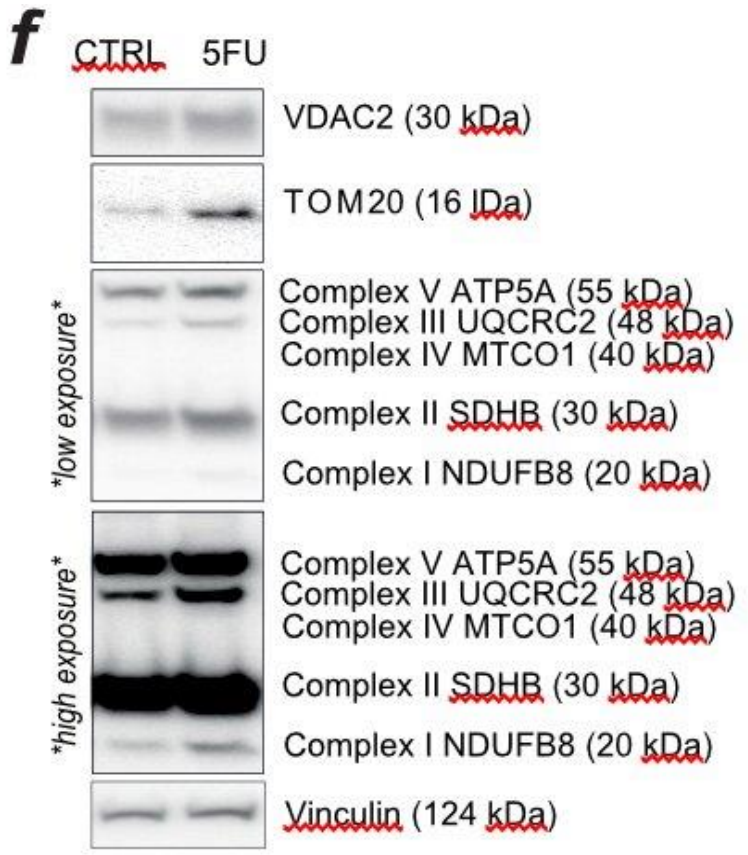

Blots show independent western images for AKP organoids in 3D culture treated ± 5FU for 72 hrs for electron transport chain panel (a, low exposure and b, high exposure), VDAC2 (c), Vinculin (d) used as loading control and TOM20 (e, low exposure and f, high exposure). Size is indicated. n indicates biological replicate.

AKP cell line 2D; *data shows three independent biological replicates run; 0, 2 and 5uM 5FU at 72 hrs*

Ext. Data Fig 2h

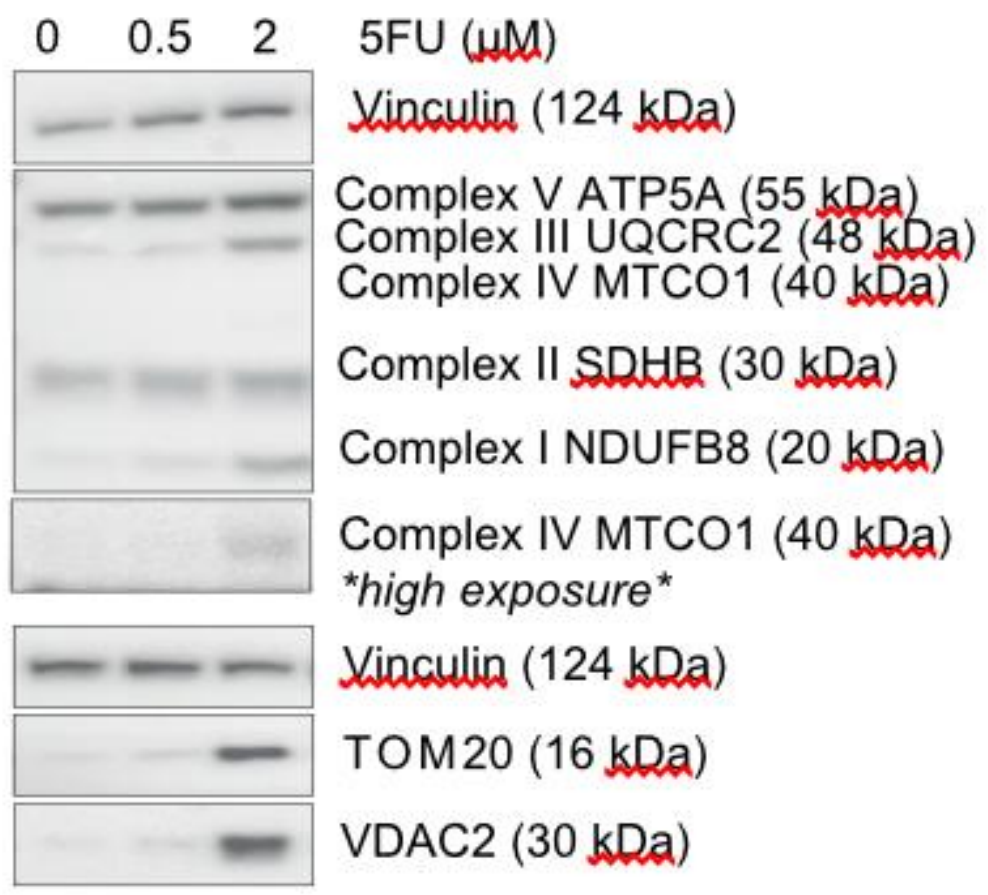

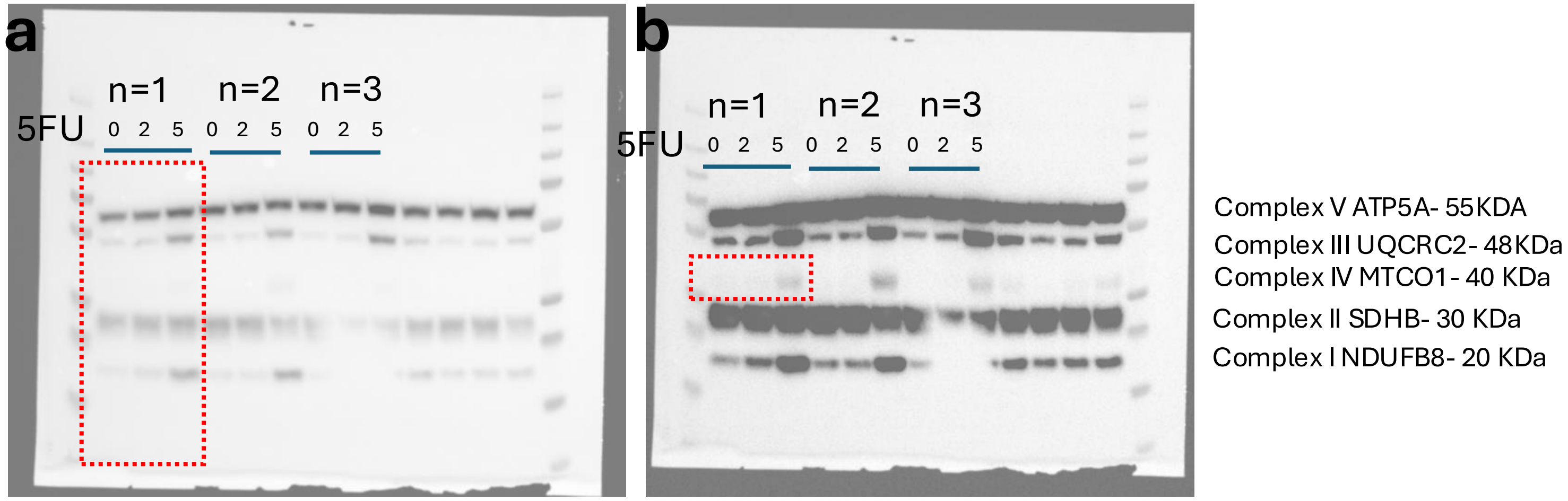

OXPHOS low and high exposure

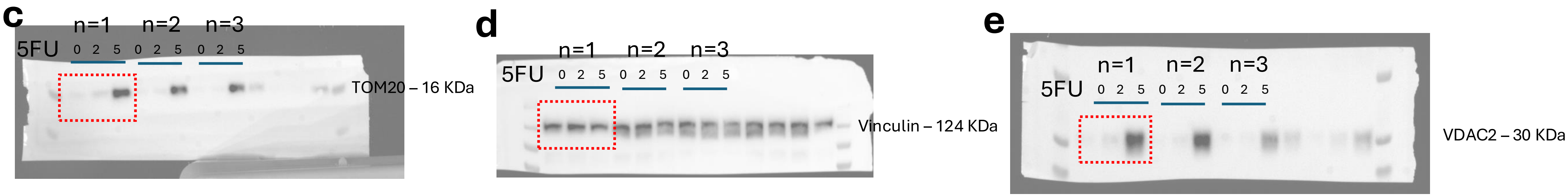

Blots show western images for three independent biological repeats of AKP cells in 2D culture treated ± 5FU for 72 hrs for electron transport chain panel (a, low exposure and b, high exposure), TOM20 (c), Vinculin (d) used as loading control and VDAC2 (e). Size is indicated. n indicates biological replicate.

Densitometry of all supplementary data presented for mitochondrial mass in excel table

SW480 data

Repeat 1	0	5	10	Repeat 2	0	5	10	Repeat 3	0	5	10 uM
V	3662.82	3592.82	2936.406	V	4877.184	4996.477	5213.77	V	2074.506	1878.506	1867.799
III	46.121	44.95	45.536	III	208.971	213.435	236.971	III	115.314	98.899	137.021
II	315.092	455.749	401.213	II	112.95	201.192	240.314	II			
IV	372.799	918.749	1286.749	IV	968.042	1823.87	2447.87	IV	123.021	174.314	198.142
I	137.314	353.971	279.142	I	336.213	551.799	567.385	I	2473.648	2924.355	4029.648
TOM20	3173.698	3857.527	4862.648	TOM20	2325.749	3380.335	3662.92	TOM20	3019.92	6505.456	5749.163
VDAC2	1646.113	3268.406	5434.991	VDAC2	1711.87	3802.87	4925.87	VDAC2	3031.92	5949.749	6039.042
ACTIN	4029.698	3577.991	4010.991	ACTIN	4356.234	4092.82	3864.82	ACTIN	2156.678	1648.506	1451.971
Norm to loading											
V	0.90895645	1.0041445	0.7320899		1.11958724	1.2207908	1.34903307		0.90895645	1.0041445	0.7320899
III	0.01144527	0.01256292	0.01135281		0.04797056	0.05214864	0.06131489		0.01144527	0.01256292	0.01135281
II	0.07819246	0.12737567	0.1000284		0.02592836	0.0491573	0.06217987		0.07819246	0.12737567	0.1000284
IV	0.09251289	0.2567779	0.32080576		0.22221993	0.44562673	0.63337232		0.09251289	0.2567779	0.32080576
I	0.03407551	0.0989301	0.06959427		0.07717974	0.13482122	0.14680761		0.03407551	0.0989301	0.06959427
TOM20	0.78757713	1.07812652	1.21233082		0.53388982	0.82591832	0.94775953		0.78757713	1.07812652	1.21233082
VDAC2	0.40849538	0.91347519	1.35502448		0.39297017	0.92915643	1.2745406		0.40849538	0.91347519	1.35502448
FC from CTRL											
V		1.10472235	0.80541801			1.09039363	1.20493788			1.10472234	0.80541802
III		1.0976509	0.99192075			1.08709669	1.27817732			1.09765169	0.99192155
II		1.62900195	1.27925886			1.89588953	2.39814128			1.62900195	1.2792589
IV		2.77559053	3.46768718			2.00534101	2.85020487			2.77559052	3.46768715
I		2.90326127	2.04235475			1.74684738	1.90215226			2.90326102	2.04235447
TOM20		1.36891547	1.53931693			1.54698269	1.77519685			1.36891547	1.53931694
VDAC2		2.23619468	3.31711094			2.3644452	3.24335206			2.23619467	3.31711091

AKP 3D organoid data

	Rep1		Rep2		Rep3	
	CTRL	5FU	CTRL	5FU	CTRL	5FU
V	5246.719	5709.012	1458.527	1656.062	1648.406	2084.113
III	2116.577	2896.698	434.799	722.406	458.627	835.406
II	8284.326	9537.033	3020.619	3532.497	2857.326	3590.447
I	838.284	1250.991	164.657	468.406	64.95	184.314
TOM20	5639.184	9792.355	817.941	1120.406	497.749	1224.82
VDAC2	7300.113	7616.991	2434.991	3059.87	3014.163	3488.163
VINCULIN	1117.163	1055.577	4888.577	4081.577	4213.991	4010.991
V	4.69646685	5.40842781	0.2983541	0.40574072	0.39117454	0.51960052
III	1.89459998	2.74418446	0.08894183	0.17699188	0.10883436	0.2082792
II	7.41550338	9.03490034	0.61789331	0.86547357	0.67805698	0.8951521
I	0.75036857	1.18512529	0.03368199	0.11476103	0.01541294	0.04595223
TOM20	5.0477719	9.27677943	0.16731679	0.27450321	0.11811819	0.30536593
VDAC2	6.53451018	7.21595014	0.49809812	0.74967837	0.71527514	0.86965117
V		1.15159501		1.3599301		1.32830862
III		1.4484242		1.98997341		1.91372657
II		1.21837991		1.40068448		1.32017238
I		1.57939091		3.40719286		2.98140575
TOM20		1.83779688		1.64061967		2.5852574
VDAC2		1.10428325		1.50508172		1.21582747

AKP 2D cell line data

	Rep1			Rep2			Rep3		
	0	0.5	2	0	0.5	2	0	0.5	2
V	5319.92	5935.627	6910.799	7035.92	7309.87	7586.042	7224.284	7336.577	7448.991
III	3149.799	3707.678	4936.092	2684.092	2356.092	3358.092	1672.385	2602.385	4537.385
IV	534.314	564.678	1101.799	875.506	421.607	1719.213	171.778	228.607	962.213
III	7270.527	7750.406	8158.991	8170.284	7831.577	6851.87	5191.527	2722.284	4502.82
I	2101.749	3325.163	5071.456	2127.749	2372.335	4399.042	105.92	249.95	375.799
TOM20	963.385	896.799	4055.506	924.799	381.971	2687.092	176.142	211.849	1157.092
VDAC2	365.849	744.556	5266.678	272.728	483.678	5082.385	277.971	441.314	2615.092
VINCULIN	3582.799	3792.799	2862.092	4176.092	4271.799	3354.799	4020.506	3263.092	3037.385
V	1.48485025	1.56497273	2.41459708	1.68480963	1.7111924	2.26125082	1.7968594	2.24835126	2.45243557
III	0.87914477	0.97755721	1.72464477	0.64272818	0.55154561	1.00098158	0.41596381	0.79752119	1.49384586
IV	0.14913312	0.1488816	0.38496282	0.2096472	0.09869542	0.51246379	0.04272547	0.0700584	0.31678994
III	2.02928688	2.04345287	2.85070885	1.95644253	1.83332058	2.0424085	1.29126209	0.83426517	1.482466
I	0.58662208	0.87670425	1.77194025	0.50950721	0.55534799	1.31126842	0.02634494	0.07659913	0.12372452
TOM20	0.26889172	0.23644781	1.41697262	0.22145082	0.08941689	0.8009696	0.0438109	0.06492278	0.38095006
VDAC2	0.10211262	0.19630779	1.84014979	0.06530699	0.11322583	1.51495961	0.06913831	0.13524412	0.86096823
V		1.05395997	1.62615528		1.0156592	1.34214026		1.25126721	1.36484556
III		1.11194111	1.96173012		0.858132	1.55739489		1.91728502	3.59128803
IV		0.99831347	2.58133681		0.47076908	2.44441034		1.63973391	7.41454574
III		1.00698077	1.40478356		0.93706845	1.04393994		0.64608508	1.14807521
I		1.49449583	3.02058226		1.08997081	2.57360129		2.9075458	4.69632905
TOM20		0.87934207	5.26967732		0.40377766	3.61691862		1.48188633	8.69532539
VDAC2		1.92246354	18.0207868		1.73374753	23.1975108		1.95613855	12.4528384

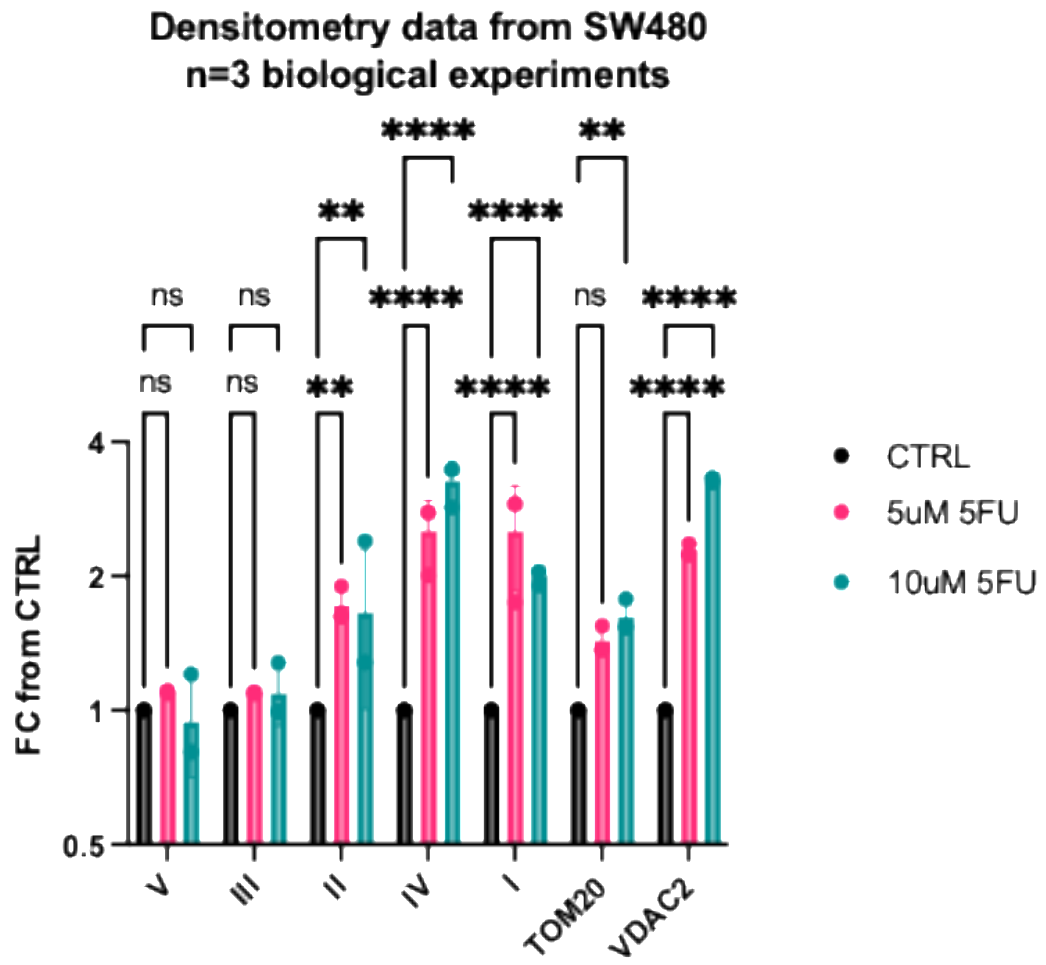

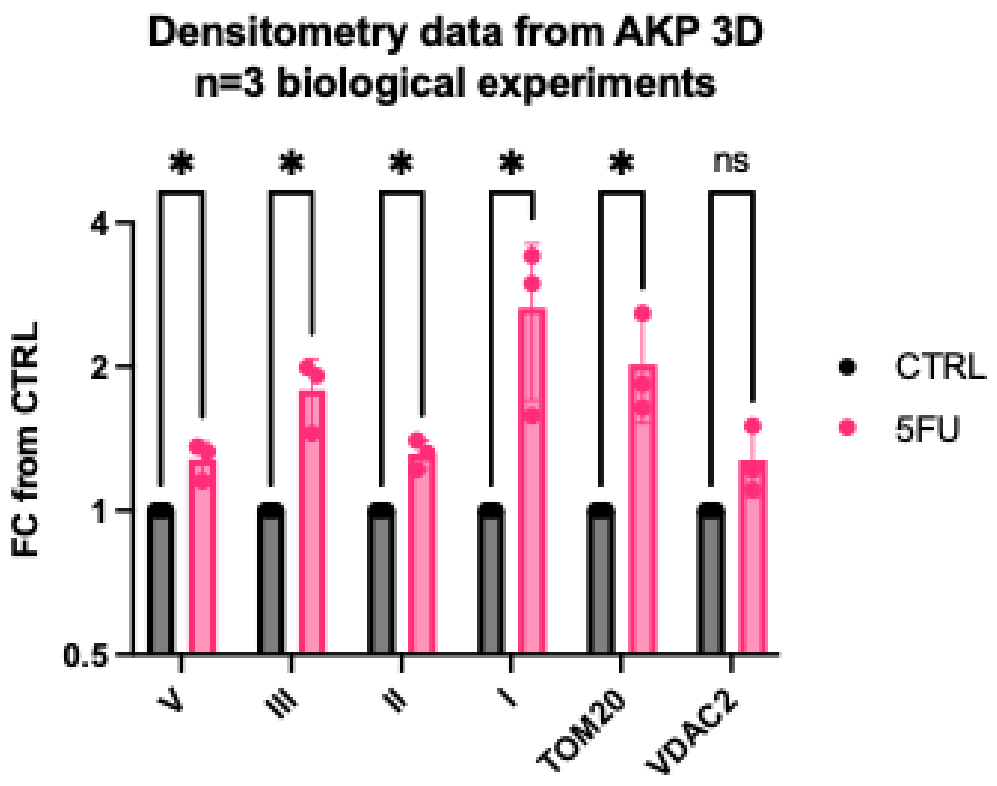

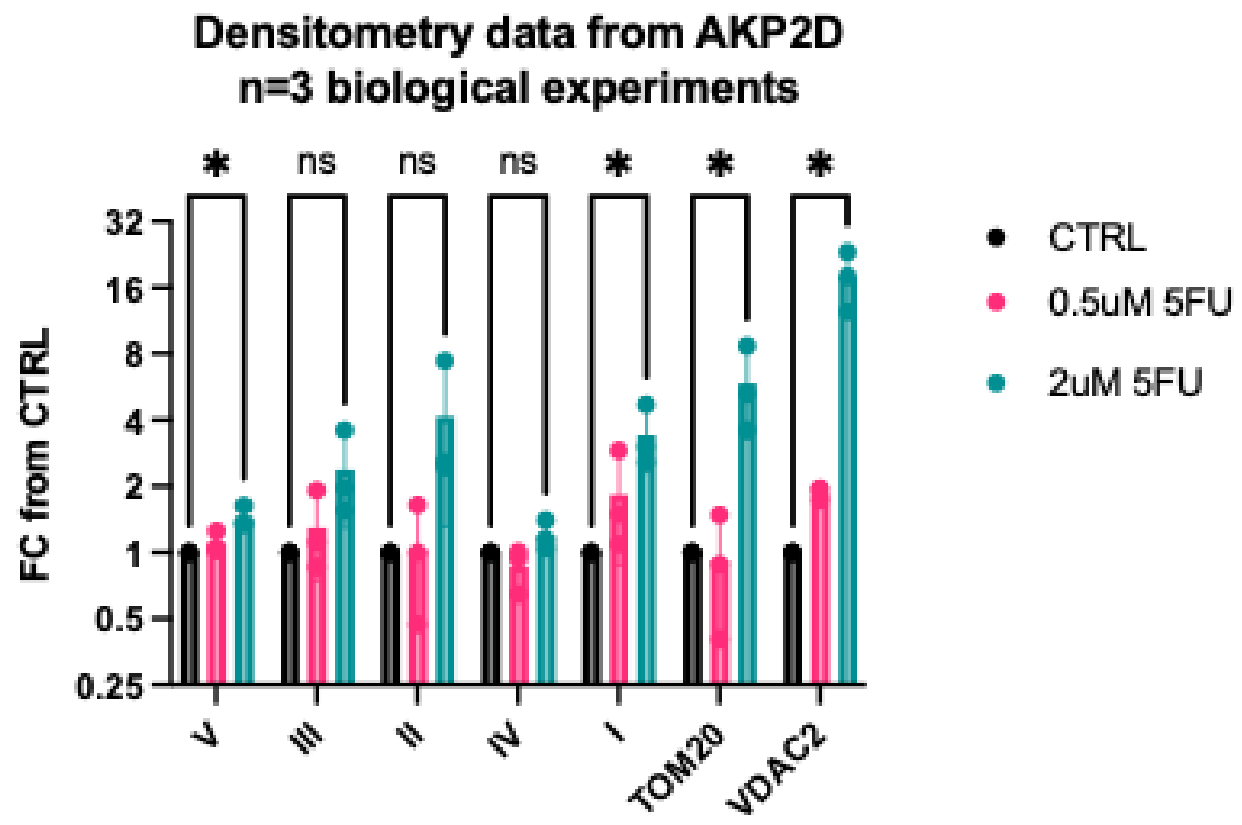

Table shows densitometry values for blots for SW480 (n=1-3, slide 9), AKP organoids (n=1-3, slide 10) and AKP 2D cell line (n=1-3, slide 11), CTRL vs 5FU treated as indicated at 72hrs, with raw values then normalised to Actin and expressed as fold change over CTRL. Graph shows expression fold changes as a function of CTRL (FC from CTRL) for each replicate ± sem, with statistical testing by ANOVA+Dunnett's/t-test as indicated. *NB. SW480 data CIII n=3, data was not analysable due to low expression.*

Figure 1f

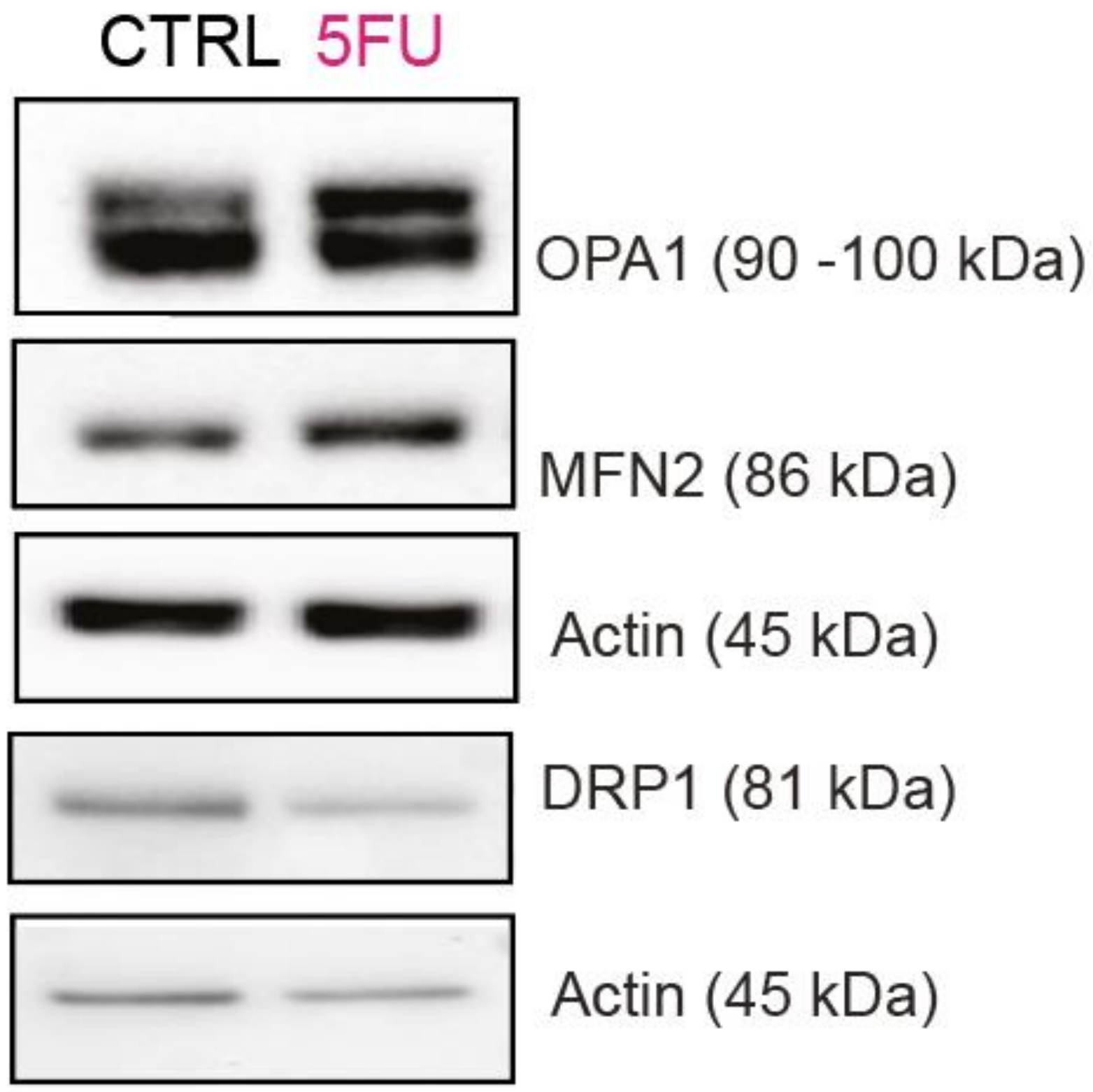

WB Observation 2
5FU treatment changes mitochondrial dynamics proteins

DRP1 blots used for image
HCT116 at 72hrs ± 5FU treatment

a

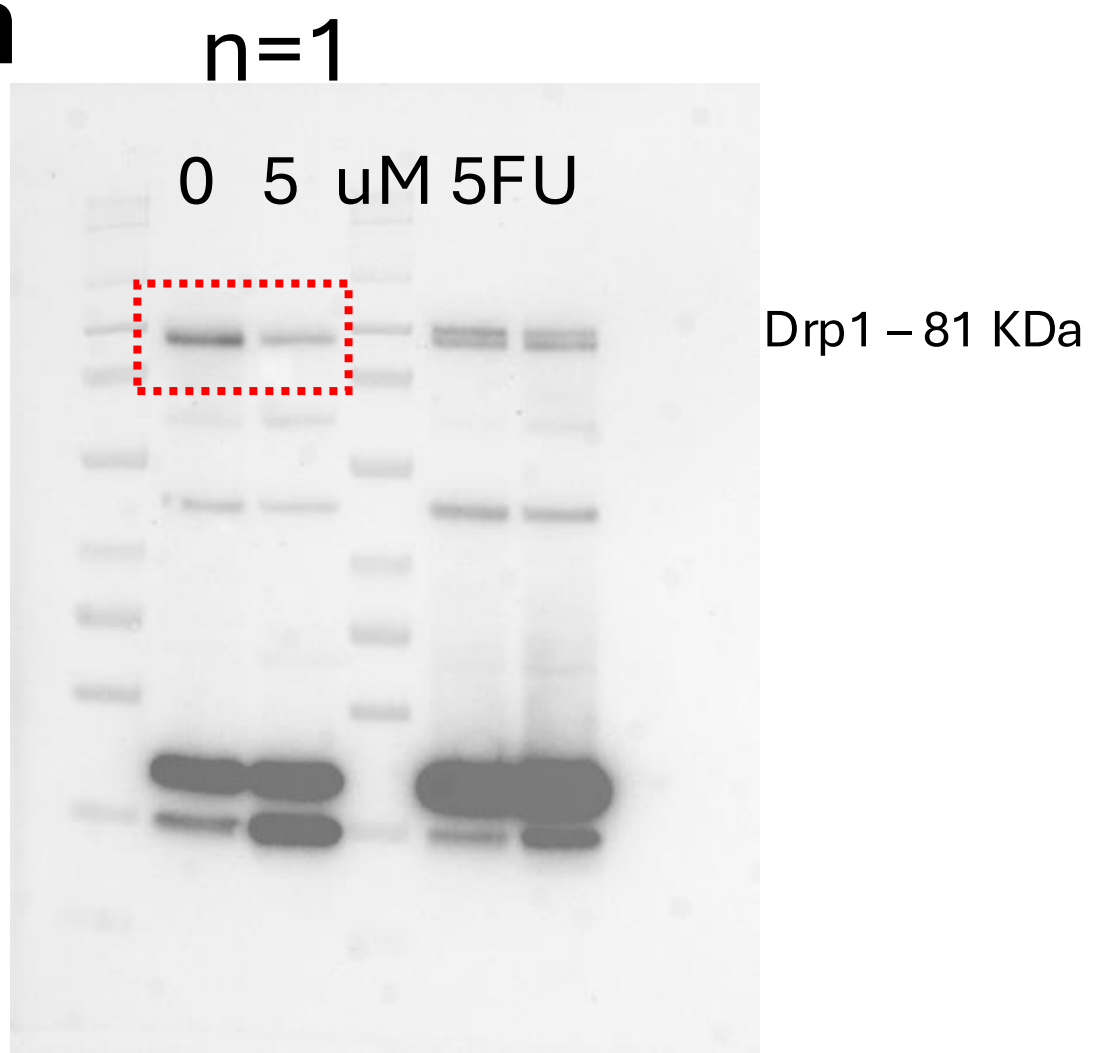

b

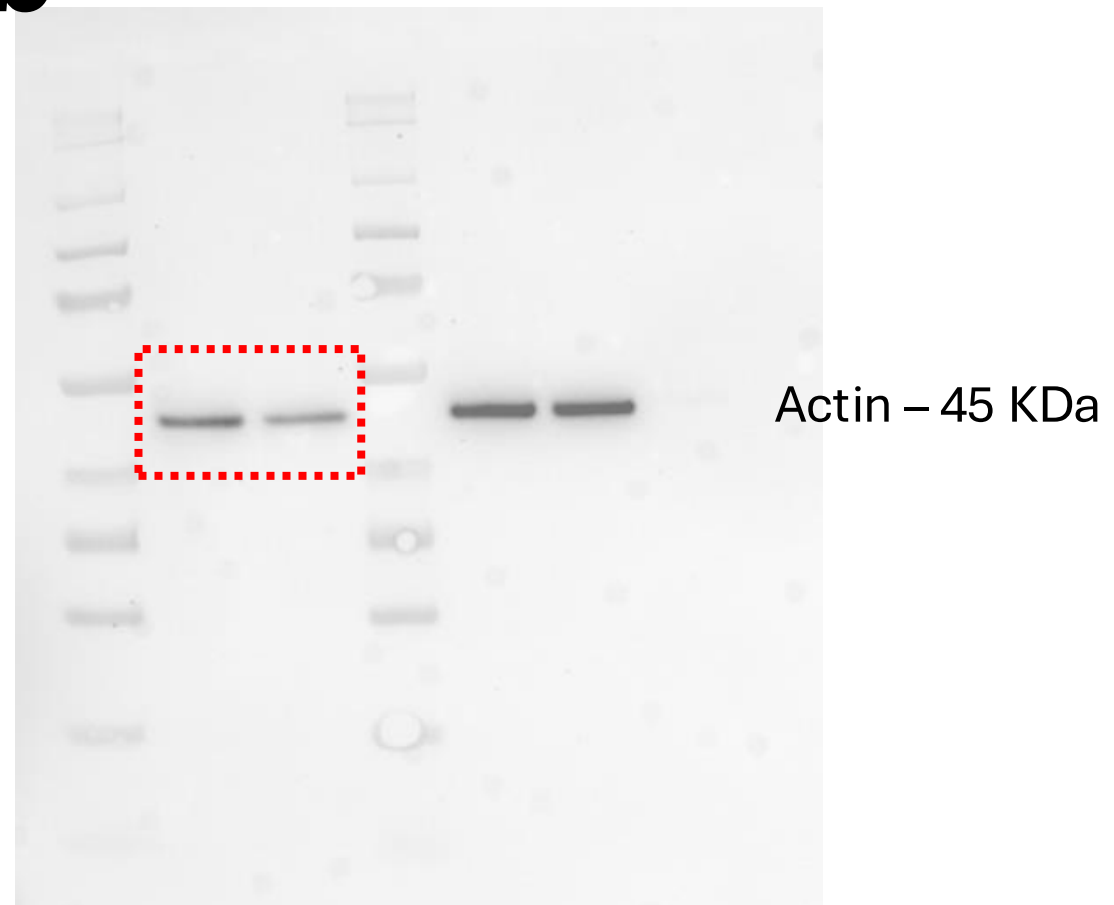

c

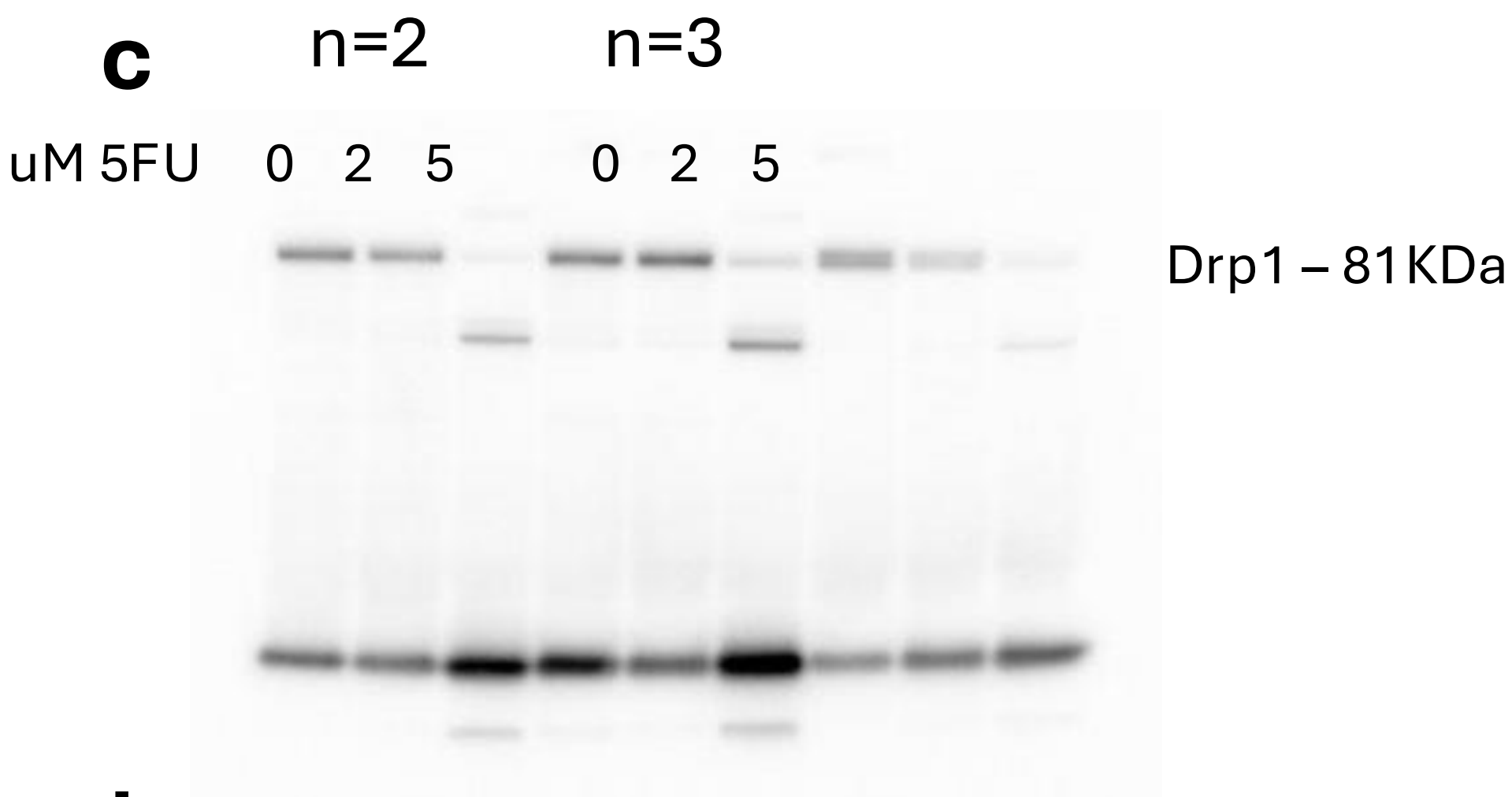

d

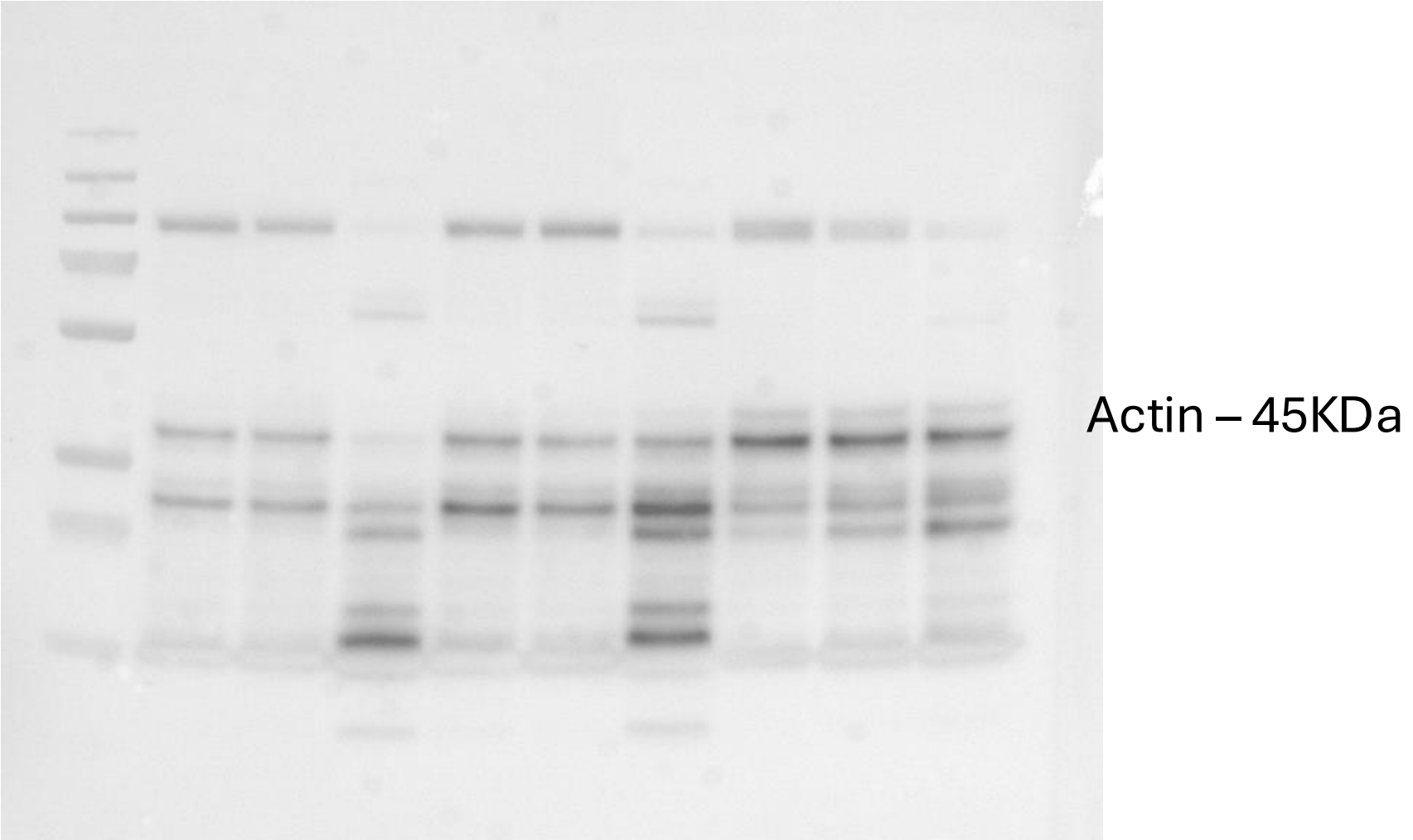

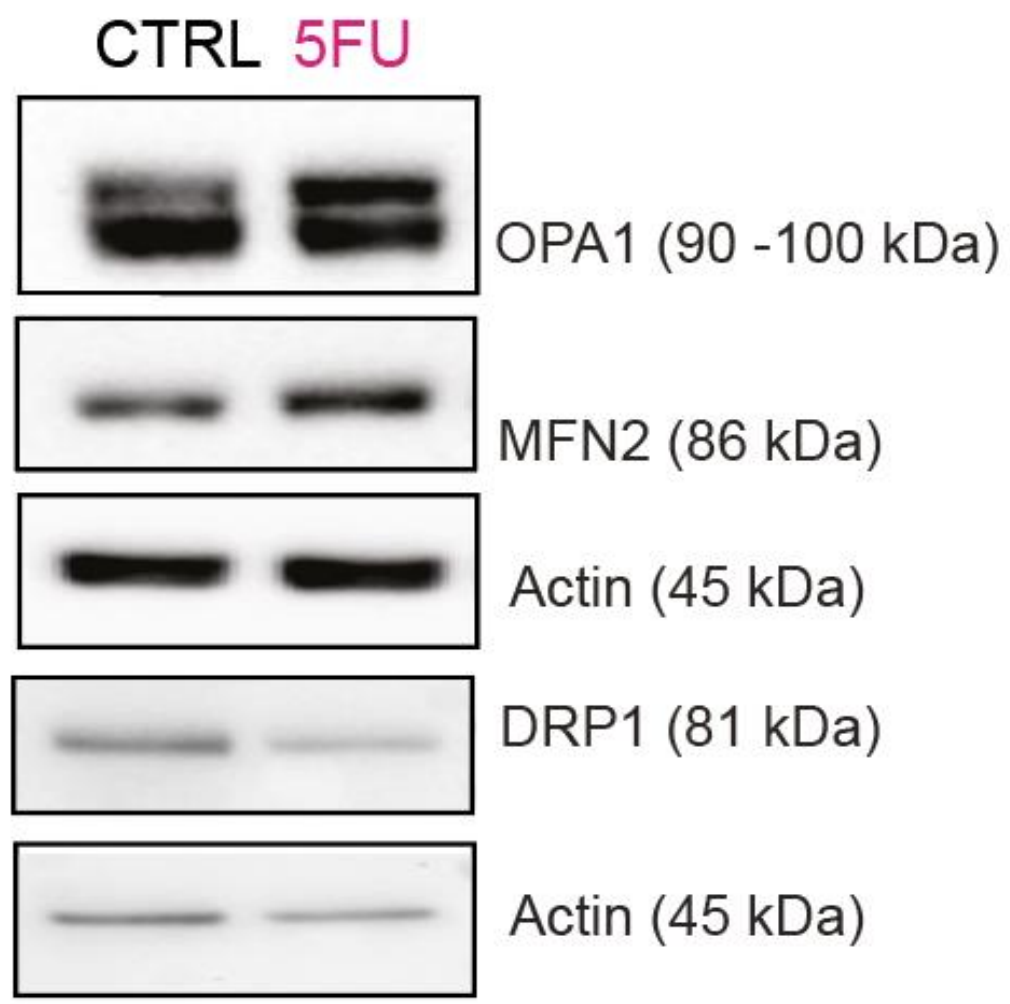

Blots show western images for three independent biological repeats of HCT116 cells treated ± 5FU for 72 hrs as indicated for DRP1 (a & c), with Actin (b & d) used as loading control. Size is indicated. n indicates biological replicate.

Blots used for OPA1 and MFN2 image ± 5FU in HCT116 at 72hrs

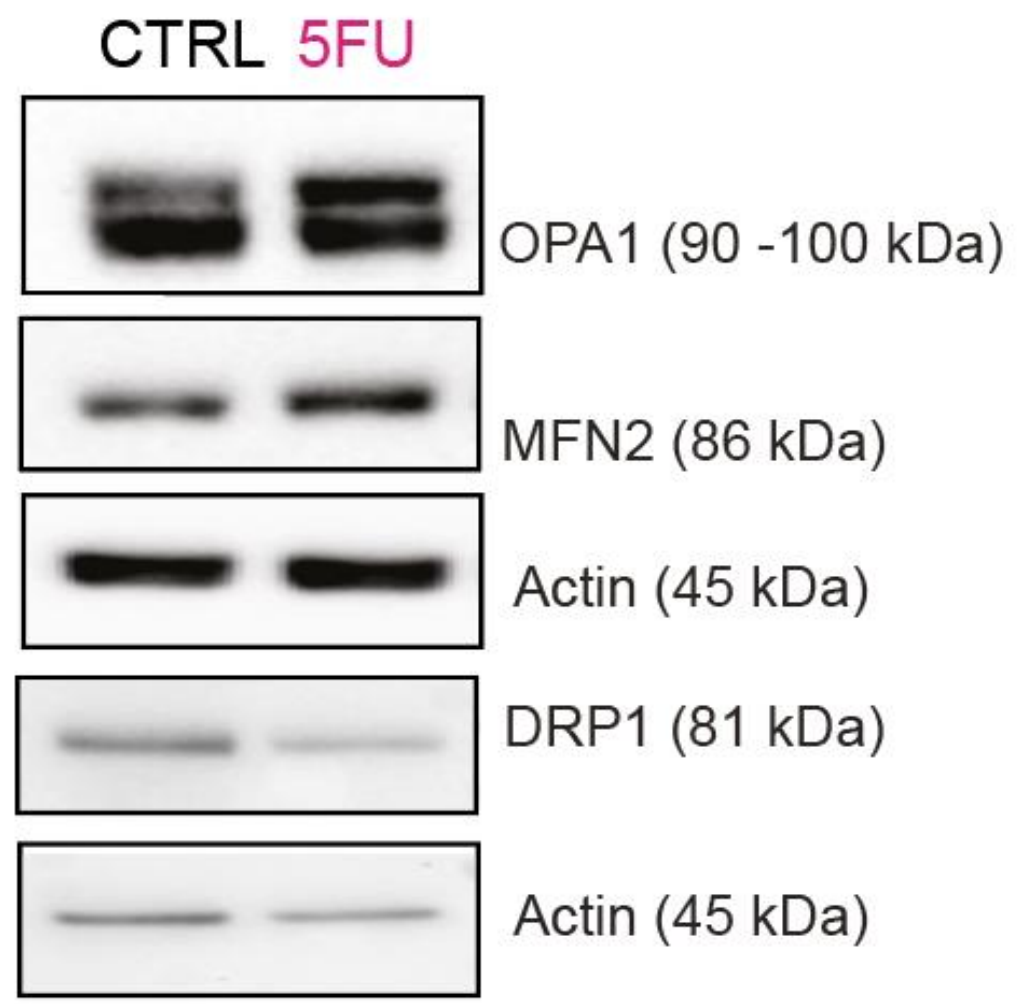

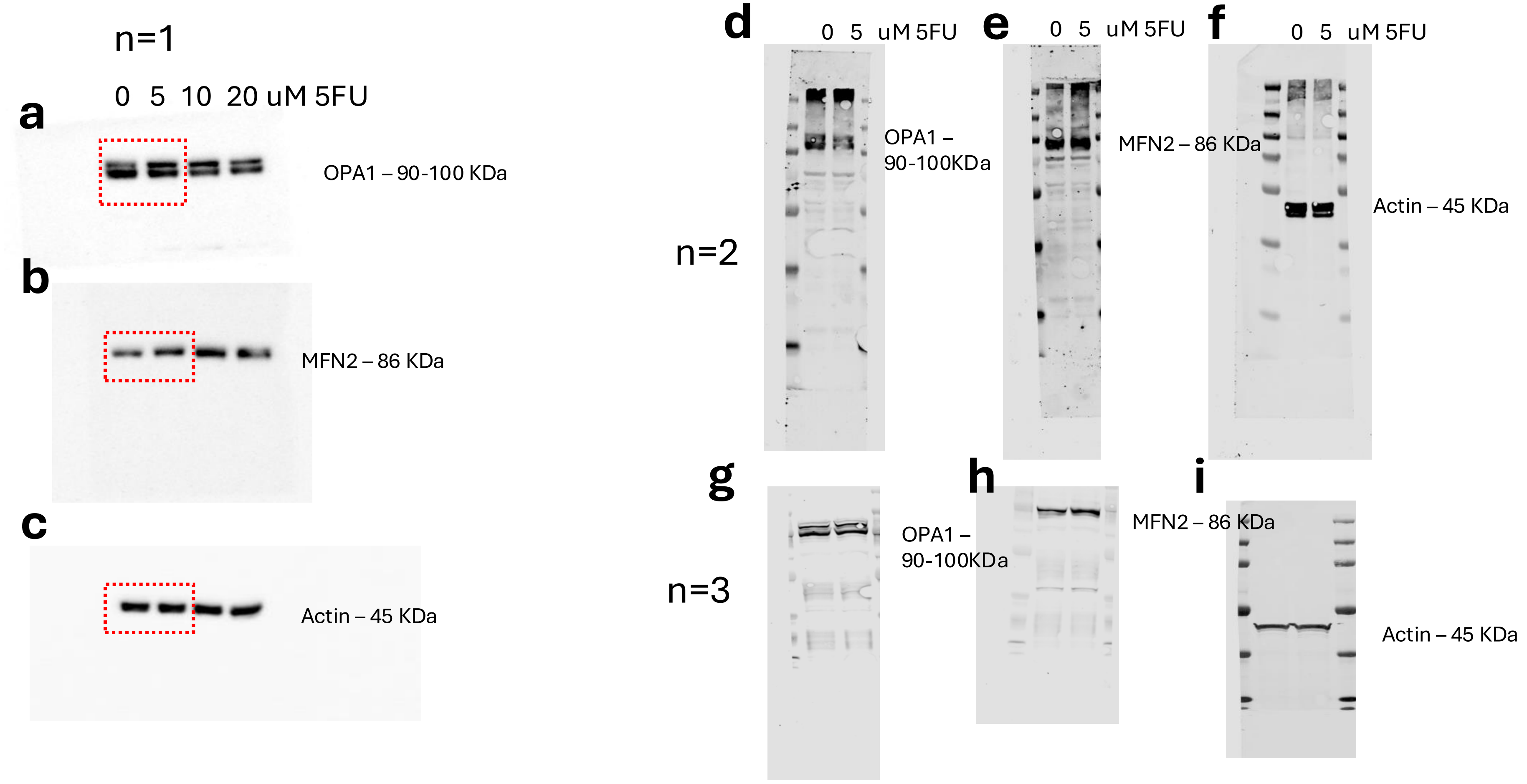

Blots show western images for three independent biological repeats of HCT116 cells treated ± 5FU for 72 hrs as indicated for OPA1 (a, d, g), MFN2 (b, e, h) with Actin (c, f, i) used as loading control. Size is indicated. n indicates biological replicate.

further repeat experiments in HCT116 lines; blots show OPA1, MFN2, Actin, Vinculin and ponceau of membranes

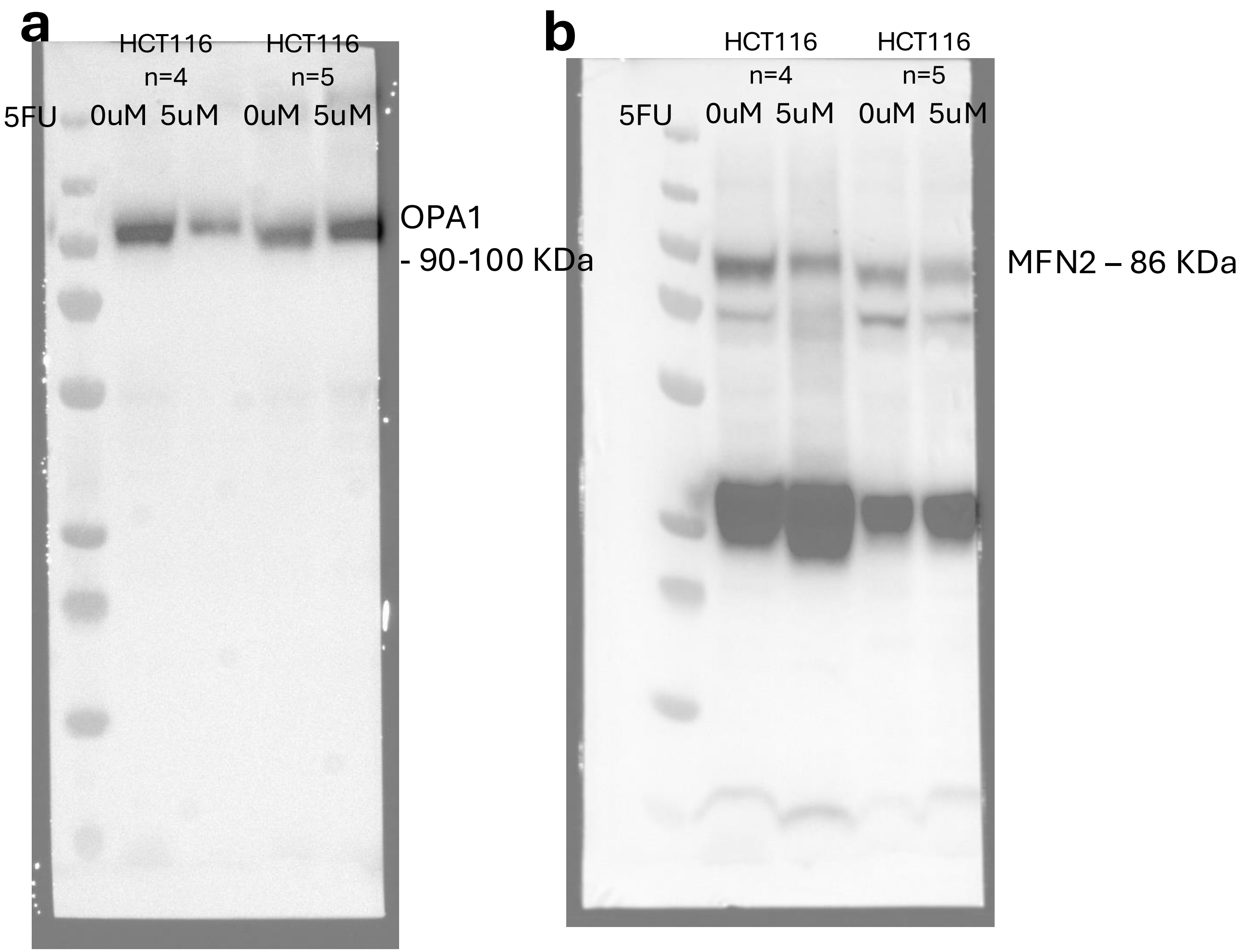

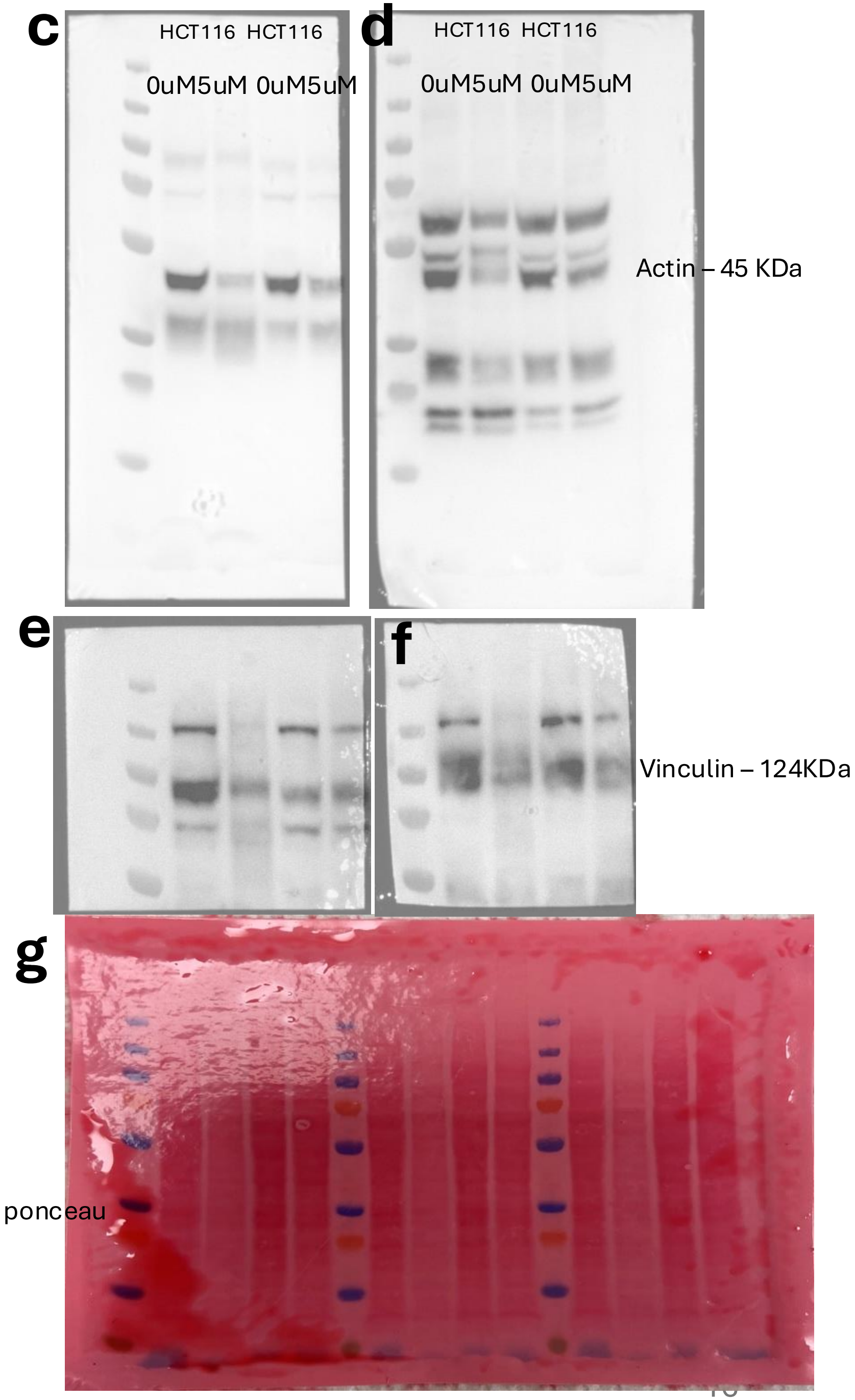

Image of ponceau

Blots show western images for two further independent biological repeats of HCT116 cells treated ± 5FU for 72 hrs as indicated for OPA1 (a), MFN2 (b) with Actin (c & d), Vinculin (e & f) and ponceau (g) used as loading control. Size is indicated. n indicates biological replicate.

Densitometry of all HCT116 data presented for mitochondrial dynamics proteins

Rep1	CTRL	5FU	Rep2	CTRL	5FU	Rep3	CTRL	5FU	Rep4	CTRL	5FU	Rep 5	CTRL	5FU
OPA U	2216.799	3325.92		4161.456	9625.326		3825.213	3280.042		4643.577	2950.82		3181.406	4951.284
OPA L	4388.92	3464.799		6001.991	3256.749		5787.627	2620.506		3174.577	467.092		2773.82	2071.335
Total OPA1	6605.719	6790.719		10163.447	12882.075		9612.84	5900.548		7818.154	3417.912		5955.226	7022.619
MFN	2302.627	3101.335		7436.284	10646.577		6237.87	7158.042		12563.012	8626.941		5767.305	6064.335
ACTIN	3817.163	3877.456		8909.991	8690.82		4571.335	2227.213		11113.305	4804.891		8854.598	5688.012
DRP1	3473.527	1493.284		2847.456	255.385		3248.87	831.335						
ACTIN	3467.477	2154.284		1759.87	1595.284		3902.284	4789.284						
Normalised														
OPA U	0.58074518	0.85775828		0.46705502	1.10752794		0.83678247	1.47271141		0.41783943	0.6141284		0.35929423	0.87047707
OPA L	1.14978585	0.89357532		0.67362481	0.37473437		1.26606932	1.17658527		0.28565553	0.09721178		0.31326323	0.36415799
Total OPA1	1.73053103	1.75133361		1.14067983	1.48226232		2.10285179	2.64929668		0.70349495	0.71134017		0.67255747	1.23463505
MFN	0.60322994	0.79983757		0.83460062	1.22503711		1.36456199	3.21390096		1.13044787	1.79544989		0.65133448	1.06616073
DRP1	1.00174478	0.69316952		1.61799224	0.16008748		0.83255601	0.17358231						
/CTRL														
OPA U		1.47699597			2.37130082			1.75996924			1.46977129			2.42274155
OPA L		0.77716674			0.55629539			0.92932136			0.34031121			1.16246641
Total OPA1		1.01202092			1.29945519			1.25985896			1.01115178			1.83573169
MFN		1.32592486			1.46781237			2.35526196			1.5882642			1.63688666
DRP1		0.69196219			0.09894206			0.20849326						

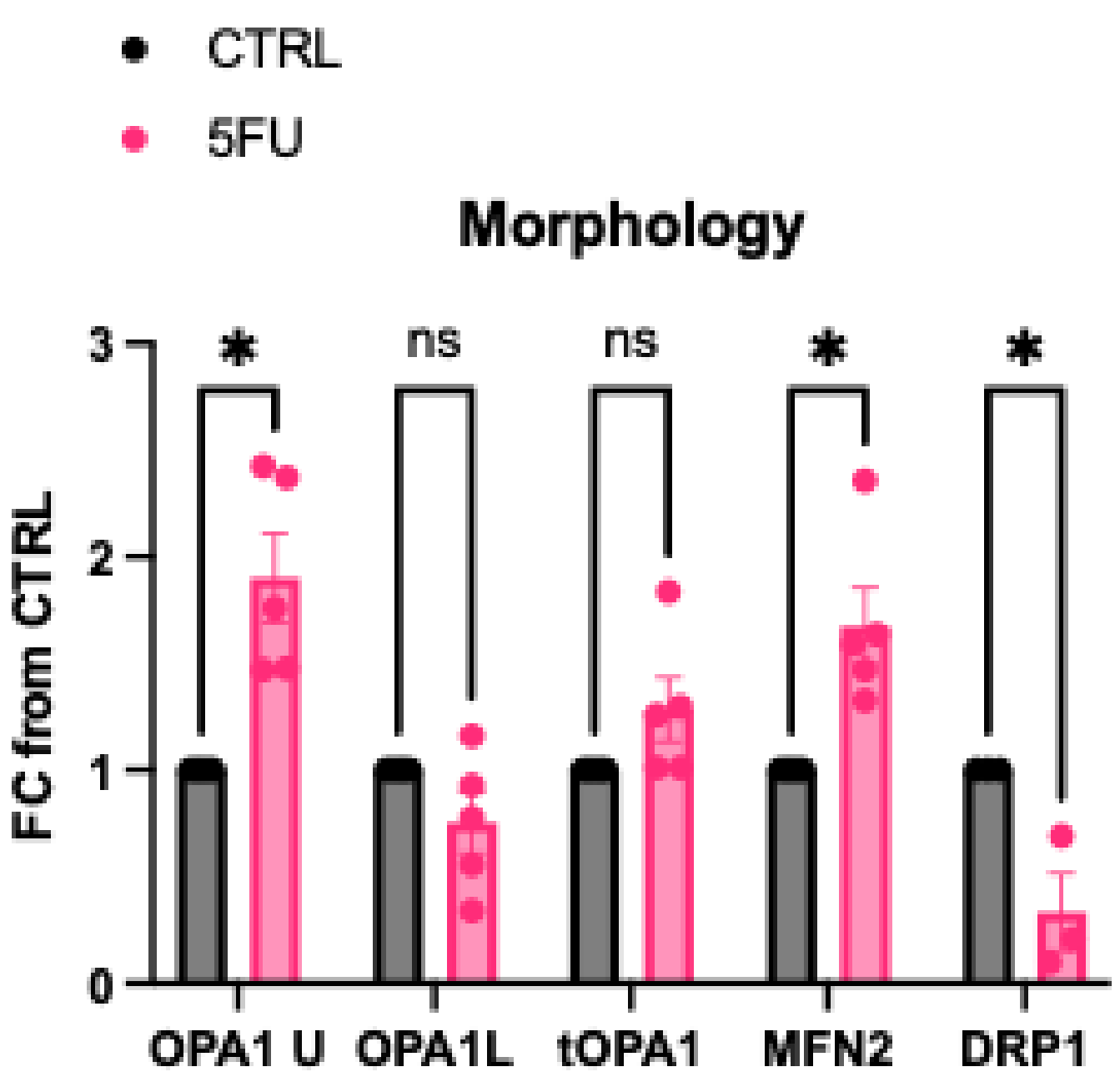

Table shows densitometry values for blots presented across slides 14-16, CTRL vs 5uM 5FU treated at 72hrs, with raw values then normalised to Actin and expressed as fold change over CTRL. Graph shows expression fold changes as a function of CTRL (FC from CTRL) for each replicate± sem, with statistical testing by t-test as indicated. *NB. DRP1 expression n=3, MFN2/OPA1 expression n=5.*

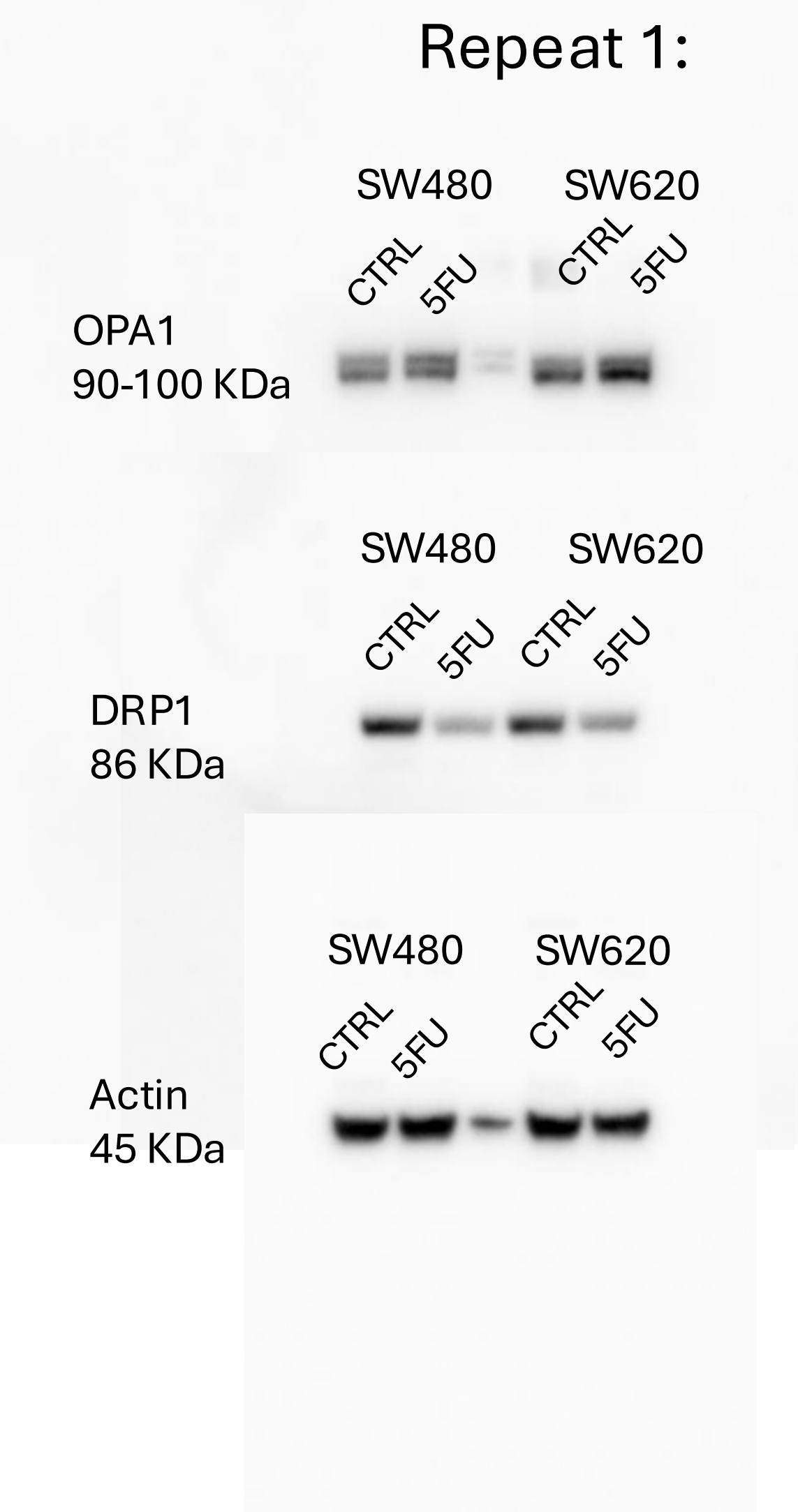

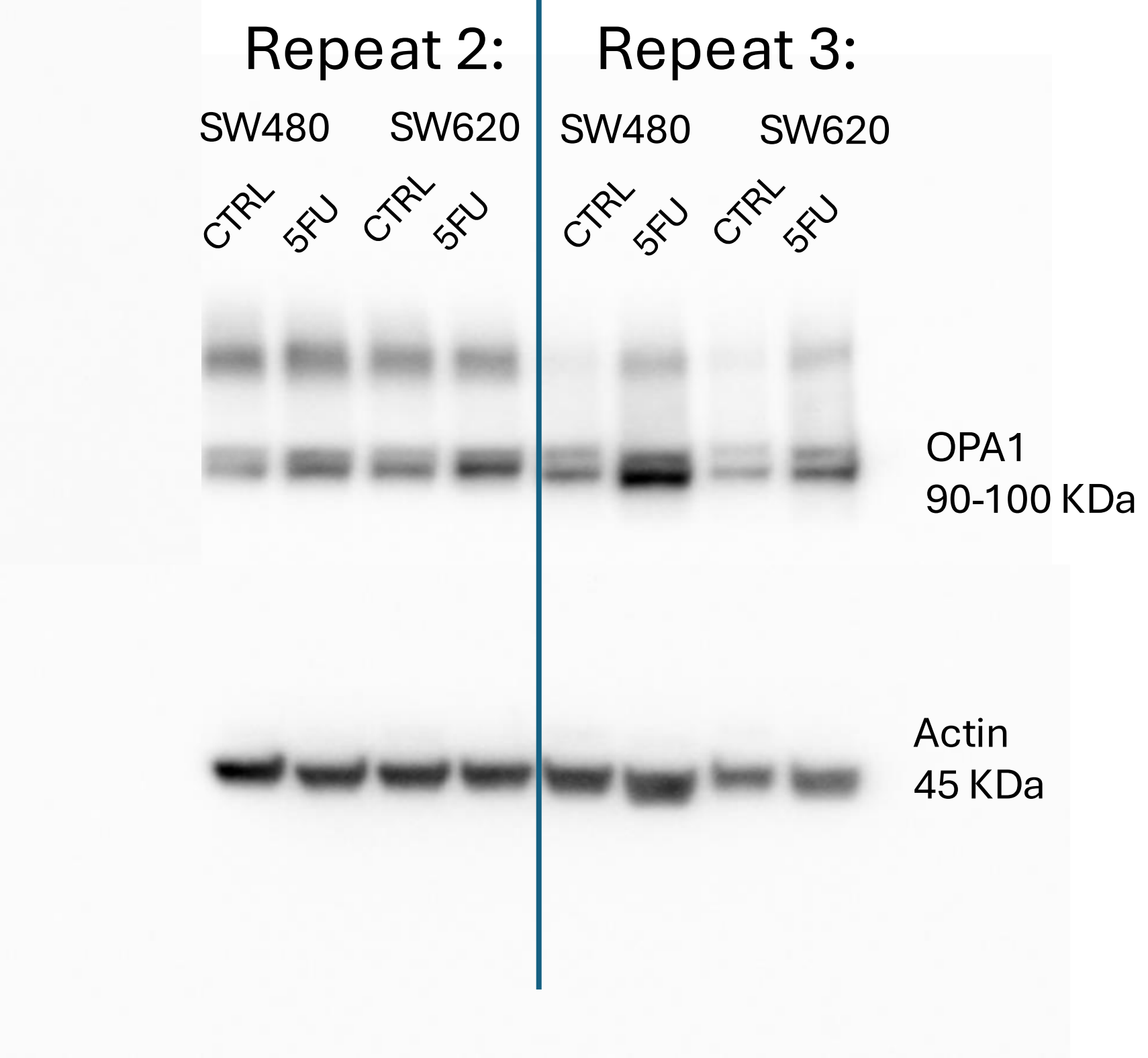

Additional models:
SW480 and SW620 (data not currently shown in manuscript but can be included in supp if needed)

	Rep1				Rep2				Rep3			
	SW480		SW620		SW480		SW620		SW480		SW620	
	CTRL	5FU	CTRL	5FU	CTRL	5FU	CTRL	5FU	CTRL	5FU	CTRL	5FU
OPA1 L	1597.435	2252.728	1645.435	2371.435	3123.163	5072.891	4058.406	5557.991	3541.456	7645.184	2576.406	5846.841
OPA1 U	1803.385	2723.678	2513.971	3032.263	3696.134	6232.527	5643.184	7996.719	5806.406	16686.619	3713.406	7884.255
OPA1 total	3400.82	4976.406	4159.406	5403.698	6819.297	11305.418	9701.59	13554.71	9347.862	24331.803	6289.812	13731.096
DRP1	11211.426	4669.527	10052.598	5357.234								
Actin	10382.82	11308.062	10825.305	9702.305	11614.477	10979.77	11133.113	10037.648	10927.527	11791.962	7319.062	7826.355
Norm. to actin												
OPA1 L	0.15385367	0.19921433	0.15199895	0.24441975	0.26890259	0.46202161	0.3645347	0.55371448	0.32408577	0.64833859	0.35201314	0.74707076
OPA1 U	0.17368933	0.24086161	0.23223096	0.31253017	0.31823508	0.5676373	0.50688285	0.79667259	0.53135591	1.41508419	0.50736092	1.00739808
OPA1 total	0.327543	0.44007594	0.38422991	0.55694992	0.58713767	1.02965891	0.87141755	1.35038706	0.85544167	2.06342278	0.85937406	1.75446884
DRP1	1.07980549	0.41293787	0.9286203	0.55216096								
FC rel to CTRL												
OPA1 L		1.29482986		1.6080358		1.7181746		1.51896233		2.00051545		2.12228089
OPA1 U		1.38673812		1.34577303		1.78370437		1.57170951		2.66315699		1.98556501
OPA1 total		1.34356692		1.44952254		1.75369246		1.54964411		2.41211393		2.04156599
DRP1		0.38241875		0.59460358								

Extended data 1e

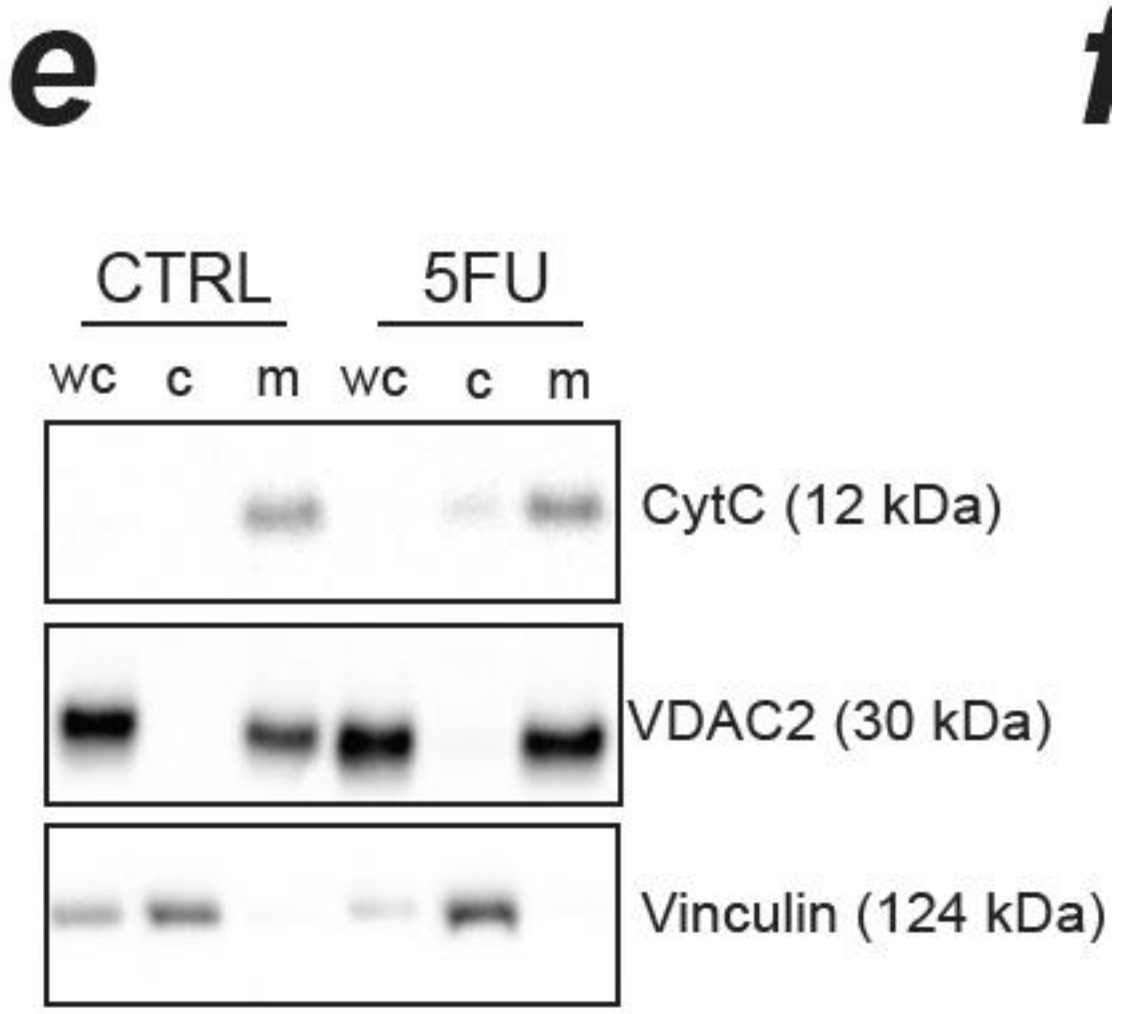

WB Observation 3

Significant cytochrome *c* release is not a major phenotype driven by 5FU treatment

Raw data and repeats for Ext. data figure 1e; blots show three independent fractionation extractions probed for cytochrome C and loadings.

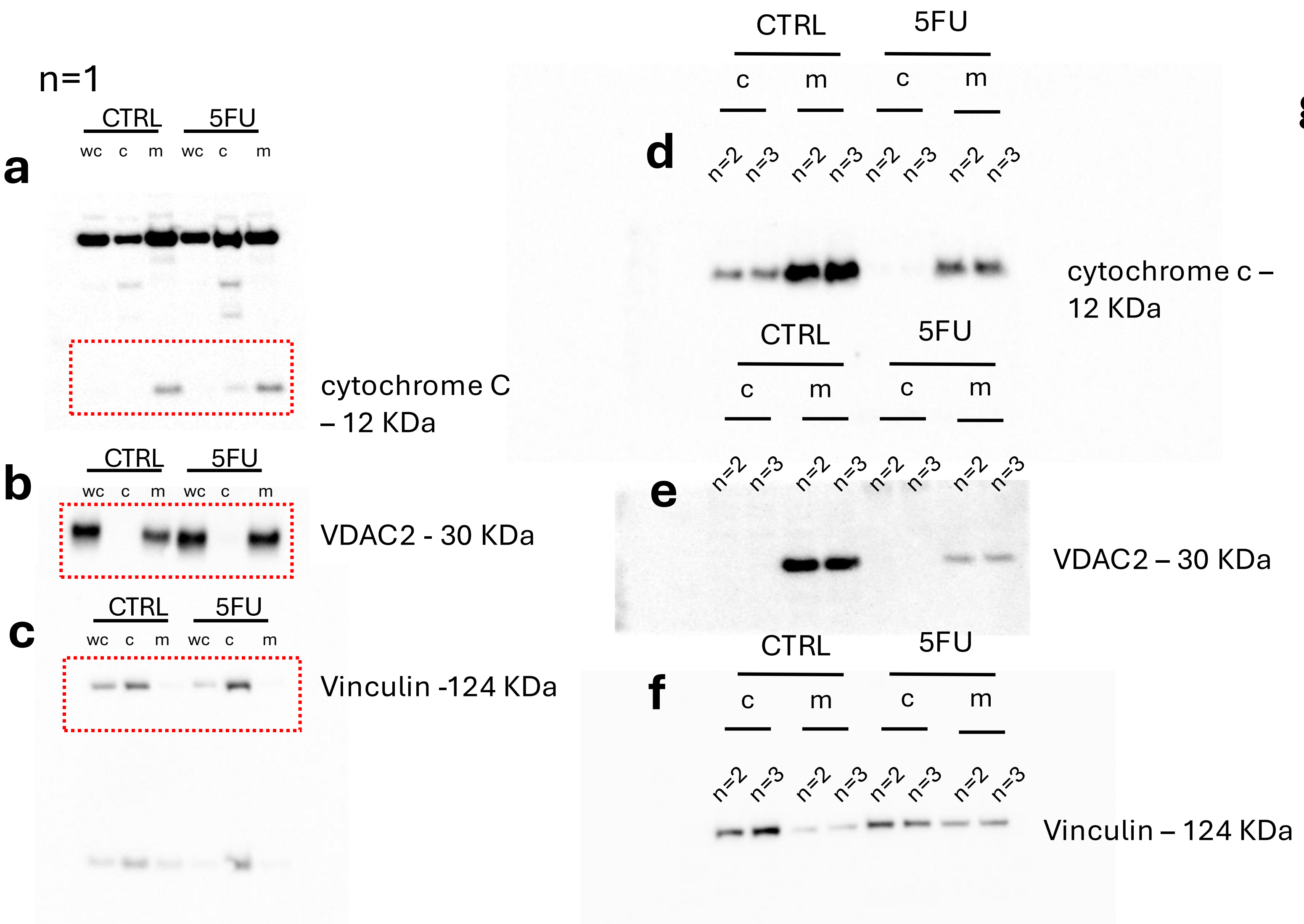

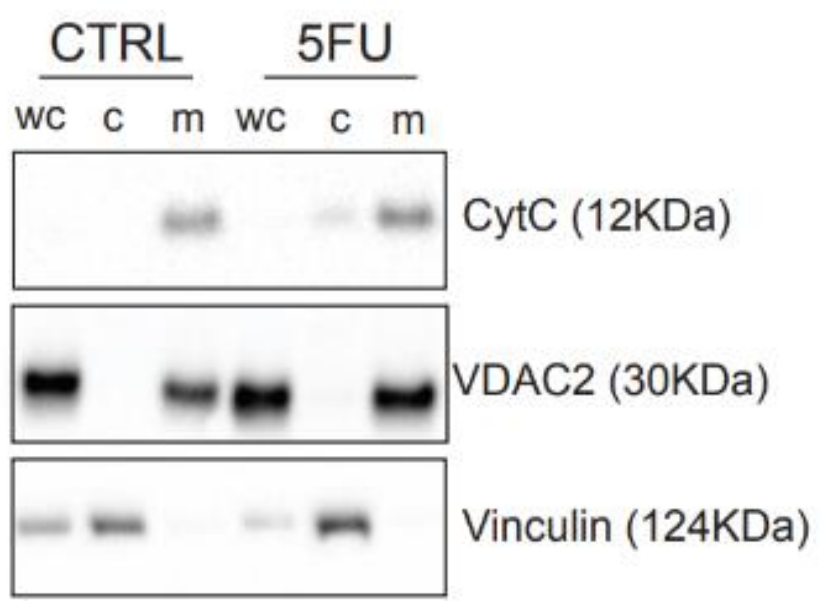

g

	Rep1			Rep2	Rep3		Rep2	Rep3		Ratio c/m	CTRL	5FU
**Cyt C**	CTRL	5FU		CTRL	CTRL		5FU	5FU		n=1	0.023	0.198
whole cell	182.19	202.31								n=2	0.231	0.307
cytosolic	95.24	963.16		1569.53	2271.23		843.41	242.44		n=3	0.323	0.088
mitochondrial	4166.46	4858.87		6780.94	7032.94		2746.23	2749.23				
Ratio cytosolic/mitochondrial	0.02	0.20		0.23	0.32		0.31	0.09				
**VIN**												
wc	1171.51	432.68										
c	2069.04	3009.28		4959.41	4910.41		2246.28	2048.28				
m	42.95	42.36		675.87	635.87		1295.41	940.41				
**VDAC**												
wc	5579.55	6732.60										
c	90.95	222.61		14.12	132.78		237.24	133.24				
m	4225.01	6237.18		6033.46	5469.87		970.63	1027.04				

h

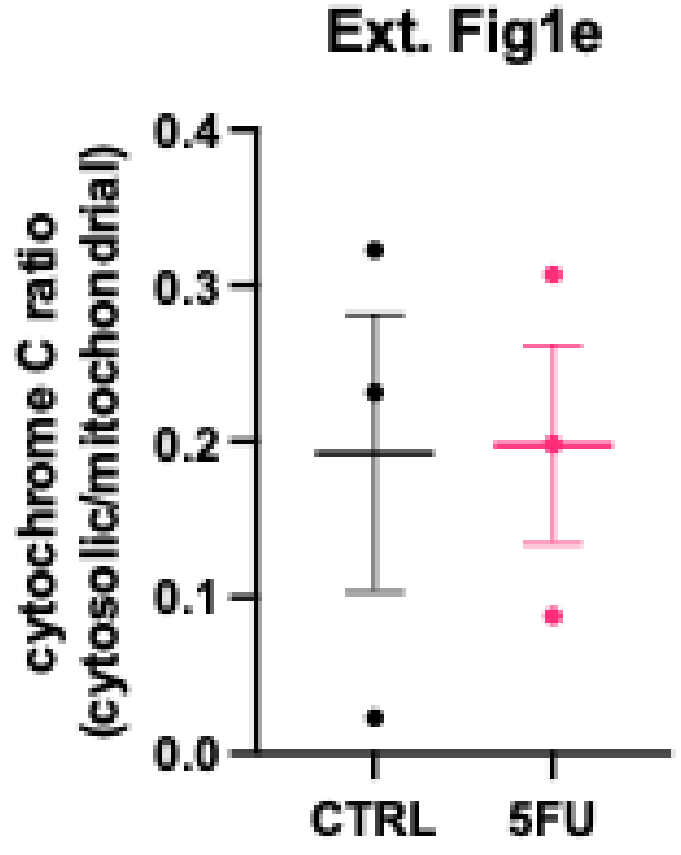

Blots show western images for three independent biological repeats of HCT116 cells treated ± 5FU for 72 hrs before mitochondrial fractionation according to kit (ab65311) as indicated for cytochrome *c* (a, d), VDAC2 (b, e) and Vinculin (c, f). Size is indicated. n indicates biological replicate. c= cytosolic, m=mitochondrial, wc=whole cell lysate. Densitometry values (g) were used to calculate cytosolic:mitochondrial fractions for three repeats (h).

Figure 3c

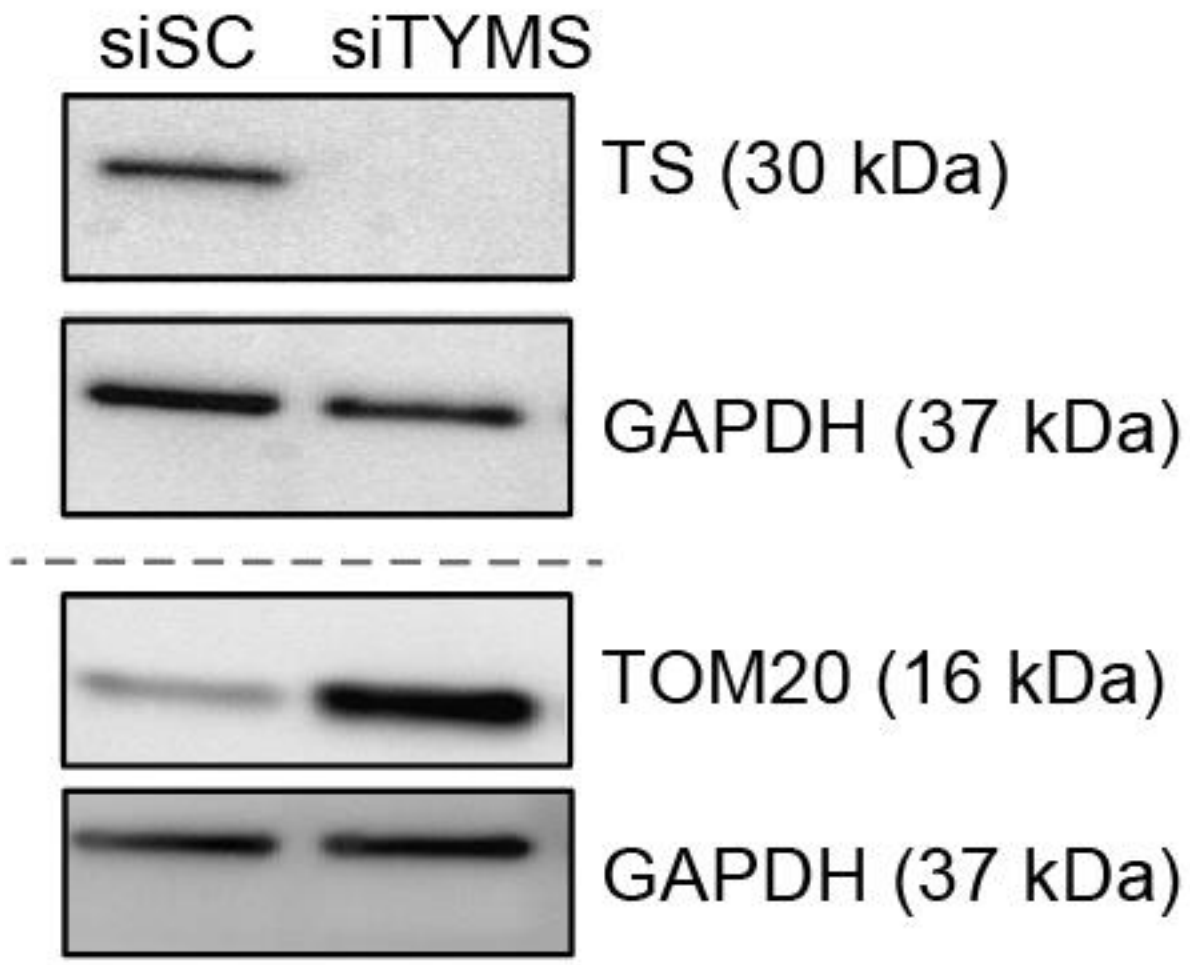

WB Observation 4

TS knockdown increases mitochondrial protein abundance

Blots used for Figure 3c image plus replicates;

** 40nM experiment was cautioned due to high cell death in controls and knockdowns

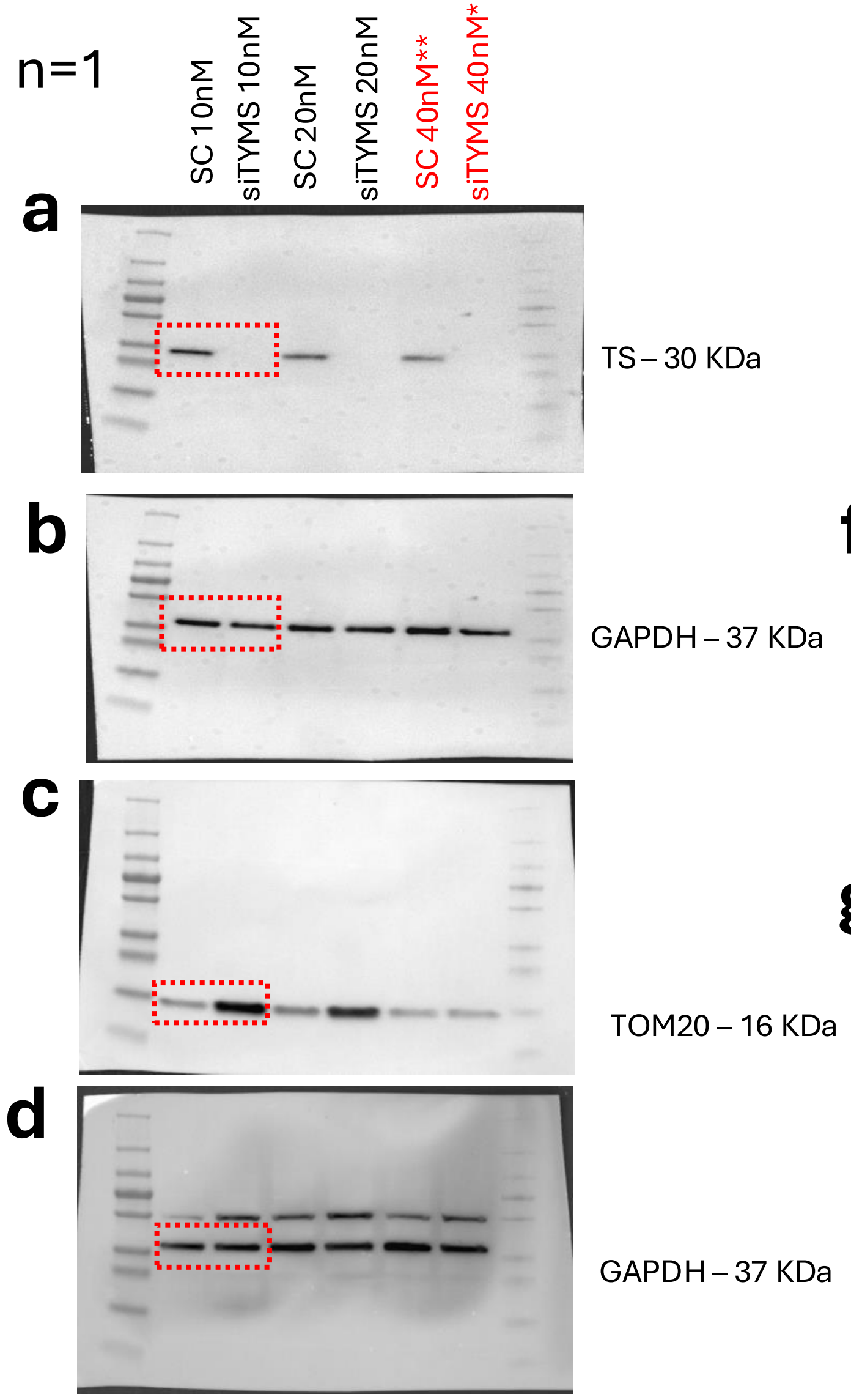

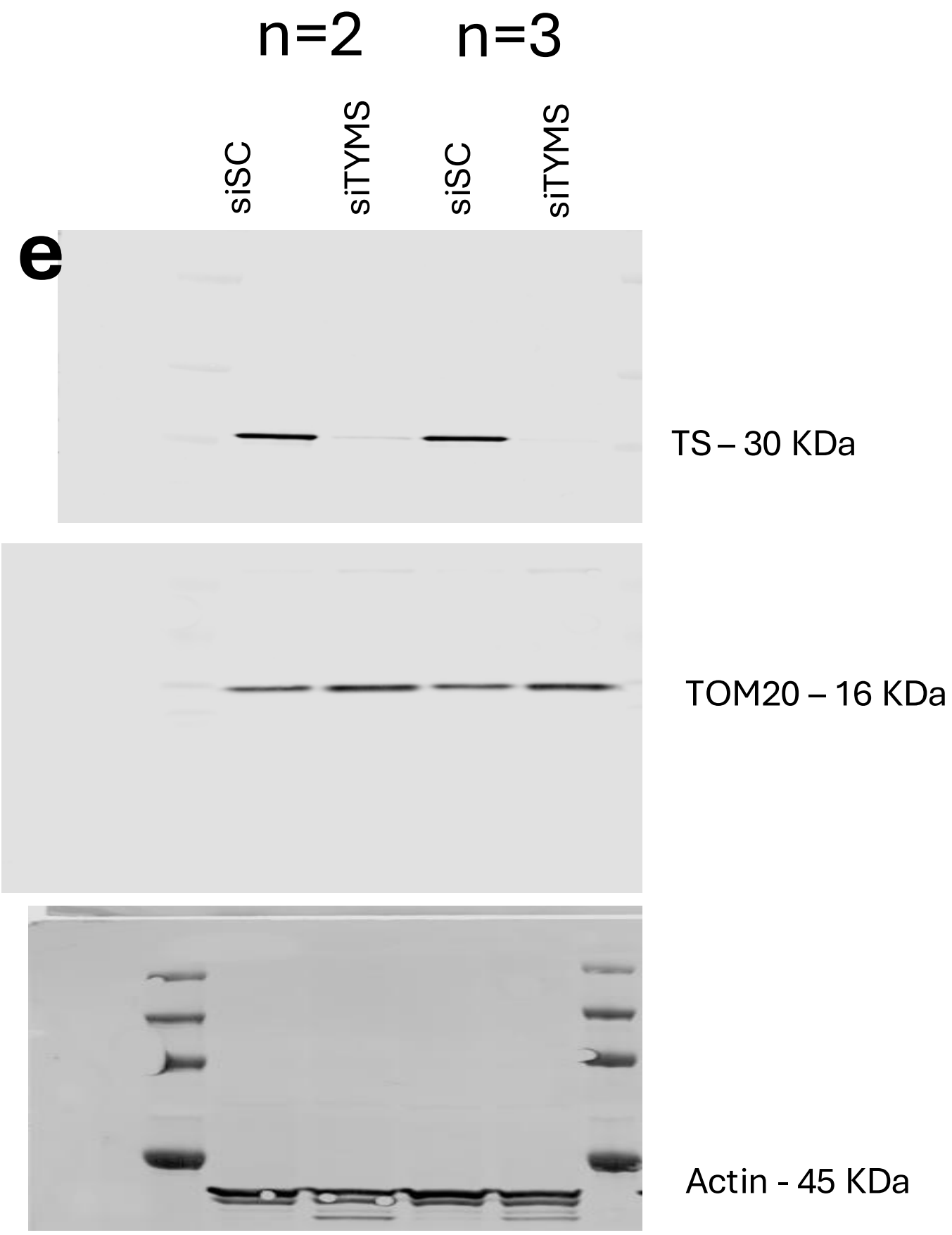

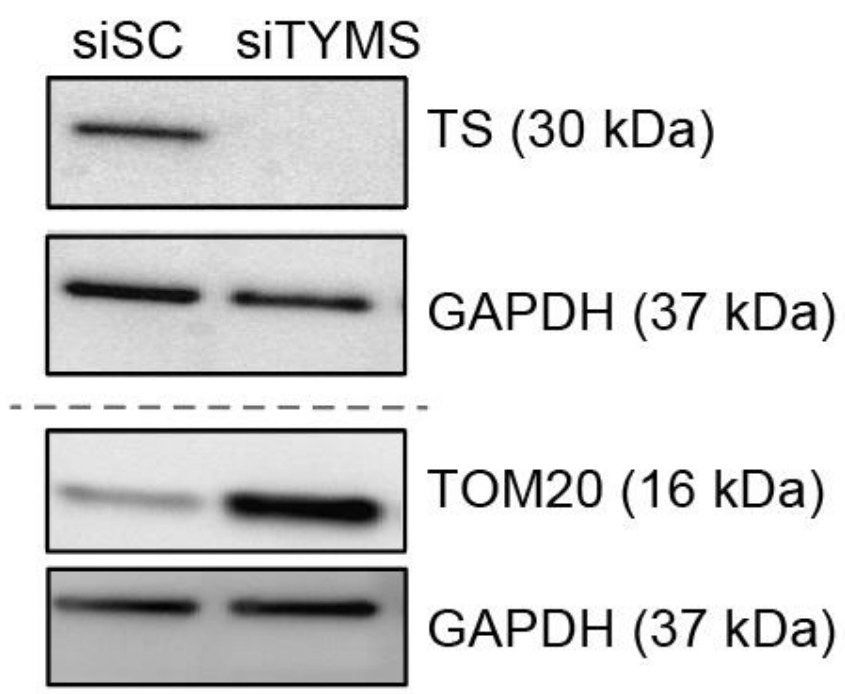

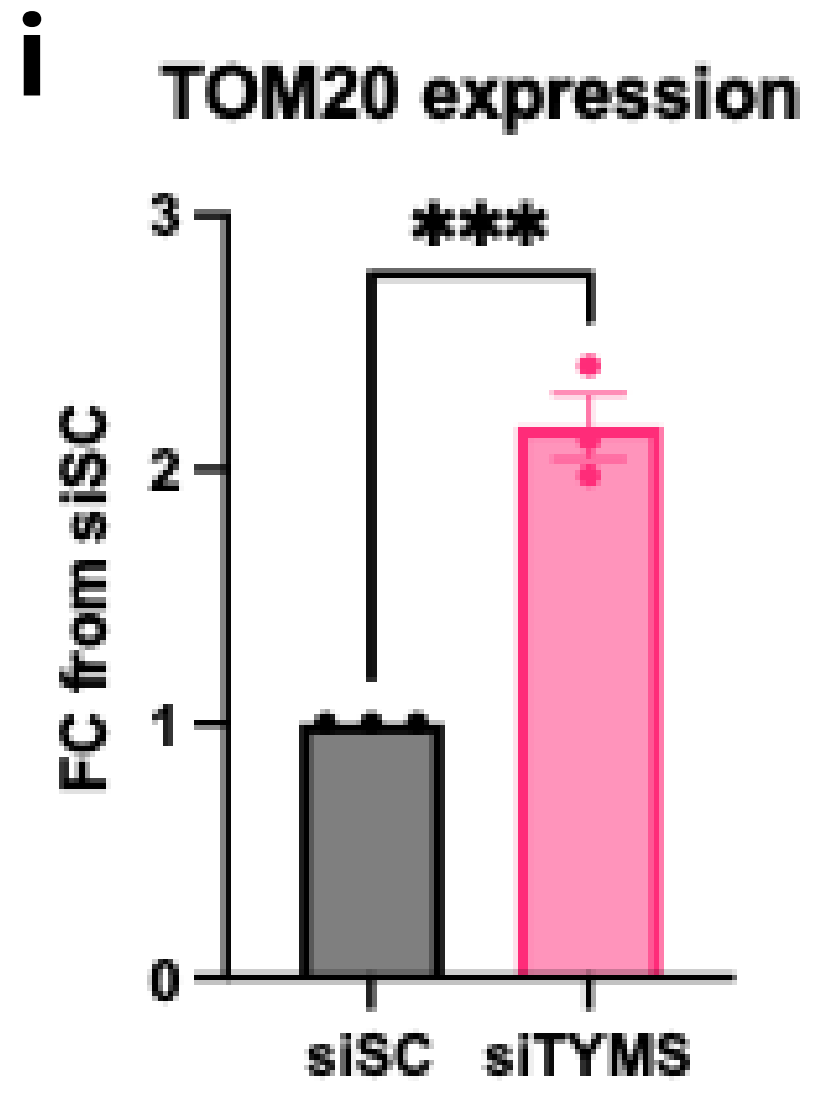

h

	SC	TYMS 10nM		SC	TYMS 20nM		SC	TYMS 40nM**		SC	TYMS		SC	TYMS
TOM20	2212.062	6881.79		2808.426	5460.548		1807.305	1957.77		12896.518	22225.317		12165.004	20878.539
GAPDH	3557.891	4594.598		5774.305	5277.305		6851.305	6052.305		19755.711	16079.125		21037.64	18259.69
TOM20/GAPI	0.62173406	1.49780024		0.48636606	1.03472284		0.26378989	0.32347511		0.65279949	1.38224667		0.57824946	1.14342242
		2.40906901			2.12745689			1.22626043			2.11741384			1.97738606
**40nM siRNA for TYMS resulted in significant cell death, so proteins not to be considered reliable**														
	Summary of FC over siSC													
		2.40906901	2.12745689	1.22626043	2.11741384	1.97738606								

Blots show Western images for independent biological repeats of HCT116 cells transfected with siRNA against Scrambled Control(SC) or *TYMS* at indicated concentrations for 72 hrs for TS (a, e), TOM20 (c, f) with GAPDH (b,d) and Actin (g) used as loading control. Size is indicated. n indicates biological replicate. Table (h) shows densitometry values and graph (i) shows replicate values expressed as FC over SC ± sem, with stats by t-test, red values not at same concentration therefore not plotted.

Figure 3e

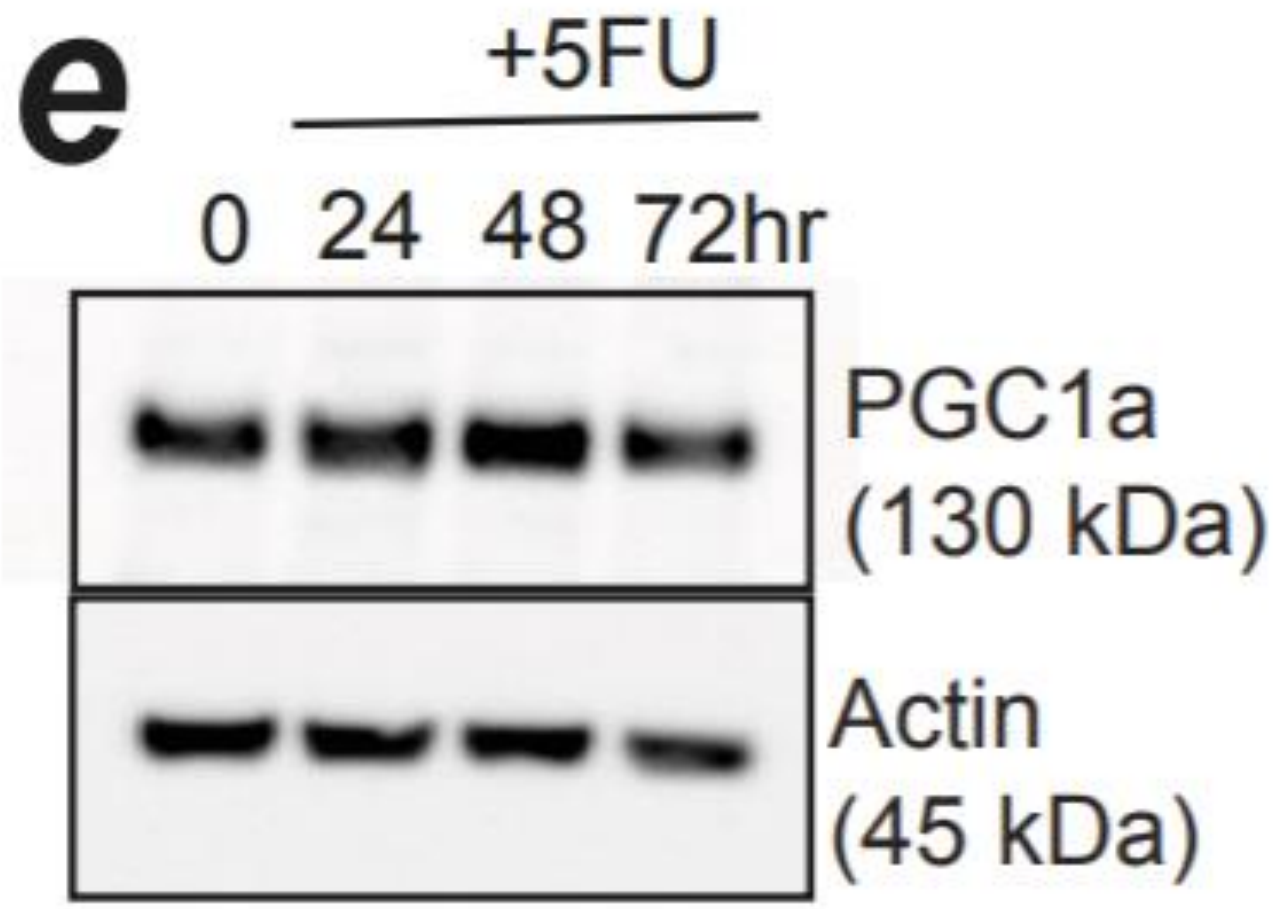

WB Observation 5
PGC1a increases over time with 5FU treatment

Four independent experiments for each timepoint, 0, 24, 48, 72hr 5uM 5FU

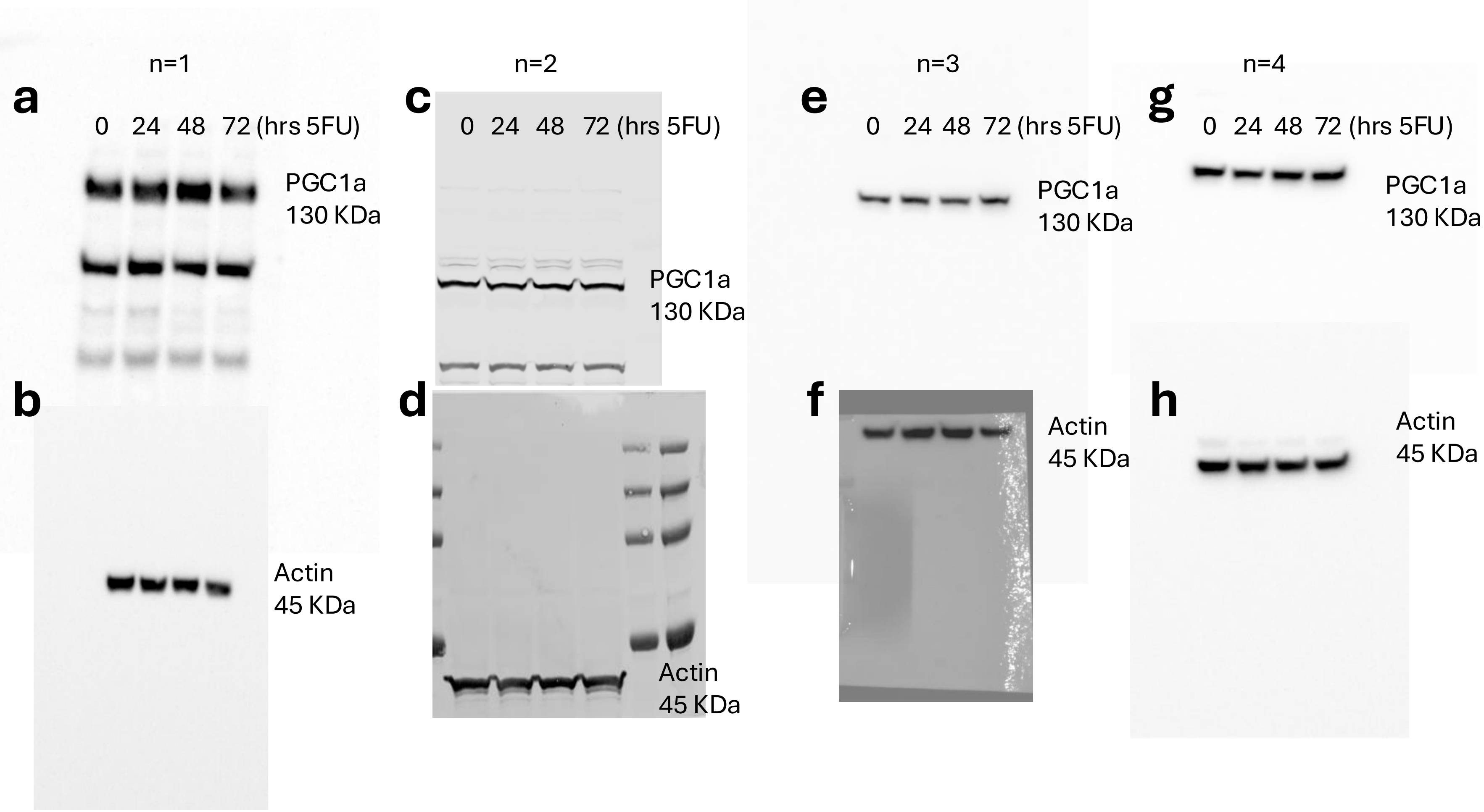

i

n=1	R3	PGC1a	Actin	PGC1a/Actin	
	0	10478.426	10304.648	1.01686404	
	24	11426.79	8874.77	1.287559	1.26620566
	48	12547.305	9140.77	1.37267484	1.34990991
	72	10236.669	7786.062	1.31474281	1.29293864
n=2	R4	PGC1a	Actin	PGC1a/Actin	
	0	13372.669	16495.376	0.81069198	
	24	13995.326	13665.447	1.02413964	1.26329071
	48	14858.912	14374.447	1.03370321	1.2750875
	72	15888.134	15180.64	1.04660502	1.29100207
n=3	R5	PGC1a	Actin	PGC1a/Actin	
	0	7449.77	8692.284	0.85705552	
	24	8466.113	11562.77	0.73218727	0.85430553
	48	8505.406	10975.698	0.77493076	0.90417802
	72	9212.477	6485.062	1.42056884	1.6574992
n=4	R6	PGC1a	Actin	PGC1a/Actin	
	0	9376.77	10259.113	0.91399422	
	24	7504.648	9943.355	0.75474002	0.82576017
	48	10005.234	8988.77	1.11308155	1.21782121
	72	10855.355	9372.477	1.15821623	1.26720301

j

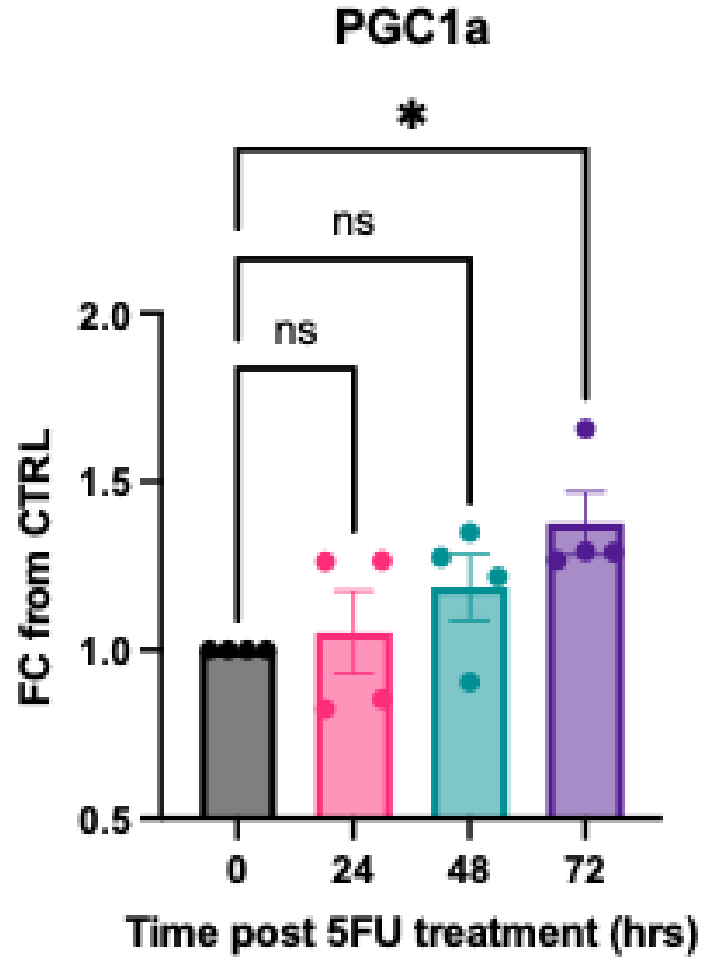

Blots show western images for 4 independent biological repeats of HCT116 cells treated with 5uM 5FU over 72 hrs for PGC1a (a,c,e,g) and Actin (b,), used as loading control. Size is indicated. n indicates biological replicate. Table (c) shows densitometry values and graph (d) shows replicate values expressed as FC over CTRL (0hr) ± sem, *p<0.05 determined by Dunnett’s post test following one-way ANOVA. Test for linear trend p = 0.0093.

Figure 3g

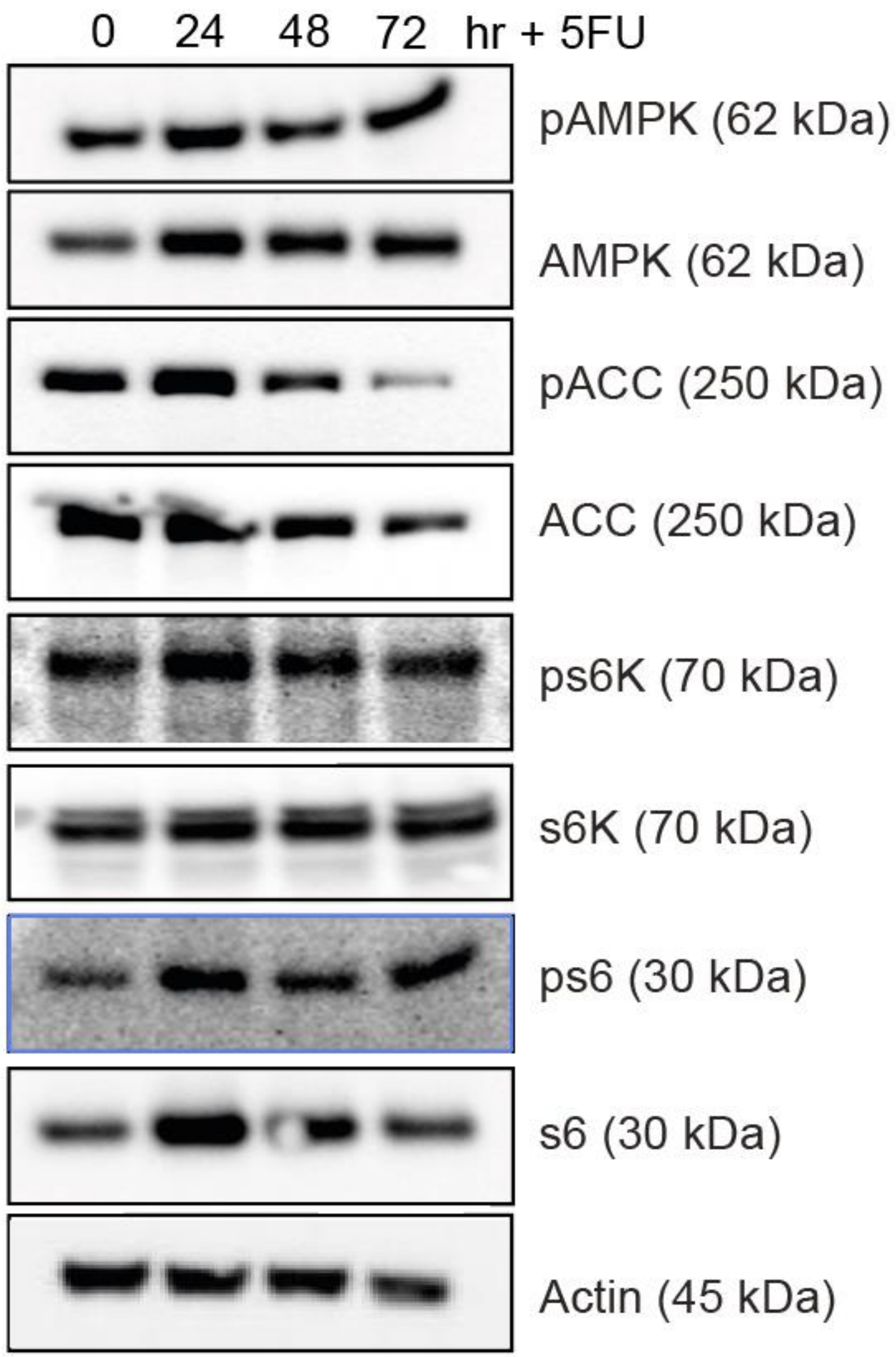

WB Observation 6
Changes in mTOR signalling with 5FU treatment

Blots used for figure: HCT116 0, 24, 48, 72hr 5uM 5FU

n=1

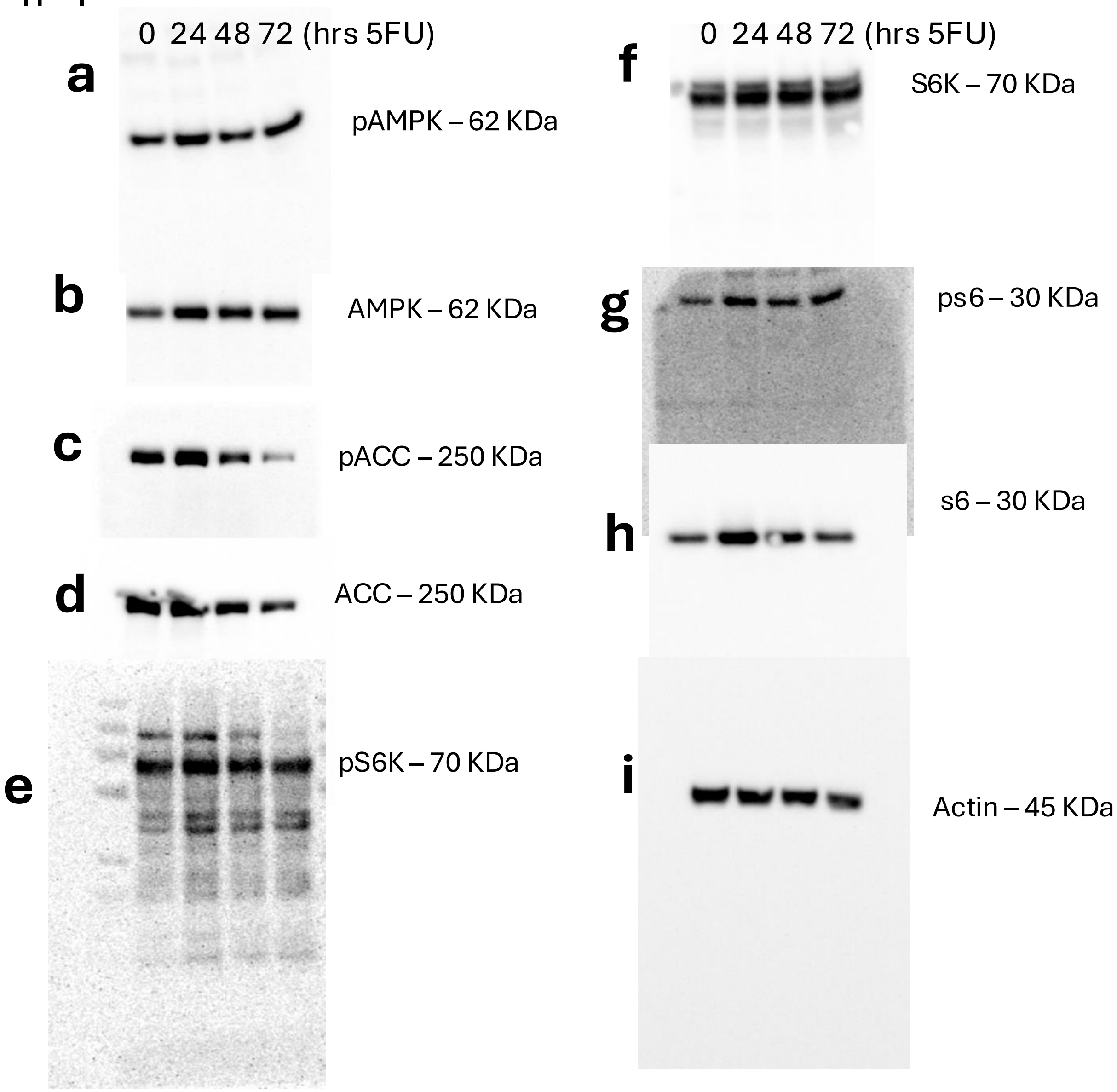

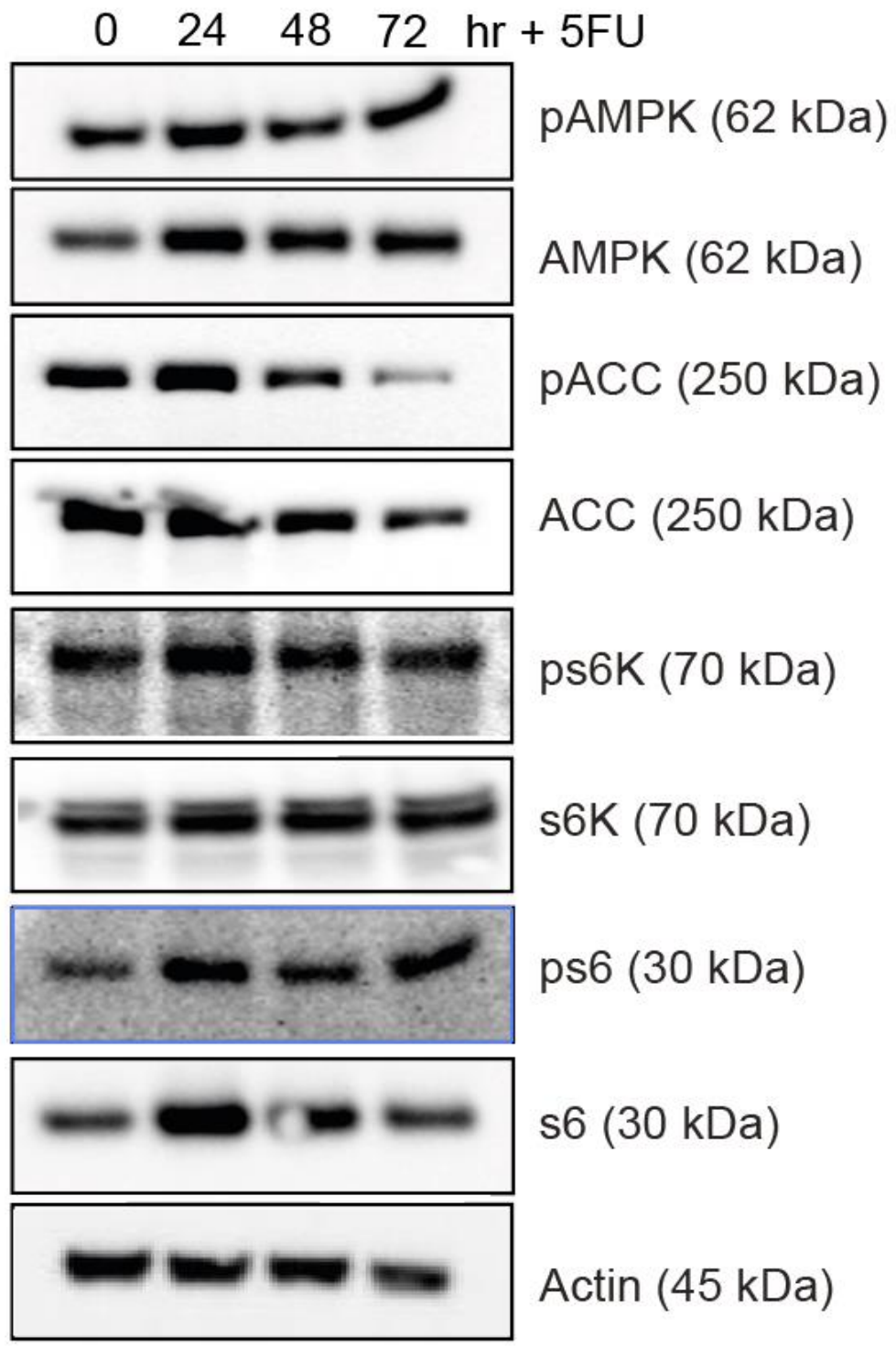

Blots show western images for HCT116 cells treated with 5uM 5FU over 72 hrs for pAMPK (a), AMPK (b), pACC (c), ACC (d), pS6K (e), S6K (f), pS6 (g), S6 (h), and Actin (i), used as loading control. Size is indicated. n indicates biological replicate.

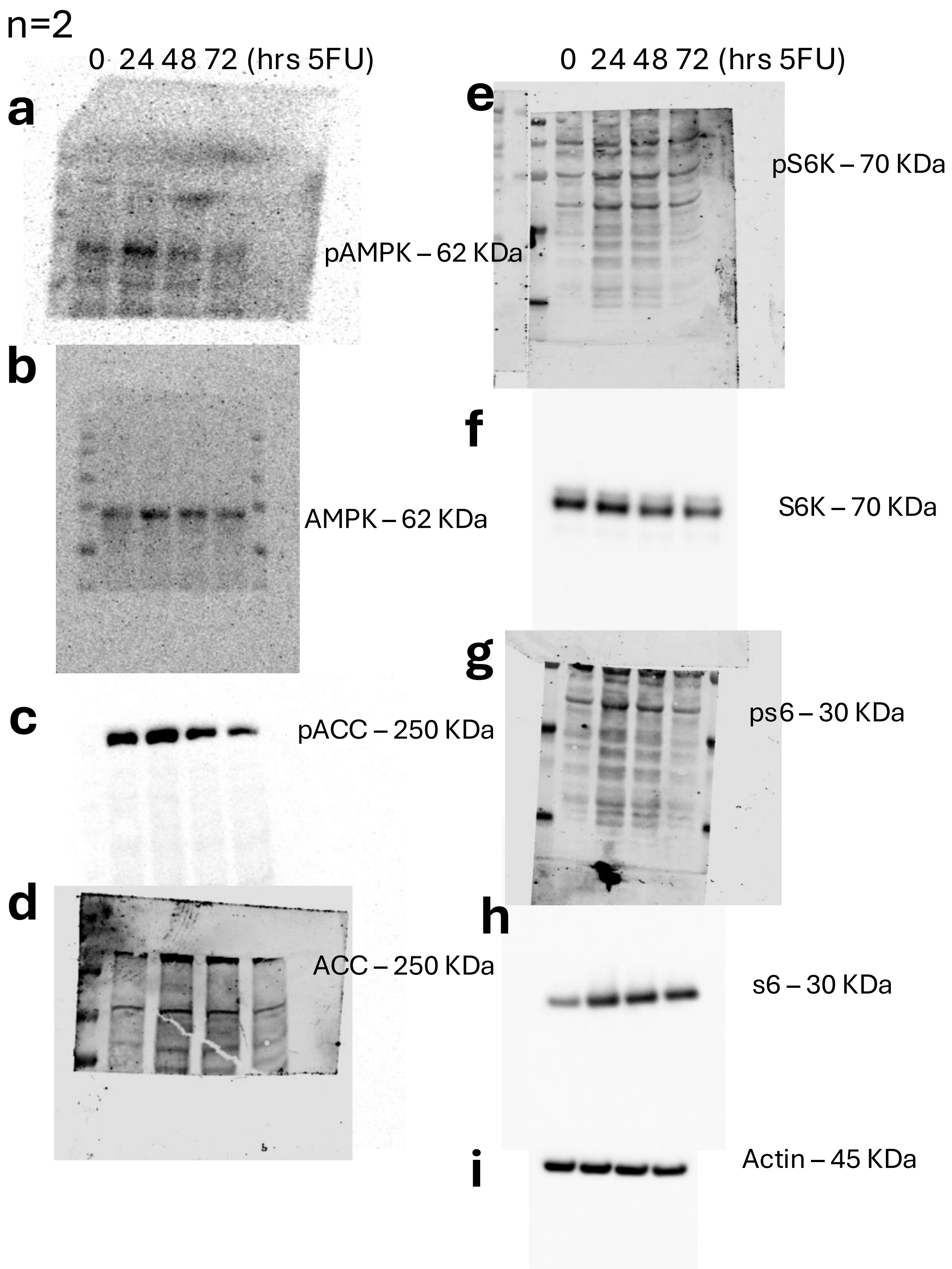

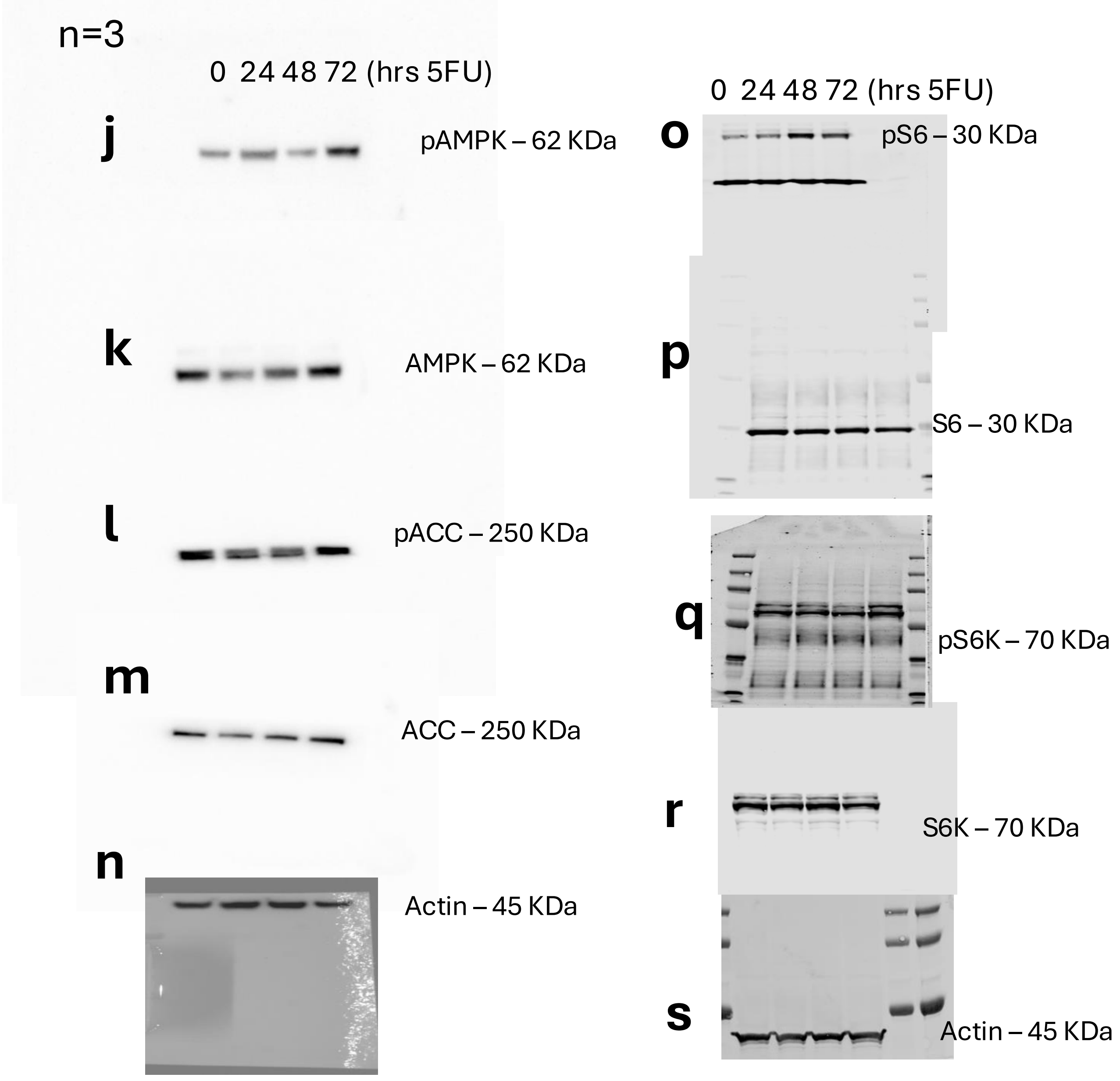

Blots show western images for HCT116 cells treated with 5uM 5FU over 72 hrs for pAMPK (a,j), AMPK (b,k), pACC (c,l), ACC (d,m), pS6K (e,q), S6K (f,r), pS6 (g,o), S6 (h,p), and Actin (I,n,s), used as loading control. Size is indicated. n indicates biological replicate.

Densitometry of all data presented

n1	pAMPK	tAMPK	pACC	tACC	pS6K	tS6K	pS6	tS6	Actin	
0hr	4134.719	4498.77	5535.305	8771.376	5030.406	6669.891	2216.991	3084.355	5901.891	
24hr	5477.962	5573.062	6698.891	8242.497	7351.991	7802.305	5094.648	5911.77	5428.598	
48hr	4150.134	4925.355	3937.477	5949.841	5816.698	7775.891	4084.355	3711.891	5330.184	
72hr	4366.426	4997.648	1549.113	3775.184	5400.991	8002.305	5217.648	3288.184	4490.305	
0hr	0.70057529	0.76225908	0.93788669	1.48619756	0.852338	1.13012778	0.37564079	0.52260453	1	
24hr	1.00909332	1.02661166	1.2340002	1.51834728	1.3543075	1.43725968	0.9384832	1.08900493	1	
48hr	0.77860989	0.92404971	0.73871315	1.11625434	1.09127527	1.45884101	0.76626904	0.69639078	1	
72hr	0.9724119	1.11298631	0.3449906	0.84074111	1.20281161	1.7821295	1.16198076	0.73228522	1	
24hr	1.44037813	1.34680148	1.31572418	1.0216322	1.58893244	1.2717674	2.49835273	2.08380307		
48hr	1.11138646	1.2122515	0.78763582	0.75108072	1.28033159	1.29086377	2.03989839	1.33253873		
72hr	1.38801912	1.46011552	0.36783825	0.56569943	1.41119087	1.57692743	3.09332955	1.40122248		
n2	pAMPK	tAMPK	pACC	tACC	pS6K	tS6K	pS6	tS6	Actin	
0hr	6426.719	4868.113	5250.648	1131.799	1720.577	4968.163	1631.092	2443.577	4604.284	
24hr	8470.426	5555.991	6256.062	3142.799	5589.991	5508.163	4603.506	5128.284	4765.577	
48hr	4670.477	4677.577	4232.82	2862.092	5263.284	4653.87	3273.92	4887.991	4702.577	
72hr	3257.355	3799.456	2682.87	1767.556	4882.991	5031.577	2482.627	4035.87	4272.87	
0hr	1.3958129	1.05730077	1.14038317	0.24581433	0.37369046	1.07903053	0.3542553	0.53071813	1	
24hr	1.77741877	1.16585903	1.31276066	0.65947922	1.17299353	1.15582289	0.96599132	1.07610978	1	
48hr	0.99317396	0.99468377	0.90010647	0.60862204	1.11923399	0.98964249	0.696197	1.03942817	1	
72hr	0.76233422	0.88920468	0.62788477	0.4136695	1.14278951	1.17756379	0.58102095	0.94453377	1	
24hr	1.27339328	1.10267492	1.15115751	2.68283469	3.13894429	1.07116793	2.7268225	2.02764841		
48hr	0.71153803	0.94077655	0.78930178	2.47594203	2.99508312	0.91715893	1.9652409	1.95853148		
72hr	0.54615788	0.84101394	0.55059105	1.68285346	3.05811797	1.09131647	1.64011931	1.77972772		
n3	pAMPK	tAMPK	pACC	tACC	pS6K	tS6K	pS6	tS6	Actin1	Actin2
0hr	4423.941	8532.062	6156.042	4926.456	10491.79	11799.426	4483.719	15691.497	9182.82	14102.79
24hr	7417.527	5190.941	4894.335	3448.456	9027.79	10089.012	5242.012	12284.426	11052.598	11089.255
48hr	3685.355	7197.062	5241.456	4478.456	8839.669	12250.376	10628.841	11546.841	11126.82	11838.962
72hr	9915.012	9234.77	5569.749	6089.456	11800.205	10130.619	10063.426	10000.79	7024.477	11383.912
0hr	0.48176279	0.9291331	0.67038687	0.53648618	0.74395137	0.83667317	0.31793135	1.11265196	1	1
24hr	0.67111162	0.46965799	0.44282213	0.31200411	0.81410248	0.9098007	0.47271093	1.10777739	1	1
48hr	0.33121368	0.6468211	0.47106505	0.402492	0.74665912	1.03475085	0.89778487	0.97532546	1	1
72hr	1.41149469	1.31465588	0.79290586	0.86689102	1.03656854	0.88990665	0.8840042	0.87850205	1	1

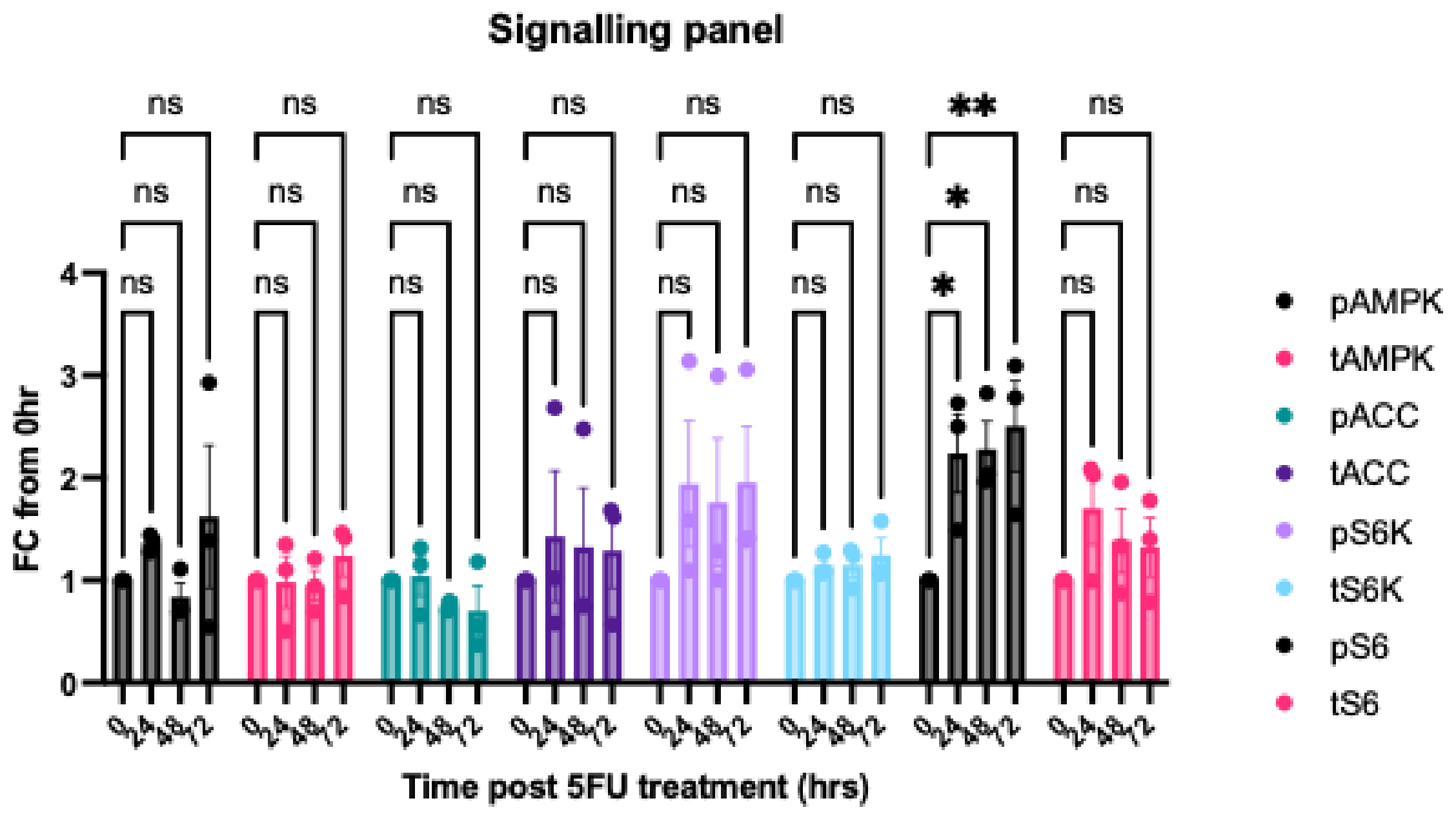

Table shows densitometry values for blots presented across slides 26-27, CTRL vs 5uM 5FU treated over 72hrs, with raw values then normalised to Actin and expressed as fold change over CTRL.

Graph shows expression fold changes as a function of CTRL (FC from CTRL) for each replicate ± sem, with statistical testing by two-way ANOVA with Dunnett post test relative to 0hr.

ED Figure 9b

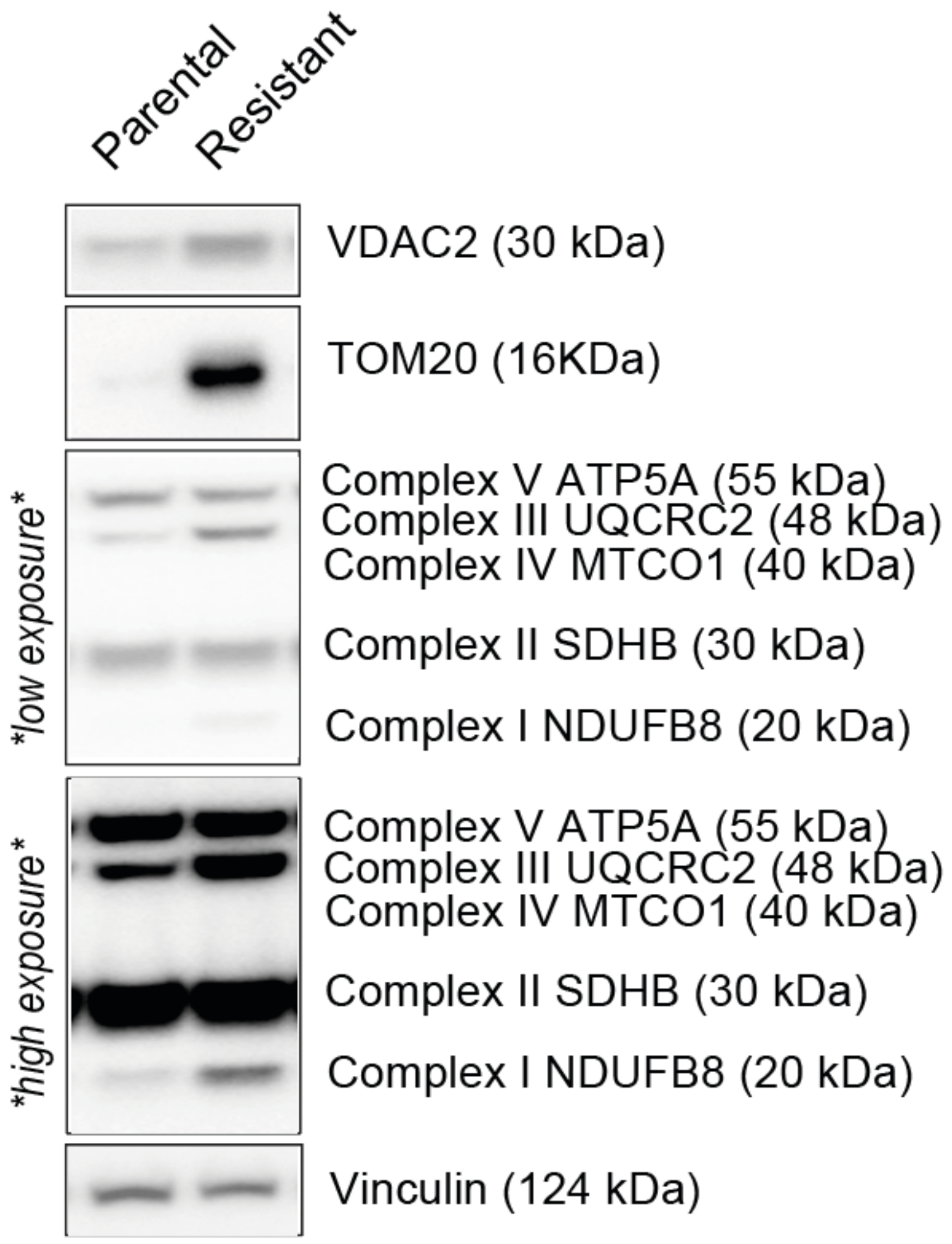

WB Observation 7

5FU resistant organoids show increased mitochondrial mass

Blots used for figure, three biological replicates of Parental (P) and Resistant (R) AKP organoids

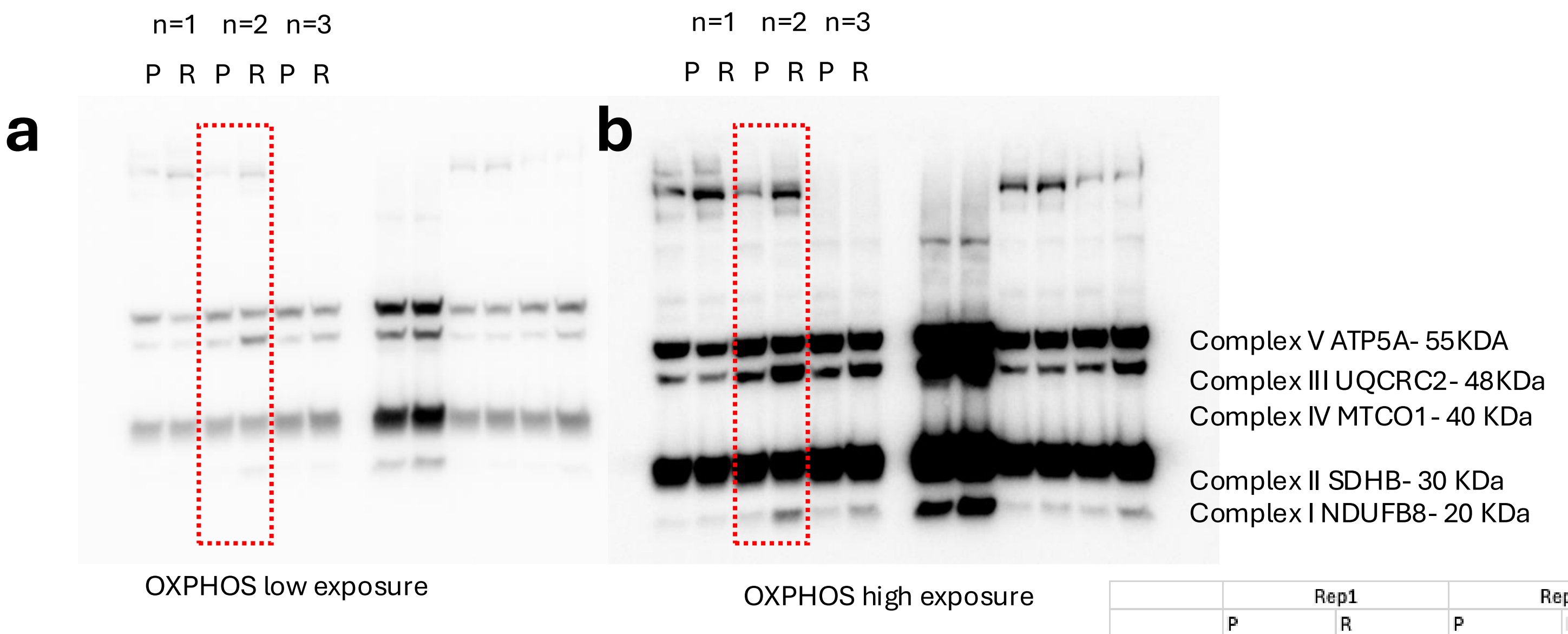

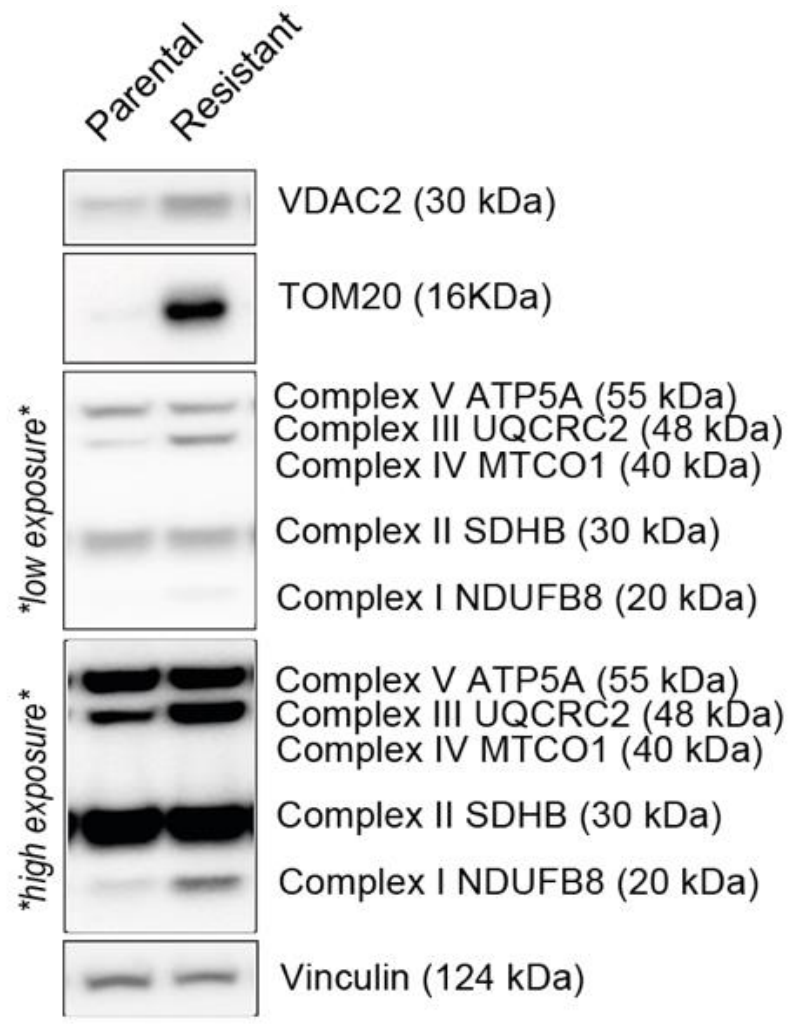

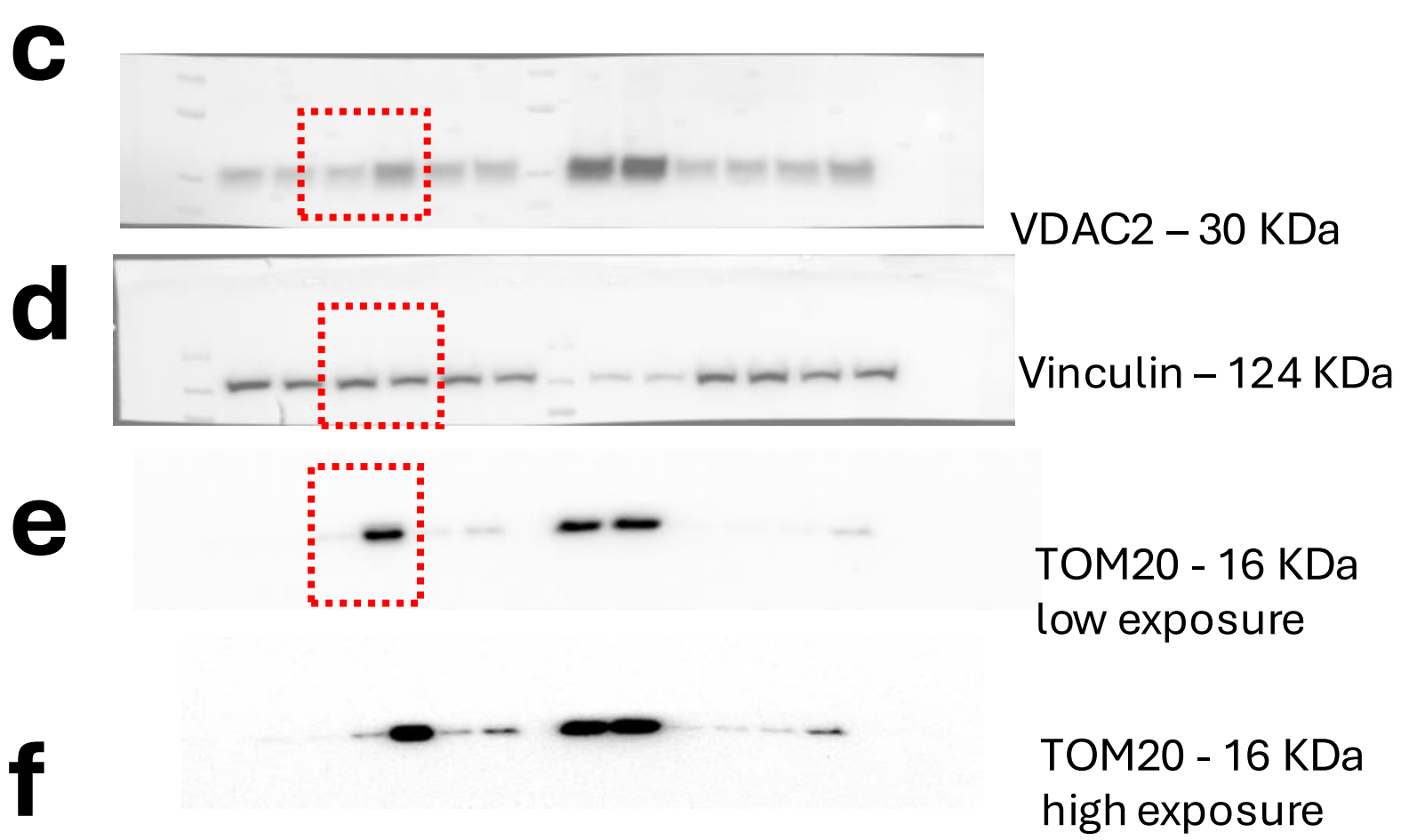

g

	Rep1		Rep2		Rep3	
	P	R	P	R	P	R
V	6741.184	5691.255	7866.012	8165.012	8788.012	8473.719
III	2681.941	2424.941	4550.941	7321.062	4531.77	5998.477
II	12208.397	12357.225	12791.104	13111.64	14948.619	15358.083
I	114.192	474.163	463.92	2191.284	804.87	1461.284
TOM20	90.192	212.607	1282.284	4362.355	1066.406	1707.335
VDAC2	3872.77	5194.77	3577.941	7291.184	3898.477	4079.941
VINCULIN	4763.87	3604.749	4002.042	3202.335	3153.627	2446.92
Norm to loading						
V	1.41506464	1.57882144	1.96549961	2.54970576	2.78663647	3.46301432
III	0.56297527	0.67270731	1.13715473	2.28616369	1.43700254	2.45143977
II	2.56270574	3.42804034	3.19614437	4.09439987	4.74013541	6.27649576
I	0.02397043	0.13153842	0.11592082	0.68427694	0.25522042	0.59719321
TOM20	0.01893251	0.0589797	0.32040743	1.36224193	0.33815223	0.6977486
VDAC2	0.8129462	1.44109063	0.89402885	2.27683362	1.23618836	1.66737817
FC from Parental						
V		1.1157239		1.29723036		1.24272196
III		1.19491449		2.01042446		1.70593976
II		1.33766444		1.28104347		1.3241174
I		5.48752933		5.90296831		2.33991156
TOM20		3.11526082		4.25159279		2.06341564
VDAC2		1.77267651		2.54671158		1.34880591

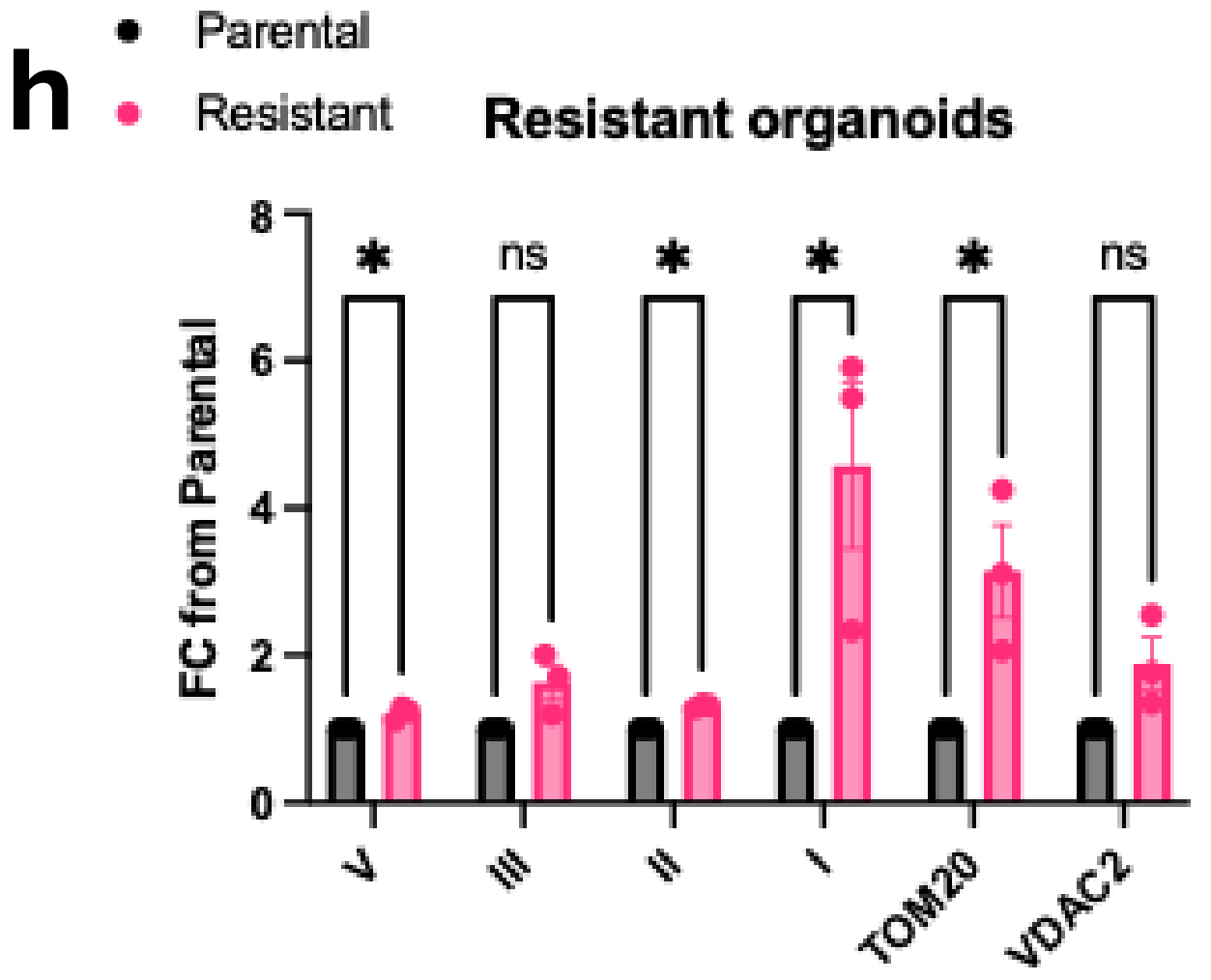

Blots show independent western images for Parental (P, 5FU sensitive) and Resistant (R, 5FU resistant) AKP organoids in 3D culture for electron transport chain panel (a, low exposure and b, high exposure), VDAC2 (c), Vinculin (d) used as loading control and TOM20 (e, low exposure and f, high exposure). Size is indicated. n indicates biological replicate. Table (g) shows densitometry values for blots presented, with raw values then normalised to Vinculin and expressed as fold change over P. Graph (h) shows expression fold change over P for each replicate ± sem, with statistical testing by t-test.

Figure 8d

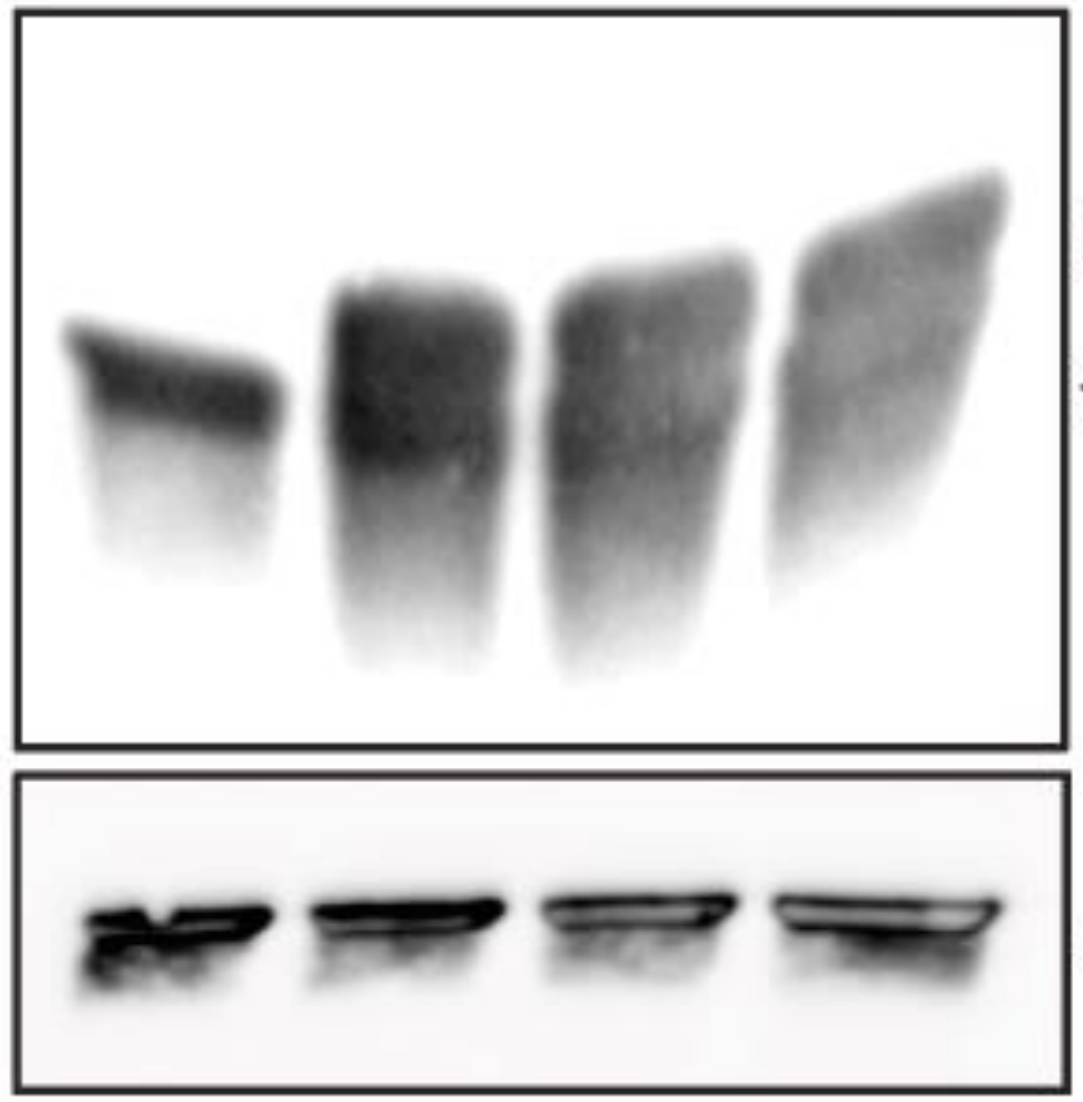

WB Observation 8
5FU promotes continued TS inhibition at 72hr evidenced by formation of ternary complex

Blots used for figure, 0, 24, 48, 72hr 5uM 5FU; Vinculin included as second reference loading given burn through.

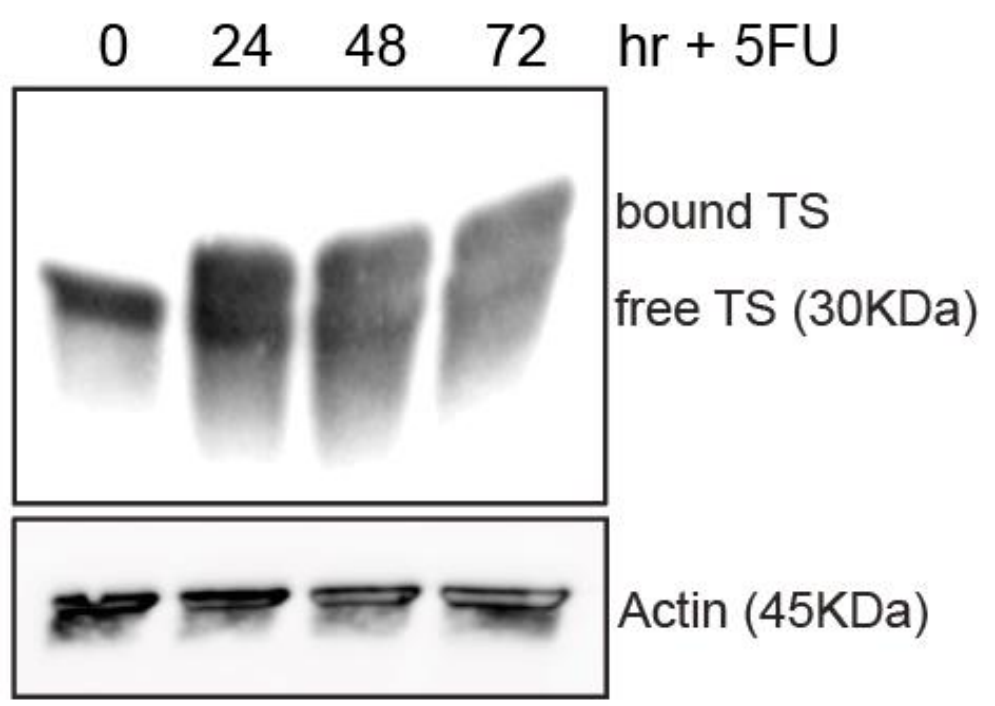

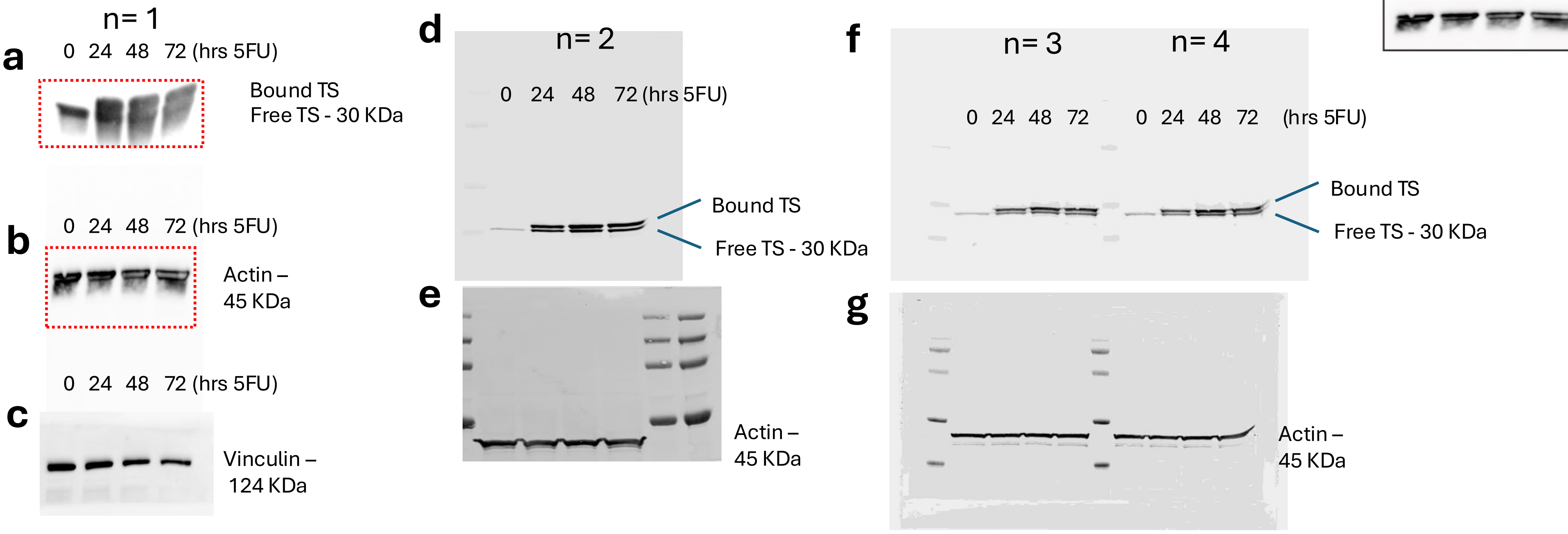

Blots show western images across biological replicates for HCT116 cells treated with 5uM 5FU over 72hrs for TS (a,d,f) with Actin (b,e,g) and Vinculin (c) used as loading. Free TS (lower band) and Bound TS (upper band) is indicated, with bound TS indicating TS inhibition.

Quantifying band shift on TS blot - data

a **Approach exemplar**

1: upper TS band

2: lower TS band

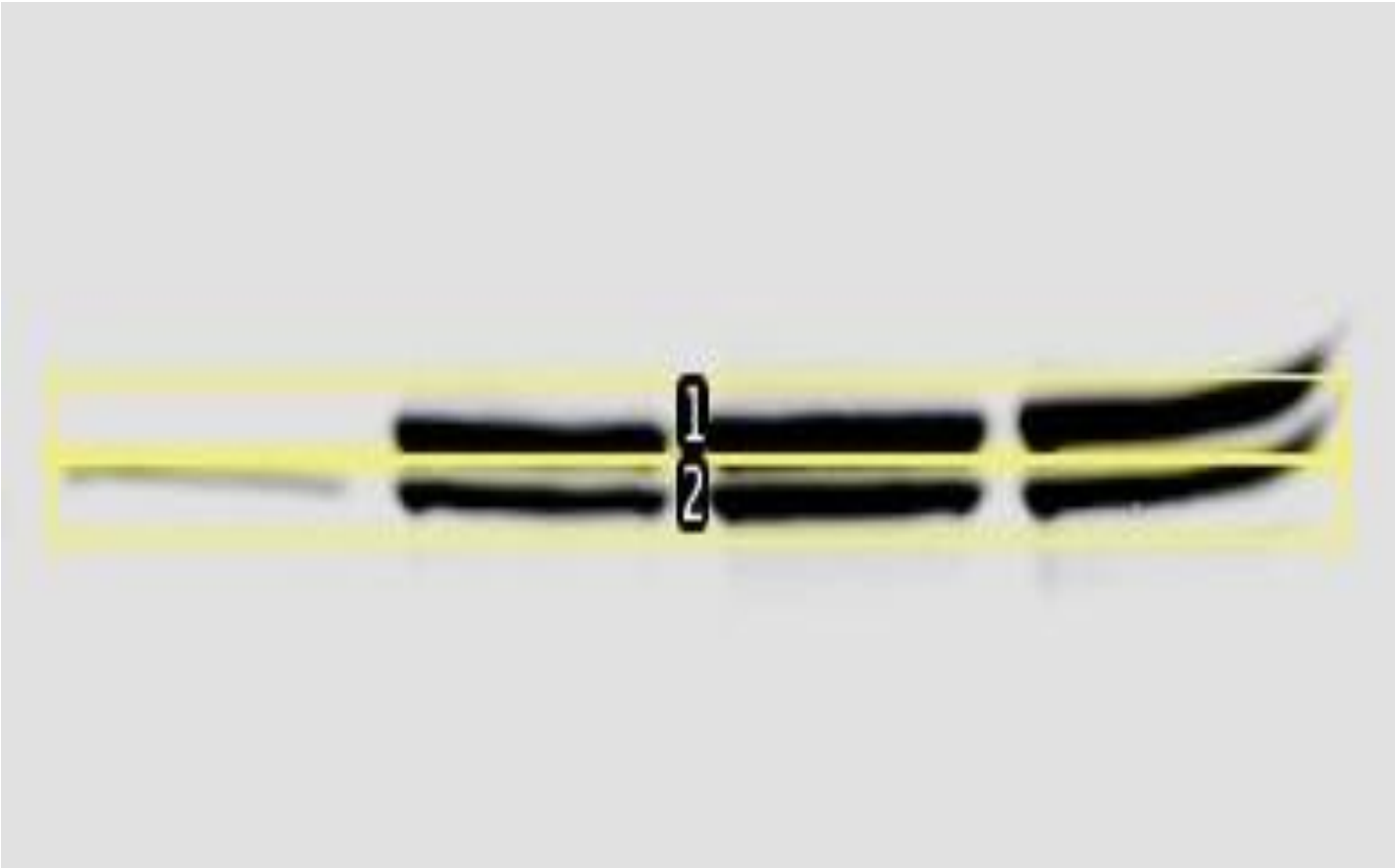

b

n=1 n=2 n=3 n=4

TS upper band

TS lower band

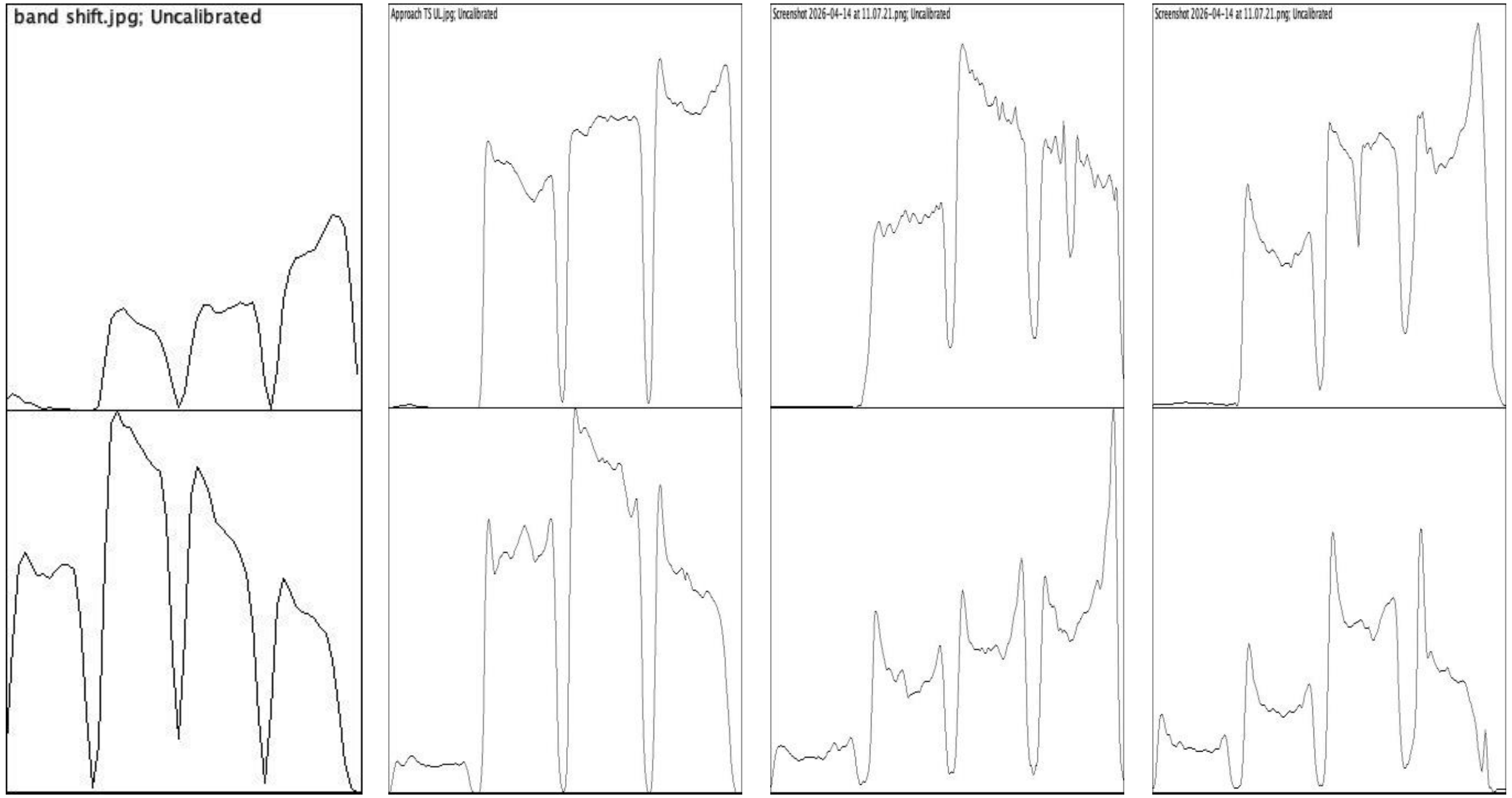

Densitomtry of blots shown in slide 35, quantifying upper and lower bands as shown (a, representative plot from blot n=2) alongside actin, with upper and lower bands quantified for each repeat (b, n=1-4).

Quantifying band shift on TS blot - data

a

Repeat 1		TS	Ratio U:L	Repeat 2		TS	Ratio U:L	Repeat 3		TS	Ratio U:L	Repeat 4		TS	Ratio U:L
	L	6954.94			L	3407.18			L	5311.69			L	5613.74	
0	U	119.19	0.017	0	U	0.00	0.000	0	U	0.00	0.000	0	U	0.00	0.000
	L	10114.89			L	27583.46			L	14486.05			L	11971.90	
24	U	2427.36	0.240	24	U	23089.37	0.837	24	U	22575.00	1.558	24	U	17046.15	1.424
	L	8352.43			L	36980.22			L	17695.39			L	24634.75	
48	U	3173.70	0.380	48	U	29550.64	0.799	48	U	35455.05	2.004	48	U	28024.17	1.138
	L	5170.60			L	24250.92			L	25097.41			L	16146.80	
72	U	5211.77	1.008	72	U	38891.85	1.604	72	U	30009.02	1.196	72	U	33222.65	2.058
		Actin				Actin				Actin				Actin	
	0	9002.18			0	37363.05			0	21911.69			0	18046.28	
	24	7881.31			24	31232.75			24	19514.03			24	16238.74	
	48	7101.65			48	33812.24			48	19255.79			48	18642.28	
	72	8998.43			72	35087.89			72	21827.74			72	18890.76	
		Total TS				Total TS				Total TS				Total TS	
	0	7074.13			0	3407.18			0	5311.69			0	5613.74	
	24	12542.25			24	50672.83			24	37061.04			24	29018.05	
	48	11526.12			48	66530.86			48	53150.43			48	52658.92	
	72	10382.37			72	63142.78			72	55106.43			72	49369.46	
		Total TS/actin				Total TS/actin				Total TS/actin				Total TS/actin	
	0	0.79			0	0.09			0	0.24			0	0.31	
	24	1.59			24	1.62			24	1.90			24	1.79	
	48	1.62			48	1.97			48	2.76			48	2.82	
	72	1.15			72	1.80			72	2.52			72	2.61	

b

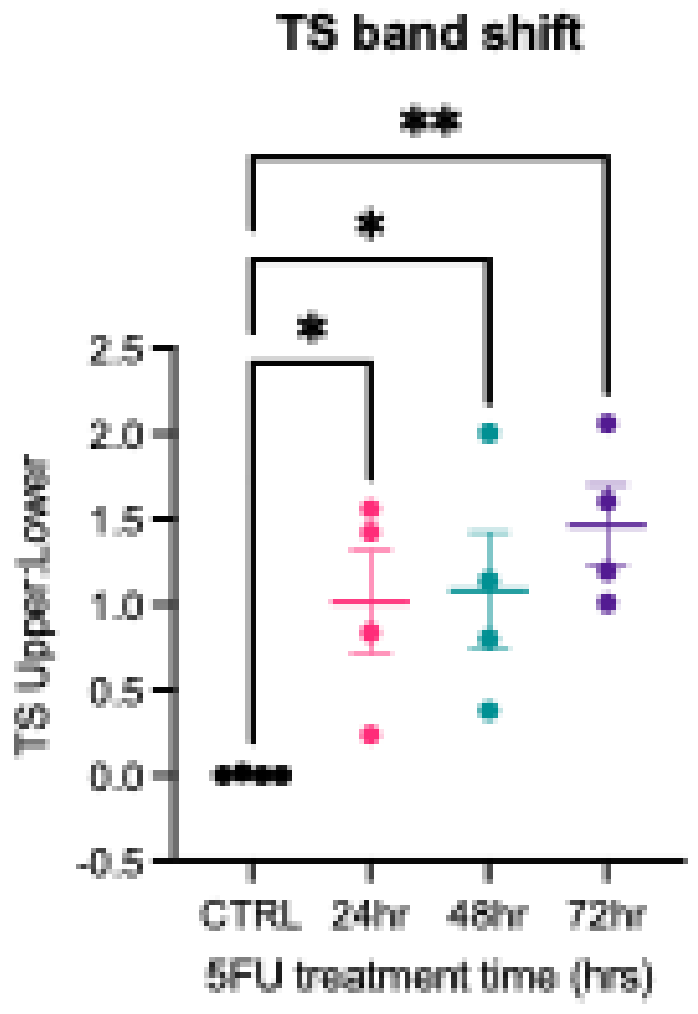

c

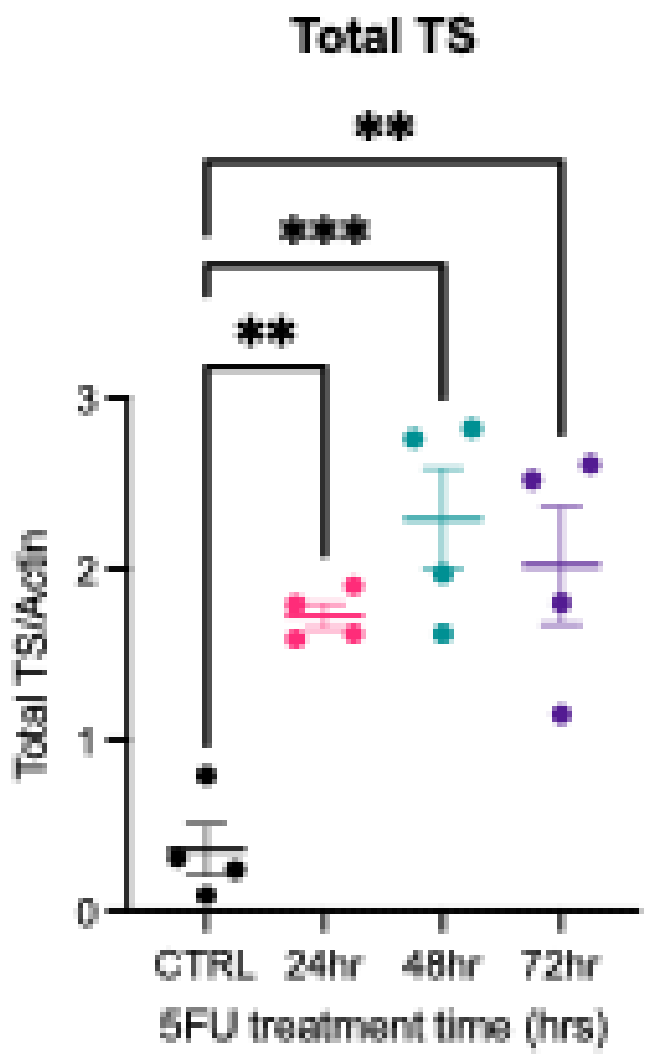

Densitometry data from blots shown in slide 35, quantified as shown in slide 36 (a), with data expressed as a ratio of TS upper (U): TS lower (L), or total TS (upper+lower) normalised to Actin for each repeat. Graphical representation of these data from n=4 repeats, for TS upper:lower ratio (b) and Total TS/Actin (c), with mean ± s.e.m. shown. Significance (indicated, *p<0.05, **p<0.01, ***p<0.001) relative to control (CTRL) by Dunnett's multiple comparisons test post one-way ANOVA. ANOVA summary p values were p=0.0104 (upper:lower) and p=0.0005 (Total TS), and linear test for trend is 0.0047 (upper:lower) and 0.0002 (Total TS).

Figure 8f

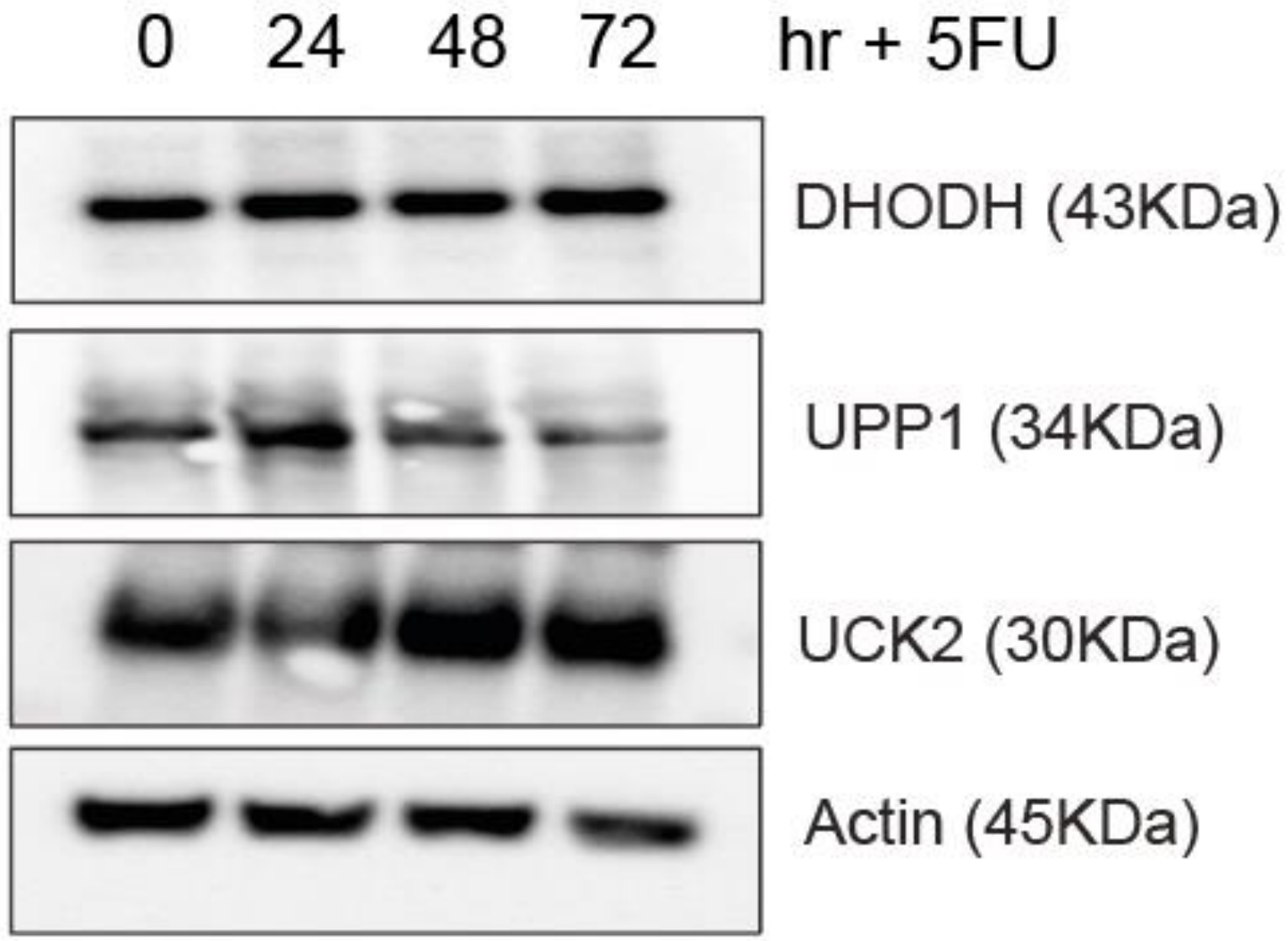

WB Observation 9
5FU increases UCK2 but not DHODH or UPP1 over time

Blots used for figure panel; 0, 24, 48, 72hr 5uM 5FU

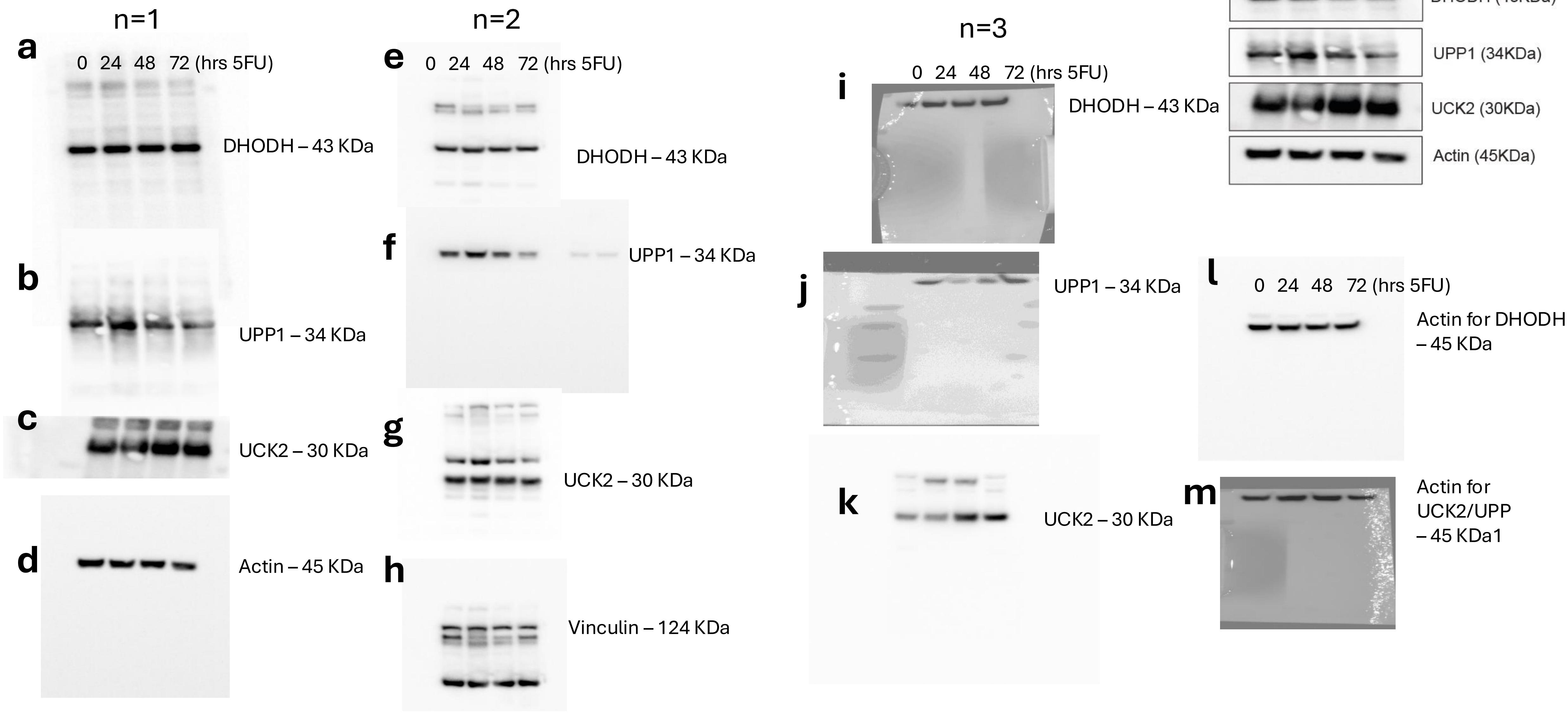

Blots show western images for HCT116 cells treated with 5uM 5FU over 72 hrs for DHODH (a,e,i), UPP1 (b,f,j), UCK2 (c,g,k) and Actin (d,l,m) or Vinculin (h), used as loading control. Size is indicated. n indicates biological replicate.

Densitometry of all data presented in slide 35

Rep1	DHODH	UPP1	UCK2	Loading
0hr	5707.698	6905.548	6454.941	5902.891
24hr	6211.991	9143.255	6563.648	5288.598
48hr	5754.284	5387.426	9378.648	5290.305
72hr	6344.991	3771.841	8390.648	4398.477
Rep2				
0hr	4991.184	4160.82	6600.012	3795.506
24hr	5400.598	5484.113	7136.012	4115.506
48hr	5117.305	3696.113	7070.012	3208.506
72hr	4610.77	1978.406	6780.719	2919.506
Rep3				
0hr	7283.82	13609.761	4596.406	10198.991
24hr	9231.355	5695.841	5855.527	10002.77
48hr	10034.477	9865.74	10249.305	9265.062
72hr	11842.305	18661.761	8828.527	9623.355

Rep1	DHODH	UPP1	UCK2
0hr	0.96693264	1.16985863	1.09352197
24hr	1.17460072	1.72886179	1.24109414
48hr	1.08770364	1.0183583	1.77279911
72hr	1.44254273	0.85753341	1.90762575
Rep2			
0hr	1.31502466	1.09624909	1.73890174
24hr	1.31225614	1.3325489	1.73393308
48hr	1.59491832	1.15197322	2.20352151
72hr	1.579298	0.67765095	2.32255697
Rep3			
0hr	0.71417065	1.3344223	0.45067262
24hr	0.92287986	0.56942637	0.58539055
48hr	1.08304478	1.06483259	1.10623167
72hr	1.23057967	1.93921569	0.91740635

Rep1	DHODH	UPP1	UCK2
0hr	1	1	1
24hr	1.21476995	1.47783821	1.13495126
48hr	1.12490115	0.87049689	1.62118289
72hr	1.49187509	0.73302311	1.74447867
Rep2			
0hr	1	1	1
24hr	0.9978947	1.21555303	0.99714264
48hr	1.2128429	1.05083163	1.2671915
72hr	1.20096455	0.61815417	1.33564589
Rep3			
0hr	1	1	1
24hr	1.29223998	0.42672126	1.29892636
48hr	1.51650699	0.79797272	2.45462364
72hr	1.72308912	1.45322488	2.03563809

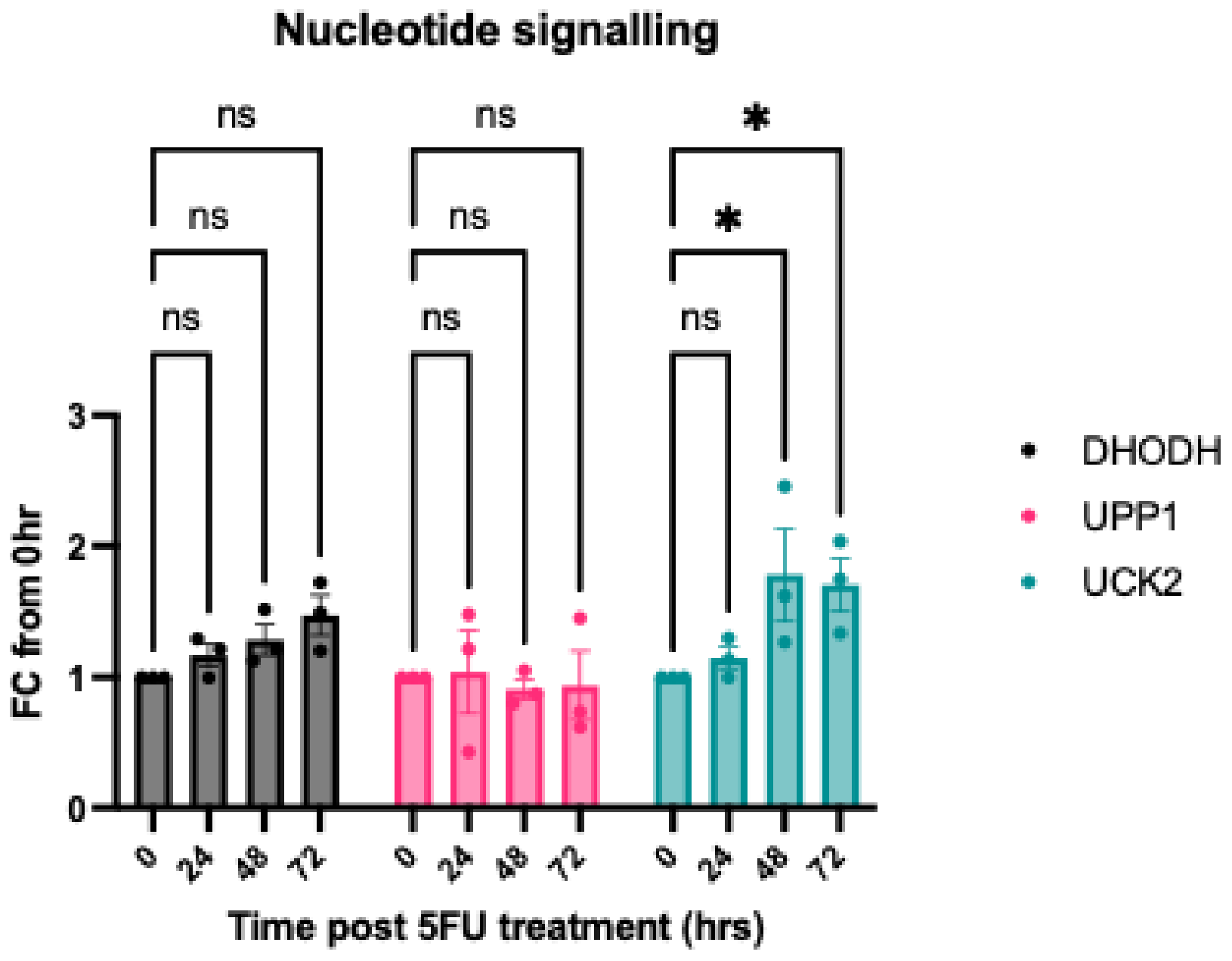

Table shows densitometry values for blots presented on slide 35, CTRL (0hr) vs 5uM 5FU treated over 72hrs, with raw values then normalised to Actin/Vinculin as loading and expressed as fold change over 0hr.

Graph shows expression fold changes as a function of CTRL (FC from CTRL) for each replicate ± sem, with statistical testing by two-way ANOVA with Dunnett post test relative to 0hr.

WB panels for **UCK2 densitometry quantification at 72hrs**, graph previously included as Fig 8i

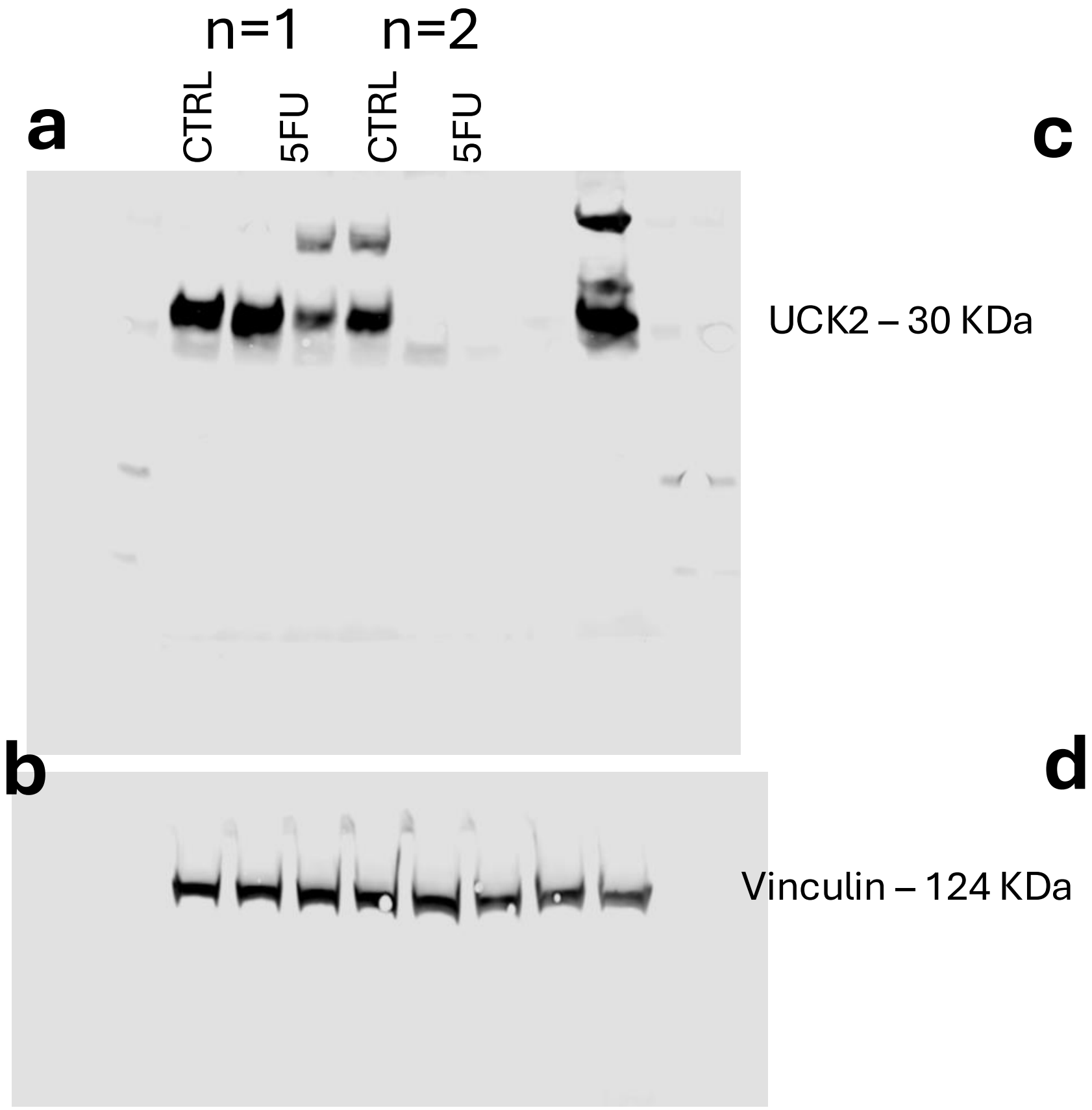

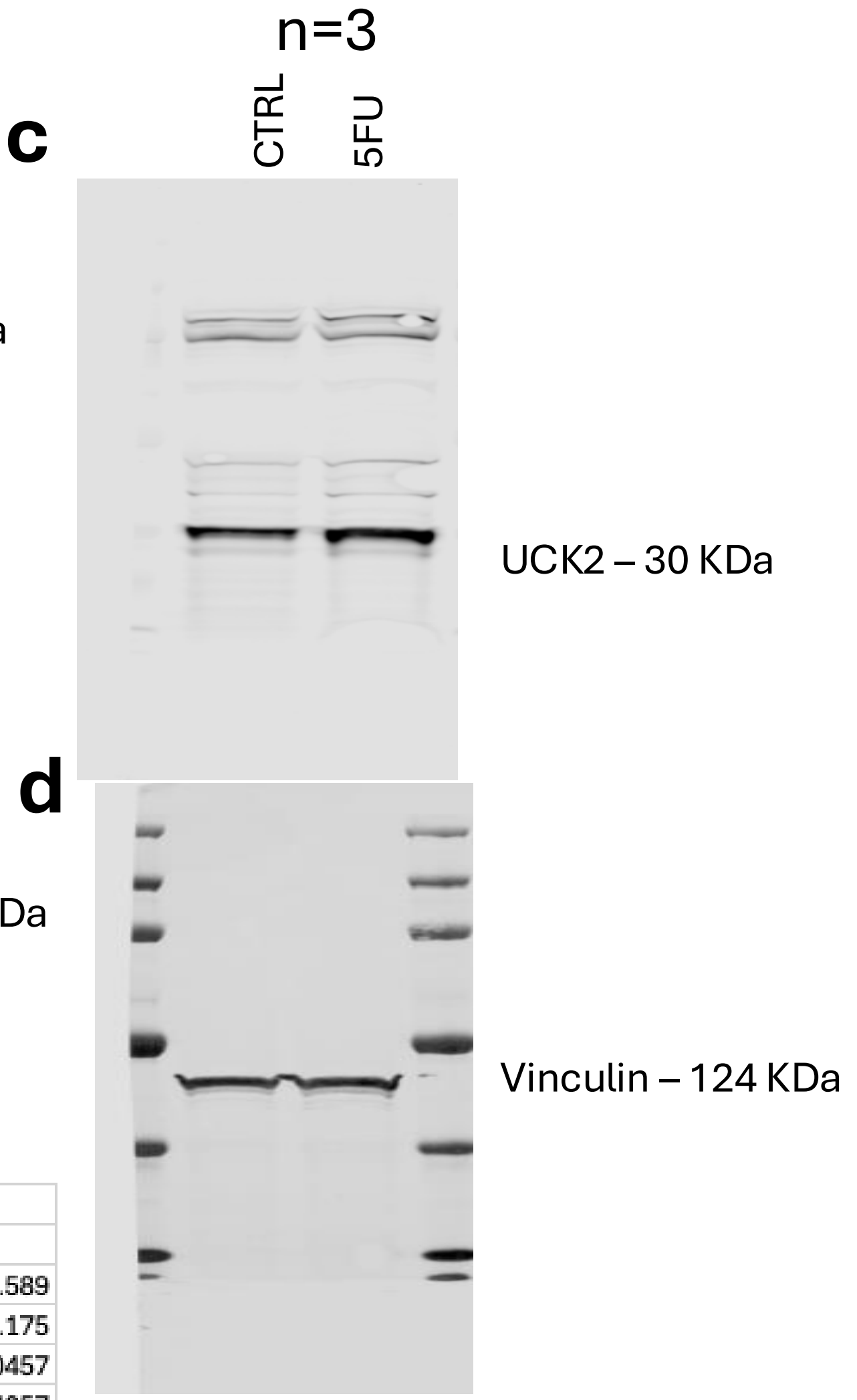

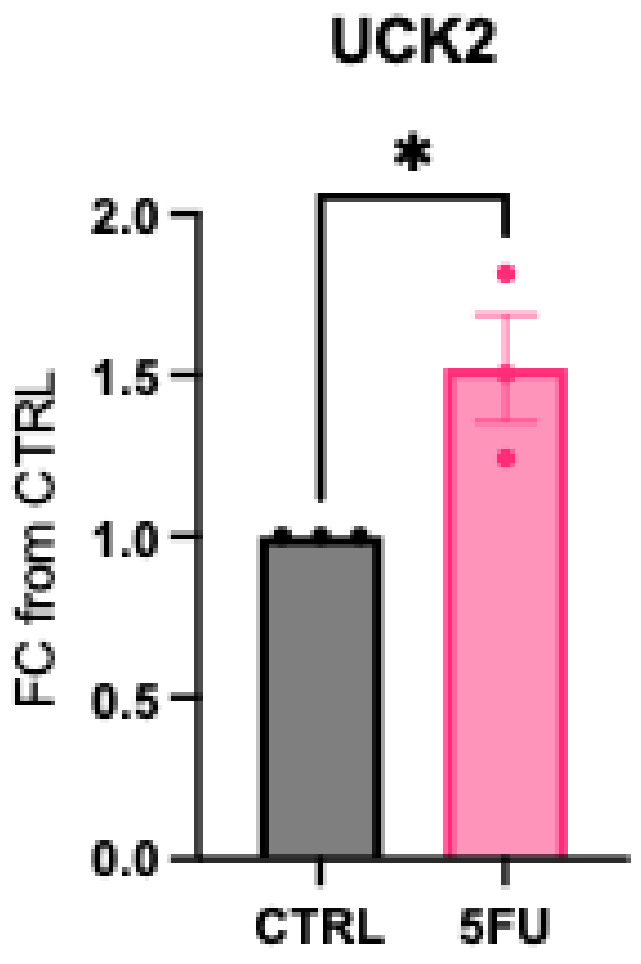

72hr additional treatments											
	R1	CTRL	5FU		R2	CTRL	5FU		R3	CTRL	5FU
	UCK2	10945.619	13994.497			5516.841	8925.305			13076.832	17351.589
	Loading	10063.891	10355.569			10883.719	9695.326			23571.66	20795.175
	UCK2/loadin	1.08761303	1.35139817			0.50688933	0.92057812			0.55476924	0.83440457
			1.24253584				1.81613238				1.504057

Blots show independent western images for HCT116 cells treated ± 5uM 5FU for 72hrs, blotted for UCK2 (a,c) and Vinculin (b,d) used as loading control. Size is indicated. n indicates biological replicate. Table shows densitometry values for blots presented, with raw values then normalised to Vinculin and expressed as fold change over CTRL. Graph shows expression fold change over CTRL for each replicate ± sem, with statistical testing by t-test.

Figure 8k

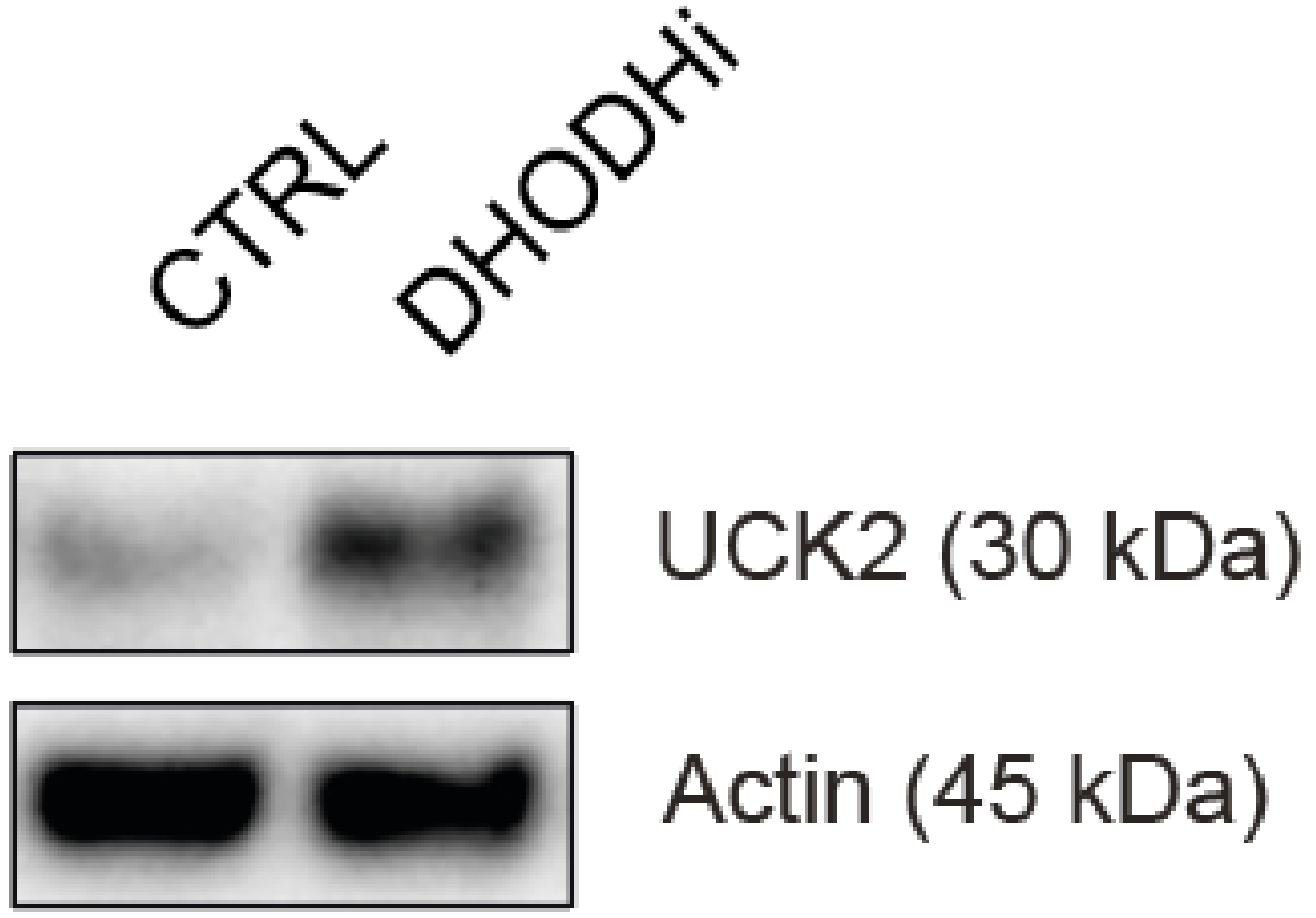

WB Observation 10
DHODHi treatment increases UCK2

Blots used for figure, n=6 biological replicates for CTRL vs DHODHi 72hrs

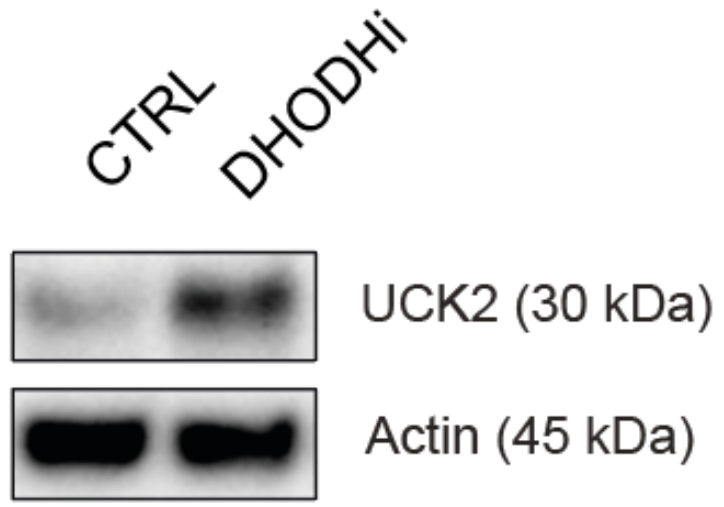

a

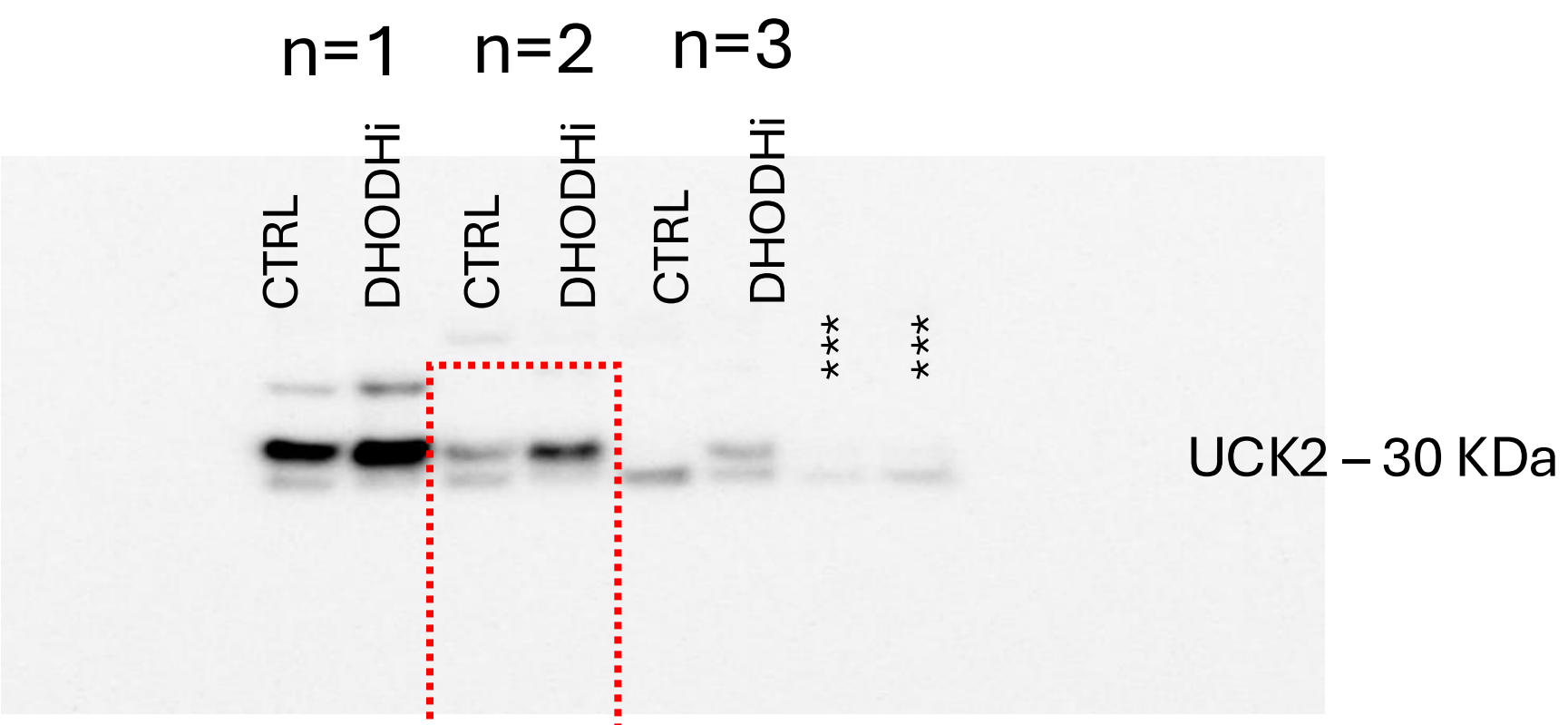

b

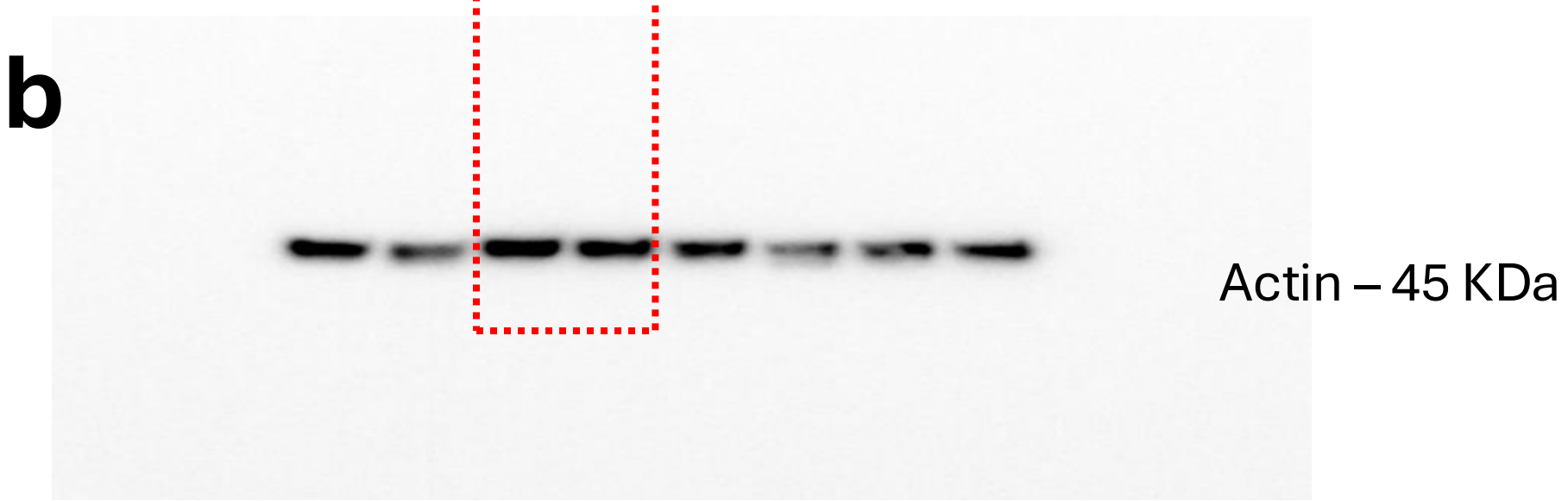

c

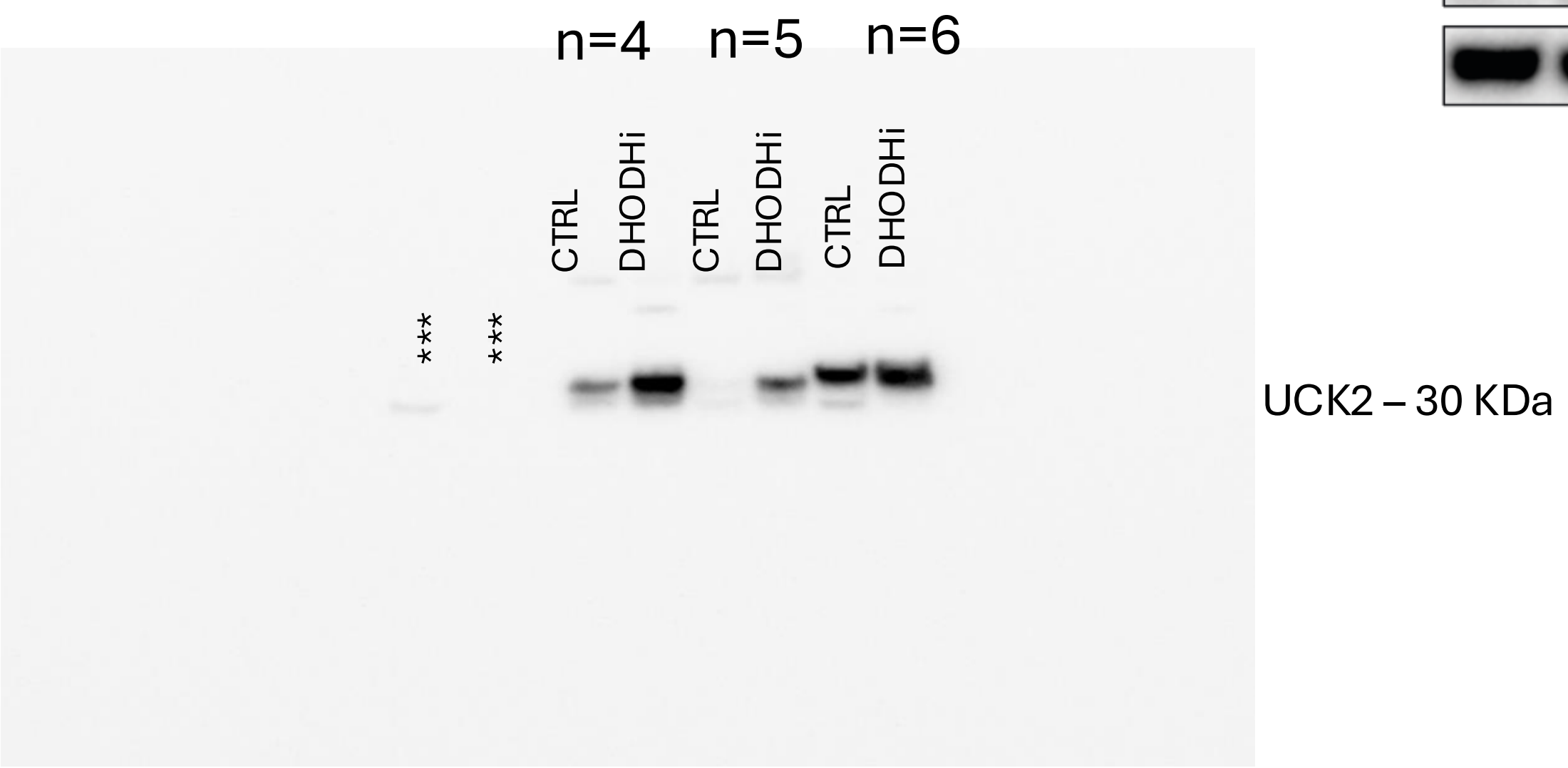

d

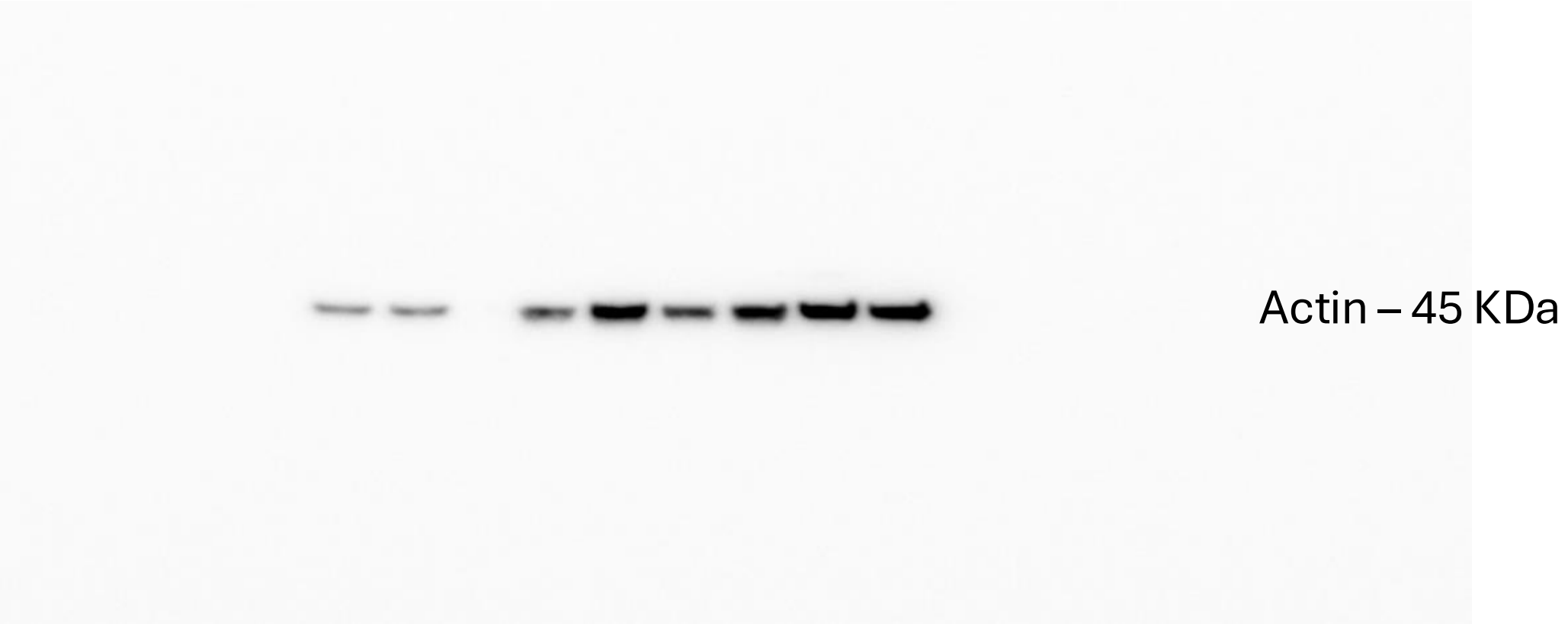

Blots show western images for HCT116 cells treated ± BAY-2402234 (DHODHi) for 72 hrs for UCK2 (a,c) and Actin (b,d) used as loading control. Size is indicated. n indicates biological replicate. *** experimental samples not related to these experiments also run on gel.

Densitometry of all DHODHi data presented on slide 39

Rep1	CTRL	BAY (DHODHi)		Rep2	CTRL	BAY (DHODHi)		Rep3	CTRL	BAY (DHODH
UCK2	7500.062	11769.527			2406.87	6290.669			729.021	1568.163
Loading	9146.891	6346.305			11454.355	9689.134			8595.841	5537.134
UCK2/loadin	0.81995751	1.85454796			0.21012707	0.64924987			0.0848109	0.28320843
FC over CTRL		2.26176104				3.0897965				3.33929282
Rep4	CTRL	BAY (DHODHi)		Rep5	CTRL	BAY (DHODHi)		Rep6	CTRL	BAY (DHODH
UCK2	3878.062	12322.719			566.728	5377.012			7497.234	11734.669
Loading	5272.134	11159.012			6310.598	10115.477			12398.598	12451.426
	0.73557728	1.10428405			0.08980575	0.53156287			0.60468401	0.94243575
FC over CTRL		1.50124818				5.91902922				1.55855906

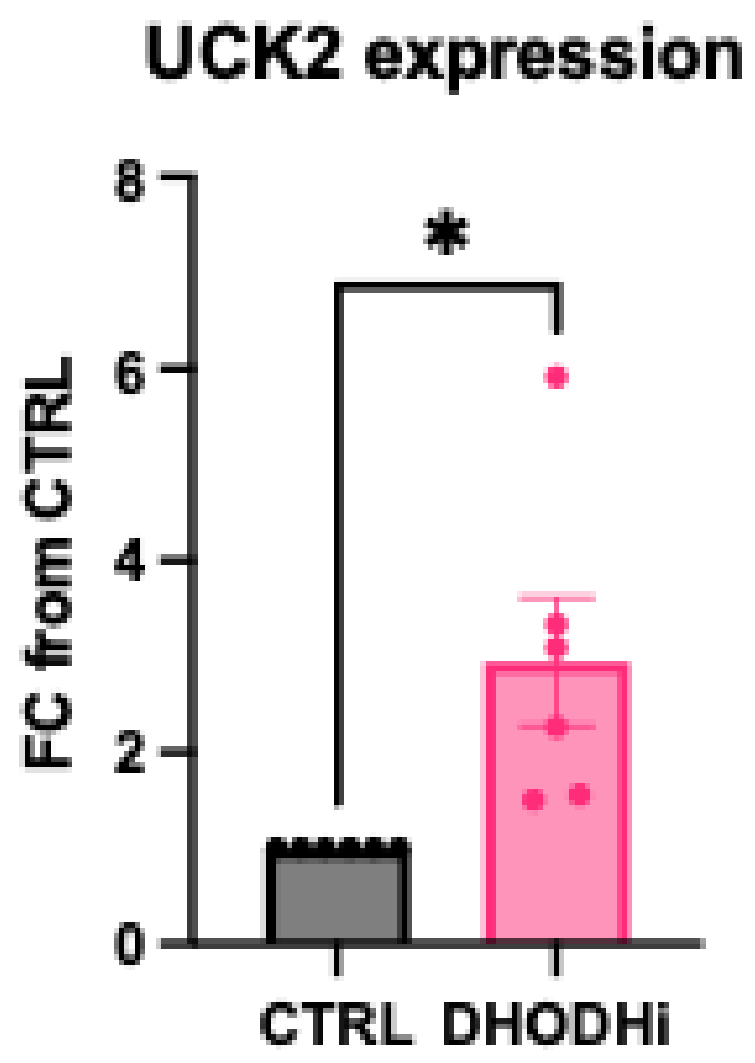

Table shows densitometry values for blots presented on slide 39, CTRL vs BAY-2402234 (DHODHi) for 72hrs, with raw values then normalised to Actin as loading and expressed as fold change over CTRL.

Graph shows expression fold changes as a function of CTRL (FC from CTRL) for each replicate ± sem, with statistical testing by t-test relative to CTRL.

WB panels confirming siRNA against UCK2 works to promote effective knockdown

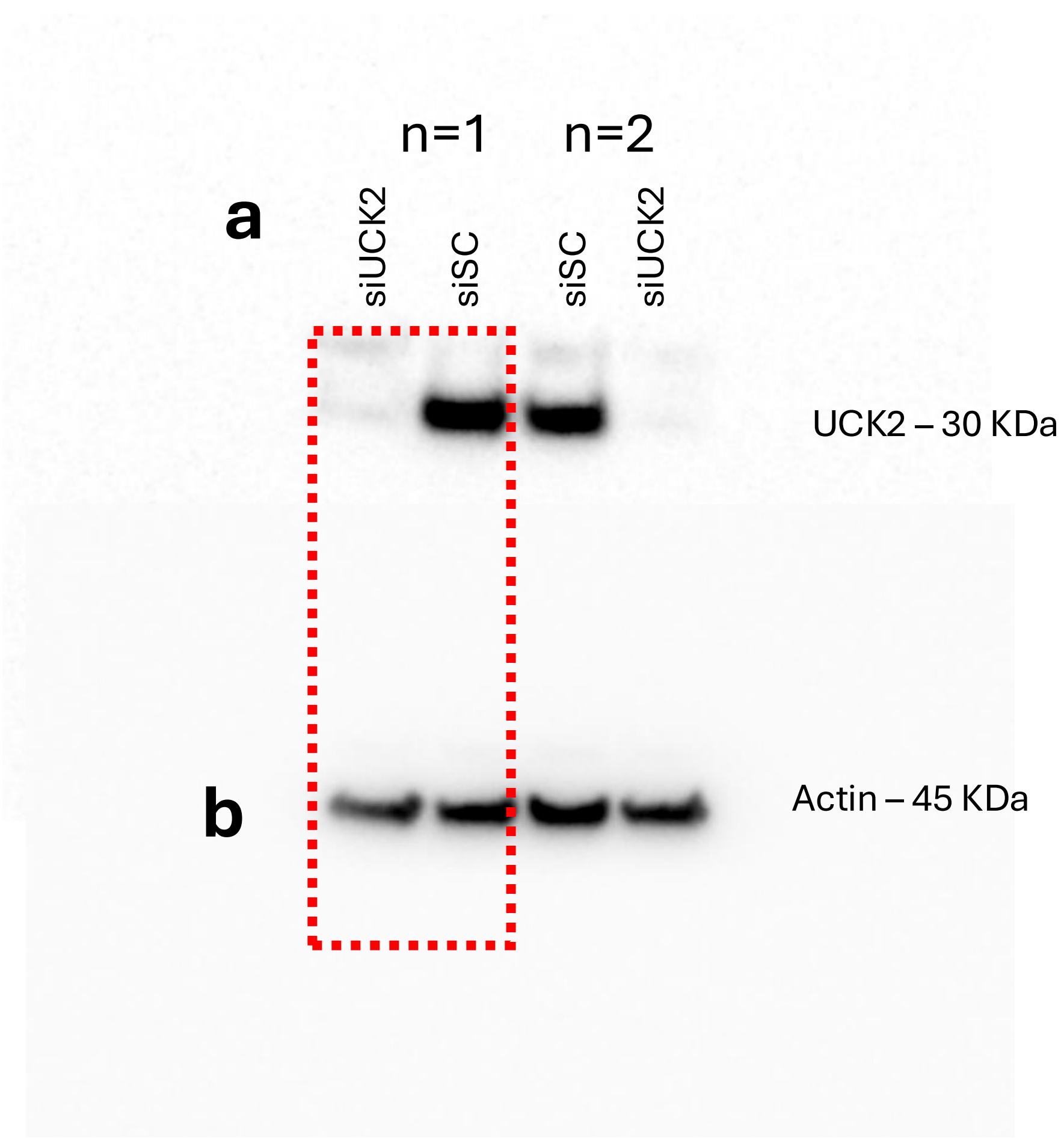

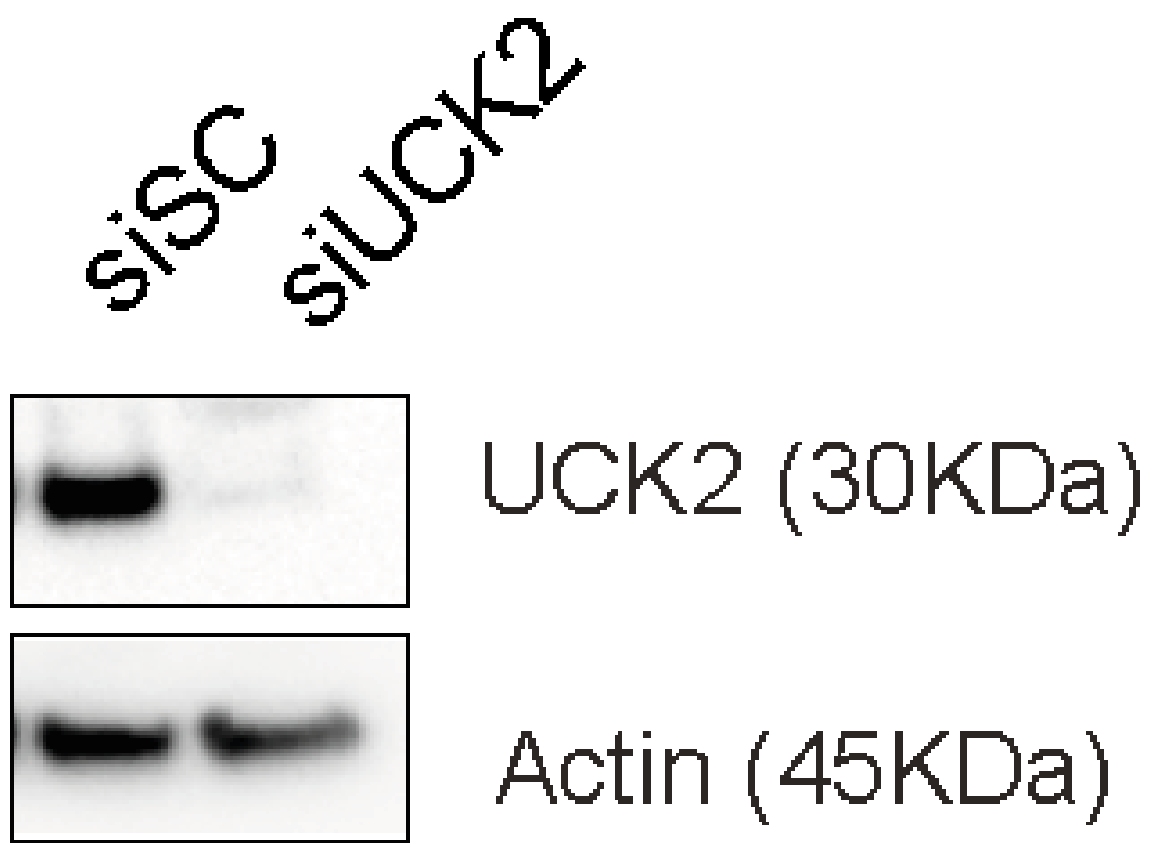

Blots show western images for HCT116 cells treated with siSC or siUCK2 for 72 hrs, blotted for UCK2 (a) and Actin (b) used as loading control. Size is indicated. n indicates biological replicate.
