# Supplementary material for: Mitochondrial metabolism determines chemotherapy sensitivity in colorectal cancer
Source: Nat Metab. 2026 Jul 23;8(8):1751–71. doi: 10.1038/s42255-026-01578-w (PMC13489958; doi:10.1038/s42255-026-01578-w)
Supplement: Supplementary file 5 [file 42255_2026_1578_MOESM21_ESM.pdf]

## *Extended Data Figure 2c: SW480 data*

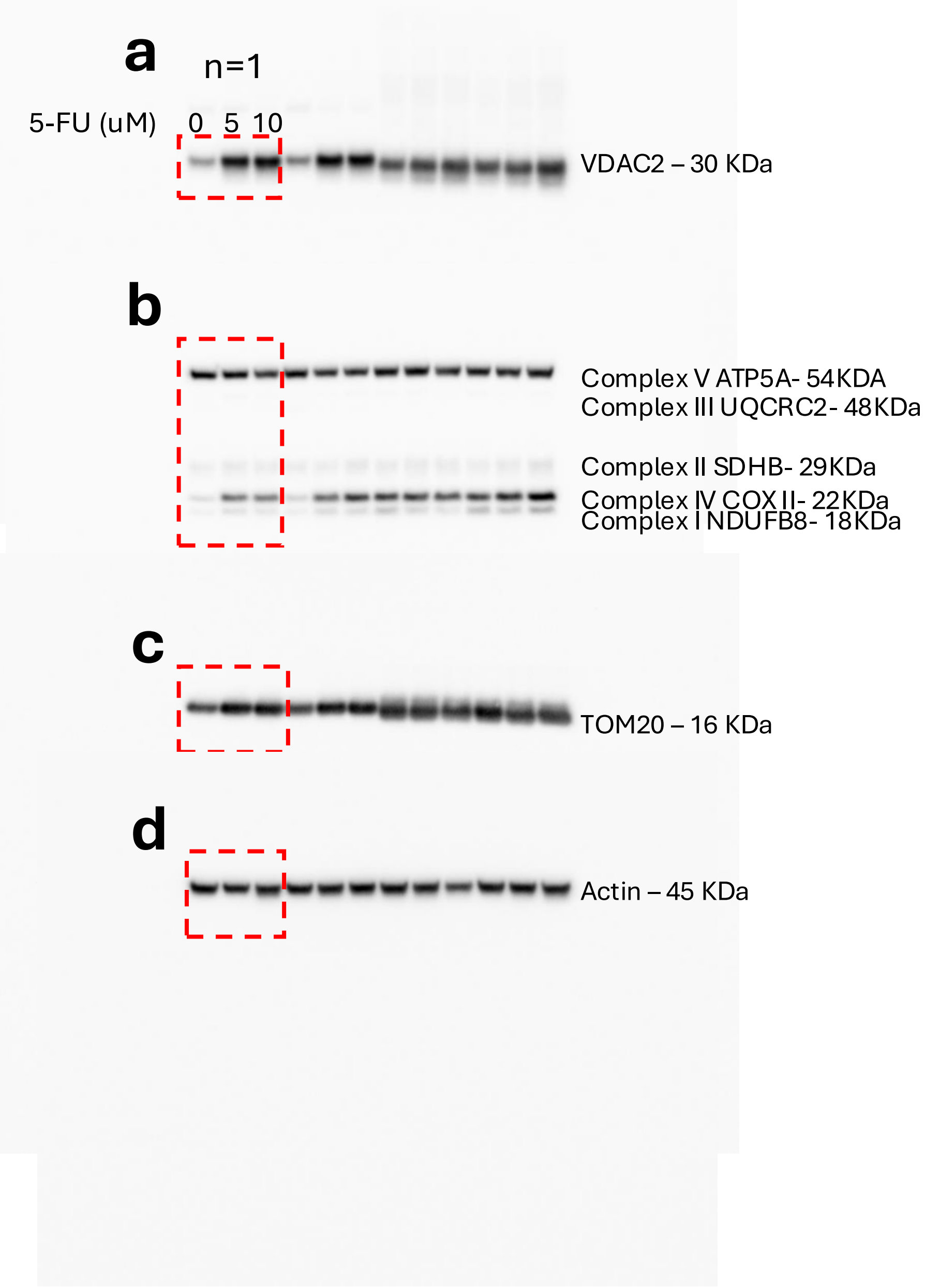


Blots show independent western images for SW480 cells treated ± 5 or 10uM 5FU for 72 hrs for VDAC2 (a), electron transport chain panel (b), TOM20 (c), Actin (d) used as a loading control. Size is indicated. Red box represents area used in figure panel.

***AKP organoids 3D;*** *data shows three independent biological replicates run, CTRL (-) and 5FU (+)*

Ext. Data Fig 2f

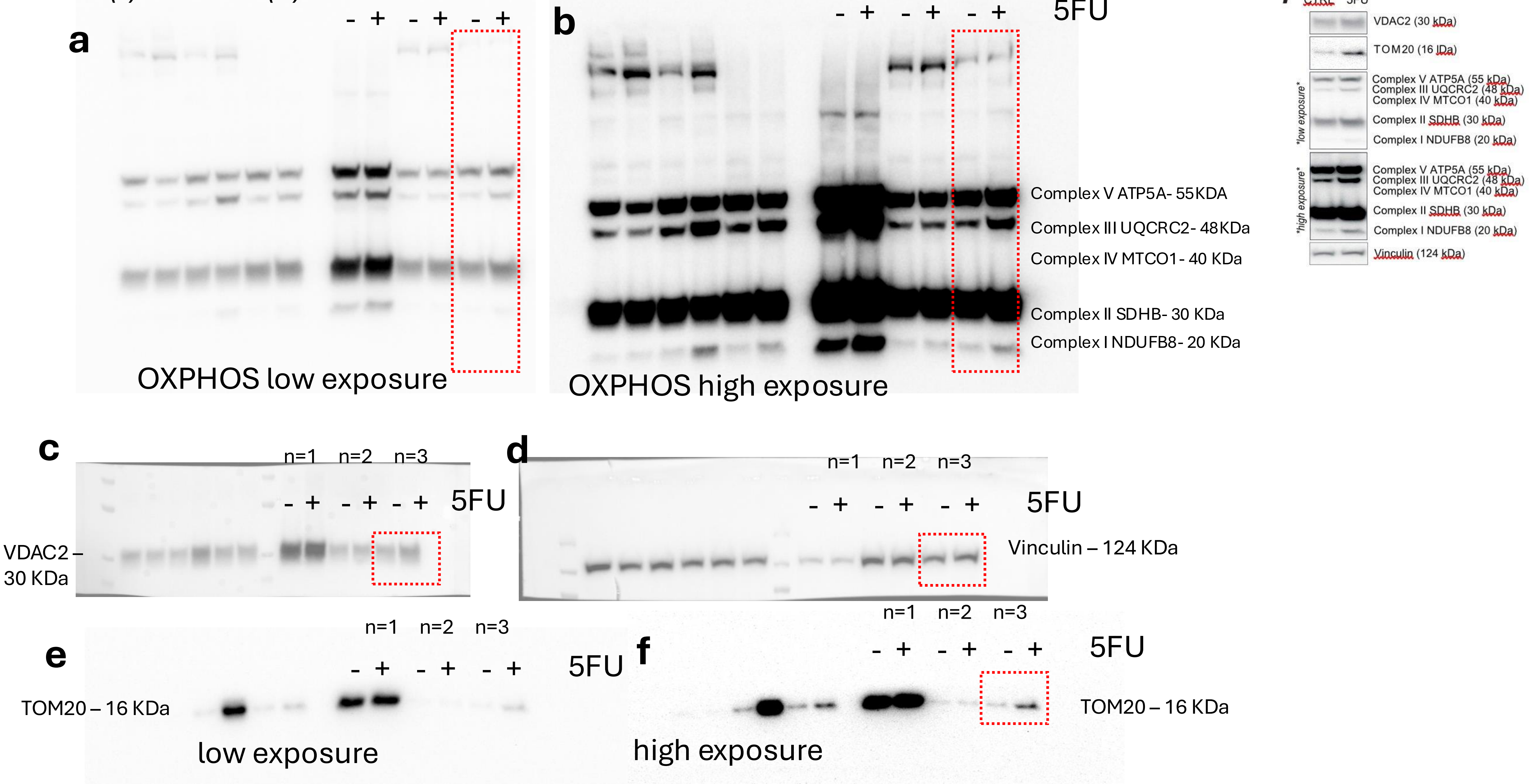


Blots show independent western images for AKP organoids in 3D culture treated ± 5FU for 72 hrs for electron transport chain panel (a, low exposure and b, high exposure), VDAC2 (c), Vinculin (d) used as loading control and TOM20 (e, low exposure and f, high exposure). Size is indicated. n indicates biological replicate.

***AKP cell line 2D;*** *data shows three independent biological replicates run; 0, 2 and 5uM 5FU at 72 hrs*

Ext. Data Fig 2h

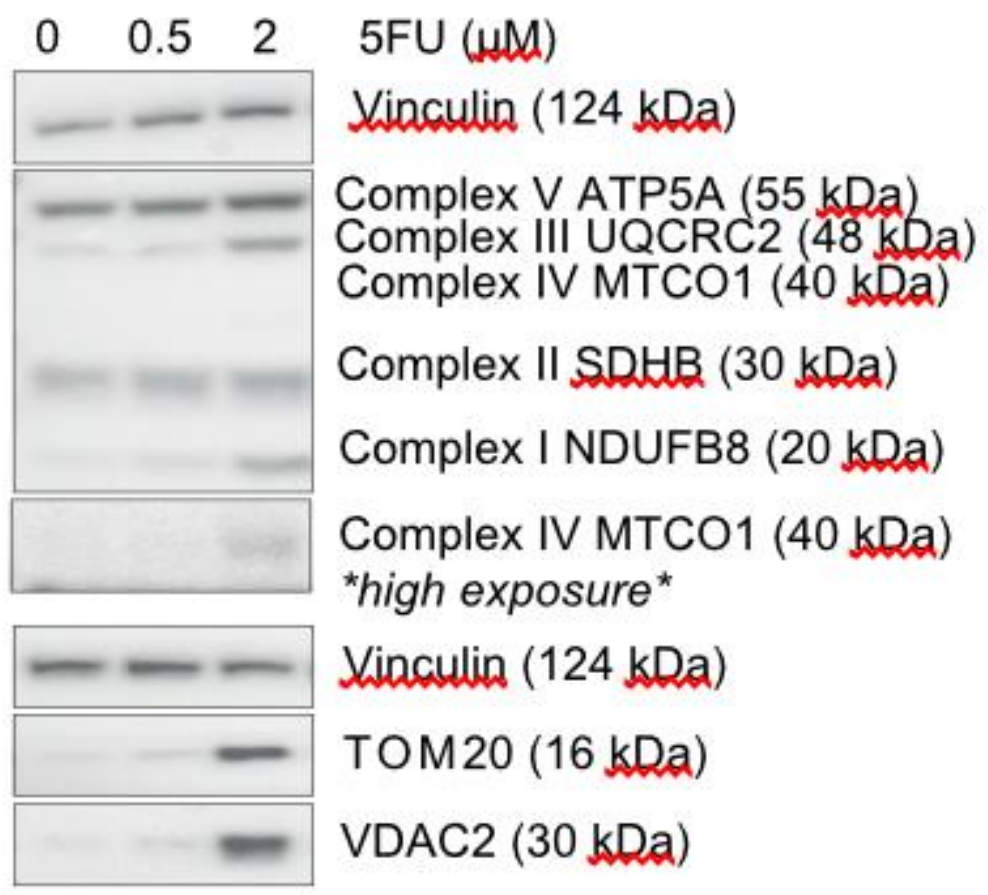


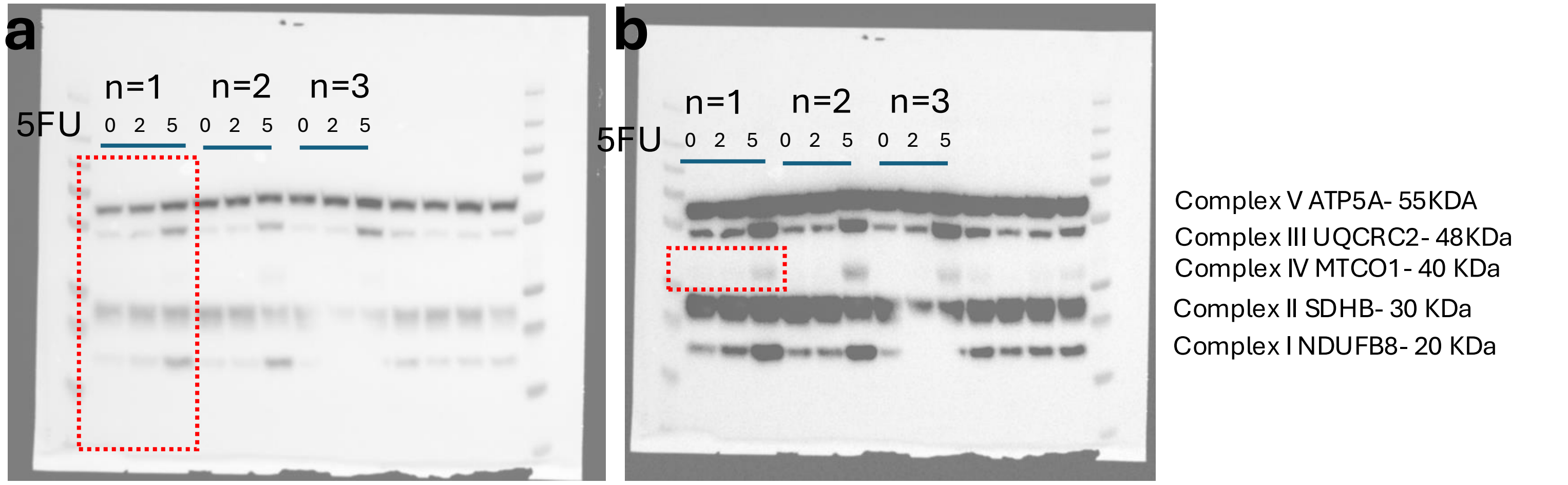


OXPHOS low and high exposure

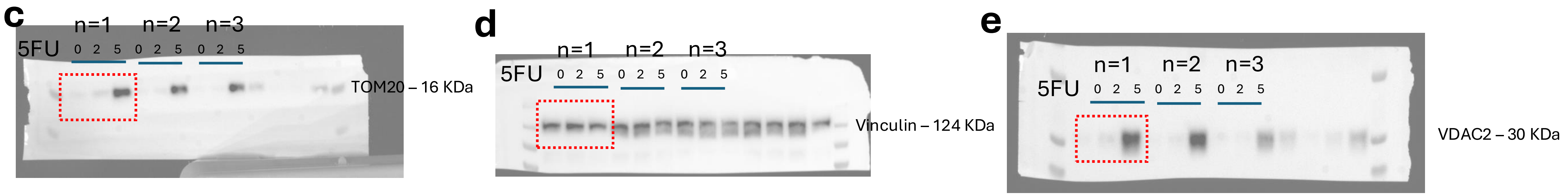


Blots show western images for three independent biological repeats of AKP cells in 2D culture treated ± 5FU for 72 hrs for electron transport chain panel (a, low exposure and b, high exposure), TOM20 (c), Vinculin (d) used as loading control and VDAC2 (e). Size is indicated. n indicates biological replicate.
