# Supplementary material for: Mitochondrial metabolism determines chemotherapy sensitivity in colorectal cancer
Source: Nat Metab. 2026 Jul 23;8(8):1751–71. doi: 10.1038/s42255-026-01578-w (PMC13489958; doi:10.1038/s42255-026-01578-w)
Supplement: Supplementary file 6 [file 42255_2026_1578_MOESM28_ESM.pdf]

Blots used for **ED Figure 9b**

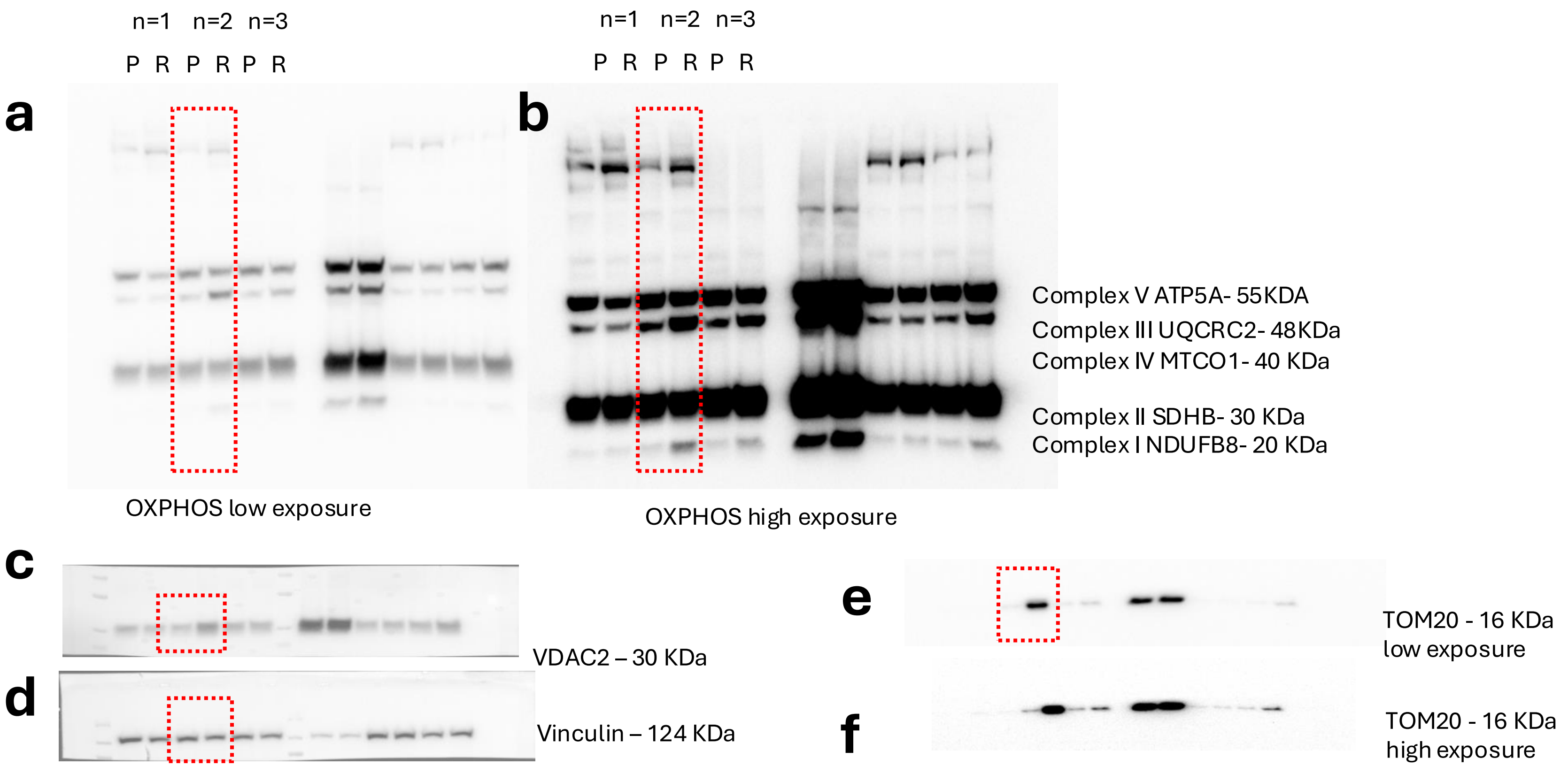

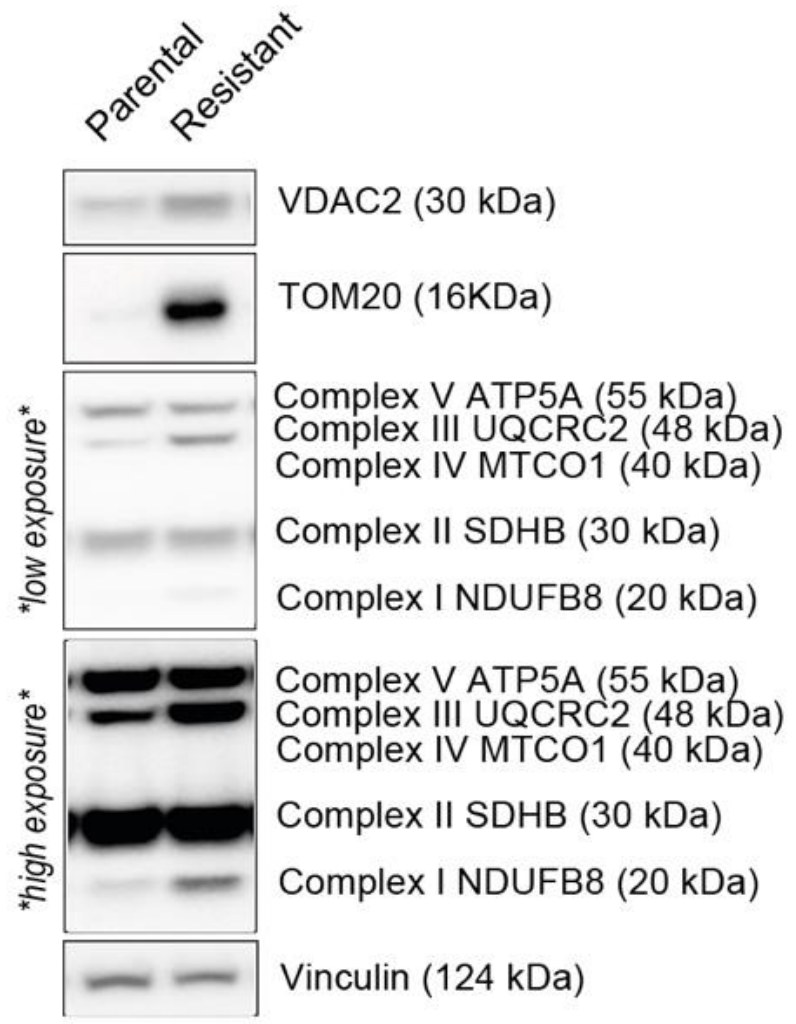

Blots show independent western images for Parental (P, 5FU sensitive) and Resistant (R, 5FU resistant) AKP organoids in 3D culture for electron transport chain panel (a, low exposure and b, high exposure), VDAC2 (c), Vinculin (d) used as loading control and TOM20 (e, low exposure and f, high exposure). Size is indicated. n indicates biological replicate. Red boxes indicate area used for figure.
