# Supplementary material for: Mitochondrial metabolism determines chemotherapy sensitivity in colorectal cancer
Source: Nat Metab. 2026 Jul 23;8(8):1751–71. doi: 10.1038/s42255-026-01578-w (PMC13489958; doi:10.1038/s42255-026-01578-w)
Supplement: Supplementary file 7 [file 42255_2026_1578_MOESM2_ESM.pdf]

nature portfolio

Corresponding author(s): E Kerr

Last updated by author(s): 2026/06/02

Reporting Summary

Nature Portfolio wishes to improve the reproducibility of the work that we publish. This form provides structure for consistency and transparency in reporting. For further information on Nature Portfolio policies, see our Editorial Policies and the Editorial Policy Checklist.

Statistics

For all statistical analyses, confirm that the following items are present in the figure legend, table legend, main text, or Methods section.

n/a	Confirmed	
☐	☒	The exact sample size (*n*) for each experimental group/condition, given as a discrete number and unit of measurement
☐	☒	A statement on whether measurements were taken from distinct samples or whether the same sample was measured repeatedly
☐	☒	The statistical test(s) used AND whether they are one- or two-sided<br>*Only common tests should be described solely by name; describe more complex techniques in the Methods section.*
☒	☐	A description of all covariates tested
☐	☒	A description of any assumptions or corrections, such as tests of normality and adjustment for multiple comparisons
☐	☒	A full description of the statistical parameters including central tendency (e.g. means) or other basic estimates (e.g. regression coefficient) AND variation (e.g. standard deviation) or associated estimates of uncertainty (e.g. confidence intervals)
☐	☒	For null hypothesis testing, the test statistic (e.g. *F*, *t*, *r*) with confidence intervals, effect sizes, degrees of freedom and *P* value noted<br>*Give P values as exact values whenever suitable.*
☒	☐	For Bayesian analysis, information on the choice of priors and Markov chain Monte Carlo settings
☒	☐	For hierarchical and complex designs, identification of the appropriate level for tests and full reporting of outcomes
☐	☒	Estimates of effect sizes (e.g. Cohen's *d*, Pearson's *r*), indicating how they were calculated

Our web collection on statistics for biologists contains articles on many of the points above.

Software and code

Policy information about availability of computer code

Data collection: No software was used

Data analysis:
STAR
DESeq2
ggplot (R package)
clusterProfiler (R package)
msigdbr (R package)
Morpheus (https://software.broadinstitute.org/morpheus/)
ImageJ
Graphpad Prism
QuPath

For manuscripts utilizing custom algorithms or software that are central to the research but not yet described in published literature, software must be made available to editors and reviewers. We strongly encourage code deposition in a community repository (e.g. GitHub). See the Nature Portfolio guidelines for submitting code & software for further information.

Data

Policy information about availability of data

All manuscripts must include a data availability statement. This statement should provide the following information, where applicable:

- Accession codes, unique identifiers, or web links for publicly available datasets
- A description of any restrictions on data availability
- For clinical datasets or third party data, please ensure that the statement adheres to our policy

The raw sequencing data files generated in this study have been deposited in the NCBI Gene Expression Omnibus (GEO) under the accession numbers GSE272391 (https://www.ncbi.nlm.nih.gov/geo/query/acc.cgi?acc=GSE272391) and GSE272456 (https://www.ncbi.nlm.nih.gov/geo/query/acc.cgi?acc=GSE272456). The Taxonomy data is from GEO under the accession number GSE103479.

Research involving human participants, their data, or biological material

Policy information about studies with human participants or human data. See also policy information about sex, gender (identity/presentation), and sexual orientation and race, ethnicity and racism.

Reporting on sex and gender	Human data was analysed from previously published studies (GSE103479). Data was split based on treatment and the adjuvant FP-based chemotherapy cohort analysed. This contained data from 35 male and 31 female participants. Sex was determined based on metadata provided to ensure even distributions. Sex was not considered within the analyses as sample size was too small to provide statistically meaningful results.
Reporting on race, ethnicity, or other socially relevant groupings	n/a
Population characteristics	n/a
Recruitment	n/a
Ethics oversight	n/a

Note that full information on the approval of the study protocol must also be provided in the manuscript.

Field-specific reporting

Please select the one below that is the best fit for your research. If you are not sure, read the appropriate sections before making your selection.

☒ Life sciences ☐ Behavioural & social sciences ☐ Ecological, evolutionary & environmental sciences

For a reference copy of the document with all sections, see nature.com/documents/nr-reporting-summary-flat.pdf

Life sciences study design

All studies must disclose on these points even when the disclosure is negative.

Sample size	In vitro experiments were preformed as stated, with all but mass spec metabolomics and transcriptomics data preformed in biolgoically independent experiments of n=3 or more. In vivo work was completed using a minimum sample size on 5 independent tumours, with power calculations (80% power, 0.05 significance, G power software) based on pilot data and cleaved caspase 3 staining used to inform readouts.
Data exclusions	in vitro: One technical replicate was excluded from qPCR for AKP1 Aldoc as had failed during qPCR run (ext data figure 4e)<br>in vivo: one animal was excluded from Tom20/Vdac2 analysis based on unusually high DAB staining across all images analysed
Replication	Reproducibility was confirmed by independent biological replicates. Where possible/relevant all biological replicates are shown. RNASeq data was not replicated in human cell models, but was validated by qPCR in 3D organoid models. LCMS metabolomics and RNASeq used a minimum of 3 independent psudeo-biological replicates. All in vivo data was analysed using digital pathology software algorithms.
Randomization	All animals were randomised into treatment groups based on tumour burden (presence/absence/size) before treatment began.
Blinding	For mitochondrial scoring (IHC, network analyses) all counter-scoring/quantification was preformed blind. Investigators were not blinded for in vitro analysis. All LCMS analysis was preformed by independent experimentators and RNASeq was completed by CTU. Investigators were not blinded for in vivo analysis due to skill set required and small team size, but all analysis were completed by two indpendent operators to ensure reproducibility of findings.

Reporting for specific materials, systems and methods

We require information from authors about some types of materials, experimental systems and methods used in many studies. Here, indicate whether each material, system or method listed is relevant to your study. If you are not sure if a list item applies to your research, read the appropriate section before selecting a response.

Materials & experimental systems

n/a	Involved in the study
☐	☒ Antibodies
☐	☒ Eukaryotic cell lines
☒	☐ Palaeontology and archaeology
☐	☒ Animals and other organisms
☐	☒ Clinical data
☒	☐ Dual use research of concern
☒	☐ Plants

Methods

n/a	Involved in the study
☒	☐ ChIP-seq
☐	☒ Flow cytometry
☒	☐ MRI-based neuroimaging

Antibodies

Antibodies used

The following antibodies
were used: Total OxPhos Human Antibody Cocktail (Abcam, ab110411; 1:500), Total OxPhos Rodent Antibody Cocktail (Abcam, ab110413; 1:250), rabbit anti-TOMM20 (Abcam, ab186734; 1:1000), rabbit anti-VDAC2 (ThermoFisher, PA5-28106; 1:1000), rabbit anti-OPA1 (Cell Signalling Technology, 80471; 1:1000), rabbit anti-DRP1 (Cell Signalling Technology, 8570; 1:1000), rabbit anti-PGC1a (Cell Signalling Technology, 2178S; 1:1000) rabbit anti-MFN2 (Cell Signalling Technology, 9482S; 1:1000), rabbit anti914 AMPK pThr172 (Cell Signalling Technology, 50081; 1:1000), rabbit anti-AMPK (Cell Signalling Technology, 5831S; 1:1000) rabbit anti-Vinculin (Cell Signalling Technology, 13901S; 1:1000), rabbit anti-ACC pSer79 (Cell Signalling Technology, 3661S; 1:1000), rabbit anti-ACC (Cell Signalling Technology, 3676S; 1:1000), rabbit anti-p70 S6 Kinase pThr389 (Cell Signalling Technology, 9205S; 1:1000), rabbit anti- p70 S6 Kinase (Cell Signalling Technology, 2708S; 1:1000), rabbit anti- S6 Ribsosomal Protein pSer240/244 (Cell Signalling Technology, 5364S; 1:1000), rabbit anti- S6 Ribsosomal Protein (Cell Signalling Technology, 2217L; 1:1000),rabbit anti Cytochrome C- (Abcam, ab65311; 1:1000), rabbit anti- DHODH (Proteintech, 14877-1-AP; 1:1000), rabbit anti-UCK2 (Proteintech, 10511-1-AP; 1:1000), rabbit anti-UPP1 (Cell signalling Technology, 48789S; 1:1000), mouse anti-Lamin B (Santa Cruz Biotechnology sc-374015; 1:1000) rabbit anti-Thymidylate Synthase (Cell Signalling Technology, 9045S; 1:1000), mouse anti-GAPDH (Santa Cruz Biotechnology, sc-47724; 1:10,000) and mouse anti-β-actin (Sigma-Aldrich, A5316; 1:10,000).

Validation

All antibodies used were chosen on the basis they had either been validated in house by the manufacturer in knock experiments or on the basis that they had been cited multiple times for use in human and murine models for the intended purpose be that immuno blotting, IHC or immunofluorescence.

Eukaryotic cell lines

Policy information about cell lines and Sex and Gender in Research

Cell line source(s)

Human cell models used: HCT116 CRC cell line was obtained from ATCC. Other cells were provided by co-authors or collaborators: (SW480, HT29: McDade/Longley; U2OS: Butterworth, QUB; hMSC: Ryan; 143b mitochondrial cybrids: Greaves). Murine cell models used: AKP organoids were provided by Sansom lab or generated in house as detailed in manuscript and references.

Authentication

Murine cell models were not authenticated, but were not used >30 passages from isolation. Human HCT116 cells were freshly purchased stocks. Other cell lines were provided by collaborators: SW480 and HT29 cells were authenticated by STR profiling, U2OS cells were not authenticated.

Mycoplasma contamination

Mycoplasma screening was performed routinely and all cell lines tested negative for mycoplasma.

Commonly misidentified lines
(See ICLAC register)

N/A

Animals and other research organisms

Policy information about studies involving animals; ARRIVE guidelines recommended for reporting animal research, and Sex and Gender in Research

Laboratory animals

Xenograft studies: athymic nude mice purchased from commercial suppliers in UK at 6-8 weeks old, and studies started >1 week post arrival. GEMM studies used two strains: VillinCreERT2;ApcFx/+;KrasG12D/+;p53Fx/Fx and VillinCreERT2;ApcFx/Fx;KrasG12D/+;p53Fx/Fx; both on a C57BL/6 background, bred in house, with tumour induction typically starting at 8-10 weeks old.

Wild animals

No wild animals were used in this study.

Reporting on sex	Sex was not reported on as a factor as all GEMM experiments were balanced for both sexes, but experiments were analysed with sex considered as a blocking factor where appropriate. Athymic mice were all female. All GEMM experiments used a mix of both sexes. Sex was determined at weaning based on examination.<br>Figure 1h 8F/5M; Figure 3b 8F/5M; Figure 6e 9F/7M; Figure 6g 6F/5M
Field-collected samples	N/A
Ethics oversight	Animals were maintained under specific-pathogen-free (SPF) conditions and experiments completed in compliance with the NI Department of Health (under PPL #2874 and #2960) regulations with approval from Queen's University Belfast (QUB) Animal Welfare and Ethical Review Body (AWERB).

Note that full information on the approval of the study protocol must also be provided in the manuscript.

Clinical data

Policy information about clinical studies

All manuscripts should comply with the ICMJE guidelines for publication of clinical research and a completed CONSORT checklist must be included with all submissions.

Clinical trial registration	N/A
Study protocol	N/A
Data collection	N/A
Outcomes	N/A

Plants

Seed stocks	N/A
Novel plant genotypes	N/A
Authentication	N/A

Flow Cytometry

Plots

Confirm that:

☒ The axis labels state the marker and fluorochrome used (e.g. CD4-FITC).

☒ The axis scales are clearly visible. Include numbers along axes only for bottom left plot of group (a 'group' is an analysis of identical markers).

☐ All plots are contour plots with outliers or pseudocolor plots.

☐ A numerical value for number of cells or percentage (with statistics) is provided.

Methodology

Sample preparation	Cells were stained with Acridine Orange 10-nonyl bromide (NAO; ThermoFisher, A1372), Tetramethylrhodamine, Ethyl Ester Perchlorate (TMRE; Sigma-Aldrich, 87917) and MitoSOX Red (ThermoFisher, M36008). NAO (200nM) or TMRE (25 nM) were added to the culture medium and incubated for 1 hr at 37°C in 5% CO2. MitoSOX Red (5 μM) was added to the medium and incubated for 30min at 37°C in 5% CO2. Cells were washed, collected and resuspended in 0.3ml of PBS. Cells were analysed by flow cytometry using a BD Accuri C6 nstrument. Cells were gated for singlets and the GeoMean NAO, TMRE or MitoSOX Red fluorescence was evaluated using FlowJo software.
Instrument	BD Accuri C6 and LSRII instruments were used
Software	Data was collected using BD softwares, all fcs files were analysed in FlowJo
Cell population abundance	All experiments collected a minimum of 10000 events within the gating strategy.

Gating strategy

Cell populations were identified using FSC/SSC. Singlets were determined by FSC-H/FSC-A.

☒ Tick this box to confirm that a figure exemplifying the gating strategy is provided in the Supplementary Information.
