# Supplementary material for: Mitochondrial metabolism determines chemotherapy sensitivity in colorectal cancer
Source: Nat Metab. 2026 Jul 23;8(8):1751–71. doi: 10.1038/s42255-026-01578-w (PMC13489958; doi:10.1038/s42255-026-01578-w)
Supplement: Supplementary file 8 [file 42255_2026_1578_MOESM8_ESM.pdf]

Blots used for image panel in **Figure 1c**

a

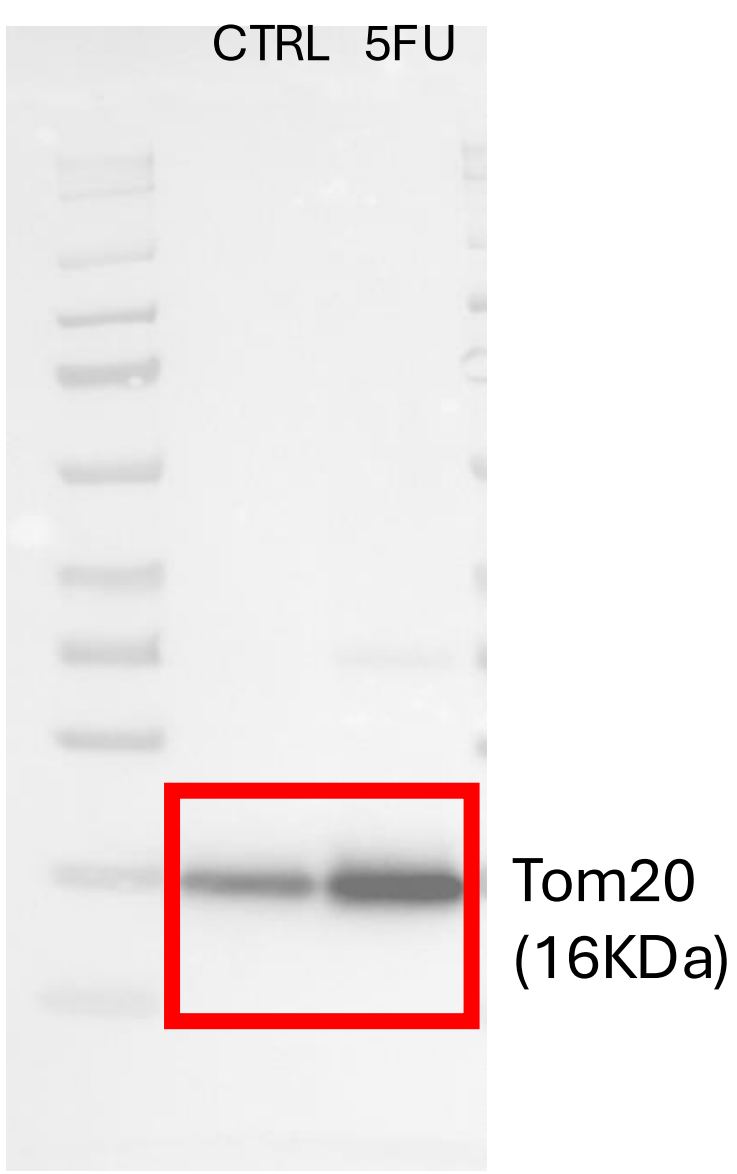

b

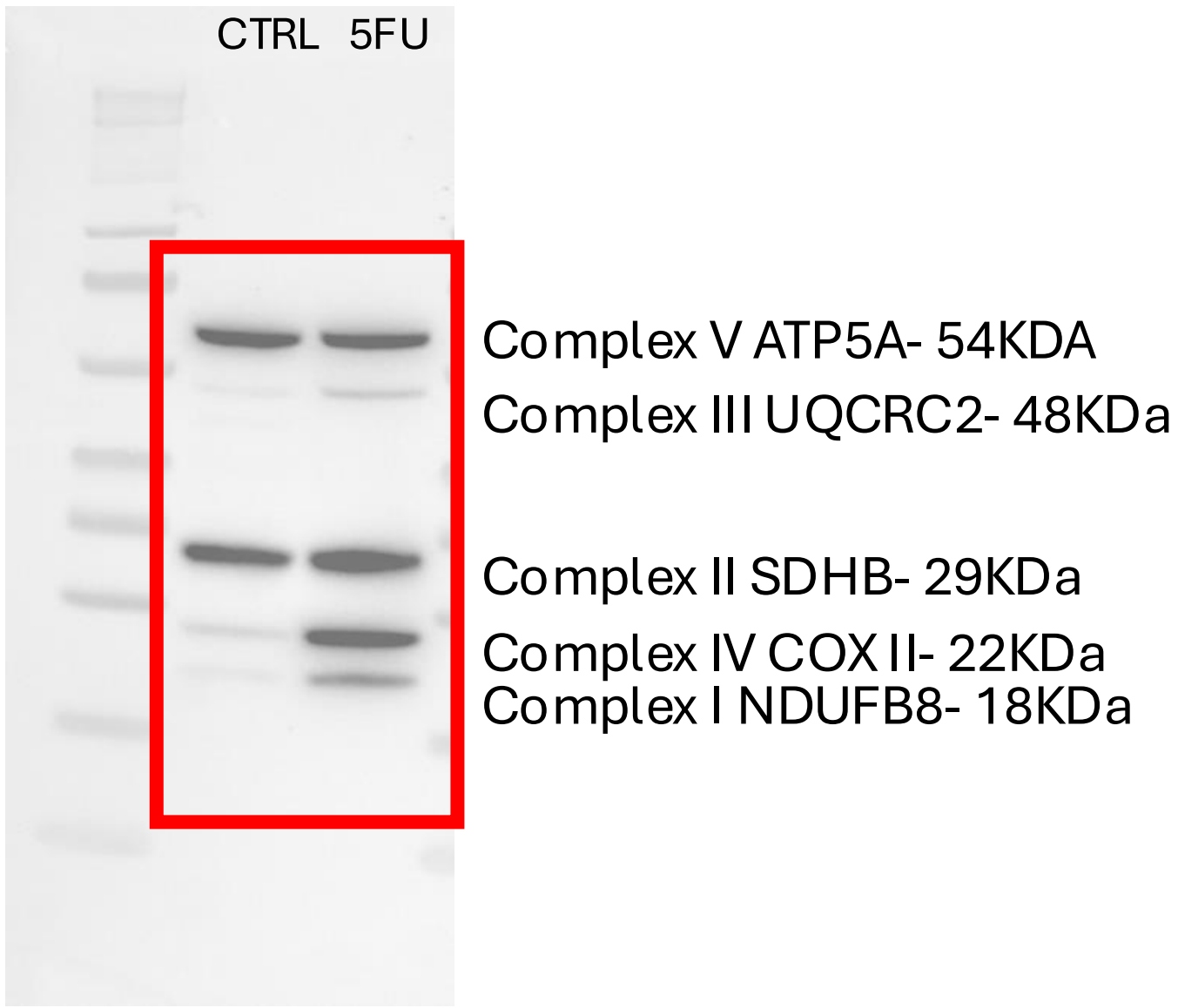

c

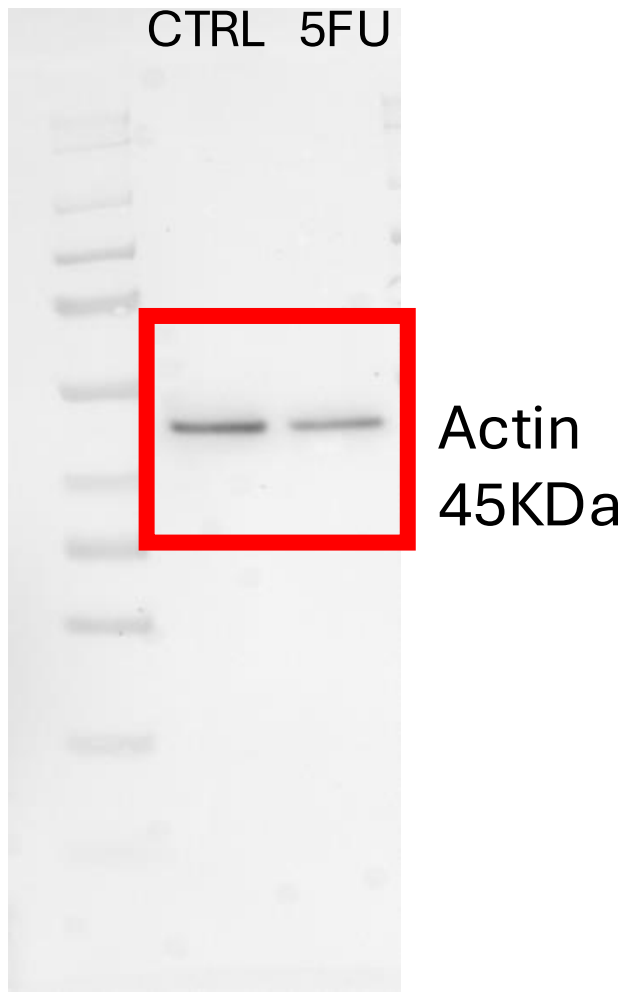

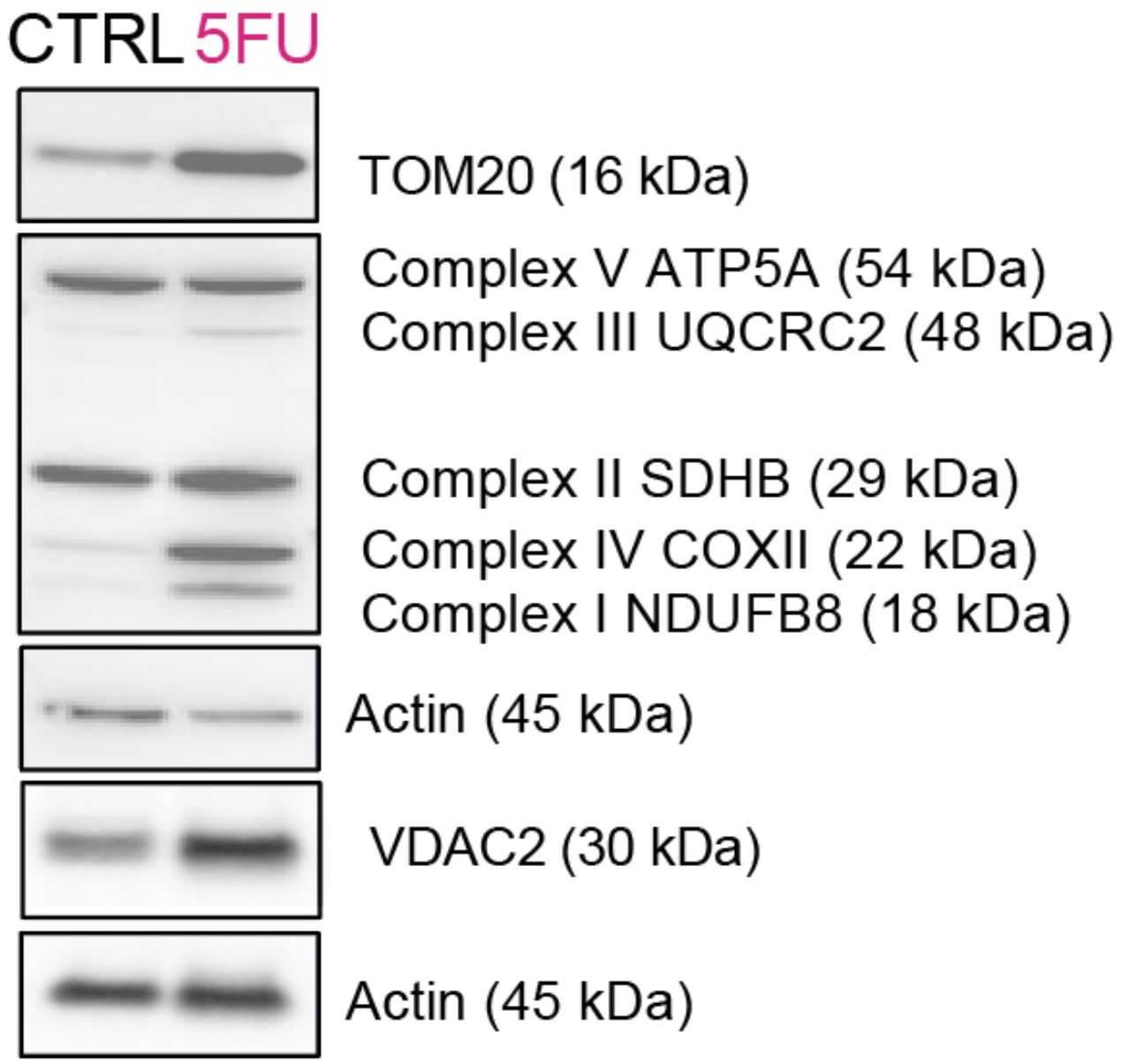

d

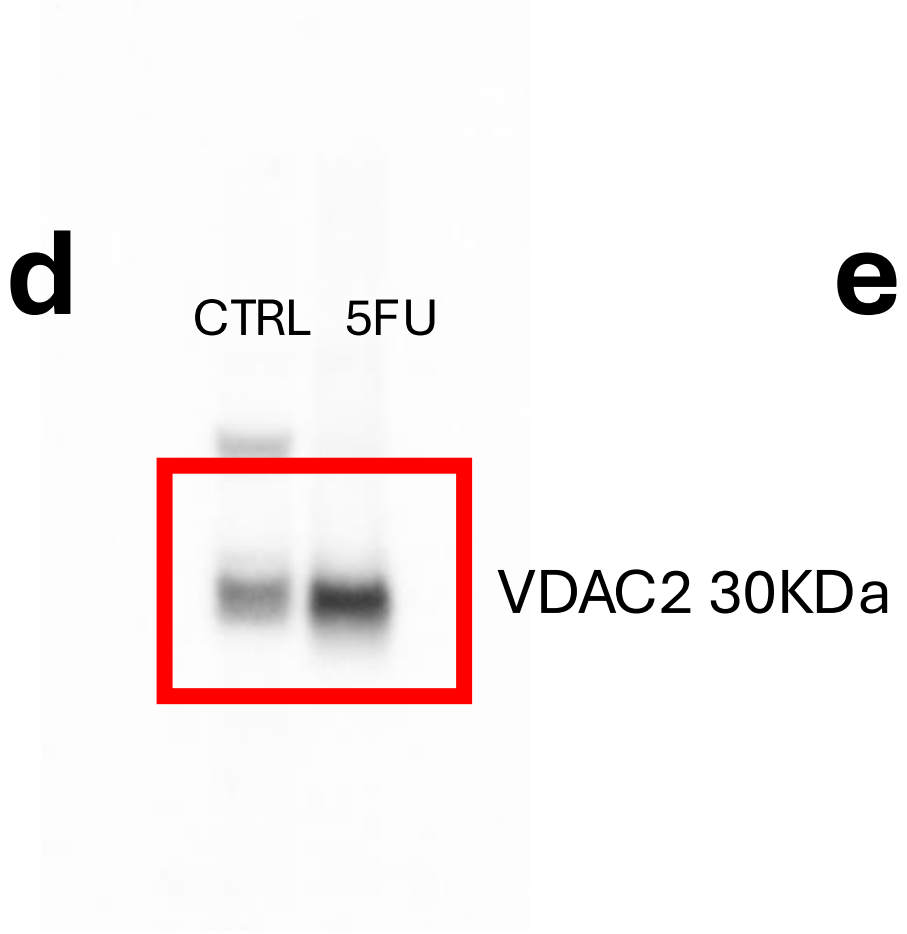

e

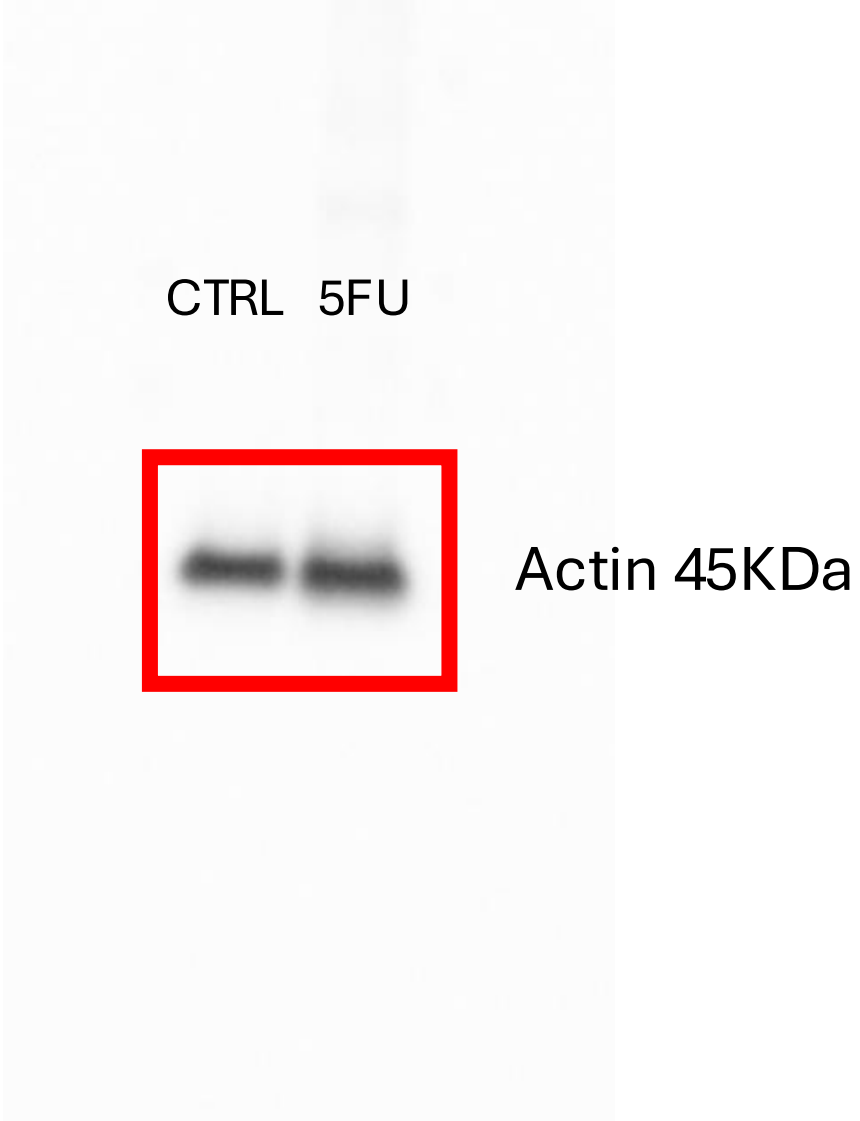

Source blot images for HCT116 cells treated ± 5FU (5 uM, 72 hrs) for Tom20 (a), electron transport chain antibody panel (b), Actin used as loading control (c), or VDAC2 (d) and Actin (e). Size is indicated, and red boxes denote area used for paper figure.

Blots used for image panel in **Figure 1f**

a

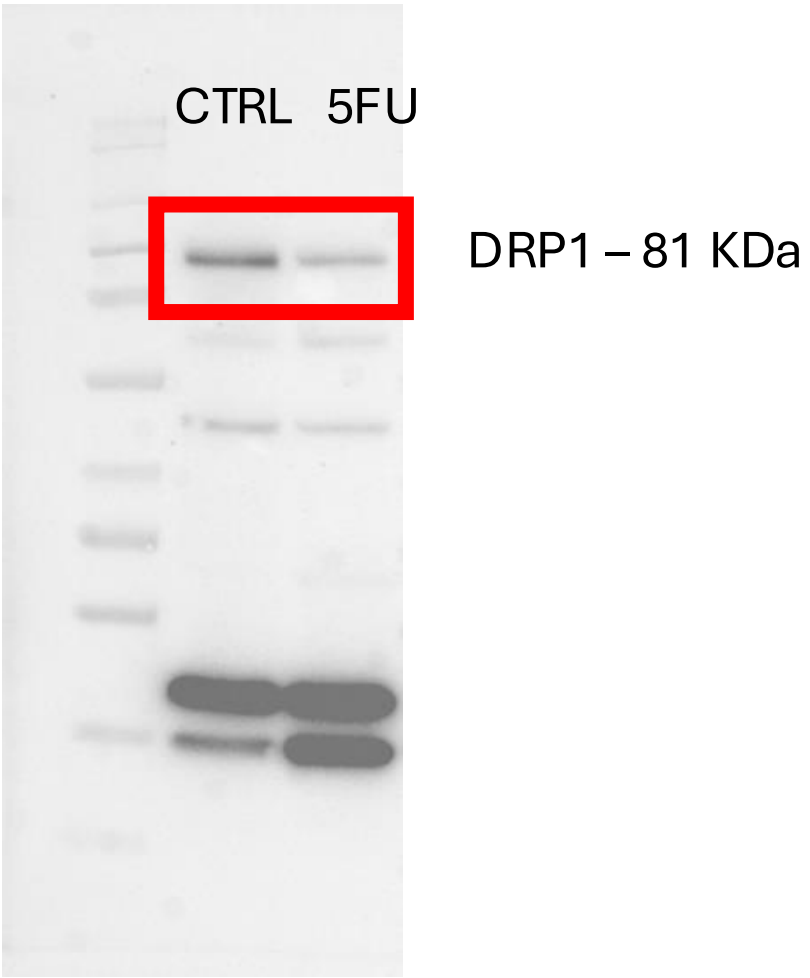

b

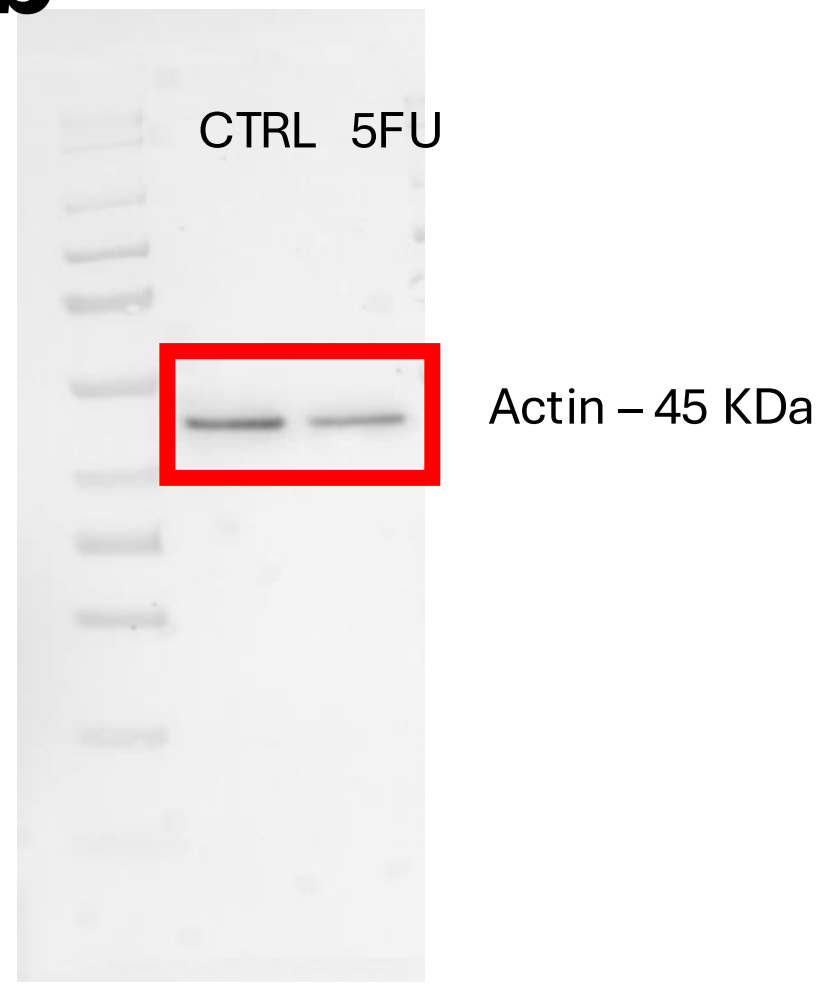

c

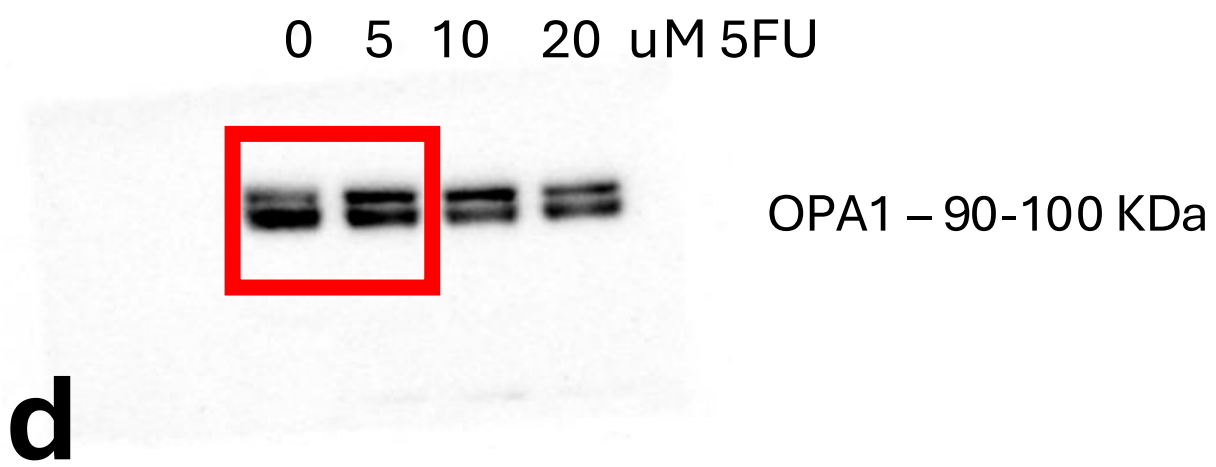

d

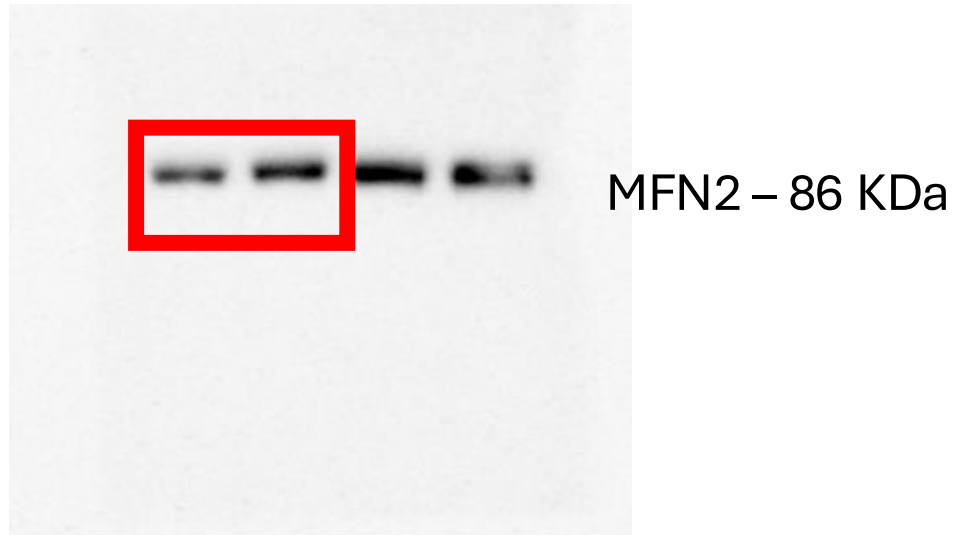

e

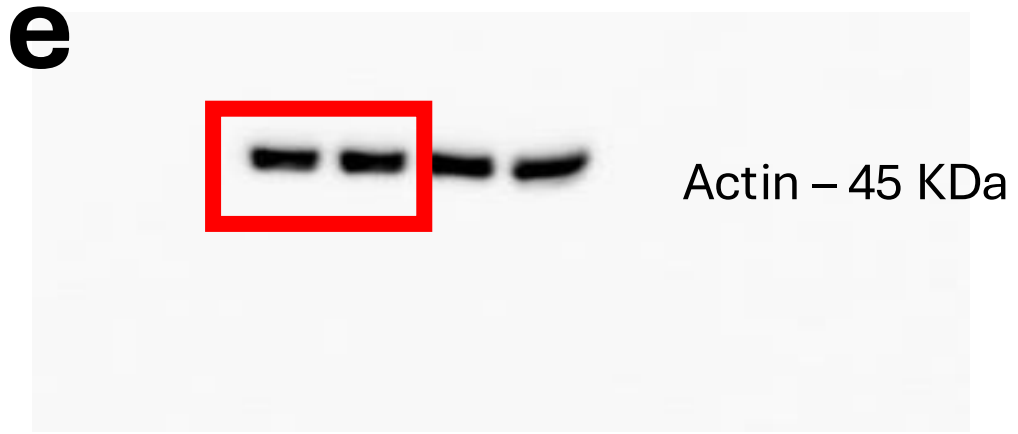

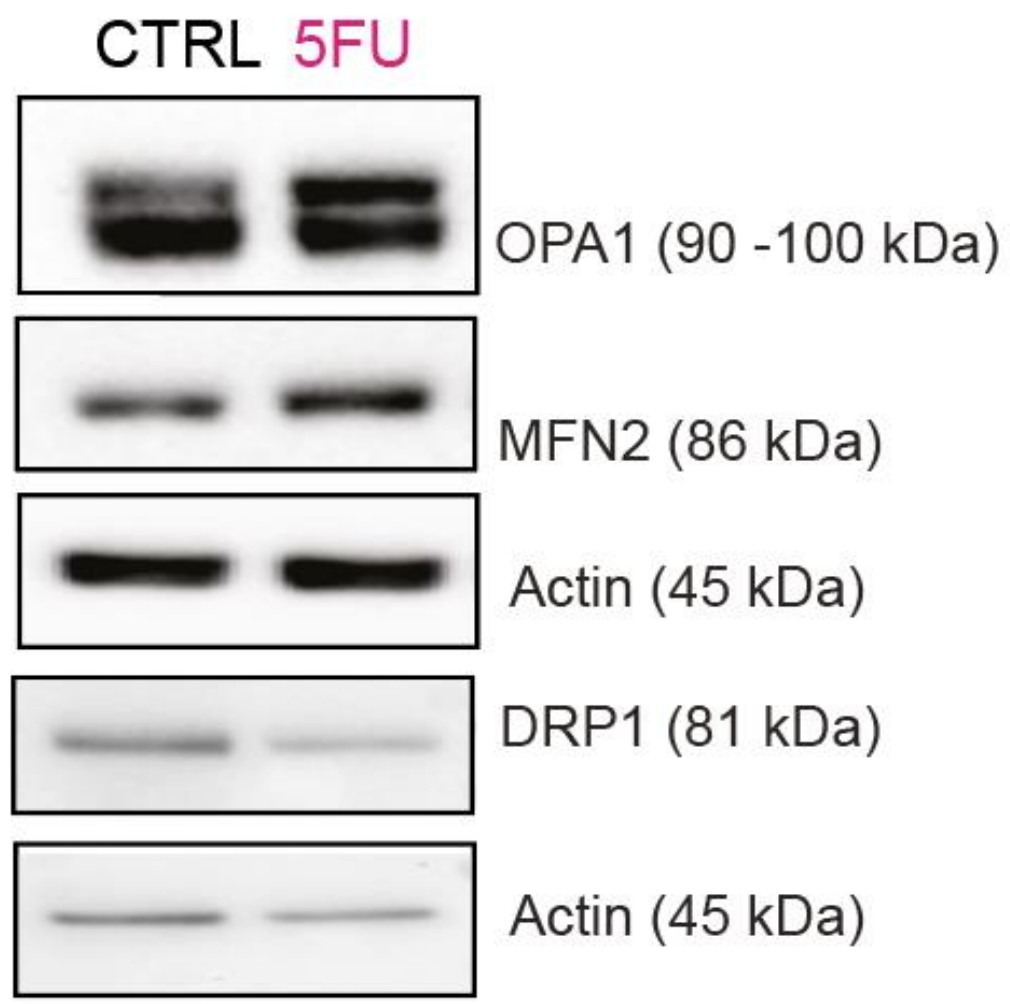

Source blot images for HCT116 cells treated ± 5FU (5 uM, 72 hrs) for DRP1 (a), with Actin used as loading control (b), or ± 5FU (72 hrs) for OPA1 (c), MFN2 (d) and Actin (e). Size is indicated, and red boxes denote area used for paper figure.
